# Supplementary material for: Genetic diversity of collaborative cross mice enables the establishment of a novel Chlamydia muridarum female genital tract infection model
Source: Infect Immun. 2026 May 5;94(6):e00746-25. doi: 10.1128/iai.00746-25 (PMC13248643; doi:10.1128/iai.00746-25)
Supplement: Fig. S1 — Chlamydial burden and infection clearance across all CC mouse strains inoculated with C. muridarum CM006. [file iai.00746-25-s0001.pdf]

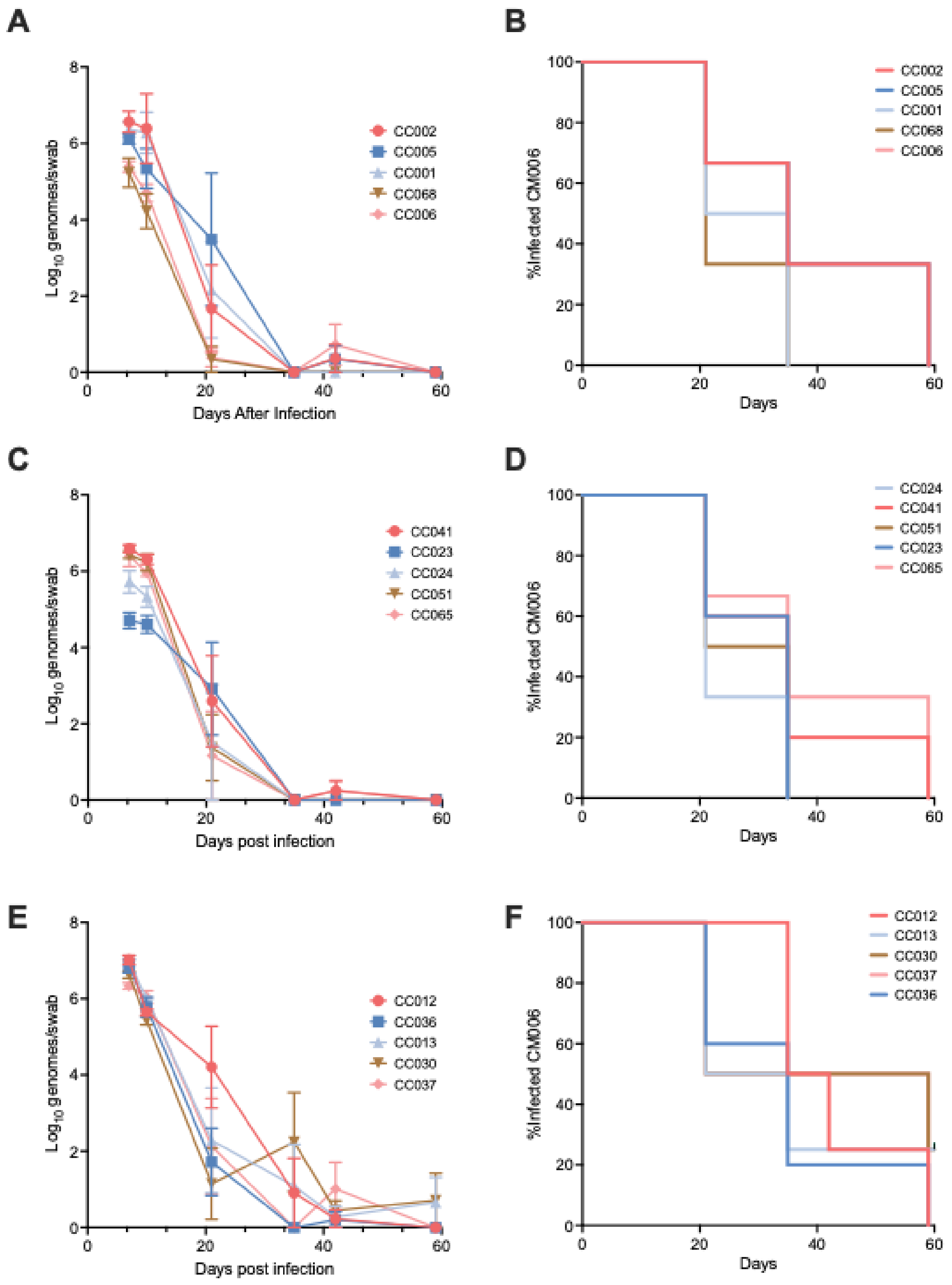

Supplementary Figure 1. Chlamydial burden and infection clearance across all CC mouse strains inoculated with *C. muridarum* CM006. Lower genital tract shedding was quantified by qPCR targeting the 23S rRNA locus. Panels A, C, and E show mean bacterial burden ± SEM for each CC strain. Infection clearance (B, D, F) was defined as the first day on which chlamydial genomic DNA fell below the limit of detection ($<8.3 \times 10^1$ genomes/swab). All qPCR reactions were performed in triplicate.

Panels represent groupings of CC strains by cohort size:
(A, B) CC strains with 3–6 mice/group: CC001 (n=4), CC002 (n=3), CC005 (n=3), CC006 (n=6), CC068 (n=3).
(C, D) CC strains with 3–5 mice/group: CC023 (n=5), CC024 (n=3), CC041 (n=5), CC051 (n=4), CC065 (n=3).
(E, F) CC strains with 4–5 mice/group: CC012 (n=4), CC013 (n=4), CC030 (n=4), CC036 (n=5), CC037 (n=4).
