# Supplementary material for: Genetic diversity of collaborative cross mice enables the establishment of a novel Chlamydia muridarum female genital tract infection model
Source: Infect Immun. 2026 May 5;94(6):e00746-25. doi: 10.1128/iai.00746-25 (PMC13248643; doi:10.1128/iai.00746-25)
Supplement: Supplemental material — Tables S1 to S5. [file iai.00746-25-s0002.pdf]

Supplementary Table 1. Host and chlamydial gene probes used for cervical transcriptional profiling in this study

Official Symbol	Accession	Alias/Previous Symbol	Target Sequence
Selected host targets			
Ccl11	NM_011330.3	eotaxin, Scya11	CAACCTCCTCTCTTGACACTAACCCAGAGCCTAAGAACTGCTTGATTCCTTCTCTTTCCTAAGACGTGCTCTGAGGGAATATCAGCACCAGTCGCCCAAG
Ccl19	NM_011888.2	exodus-3, Scya19, CKb11	CTTCTGCCAAGAACAAAGGCAACAGCACCAGAAGGAGCCCTGTGTCTTGAGTAAAGAGATGTGAATCACTCTGGCCCAGGAAACCAAGGACCAGAAGAGA
Ccl2	NM_011333.3	monocyte chemotactic protein, MCP-1, monocyte chemoattractant protein-1, Sigje, MCAF, SMC-CF, HC11, MCP1, Scya2	TCTTCAGCACCTTTGAATGTGAAGTTGACCCGTAAATCTGAAGCTAATGCATCCACTACCTTTTCCACAACCACCTCAAGCACTTCTGTAGGAGTGACCA
Ccl3	NM_011337.1	MIP-1 alpha, MIP1-alpha, MIP1-(a), macrophage inflammatory protein-1alpha, MIP-1alpha, Mip1a, CCL3, G0S19-1, LD78alpha, Scya3	TCTGTCACCTGCTCAACATCATGAAGGTCTCCACCACTGCCCTTGCTGTTCTTCTCTGTACCATGACACTCTGCAACCAAGTCTTCTCAGCGCCATATGG
Ccl4	NM_013652.1	MIP-1 beta, MIP-1B, Mip1b, Act-2, AT744.1, Scya4	TTCTCAGCACCAATGGGCTCTGACCCTCCCACTTCCTGCTGTTTCTCTTACACCTCCCGGCAGCTTCACAGAAGCTTTGTGATGGATTACTATGAGACCA
Ccl5	NM_013653.1	MuRantes, Scya5, SISd, TCP228, RANTES	CCTCGTGCCCACGTCAAGGAGTATTTCTACACCAGCAGCAAGTGCTCCAATCTTGCAGTCGTGTTTGTCACTCGAAGGAACCGCCAAGTGTGTGCCAACC
Cd4	NM_013488.2	L3T4, Ly-4	AAGAGGTGTCCGTACAAAAGTCCACCAAAGACCTCAAGCTCCAGCTGAAGGAAACGCTCCCACTCACCCTCAAGATACCCCAGGTCTCGCTTCAGTTTGC
Cd40lg	NM_011616.2	CD154, gp39, Ly-62, Cd40L, IMD3, HIGM1, T-BAM, Tnfsf5	AGCAGTGGATCTGAGAGAATCTTACTCAAGGCGGCAAATACCCACAGTTCCTCCCAGCTTTGCGAGCAGCAGTCTGTTCACTTGGGCGGAGTGTTTGAAT
Csf1	NM_001113530.1	M-CSF, CSF-1, colony-stimulating factor-1, Csfm	TCCAGCTGCTGGAGAAGATCAAGAACTTCTTTAATGAAACAAAGAATCTCCTTGAAAAGGACTGGAACATTTTTACCAAGAACTGCAACAACAGCTTTGC
Csf2	NM_009969.4	Gm-CSf, Csfgm, MGI-IGM, GMCSF	AAGTCGTCTCTAACGAGTTCTCCTTCAAGAAGCTAACATGTGTGCAGACCCGCCTGAAGATATTCGAGCAGGGTCTACGGGGCAATTTCACCAAACTCAA

Cxcl1	NM_008176.1	N51, Mgsa, Fsp, Gro1, Scyb1, KC/GRO-alpha, KC	TGCTAGTAGAAGGGTGTTGTGCGAAAAGAAGTGCAGAGAGA TAGAGTTTAGTATTATGTTTTGTATGTATTAGGGTGAGGACAT GTGTGGGAGGCTGTGT
Cxcl10	NM_021274.1	CRG-2, IP-10, Ifi10, mob-1, INP10, C7, gIP-10, Scyb10, IP10	AGGACGGTCCGCTGCAACTGCATCCATATCGATGACGGGCC AGTGAGAATGAGGGCCATAGGGAAGCTTGAAATCATCCCTG CGAGCCTATCCTGCCCAC
Cxcl12	NM_021704.3	Sdf1a, Sdf1b, SDF-1, PBSF/SDF-1, Scyb12, Sdf1, TPAR1, TLSF-a, TLSF-b, PBSF	CTGAAAATCCTCAACACTCCAAACTGTGCCCTTCAGATTGTT GCACGGCTGAAGAACAACAACAGACAAGTGTGCATTGACCC GAAATTAAAGTGGATCC
Cxcl9	NM_008599.2	Mig, CMK, crg-10, Scyb9	TAGAACTCAGCTCTGCCATGAAGTCCGCTGTTCTTTTCCTCT TGGGCATCATCTTCCTGGAGCAGTGTGGAGTTCGAGGAACC CTAGTGATAAGGAATGC
Ifna1	NM_010502.2	RP23-360M23.1, Ifa1	CTGCAAGGCTGTCTGATGCAGCAGGTGGGGGTGCAGGAATT TCCCCTGACCCAGGAAGATGCCCTGCTGGCTGTGAGGAAAT ACTTCCACAGGATCACTG
Ifna2	NM_010503.2	RP23-139P14.4, Ifa2	TAACCTCAGGAACAAGAGGGCCTTGAAGGTCCTGGCACAGA TGAGGAGGCTCCCCTTTCTCTCCTGCCTGAAGGACAGGCAG GACTTTGGATTCCCCCTG
Ifnb1	NM_010510.1	interferon beta 1, fibroblast, Ifb, IFNB, IFN-beta	GATGAACTCCACCAGCAGACAGTGTTTCTGAAGACAGTACTA GAGGAAAAGCAAGAGGAAAGATTGACGTGGGAGATGTCCTC AACTGCTCTCCACTTGA
Ifng	NM_008337.1	IFN-gamma, Ifg	CTAGCTCTGAGACAATGAACGCTACACACTGCATCTTGGCTT TGCAGCTCTTCCTCATGGCTGTTTCTGGCTGTTACTGCCACG GCACAGTCATTGAAAG
Il10	NM_010548.1	cytokine synthesis inhibitory factor, IL-10	GGGCCCTTTGCTATGGTGTCCTTTCAATTGCTCTCATCCCTG AGTTCAGAGCTCCTAAGAGAGTTGTGAAGAAACTCATGGGTC TTGGGAAGAGAAACCA
Il12a	NM_008351.1	IL-12p35, p35	TCATGAAGACATCACACGGGACCAAACCAGCACATTGAAGAC CTGTTTACCACTGGAACTACACAAGAACGAGAGTTGCCTGGC TACTAGAGAGACTTCT
Il12b	NM_008352.1	Il-12p40, IL-12 p40, Il-12b, IL-23 subunit p40	TCGTAGAGAAGACATCTACCGAAGTCCAATGCAAAGGCGGG AATGTCTGCGTGCAAGCTCAGGATCGCTATTACAATTCCTCG TGCAGCAAGTGGGCATG
Il13	NM_008355.2	Il-13	AGCTACACAAAGCAACTGTTTCGCCACGGCCCCTTCTAATGA GGAGAGACCATCCCTGGGCATCTCAGCTGTGGACTCATTTT CCTTTCTCACATCAGAC

Il15	NM_008357.2		CTTGCAAACAGCACTCTGTCTTCTAACAAGAATGTAGCAGAATCTGGCTGCAAGGAATGTGAGGAGCTGGAGGAGAAAACCTTCACAGAGTTTTTGCAAA
Il17a	NM_010552.3	Ctla-8, Ctla8, Il17	ACCTCAAAGTCTTTAACTCCCTTGGCGCAAAAGTGAGCTCCAGAAGGCCCTCAGACTACCTCAACCGTTCCACGTCACCCTGGACTCTCCACCGCAATGA
Il18	NM_008360.1	Il-18, Igif	ACTTTGGCCGACTTCACTGTACAACCGCAGTAATACGGAATATAAATGACCAAGTTCTCTTCGTTGACAAAAGACAGCCTGTGTTCGAGGATATGACTGA
Il1a	NM_010554.4	Il-1a	ACCTCTGAAACGTCAAAGATGTCCAACTTCACCTTCAAGGAGAGCCGGGTGACAGTATCAGCAACGTCAAGCAACGGGAAGATTCTGAAGAAGAGACGGC
Il1b	NM_008361.3	IL-1B, IL-1beta	GTTGATTCAAGGGGACATTAGGCAGCACTCTCTAGAACAGAACCTAGCTGTCAACGTGTGGGGGATGAATTGGTCATAGCCCGCACTGAGGTCTTTCATT
Il1rn	NM_031167.5	IL-1ra	CAACCAGCTCATTGCTGGGTACTTACAAGGACCAAATATCAAACTAGAAGAAAAGATAGACATGGTGCCTATTGACCTTCATAGTGTGTTCTTGGGCATC
Il2	NM_008366.2	IL-2	GCAACTGTGGTGGACTTTCTGAGGAGATGGATAGCCTTCTGTCAAAGCATCATCTCAACAAGCCCTCAATAACTATGTACCTCCTGCTTACAACACATAA
Il21	NM_021782.2		ATGGCCTGGGGGATGGTTTTGATCTAAGGAAAAAGGTGTCTGTACCTCACAGTGCCTTTAAAACAAGCAGAGATCCCGTGTACCGCCCTAAGATAGCACA
Il22	NM_016971.1	mCG_123110, IL-22, IL-22a, ILTIFa, Iltif	AGAAGAATGTCAGAAGGCTGAAGGAGACAGTGAAAAAGCTTGGAGAGAGTGGAGAGATCAAGGCGATTGGGGAACTGGACCTGCTGTTTATGTCTCTGAG
Il23a	NM_031252.1	p19, IL-23, IL-23p19	CAAGGACAACAGCCAGTTCTGCTTGCAAAGGATCCGCCAAGGTCTGGCTTTTTATAAGCACCTGCTTGACTCTGACATCTTCAAAGGGGAGCCTGCTCTA
Il27	NM_145636.1	p28, IL-27, Il30, IL-27p28	GCTATGTCCACAGCTTTGCTGAATCTCGATTGCCAGGAGTGAACCTGGACCTCCTGCCCCTGGGATACCATCTTCCCAATGTTTCCCTGACTTTCCAGGC
Il4	NM_021283.1	Il-4	TGCTTGAAGAAGAACTCTAGTGTTCTCATGGAGCTGCAGAGACTCTTTCGGGCTTTTCGATGCCTGGATTCATCGATAAGCTGCACCATGAATGAGTCCA

Il6	NM_031168.1	Il-6	CTCTCTGCAAGAGACTTCCATCCAGTTGCCTTCTTGGGACTGATGCTGGTGACAACCACGGCCTTCCCTACTTCACAAGTCCGGAGAGGAGACTTCACAG
Il7	NM_008371.2	Il-7, hlb368	AAACATTCATTGGTGAACCACTGGGGGAGTGGAACTGTCCTGTTTTAGACTGGAGATACTGGAGGGCTCACGGTGATGGATAATGCTCTTGAAAACAAGA
Ltb	NM_008518.2	lymphotoxin beta, Tnfc, LTbeta, Tnfsf3, p33	ATCAGGGACGTCGGGTTGAGAAGATCATTGGCTCAGGAGCACAGGCTCAGAAAAGACTGGATGACAGCAAACCGTCGTGCATCTTGCCCTCACCCTCTAG
Tnf	NM_013693.1	tumor necrosis factor-alpha, TNF-alpha, TNFalpha, TNF alpha, DIF, Tnfsf1a, Tnfa	TTCCTGAGTTCTGCAAAGGGAGAGTGGTCAGGTTGCCTCTGTCTCAGAATGAGGCTGGATAAGATCTCAGGCCTTCCTACCTTCAGACCTTTCCAGACTC
Stat1	NM_009283.3		ACGCTGGGAACAGAACTAATGAGGGGCCTCTCATTGTCACCGAAGAACTTCACTCTCTTAGCTTTGAAACCCAGTTGTGCCAGCCAGGCTTGGTGATTGA
Stat3	NM_213659.2	Aprf	GGGGTCACTTTCACTTGGGTGGAAAAGGACATCAGTGGCAAGACCCAGATCCAGTCTGTAGAGCCATACACCAAGCAGCAGCTGAACAACATGTCATTTG
Ccr5	NM_009917.5	CD195, Cmkbr5	GGAGCAGGGAGAACGAGTCTTTTATCAGGGCCGGGAAATATGCACAAAGAGACTTGAGGCAGGTGCCATGACCCATATGCAAAGGGACGGACACAGGGCC
Stat4	NM_011487.4		TAGAGACCAGCTCATTACCTGTCGTGATGATTTCTAATGTCAGCCAACTACCTAATGCATGGGCATCCATCATTTGGTACAATGTATCAACTAACGACTC
Tlr2	NM_011905.2	Ly105	GCAGGCGGTCACTGGCAGGAGATGTGTCCGCAATCATAGTTTCTGATGGTGAAGGTTGGACGGCAGTCTCTGCGACCTAGAAGTGGAAAAGATGTCGTTC
Cxcr3	NM_009910.2	Cxcr3, Cmkar3, Cd183	GTTGTATGGGGTCTCTGTCTGCTCTTTGCCCTCCCAGATTTCATCTACCTATCAGCCAACTACGATCAGCGCCTCAATGCCACCCATTGCCAGTACAACT
Eomes	NM_010136.2	Tbr2	ACTGAAAAGGTCGTTCAAGGTGCTGGATTGATTCATTTATGGGAAACGAGAAATGTTCAGAAAAAGCAGGCTATGAAGAACGAGTGCCCGGTGCTATTAA
Tbx21	NM_019507.1	TBT1, Tblym, T-bet	CACTAAGCAAGGACGGCGAATGTTCCCATTCCTGTCCTTCACCGTGGCTGGGCTGGAGCCCACAAGCCATTACAGGATGTTTGTGGATGTGGTCTTGGTG

Cd3e	NM_007648.4	T3e, CD3, CD3epsilon	TGAGTACTGTGTGGAGGTGGACCTGACAGCAGTAGCCATAATCATCATTGTTGACATCTGTATCACTCTGGGCTTGCTGATGGTCATTTATTACTGGAGC
Il16	NM_010551.3		TGCGAGACAAAGCTGTTGGATGAAAAGGCCAGTAAGCTTTACTCCATCAGCAGCCAGCTATCATCTGCTGTCATGAAATCCCTGCTGTGCCTTCCATCTT
Cxcl13	NM_018866.2	BLC, BCA-1, ANGIE2, Scyb13	CTGAGAGGGAATGCTCAAGCTCCGTTGCATACCCAACCCACATCCTTGTTCCTTAAGAAAGGCTATTTGAGAACAGGCATTTAGTGACAACCCACTTCAG
Foxp3	NM_054039.1	JM2, scurfin	CCAGCTCCCGGCAACTTCTCCTGACTCTGCCTTCAGACGAGACTTGGAAGACAGTCACATCTCAGCAGCTCCTCTGCCGTTATCCAGCCTGCCTCTGACA
Gata3	NM_008091.3	Gata-3	CATGCGTGAGGAGTCTCCAAGTGTGCGAAGAGTTCCTCCGACCCCTTCTACTTGCGTTTTTCGCAGGAGCAGTATCATGAAGCCCGAAAGCGACAGATCT
Chlamydial targets			
plasmid	CT_pGP8_as.1	CT_pGP8 anti-sense	CACAGCGGTTGCTCGAAGCACGTGCGGGGTTATCTTAAAAGGGATTGCAGCTTGTAGTCCTGCTTGAGAG
omcA	CT_444.1	omcA	CCATGCGCACCTATCCAATGTTCACCTTGTGAATCTAAGAAGAAAGACGTAGACGGTGGTTGCAACTCTTGTAACGGGTATGTCCCAGCTTGCAAACCTT
23S	CT_r02.1	CT_23SrRNA	AACGGTTAGTAGGCAAATCCGCTAACATAAGATCAGGTCGCGATCAAGGGGAATCTTCGGGGGAACCGATGGTGTGGAGCGAGGCTTTCAAGAAATAATT
Internal Reference Targets			
Cltc	NM_001003908.1	3110065L21Rik, CHC, R74732	GAGTCTCAGCCAGTGAAAATGTTTGATCGTCATTCTAGCCTCGCAGGATGCCAGATCATCAATTACCGTACAGATGCAAAGCAGAAATGGTTGCTTCTCA
Gapdh	NM_001001303.1	Gapd	AGGTTGTCTCCTGCGACTTCAACAGCAACTCCCACTCTTCCACCTTCGATGCCGGGGCTGGCATTGCTCTCAATGACAACTTTGTCAAGCTCATTTCCTG

Gusb	NM_010368.1	g, Gut, Gur, asd, adipose storage deficiency, Gus-r, Gus-s, Gus-t, Gus-u, Gus	AATACGTGGTCGGAGAGCTCATCTGGAATTTCGCCGACTTCATGACGAACCAGTCACCGCTGAGAGTAATCGGAAACAAGAAGGGGATCTTCACTCGCCA
Hprt	NM_013556.2	Hprt1	TGCTGAGGCGGCGAGGGAGAGCGTTGGGCTTACCTCACTGCTTTCCGGAGCGGTAGCACCTCCTCCGCCGGCTTCCTCCTCAGACCGCTTTTTGCCGCGA
Pgk1	NM_008828.2	Pgk-1	CCGGCATTCTGCACGCTTCAAAAGCGCACGTCTGCCGCGCTGTTCTCCTCTTCCTCATCTCCGGGCCTTTCGACCTCACGGTGTTGCCAAAATGTCGCTT
Tubb5	NM_011655.4	AA408537, AI596182, B130022C14Rik, M(beta)5	ATTGGAAGTGTCTTCCCTGTATTGGTTCTCCTTTCTCGGAGAGATGGGGGTTGGGGGTGCGGCAAGGTCTTGGTCTTGGTCTCTGAACACTCCCAATTCC

Supplementary Table 2. Protein-coding genes located within ±10 Mb of the Day 7 bacterial-load peak QTL on chromosome 16 (peak~7.1Mb)

Chr	type	start	stop	gene_id	mgi_type	gene_symbol	description
16	protein_coding	5124665	7229391	ENSMUSG00000008658	MGI:1926224	Rbfox1	RNA binding protein, fox-1 homolog (C. elegans) 1 [Source:MGI Symbol;Acc:MGI:1926224]
16	protein_coding	8227139	8243000	ENSMUSG00000022715	MGI:1921970	Tmem114	transmembrane protein 114 [Source:MGI Symbol;Acc:MGI:1921970]
16	protein_coding	8288652	8308548	ENSMUSG00000039345	MGI:2384301	Mettl22	methyltransferase 22, Kin17 lysine [Source:MGI Symbol;Acc:MGI:2384301]
16	protein_coding	8331293	8439432	ENSMUSG00000057880	MGI:2443582	Abat	4-aminobutyrate aminotransferase [Source:MGI Symbol;Acc:MGI:2443582]
16	protein_coding	8451093	8455576	ENSMUSG00000043140	MGI:1913940	Tmem186	transmembrane protein 186 [Source:MGI Symbol;Acc:MGI:1913940]
16	protein_coding	8455538	8480331	ENSMUSG00000022711	MGI:1859214	Pmm2	phosphomannomutase 2 [Source:MGI Symbol;Acc:MGI:1859214]
16	protein_coding	8476444	8490019	ENSMUSG00000008393	MGI:1196368	Carhsp1	calcium regulated heat stable protein 1 [Source:MGI Symbol;Acc:MGI:1196368]
16	protein_coding	8498634	8501775	ENSMUSG00000107252	MGI:3643507	Litafd	LITAF domain containing [Source:MGI Symbol;Acc:MGI:3643507]
16	protein_coding	8507459	8610172	ENSMUSG00000022710	MGI:2182061	Usp7	ubiquitin specific peptidase 7 [Source:MGI Symbol;Acc:MGI:2182061]
16	protein_coding	8647964	8676786	ENSMUSG00000022507	MGI:1916303	Hapstr1	HUWE1 associated protein modifying stress responses [Source:MGI Symbol;Acc:MGI:1916303]
16	protein_coding	9385762	9813424	ENSMUSG00000059003	MGI:95820	Grin2a	glutamate receptor, ionotropic, NMDA2A (epsilon 1) [Source:MGI Symbol;Acc:MGI:95820]
16	protein_coding	9988090	9992775	ENSMUSG00000039209	MGI:1915422	Rpl39l	ribosomal protein L39-like [Source:MGI Symbol;Acc:MGI:1915422]
16	protein_coding	10010576	10069342	ENSMUSG00000039200	MGI:1922579	Atf7ip2	activating transcription factor 7 interacting protein 2 [Source:MGI Symbol;Acc:MGI:1922579]
16	protein_coding	10099613	10131832	ENSMUSG00000022505	MGI:1098726	Emp2	epithelial membrane protein 2 [Source:MGI Symbol;Acc:MGI:1098726]
16	protein_coding	10175812	10213354	ENSMUSG00000039179	MGI:1917676	Tekt5	tektin 5 [Source:MGI Symbol;Acc:MGI:1917676]

16	protein_coding	10229812	10242292	ENSMUSG00000022503	MGI:1347073	Nubp1	nucleotide binding protein 1 [Source:MGI Symbol;Acc:MGI:1347073]
16	protein_coding	10238421	10265226	ENSMUSG00000050908	MGI:3665441	Tvp23a	trans-golgi network vesicle protein 23A [Source:MGI Symbol;Acc:MGI:3665441]
16	protein_coding	10297923	10346282	ENSMUSG00000022504	MGI:108445	Ciita	class II transactivator [Source:MGI Symbol;Acc:MGI:108445]
16	protein_coding	10320395	10360918	ENSMUSG00000038055	MGI:1926236	Dexi	dexamethasone-induced transcript [Source:MGI Symbol;Acc:MGI:1926236]
16	protein_coding	10363203	10562742	ENSMUSG00000068663	MGI:1921624	Clec16a	C-type lectin domain family 16, member A [Source:MGI Symbol;Acc:MGI:1921624]
16	protein_coding	10600104	10603400	ENSMUSG00000038037	MGI:1354910	Socs1	suppressor of cytokine signaling 1 [Source:MGI Symbol;Acc:MGI:1354910]
16	protein_coding	10605800	10606524	ENSMUSG00000043050	MGI:98785	Tnp2	transition protein 2 [Source:MGI Symbol;Acc:MGI:98785]
16	protein_coding	10608369	10608778	ENSMUSG00000050058	MGI:106601	Prm3	protamine 3 [Source:MGI Symbol;Acc:MGI:106601]
16	protein_coding	10609244	10613998	ENSMUSG00000038015	MGI:97766	Prm2	protamine 2 [Source:MGI Symbol;Acc:MGI:97766]
16	protein_coding	10614190	10623703	ENSMUSG00000022501	MGI:97765	Prm1	protamine 1 [Source:MGI Symbol;Acc:MGI:97765]
16	protein_coding	10652923	10710830	ENSMUSG00000037991	MGI:2685383	Rmi2	RecQ mediated genome instability 2 [Source:MGI Symbol;Acc:MGI:2685383]
16	protein_coding	10777139	10884021	ENSMUSG00000022500	MGI:1929512	Litaf	LPS-induced TN factor [Source:MGI Symbol;Acc:MGI:1929512]
16	protein_coding	10878809	10892849	ENSMUSG00000037972	MGI:1276549	Snn	stannin [Source:MGI Symbol;Acc:MGI:1276549]
16	protein_coding	10892775	10952514	ENSMUSG00000022498	MGI:1923620	Txndc11	thioredoxin domain containing 11 [Source:MGI Symbol;Acc:MGI:1923620]
16	protein_coding	10954456	10994257	ENSMUSG00000037965	MGI:2445044	Zc3h7a	zinc finger CCCH type containing 7 A [Source:MGI Symbol;Acc:MGI:2445044]
16	protein_coding	11010834	11021195	ENSMUSG00000005846	MGI:1913659	Rsl1d1	ribosomal L1 domain containing 1 [Source:MGI Symbol;Acc:MGI:1913659]
16	protein_coding	11037156	11072189	ENSMUSG00000062203	MGI:1316728	Gspt1	G1 to S phase transition 1 [Source:MGI Symbol;Acc:MGI:1316728]

16	protein_coding	11131676	11137938	ENSMUSG00000022496	MGI:1343050	Tnfrsf17	tumor necrosis factor receptor superfamily, member 17 [Source:MGI Symbol;Acc:MGI:1343050]
16	protein_coding	11140772	11573336	ENSMUSG00000071669	MGI:1921728	Snx29	sorting nexin 29 [Source:MGI Symbol;Acc:MGI:1921728]
16	protein_coding	11621585	11727309	ENSMUSG00000065979	MGI:2443300	Cpped1	calcineurin-like phosphoesterase domain containing 1 [Source:MGI Symbol;Acc:MGI:2443300]
16	protein_coding	11801977	12088766	ENSMUSG00000022494	MGI:1919805	Shisa9	shisa family member 9 [Source:MGI Symbol;Acc:MGI:1919805]
16	protein_coding	12927548	12968481	ENSMUSG00000022545	MGI:1354163	Ercc4	excision repair cross-complementing rodent repair deficiency, complementation group 4 [Source:MGI Symbol;Acc:MGI:1354163]
16	protein_coding	13074345	13235393	ENSMUSG00000009569	MGI:3050795	Mrtfb	myocardin related transcription factor B [Source:MGI Symbol;Acc:MGI:3050795]
16	protein_coding	13355824	13486034	ENSMUSG00000022685	MGI:1921358	Parn	poly(A)-specific ribonuclease (deadenylation nuclease) [Source:MGI Symbol;Acc:MGI:1921358]
16	protein_coding	13489722	13521476	ENSMUSG00000022684	MGI:1914368	Bfar	bifunctional apoptosis regulator [Source:MGI Symbol;Acc:MGI:1914368]
16	protein_coding	13532921	13548847	ENSMUSG00000022683	MGI:1347522	Pla2g10	phospholipase A2, group X [Source:MGI Symbol;Acc:MGI:1347522]
16	protein_coding	13572430	13576401	ENSMUSG00000118559	MGI:3045309	A930007A09 Rik	RIKEN cDNA A930007A09 gene [Source:MGI Symbol;Acc:MGI:3045309]
16	protein_coding	13598572	13632703	ENSMUSG00000022682	MGI:1925255	Rrn3	RRN3 RNA polymerase I transcription factor homolog (yeast) [Source:MGI Symbol;Acc:MGI:1925255]
16	protein_coding	13636709	13653315	ENSMUSG00000022681	MGI:108471	Ntan1	N-terminal Asn amidase [Source:MGI Symbol;Acc:MGI:108471]
16	protein_coding	13651012	13720995	ENSMUSG00000022680	MGI:1920909	Pdxdc1	pyridoxal-dependent decarboxylase domain containing 1 [Source:MGI Symbol;Acc:MGI:1920909]
16	protein_coding	13721025	13767483	ENSMUSG00000022679	MGI:2135951	Mpv17l	Mpv17 transgene, kidney disease mutant-like [Source:MGI Symbol;Acc:MGI:2135951]

16	protein_coding	13799563	13804752	ENSMUSG00000065968	MGI:1921732	Ifitm7	interferon induced transmembrane protein 7 [Source:MGI Symbol;Acc:MGI:1921732]
16	protein_coding	13804468	13919364	ENSMUSG00000044117	MGI:1914504	Bmerb1	bMERB domain containing 1 [Source:MGI Symbol;Acc:MGI:1914504]
16	protein_coding	13927037	13981215	ENSMUSG00000060657	MGI:2444505	Marf1	meiosis regulator and mRNA stability 1 [Source:MGI Symbol;Acc:MGI:2444505]
16	protein_coding	13981139	14010792	ENSMUSG00000022678	MGI:1914453	Nde1	nudE neurodevelopment protein 1 [Source:MGI Symbol;Acc:MGI:1914453]
16	protein_coding	14012399	14109236	ENSMUSG00000018830	MGI:102643	Myh11	myosin, heavy polypeptide 11, smooth muscle [Source:MGI Symbol;Acc:MGI:102643]
16	protein_coding	14117108	14135269	ENSMUSG00000022677	MGI:1913336	Cep20	centrosomal protein 20 [Source:MGI Symbol;Acc:MGI:1913336]
16	protein_coding	14179422	14293601	ENSMUSG00000023088	MGI:102676	Abcc1	ATP-binding cassette, sub-family C member 1 [Source:MGI Symbol;Acc:MGI:102676]
16	protein_coding	14523716	14527249	ENSMUSG00000022676	MGI:1096393	Snai2	snail family zinc finger 2 [Source:MGI Symbol;Acc:MGI:1096393]
16	protein_coding	14724470	14799235	ENSMUSG00000068617	MGI:1914043	Clxn	calaxin [Source:MGI Symbol;Acc:MGI:1914043]
16	protein_coding	15369941	15413637	ENSMUSG00000022674	MGI:1917870	Ube2v2	ubiquitin-conjugating enzyme E2 variant 2 [Source:MGI Symbol;Acc:MGI:1917870]
16	protein_coding	15441761	15455264	ENSMUSG00000022673	MGI:103199	Mcm4	minichromosome maintenance complex component 4 [Source:MGI Symbol;Acc:MGI:103199]
16	protein_coding	15455730	15660099	ENSMUSG00000022672	MGI:104779	Prkdc	protein kinase, DNA activated, catalytic polypeptide [Source:MGI Symbol;Acc:MGI:104779]
16	protein_coding	15666305	15681233	ENSMUSG00000022671	MGI:1922845	Mzt2	mitotic spindle organizing protein 2 [Source:MGI Symbol;Acc:MGI:1922845]
16	protein_coding	15705150	15708895	ENSMUSG00000071637	MGI:103573	Cebpd	CCAAT/enhancer binding protein delta [Source:MGI Symbol;Acc:MGI:103573]
16	protein_coding	15707088	15964715	ENSMUSG00000041974	MGI:1924834	Spidr	scaffolding protein involved in DNA repair [Source:MGI Symbol;Acc:MGI:1924834]
16	protein_coding	16031182	16090576	ENSMUSG00000041957	MGI:1914701	Pkp2	plakophilin 2 [Source:MGI Symbol;Acc:MGI:1914701]

16	protein_coding	16120829	16127504	ENSMUSG00000022792	MGI:1917370	Yars2	tyrosyl-tRNA synthetase 2 (mitochondrial) [Source:MGI Symbol;Acc:MGI:1917370]
16	protein_coding	16130094	16176823	ENSMUSG00000022789	MGI:1921256	Dnm1l	dynamin 1-like [Source:MGI Symbol;Acc:MGI:1921256]
16	protein_coding	16234781	16418413	ENSMUSG00000022788	MGI:2183747	Fgd4	FYVE, RhoGEF and PH domain containing 4 [Source:MGI Symbol;Acc:MGI:2183747]
16	protein_coding	16490092	16494269	ENSMUSG00000048101	MGI:109316	Or7a40	olfactory receptor family 7 subfamily A member 40 [Source:MGI Symbol;Acc:MGI:109316]
16	protein_coding	16570880	16647320	ENSMUSG00000022783	MGI:1354388	Spag6l	sperm associated antigen 6-like [Source:MGI Symbol;Acc:MGI:1354388]
16	protein_coding	16678535	16681849	ENSMUSG00000075370	MGI:96529	Igll1	immunoglobulin lambda-like polypeptide 1 [Source:MGI Symbol;Acc:MGI:96529]
16	protein_coding	16686267	16688707	ENSMUSG00000059305	MGI:98936	Vpreb1a	V-set pre-B cell surrogate light chain 1A [Source:MGI Symbol;Acc:MGI:98936]
16	protein_coding	16688600	16710854	ENSMUSG00000022779	MGI:1333803	Top3b	topoisomerase (DNA) III beta [Source:MGI Symbol;Acc:MGI:1333803]
16	protein_coding	16714333	16745228	ENSMUSG00000026181	MGI:1918464	Ppm1f	protein phosphatase 1F (PP2C domain containing) [Source:MGI Symbol;Acc:MGI:1918464]
16	protein_coding	16801246	16865317	ENSMUSG00000063358	MGI:1346858	Mapk1	mitogen-activated protein kinase 1 [Source:MGI Symbol;Acc:MGI:1346858]
16	protein_coding	16887560	16904909	ENSMUSG00000022773	MGI:1913303	Ypel1	yippee like 1 [Source:MGI Symbol;Acc:MGI:1913303]
16	protein_coding	16904419	16929121	ENSMUSG00000022771	MGI:2447857	Ppil2	peptidylprolyl isomerase (cyclophilin)-like 2 [Source:MGI Symbol;Acc:MGI:2447857]
16	protein_coding	16931262	16943031	ENSMUSG00000049916	MGI:1917708	2610318N02 Rik	RIKEN cDNA 2610318N02 gene [Source:MGI Symbol;Acc:MGI:1917708]
16	protein_coding	16948002	16950247	ENSMUSG00000022769	MGI:2149842	Sdf2l1	stromal cell-derived factor 2-like 1 [Source:MGI Symbol;Acc:MGI:2149842]
16	protein_coding	16956928	16965093	ENSMUSG00000022768	MGI:1924122	Ccdc116	coiled-coil domain containing 116 [Source:MGI Symbol;Acc:MGI:1924122]
16	protein_coding	16962485	16978565	ENSMUSG00000041774	MGI:1916351	Ydjc	YdjC homolog (bacterial) [Source:MGI Symbol;Acc:MGI:1916351]
16	protein_coding	16969877	17020513	ENSMUSG00000038965	MGI:109240	Ube2l3	ubiquitin-conjugating enzyme E2L 3 [Source:MGI Symbol;Acc:MGI:109240]

16	protein_coding	17026467	17031846	ENSMUSG00000071636	MGI:2685449	Rimbp3	RIMS binding protein 3 [Source:MGI Symbol;Acc:MGI:2685449]
16	protein_coding	17051436	17081294	ENSMUSG00000050240	MGI:1929869	Hic2	hypermethylated in cancer 2 [Source:MGI Symbol;Acc:MGI:1929869]
16	protein_coding	17093941	17101093	ENSMUSG00000055692	MGI:107238	Tmem191	transmembrane protein 191 [Source:MGI Symbol;Acc:MGI:107238]
16	protein_coding	17098215	17224178	ENSMUSG00000041720	MGI:2448506	Pi4ka	phosphatidylinositol 4-kinase alpha [Source:MGI Symbol;Acc:MGI:2448506]
16	protein_coding	17149235	17161439	ENSMUSG00000022766	MGI:96051	Serpind1	serine (or cysteine) peptidase inhibitor, clade D, member 1 [Source:MGI Symbol;Acc:MGI:96051]

Supplementary Table 3. All annotated genes within the 95% Bayesian credible interval for the Day 7 bacterial-load QTL on chromosome 16

Chr	type	start	stop	gene_id	mgi_type	gene_symbol	description
16	IG_C_gene	18880368	18880821	ENSMUSG00000105906	MGI:99546	Iglc1	immunoglobulin lambda constant 1 [Source:MGI Symbol;Acc:MGI:99546]
16	IG_C_gene	18883980	18884431	ENSMUSG00000105547	MGI:99886	Iglc3	immunoglobulin lambda constant 3 [Source:MGI Symbol;Acc:MGI:99886]
16	IG_C_gene	19013749	19014062	ENSMUSG00000106039	MGI:3642936	Iglc4	immunoglobulin lambda constant 4 [Source:MGI Symbol;Acc:MGI:3642936]
16	IG_C_gene	19017146	19019101	ENSMUSG00000076937	MGI:99547	Iglc2	immunoglobulin lambda constant 2 [Source:MGI Symbol;Acc:MGI:99547]
16	IG_J_gene	18881975	18882012	ENSMUSG00000106668	MGI:5009822	Iglj1	immunoglobulin lambda joining 1 [Source:MGI Symbol;Acc:MGI:5009822]
16	IG_J_gene	18885121	18885158	ENSMUSG00000106405	MGI:3645901	Iglj3p	immunoglobulin lambda joining 3 pseudogene [Source:MGI Symbol;Acc:MGI:3645901]
16	IG_J_gene	18885791	18885828	ENSMUSG00000105231	MGI:5293407	Iglj3	immunoglobulin lambda joining 3 [Source:MGI Symbol;Acc:MGI:5293407]
16	IG_J_gene	19015245	19015286	ENSMUSG00000105066	MGI:3645510	Iglj4	immunoglobulin lambda joining 4 [Source:MGI Symbol;Acc:MGI:3645510]
16	IG_J_gene	19018948	19018985	ENSMUSG00000104975	MGI:5293409	Iglj2	immunoglobulin lambda joining 2 [Source:MGI Symbol;Acc:MGI:5293409]
16	IG_V_gene	18903767	18904250	ENSMUSG00000076934	MGI:96530	Iglv1	immunoglobulin lambda variable 1 [Source:MGI Symbol;Acc:MGI:96530]
16	IG_V_gene	19059957	19060440	ENSMUSG00000076939	MGI:3645509	Iglv3	immunoglobulin lambda variable 3 [Source:MGI Symbol;Acc:MGI:3645509]
16	IG_V_gene	19079151	19079609	ENSMUSG00000076940	MGI:99548	Iglv2	immunoglobulin lambda variable 2 [Source:MGI Symbol;Acc:MGI:99548]
16	lncRNA	6028260	6049376	ENSMUSG00000143715	MGI:7805634	Gm61295	predicted gene, 61295 [Source:MGI Symbol;Acc:MGI:7805634]

16	lncRNA	6163728	6166837	ENSMUSG00000143829	MGI:7805644	Gm61302	predicted gene, 61302 [Source:MGI Symbol;Acc:MGI:7805644]
16	lncRNA	6298226	6330952	ENSMUSG00000143626	MGI:7805606	Gm61281	predicted gene, 61281 [Source:MGI Symbol;Acc:MGI:7805606]
16	lncRNA	6582243	6597040	ENSMUSG00000143791	MGI:7805640	Gm61298	predicted gene, 61298 [Source:MGI Symbol;Acc:MGI:7805640]
16	lncRNA	6836996	6842096	ENSMUSG00000138950	MGI:7804973	Gm60964	predicted gene, 60964 [Source:MGI Symbol;Acc:MGI:7804973]
16	lncRNA	6904486	6917736	ENSMUSG00000123065	MGI:7802569	Gm59754	predicted gene, 59754 [Source:MGI Symbol;Acc:MGI:7802569]
16	lncRNA	6977986	6979694	ENSMUSG00000123172	MGI:7802583	Gm59761	predicted gene, 59761 [Source:MGI Symbol;Acc:MGI:7802583]
16	lncRNA	7028135	7036670	ENSMUSG00000123107	MGI:7802573	Gm59756	predicted gene, 59756 [Source:MGI Symbol;Acc:MGI:7802573]
16	lncRNA	7104704	7107338	ENSMUSG00000123137	MGI:7802577	Gm59758	predicted gene, 59758 [Source:MGI Symbol;Acc:MGI:7802577]
16	lncRNA	7237187	7241286	ENSMUSG00000128473	MGI:7803205	Gm60076	predicted gene, 60076 [Source:MGI Symbol;Acc:MGI:7803205]
16	lncRNA	7302513	7303200	ENSMUSG00000139398	MGI:7805035	Gm60995	predicted gene, 60995 [Source:MGI Symbol;Acc:MGI:7805035]
16	lncRNA	7584296	7584981	ENSMUSG00000125667	MGI:7802783	Gm59861	predicted gene, 59861 [Source:MGI Symbol;Acc:MGI:7802783]
16	lncRNA	7585726	7588456	ENSMUSG00000124707	MGI:5589013	Gm29854	predicted gene, 29854 [Source:MGI Symbol;Acc:MGI:5589013]
16	lncRNA	7630206	7631750	ENSMUSG00000123267	MGI:7802603	Gm59771	predicted gene, 59771 [Source:MGI Symbol;Acc:MGI:7802603]
16	lncRNA	7687966	7697723	ENSMUSG00000116450	MGI:6155235	Gm49534	predicted gene, 49534 [Source:MGI Symbol;Acc:MGI:6155235]
16	lncRNA	7765480	7769537	ENSMUSG00000139532	MGI:7805061	Gm61008	predicted gene, 61008 [Source:MGI Symbol;Acc:MGI:7805061]
16	lncRNA	7886440	7891071	ENSMUSG00000137024	MGI:7804593	Gm60774	predicted gene, 60774 [Source:MGI Symbol;Acc:MGI:7804593]
16	lncRNA	8160162	8177767	ENSMUSG00000126210	MGI:5589095	Gm29936	predicted gene, 29936 [Source:MGI Symbol;Acc:MGI:5589095]
16	lncRNA	8275922	8289483	ENSMUSG00000128980	MGI:5589171	Gm30012	predicted gene, 30012 [Source:MGI Symbol;Acc:MGI:5589171]

16	lncRNA	8315029	8317226	ENSMUSG00000143008	MGI:7805511	Gm61233	predicted gene, 61233 [Source:MGI Symbol;Acc:MGI:7805511]
16	lncRNA	8495965	8500615	ENSMUSG00000125402	MGI:7802771	Gm59855	predicted gene, 59855 [Source:MGI Symbol;Acc:MGI:7802771]
16	lncRNA	8538592	8545526	ENSMUSG00000125362	MGI:7802763	Gm59851	predicted gene, 59851 [Source:MGI Symbol;Acc:MGI:7802763]
16	lncRNA	8555352	8563430	ENSMUSG00000125319	MGI:7802759	Gm59849	predicted gene, 59849 [Source:MGI Symbol;Acc:MGI:7802759]
16	lncRNA	8588468	8588618	ENSMUSG00000125487	MGI:7802775	Gm59857	predicted gene, 59857 [Source:MGI Symbol;Acc:MGI:7802775]
16	lncRNA	8599272	8603553	ENSMUSG00000125445	MGI:7802773	Gm59856	predicted gene, 59856 [Source:MGI Symbol;Acc:MGI:7802773]
16	lncRNA	8640009	8641685	ENSMUSG00000122939	MGI:7802553	Gm59746	predicted gene, 59746 [Source:MGI Symbol;Acc:MGI:7802553]
16	lncRNA	8700834	8702196	ENSMUSG00000125833	MGI:7802797	Gm59868	predicted gene, 59868 [Source:MGI Symbol;Acc:MGI:7802797]
16	lncRNA	8762756	8787764	ENSMUSG00000116525	MGI:5589530	Gm30371	predicted gene, 30371 [Source:MGI Symbol;Acc:MGI:5589530]
16	lncRNA	8815371	8825701	ENSMUSG00000136575	MGI:5589603	Gm30444	predicted gene, 30444 [Source:MGI Symbol;Acc:MGI:5589603]
16	lncRNA	8820898	8822122	ENSMUSG00000136615	MGI:7804559	Gm60757	predicted gene, 60757 [Source:MGI Symbol;Acc:MGI:7804559]
16	lncRNA	8827559	8834631	ENSMUSG00000138549	MGI:7804869	Gm60912	predicted gene, 60912 [Source:MGI Symbol;Acc:MGI:7804869]
16	lncRNA	8867836	8873284	ENSMUSG00000116426	MGI:5589726	Gm30567	predicted gene, 30567 [Source:MGI Symbol;Acc:MGI:5589726]
16	lncRNA	8897260	9038550	ENSMUSG00000116469	MGI:6155097	Gm49448	predicted gene, 49448 [Source:MGI Symbol;Acc:MGI:6155097]
16	lncRNA	8981276	8990641	ENSMUSG00000138255	MGI:7804833	Gm60894	predicted gene, 60894 [Source:MGI Symbol;Acc:MGI:7804833]
16	lncRNA	9097790	9108447	ENSMUSG00000133742	MGI:5624303	Gm41418	predicted gene, 41418 [Source:MGI Symbol;Acc:MGI:5624303]
16	lncRNA	9114599	9116518	ENSMUSG00000137900	MGI:7804817	Gm60886	predicted gene, 60886 [Source:MGI Symbol;Acc:MGI:7804817]
16	lncRNA	9116612	9119168	ENSMUSG00000129757	MGI:7803371	Gm60159	predicted gene, 60159 [Source:MGI Symbol;Acc:MGI:7803371]

16	lncRNA	9212100	9213004	ENSMUSG00000142707	MGI:7805503	Gm61229	predicted gene, 61229 [Source:MGI Symbol;Acc:MGI:7805503]
16	lncRNA	9815073	9820381	ENSMUSG00000122586	MGI:5589827	Gm30668	predicted gene, 30668 [Source:MGI Symbol;Acc:MGI:5589827]
16	lncRNA	10004157	10045388	ENSMUSG00000115943	MGI:6155110	Gm49455	predicted gene, 49455 [Source:MGI Symbol;Acc:MGI:6155110]
16	lncRNA	10141631	10169214	ENSMUSG00000116270	MGI:2686446	Gm1600	predicted gene 1600 [Source:MGI Symbol;Acc:MGI:2686446]
16	lncRNA	10167472	10171739	ENSMUSG00000129485	MGI:5589883	Gm30724	predicted gene, 30724 [Source:MGI Symbol;Acc:MGI:5589883]
16	lncRNA	10200623	10201046	ENSMUSG00000143676	MGI:7805632	Gm61294	predicted gene, 61294 [Source:MGI Symbol;Acc:MGI:7805632]
16	lncRNA	10265251	10265526	ENSMUSG00000138914	MGI:7804971	Gm60963	predicted gene, 60963 [Source:MGI Symbol;Acc:MGI:7804971]
16	lncRNA	10267228	10269692	ENSMUSG00000142680	MGI:7805499	Gm61227	predicted gene, 61227 [Source:MGI Symbol;Acc:MGI:7805499]
16	lncRNA	10466165	10476113	ENSMUSG00000129964	MGI:6366384	Gm52264	predicted gene, 52264 [Source:MGI Symbol;Acc:MGI:6366384]
16	lncRNA	10487112	10501456	ENSMUSG00000087384	MGI:3783007	Gm15558	predicted gene 15558 [Source:MGI Symbol;Acc:MGI:3783007]
16	lncRNA	10510191	10515959	ENSMUSG00000130911	MGI:7803645	Gm60296	predicted gene, 60296 [Source:MGI Symbol;Acc:MGI:7803645]
16	lncRNA	10566395	10582213	ENSMUSG00000140467	MGI:7805191	Gm61073	predicted gene, 61073 [Source:MGI Symbol;Acc:MGI:7805191]
16	lncRNA	10589796	10590068	ENSMUSG00000140592	MGI:7805209	Gm61082	predicted gene, 61082 [Source:MGI Symbol;Acc:MGI:7805209]
16	lncRNA	10590215	10590598	ENSMUSG00000140555	MGI:7805201	Gm61078	predicted gene, 61078 [Source:MGI Symbol;Acc:MGI:7805201]
16	lncRNA	10602518	10630590	ENSMUSG00000116038	MGI:5826200	Gm46563	predicted gene, 46563 [Source:MGI Symbol;Acc:MGI:5826200]
16	lncRNA	10603508	10604089	ENSMUSG00000140525	MGI:7805199	Gm61077	predicted gene, 61077 [Source:MGI Symbol;Acc:MGI:7805199]
16	lncRNA	10645765	10691449	ENSMUSG00000096921	MGI:5477316	Gm26822	predicted gene, 26822 [Source:MGI Symbol;Acc:MGI:5477316]
16	lncRNA	10729865	10777126	ENSMUSG00000095369	MGI:5434023	Gm21859	predicted gene, 21859 [Source:MGI Symbol;Acc:MGI:5434023]

16	lncRNA	10816946	10821356	ENSMUSG00000125386	MGI:7802769	Gm59854	predicted gene, 59854 [Source:MGI Symbol;Acc:MGI:7802769]
16	lncRNA	10849820	10854284	ENSMUSG00000115992	MGI:3642210	Gm10832	predicted gene 10832 [Source:MGI Symbol;Acc:MGI:3642210]
16	lncRNA	10864620	10868803	ENSMUSG00000138912	MGI:7805754	Gm61360	predicted gene, 61360 [Source:MGI Symbol;Acc:MGI:7805754]
16	lncRNA	10924374	10925211	ENSMUSG00000138960	MGI:7804977	Gm60966	predicted gene, 60966 [Source:MGI Symbol;Acc:MGI:7804977]
16	lncRNA	10952801	10968472	ENSMUSG00000141422	MGI:7805295	Gm61125	predicted gene, 61125 [Source:MGI Symbol;Acc:MGI:7805295]
16	lncRNA	10994094	11010156	ENSMUSG00000096959	MGI:1914984	4930509G22Rik	RIKEN cDNA 4930509G22 gene [Source:MGI Symbol;Acc:MGI:1914984]
16	lncRNA	11020710	11082342	ENSMUSG00000097537	MGI:1917168	2610020C07Rik	RIKEN cDNA 2610020C07 gene [Source:MGI Symbol;Acc:MGI:1917168]
16	lncRNA	11021207	11033249	ENSMUSG00000133226	MGI:7804043	Gm60496	predicted gene, 60496 [Source:MGI Symbol;Acc:MGI:7804043]
16	lncRNA	11071866	11074500	ENSMUSG00000120347	MGI:7337108	Gm56597	predicted gene, 56597 [Source:MGI Symbol;Acc:MGI:7337108]
16	lncRNA	11449930	11451039	ENSMUSG00000127042	MGI:7802969	Gm59955	predicted gene, 59955 [Source:MGI Symbol;Acc:MGI:7802969]
16	lncRNA	11506033	11509721	ENSMUSG00000085732	MGI:3782456	Gm4279	predicted gene 4279 [Source:MGI Symbol;Acc:MGI:3782456]
16	lncRNA	11596255	11599365	ENSMUSG00000115936	MGI:1921011	4833415N18Rik	RIKEN cDNA 4833415N18 gene [Source:MGI Symbol;Acc:MGI:1921011]
16	lncRNA	11719214	11748464	ENSMUSG00000055015	MGI:3642444	Gm9961	predicted gene 9961 [Source:MGI Symbol;Acc:MGI:3642444]
16	lncRNA	11796526	11803089	ENSMUSG00000139264	MGI:5591437	Gm32278	predicted gene, 32278 [Source:MGI Symbol;Acc:MGI:5591437]
16	lncRNA	12120848	12126717	ENSMUSG00000143211	MGI:7805533	Gm61244	predicted gene, 61244 [Source:MGI Symbol;Acc:MGI:7805533]
16	lncRNA	12128412	12135411	ENSMUSG00000143401	MGI:7805582	Gm61269	predicted gene, 61269 [Source:MGI Symbol;Acc:MGI:7805582]

16	lncRNA	12380552	12393444	ENSMUSG00000123409	MGI:7802611	Gm59775	predicted gene, 59775 [Source:MGI Symbol;Acc:MGI:7802611]
16	lncRNA	12381586	12400760	ENSMUSG00000123373			novel transcript
16	lncRNA	12446948	12763828	ENSMUSG00000116520	MGI:1926040	4930414F18Rik	RIKEN cDNA 4930414F18 gene [Source:MGI Symbol;Acc:MGI:1926040]
16	lncRNA	12467885	12490637	ENSMUSG00000140920	MGI:7805227	Gm61091	predicted gene, 61091 [Source:MGI Symbol;Acc:MGI:7805227]
16	lncRNA	12522232	12530015	ENSMUSG00000116197	MGI:5621504	Gm38619	predicted gene, 38619 [Source:MGI Symbol;Acc:MGI:5621504]
16	lncRNA	12618172	12679236	ENSMUSG00000101565	MGI:1922743	1700003L19Rik	RIKEN cDNA 1700003L19 gene [Source:MGI Symbol;Acc:MGI:1922743]
16	lncRNA	12761874	12783117	ENSMUSG00000140400	MGI:7805165	Gm61060	predicted gene, 61060 [Source:MGI Symbol;Acc:MGI:7805165]
16	lncRNA	12835898	12858295	ENSMUSG00000139816	MGI:7805105	Gm61030	predicted gene, 61030 [Source:MGI Symbol;Acc:MGI:7805105]
16	lncRNA	12893934	12903277	ENSMUSG00000138678	MGI:7804887	Gm60921	predicted gene, 60921 [Source:MGI Symbol;Acc:MGI:7804887]
16	lncRNA	12924490	12927725	ENSMUSG00000133609	MGI:7804069	Gm60509	predicted gene, 60509 [Source:MGI Symbol;Acc:MGI:7804069]
16	lncRNA	12945386	12946851	ENSMUSG00000133642	MGI:7804071	Gm60510	predicted gene, 60510 [Source:MGI Symbol;Acc:MGI:7804071]
16	lncRNA	13007358	13007728	ENSMUSG00000120723	MGI:7337150	Gm56618	predicted gene, 56618 [Source:MGI Symbol;Acc:MGI:7337150]
16	lncRNA	13172238	13286862	ENSMUSG00000087526	MGI:3783180	Gm15738	predicted gene 15738 [Source:MGI Symbol;Acc:MGI:3783180]
16	lncRNA	13255098	13257252	ENSMUSG00000138767	MGI:5624306	Gm41421	predicted gene, 41421 [Source:MGI Symbol;Acc:MGI:5624306]
16	lncRNA	13265280	13292725	ENSMUSG00000109857	MGI:6435685	Gm53058	predicted gene 53058 [Source:MGI Symbol;Acc:MGI:6435685]
16	lncRNA	13331995	13338971	ENSMUSG00000099411	MGI:1917350	2310015D24Rik	RIKEN cDNA 2310015D24 gene [Source:MGI Symbol;Acc:MGI:1917350]
16	lncRNA	13357121	13359618	ENSMUSG00000129391	MGI:7803309	Gm60128	predicted gene, 60128 [Source:MGI Symbol;Acc:MGI:7803309]

16	lncRNA	13489793	13496252	ENSMUSG00000121810	MGI:1913848	Pphln1-ps1	periphilin 1, pseudogene 1 [Source:MGI Symbol;Acc:MGI:1913848]
16	lncRNA	13547660	13557951	ENSMUSG00000086550	MGI:1923934	Pla2g10os	phospholipase A2, group X, opposite strand [Source:MGI Symbol;Acc:MGI:1923934]
16	lncRNA	13555685	13557150	ENSMUSG00000115907	MGI:6155072	Gm49432	predicted gene, 49432 [Source:MGI Symbol;Acc:MGI:6155072]
16	lncRNA	13597524	13598492	ENSMUSG00000133370	MGI:7804061	Gm60505	predicted gene, 60505 [Source:MGI Symbol;Acc:MGI:7804061]
16	lncRNA	13735512	13747906	ENSMUSG00000135269	MGI:7804413	Gm60684	predicted gene, 60684 [Source:MGI Symbol;Acc:MGI:7804413]
16	lncRNA	13977162	13978745	ENSMUSG00000132903	MGI:7804023	Gm60486	predicted gene, 60486 [Source:MGI Symbol;Acc:MGI:7804023]
16	lncRNA	14129705	14131878	ENSMUSG00000085612	MGI:3801810	Gm15868	predicted gene 15868 [Source:MGI Symbol;Acc:MGI:3801810]
16	lncRNA	14135026	14136102	ENSMUSG00000134352	MGI:7804215	Gm60584	predicted gene, 60584 [Source:MGI Symbol;Acc:MGI:7804215]
16	lncRNA	14282780	14285186	ENSMUSG00000127938	MGI:7803143	Gm60045	predicted gene, 60045 [Source:MGI Symbol;Acc:MGI:7803143]
16	lncRNA	14305705	14320267	ENSMUSG00000137348	MGI:7804727	Gm60841	predicted gene, 60841 [Source:MGI Symbol;Acc:MGI:7804727]
16	lncRNA	14380181	14451972	ENSMUSG00000075395	MGI:3584518	A630010A05Rik	RIKEN cDNA A630010A05 gene [Source:MGI Symbol;Acc:MGI:3584518]
16	lncRNA	14522512	14523294	ENSMUSG00000140603	MGI:7805213	Gm61084	predicted gene, 61084 [Source:MGI Symbol;Acc:MGI:7805213]
16	lncRNA	14554607	14575347	ENSMUSG00000138555	MGI:7804871	Gm60913	predicted gene, 60913 [Source:MGI Symbol;Acc:MGI:7804871]
16	lncRNA	14655394	14686705	ENSMUSG00000129731	MGI:7803367	Gm60157	predicted gene, 60157 [Source:MGI Symbol;Acc:MGI:7803367]
16	lncRNA	14710178	14810477	ENSMUSG00000116324	MGI:5592855	Gm33696	predicted gene, 33696 [Source:MGI Symbol;Acc:MGI:5592855]
16	lncRNA	14753434	14754793	ENSMUSG00000134967	MGI:7804365	Gm60660	predicted gene, 60660 [Source:MGI Symbol;Acc:MGI:7804365]
16	lncRNA	14871190	14873987	ENSMUSG00000122977	MGI:7802561	Gm59750	predicted gene, 59750 [Source:MGI Symbol;Acc:MGI:7802561]

16	lncRNA	14919773	14931959	ENSMUSG00000129831	MGI:5624308	Gm41423	predicted gene, 41423 [Source:MGI Symbol;Acc:MGI:5624308]
16	lncRNA	15420808	15422665	ENSMUSG00000132084	MGI:7803895	Gm60422	predicted gene, 60422 [Source:MGI Symbol;Acc:MGI:7803895]
16	lncRNA	15422303	15422514	ENSMUSG00000132119	MGI:7803901	Gm60425	predicted gene, 60425 [Source:MGI Symbol;Acc:MGI:7803901]
16	lncRNA	15681222	15695451	ENSMUSG00000143526	MGI:5624310	Gm41425	predicted gene, 41425 [Source:MGI Symbol;Acc:MGI:5624310]
16	lncRNA	15702802	15705084	ENSMUSG00000120071	MGI:7337110	Gm56598	predicted gene, 56598 [Source:MGI Symbol;Acc:MGI:7337110]
16	lncRNA	15967184	16037806	ENSMUSG00000133200	MGI:7804041	Gm60495	predicted gene, 60495 [Source:MGI Symbol;Acc:MGI:7804041]
16	lncRNA	16167067	16176875	ENSMUSG00000115923	MGI:6155216	Gm49521	predicted gene, 49521 [Source:MGI Symbol;Acc:MGI:6155216]
16	lncRNA	16182848	16187925	ENSMUSG00000126709	MGI:7805696	Gm61329	predicted gene, 61329 [Source:MGI Symbol;Acc:MGI:7805696]
16	lncRNA	16502799	16536017	ENSMUSG00000116096	MGI:1921316	4933404G15Rik	RIKEN cDNA 4933404G15 gene [Source:MGI Symbol;Acc:MGI:1921316]
16	lncRNA	16536064	16549298	ENSMUSG00000133884	MGI:5826207	Gm46570	predicted gene, 46570 [Source:MGI Symbol;Acc:MGI:5826207]
16	lncRNA	16547849	16550171	ENSMUSG00000134003	MGI:5593499	Gm34340	predicted gene, 34340 [Source:MGI Symbol;Acc:MGI:5593499]
16	lncRNA	16710097	16715212	ENSMUSG00000133917	MGI:7804155	Gm60552	predicted gene, 60552 [Source:MGI Symbol;Acc:MGI:7804155]
16	lncRNA	16727142	16730903	ENSMUSG00000116823	MGI:6215001	Gm49595	predicted gene, 49595 [Source:MGI Symbol;Acc:MGI:6215001]
16	lncRNA	16865352	16866982	ENSMUSG00000084792	MGI:1920642	1700056N10Rik	RIKEN cDNA 1700056N10 gene [Source:MGI Symbol;Acc:MGI:1920642]
16	lncRNA	16886863	16887904	ENSMUSG00000124450	MGI:7802675	Gm59807	predicted gene, 59807 [Source:MGI Symbol;Acc:MGI:7802675]
16	lncRNA	16910679	16914214	ENSMUSG00000116645	MGI:6214984	Gm49584	predicted gene, 49584 [Source:MGI Symbol;Acc:MGI:6214984]
16	lncRNA	16950326	16962363	ENSMUSG00000136619	MGI:7804561	Gm60758	predicted gene, 60758 [Source:MGI Symbol;Acc:MGI:7804561]

16	lncRNA	17051465	17094582	ENSMUSG00000116743	MGI:6214966	Gm49573	predicted gene, 49573 [Source:MGI Symbol;Acc:MGI:6214966]
16	lncRNA	17269243	17269754	ENSMUSG00000134583	MGI:7804283	Gm60618	predicted gene, 60618 [Source:MGI Symbol;Acc:MGI:7804283]
16	lncRNA	17305055	17309053	ENSMUSG00000125877	MGI:7802811	Gm59875	predicted gene, 59875 [Source:MGI Symbol;Acc:MGI:7802811]
16	lncRNA	17348623	17349393	ENSMUSG00000116898	MGI:6215307	Gm49785	predicted gene, 49785 [Source:MGI Symbol;Acc:MGI:6215307]
16	lncRNA	17372506	17372706	ENSMUSG00000132968	MGI:7804035	Gm60492	predicted gene, 60492 [Source:MGI Symbol;Acc:MGI:7804035]
16	lncRNA	17414960	17437229	ENSMUSG00000122877	MGI:7802545	Gm59742	predicted gene, 59742 [Source:MGI Symbol;Acc:MGI:7802545]
16	lncRNA	17459885	17464310	ENSMUSG00000122922	MGI:7802551	Gm59745	predicted gene, 59745 [Source:MGI Symbol;Acc:MGI:7802551]
16	lncRNA	17637250	17640232	ENSMUSG00000125857	MGI:7802809	Gm59874	predicted gene, 59874 [Source:MGI Symbol;Acc:MGI:7802809]
16	lncRNA	17739113	17740370	ENSMUSG00000120128	MGI:7337122	Gm56604	predicted gene, 56604 [Source:MGI Symbol;Acc:MGI:7337122]
16	lncRNA	17739423	17741632	ENSMUSG00000123401	MGI:7802609	Gm59774	predicted gene, 59774 [Source:MGI Symbol;Acc:MGI:7802609]
16	lncRNA	17829693	17834855	ENSMUSG00000125584	MGI:7802779	Gm59859	predicted gene, 59859 [Source:MGI Symbol;Acc:MGI:7802779]
16	lncRNA	17836792	17846448	ENSMUSG00000131237	MGI:7803737	Gm60343	predicted gene, 60343 [Source:MGI Symbol;Acc:MGI:7803737]
16	lncRNA	17846509	17849732	ENSMUSG00000116658	MGI:6214978	Gm49580	predicted gene, 49580 [Source:MGI Symbol;Acc:MGI:6214978]
16	lncRNA	17907246	17911119	ENSMUSG00000139216	MGI:7805003	Gm60979	predicted gene, 60979 [Source:MGI Symbol;Acc:MGI:7805003]
16	lncRNA	17932751	17934859	ENSMUSG00000131794	MGI:7803837	Gm60393	predicted gene, 60393 [Source:MGI Symbol;Acc:MGI:7803837]
16	lncRNA	17974398	17978508	ENSMUSG00000127684	MGI:6722909	Gm53937	predicted gene, 53937 [Source:MGI Symbol;Acc:MGI:6722909]
16	lncRNA	17987405	17992187	ENSMUSG00000142704	MGI:5593838	Gm34679	predicted gene, 34679 [Source:MGI Symbol;Acc:MGI:5593838]

16	lncRNA	18016530	18035679	ENSMUSG00000116557	MGI:1914029	4933432I09Rik	RIKEN cDNA 4933432I09 gene [Source:MGI Symbol;Acc:MGI:1914029]
16	lncRNA	18031117	18033910	ENSMUSG00000116772	MGI:1925419	4930483P17Rik	RIKEN cDNA 4930483P17 gene [Source:MGI Symbol;Acc:MGI:1925419]
16	lncRNA	18053109	18058284	ENSMUSG00000131206	MGI:7803735	Gm60342	predicted gene, 60342 [Source:MGI Symbol;Acc:MGI:7803735]
16	lncRNA	18107303	18118210	ENSMUSG00000141558	MGI:7805321	Gm61138	predicted gene, 61138 [Source:MGI Symbol;Acc:MGI:7805321]
16	lncRNA	18197049	18198469	ENSMUSG00000126711	MGI:7802891	Gm59915	predicted gene, 59915 [Source:MGI Symbol;Acc:MGI:7802891]
16	lncRNA	18218314	18219589	ENSMUSG00000126471	MGI:7802863	Gm59901	predicted gene, 59901 [Source:MGI Symbol;Acc:MGI:7802863]
16	lncRNA	18235419	18241192	ENSMUSG00000087556	MGI:3783206	Gm15764	predicted gene 15764 [Source:MGI Symbol;Acc:MGI:3783206]
16	lncRNA	18317651	18320408	ENSMUSG00000121876	MGI:3833934	Rtl10	retrotransposon Gag like 10 [Source:MGI Symbol;Acc:MGI:3833934]
16	lncRNA	18405901	18410211	ENSMUSG00000132162			novel transcript, antisense to Tbx1
16	lncRNA	18409743	18420004	ENSMUSG00000132088	MGI:7803897	Gm60423	predicted gene, 60423 [Source:MGI Symbol;Acc:MGI:7803897]
16	lncRNA	18410669	18428506	ENSMUSG00000075335	MGI:3588254	4930588K23Rik	RIKEN cDNA 4930588K23 gene [Source:MGI Symbol;Acc:MGI:3588254]
16	lncRNA	18427517	18436688	ENSMUSG00000132123	MGI:7803903	Gm60426	predicted gene, 60426 [Source:MGI Symbol;Acc:MGI:7803903]
16	lncRNA	18435825	18436786	ENSMUSG00000132197	MGI:7803913	Gm60431	predicted gene, 60431 [Source:MGI Symbol;Acc:MGI:7803913]
16	lncRNA	18500866	18573973	ENSMUSG00000143558			novel transcript
16	lncRNA	18513380	18530525	ENSMUSG00000143641	MGI:7805610	Gm61283	predicted gene, 61283 [Source:MGI Symbol;Acc:MGI:7805610]
16	lncRNA	18532404	18558588	ENSMUSG00000143599	MGI:7805600	Gm61278	predicted gene, 61278 [Source:MGI Symbol;Acc:MGI:7805600]
16	lncRNA	18594613	18595436	ENSMUSG00000129554	MGI:7803339	Gm60143	predicted gene, 60143 [Source:MGI Symbol;Acc:MGI:7803339]

16	lncRNA	18598650	18605054	ENSMUSG00000131881	MGI:7803867	Gm60408	predicted gene, 60408 [Source:MGI Symbol;Acc:MGI:7803867]
16	lncRNA	18614470	18616329	ENSMUSG00000131917	MGI:6366339	Gm52240	predicted gene, 52240 [Source:MGI Symbol;Acc:MGI:6366339]
16	lncRNA	18695432	18695601	ENSMUSG00000130102	MGI:7803395	Gm60171	predicted gene, 60171 [Source:MGI Symbol;Acc:MGI:7803395]
16	lncRNA	18830004	18869920	ENSMUSG00000129794	MGI:7803377	Gm60162	predicted gene, 60162 [Source:MGI Symbol;Acc:MGI:7803377]
16	lncRNA	18870082	18925657	ENSMUSG00000106567	MGI:1917268	2010309G21Rik	RIKEN cDNA 2010309G21 gene [Source:MGI Symbol;Acc:MGI:1917268]
16	lncRNA	19031405	19055691	ENSMUSG00000104927	MGI:5663525	Gm43388	predicted gene 43388 [Source:MGI Symbol;Acc:MGI:5663525]
16	lncRNA	19059925	19061438	ENSMUSG00000120432	MGI:7337114	Gm56600	predicted gene, 56600 [Source:MGI Symbol;Acc:MGI:7337114]
16	lncRNA	19249434	19264227	ENSMUSG00000141973	MGI:7805369	Gm61162	predicted gene, 61162 [Source:MGI Symbol;Acc:MGI:7805369]
16	lncRNA	19255062	19256544	ENSMUSG00000142056	MGI:7805415	Gm61185	predicted gene, 61185 [Source:MGI Symbol;Acc:MGI:7805415]
16	lncRNA	19667792	19669093	ENSMUSG00000139649	MGI:7805067	Gm61011	predicted gene, 61011 [Source:MGI Symbol;Acc:MGI:7805067]
16	lncRNA	19702254	19703034	ENSMUSG00000116712	MGI:6215005	Gm49598	predicted gene, 49598 [Source:MGI Symbol;Acc:MGI:6215005]
16	lncRNA	19736660	19737002	ENSMUSG00000143559	MGI:7805598	Gm61277	predicted gene, 61277 [Source:MGI Symbol;Acc:MGI:7805598]
16	lncRNA	19881953	19916124	ENSMUSG00000121994	MGI:7802475	Gm59707	predicted gene, 59707 [Source:MGI Symbol;Acc:MGI:7802475]
16	lncRNA	19994858	19995848	ENSMUSG00000128869	MGI:7805756	Gm61361	predicted gene, 61361 [Source:MGI Symbol;Acc:MGI:7805756]
16	lncRNA	19998599	20001075	ENSMUSG00000128829	MGI:7803257	Gm60102	predicted gene, 60102 [Source:MGI Symbol;Acc:MGI:7803257]
16	lncRNA	20018424	20021080	ENSMUSG00000134705	MGI:7804303	Gm60629	predicted gene, 60629 [Source:MGI Symbol;Acc:MGI:7804303]
16	lncRNA	20059407	20060864	ENSMUSG00000116913	MGI:6214956	Gm49566	predicted gene, 49566 [Source:MGI Symbol;Acc:MGI:6214956]

16	lncRNA	20194384	20198690	ENSMUSG00000134802	MGI:7804329	Gm60642	predicted gene, 60642 [Source:MGI Symbol;Acc:MGI:7804329]
16	lncRNA	20263987	20270959	ENSMUSG00000134765	MGI:7804323	Gm60639	predicted gene, 60639 [Source:MGI Symbol;Acc:MGI:7804323]
16	lncRNA	20270241	20273128	ENSMUSG00000120468	MGI:7337120	Gm56603	predicted gene, 56603 [Source:MGI Symbol;Acc:MGI:7337120]
16	lncRNA	20271000	20278095	ENSMUSG00000134726	MGI:5624317	Gm41432	predicted gene, 41432 [Source:MGI Symbol;Acc:MGI:5624317]
16	lncRNA	20292658	20303397	ENSMUSG00000134688	MGI:7804299	Gm60627	predicted gene, 60627 [Source:MGI Symbol;Acc:MGI:7804299]
16	lncRNA	20308232	20310021	ENSMUSG00000129936	MGI:7805758	Gm61362	predicted gene, 61362 [Source:MGI Symbol;Acc:MGI:7805758]
16	lncRNA	20329883	20336516	ENSMUSG00000086837	MGI:4439542	Gm16618	predicted gene, 16618 [Source:MGI Symbol;Acc:MGI:4439542]
16	lncRNA	20337986	20339633	ENSMUSG00000141814	MGI:7805357	Gm61156	predicted gene, 61156 [Source:MGI Symbol;Acc:MGI:7805357]
16	lncRNA	20363953	20367283	ENSMUSG00000121849			mitochondrial ribosomal protein S18B pseudogene [Source:NCBI gene (formerly Entrezgene);Acc:100042948]
16	lncRNA	20385108	20387258	ENSMUSG00000116604	MGI:6215237	Gm49745	predicted gene, 49745 [Source:MGI Symbol;Acc:MGI:6215237]
16	lncRNA	20397869	20408808	ENSMUSG00000134179	MGI:5594844	Gm35685	predicted gene, 35685 [Source:MGI Symbol;Acc:MGI:5594844]
16	lncRNA	20408867	20409434	ENSMUSG00000116764	MGI:6215242	Gm49748	predicted gene, 49748 [Source:MGI Symbol;Acc:MGI:6215242]
16	lncRNA	20465948	20470320	ENSMUSG00000134212	MGI:7804197	Gm60574	predicted gene, 60574 [Source:MGI Symbol;Acc:MGI:7804197]
16	lncRNA	20619042	20622135	ENSMUSG00000116922	MGI:1921159	4833419O12Rik	RIKEN cDNA 4833419O12 gene [Source:MGI Symbol;Acc:MGI:1921159]
16	lncRNA	20638540	20640264	ENSMUSG00000143957	MGI:7805714	Gm61338	predicted gene, 61338 [Source:MGI Symbol;Acc:MGI:7805714]
16	lncRNA	20647788	20732730	ENSMUSG00000129217	MGI:7803285	Gm60116	predicted gene, 60116 [Source:MGI Symbol;Acc:MGI:7803285]
16	lncRNA	20694007	20712309	ENSMUSG00000116850	MGI:6215232	Gm49742	predicted gene, 49742 [Source:MGI Symbol;Acc:MGI:6215232]

16	lncRNA	20747265	20749766	ENSMUSG00000134417	MGI:7804239	Gm60596	predicted gene, 60596 [Source:MGI Symbol;Acc:MGI:7804239]
16	lncRNA	20747436	20749160	ENSMUSG00000134456			predicted gene 6551 [Source:NCBI gene (formerly Entrezgene);Acc:625060]
16	lncRNA	20880680	20882067	ENSMUSG00000130835			predicted gene 6557 [Source:NCBI gene (formerly Entrezgene);Acc:625123]
16	lncRNA	20929759	20935770	ENSMUSG00000141159	MGI:7805241	Gm61098	predicted gene, 61098 [Source:MGI Symbol;Acc:MGI:7805241]
16	lncRNA	20931581	20938100	ENSMUSG00000141120	MGI:7805235	Gm61095	predicted gene, 61095 [Source:MGI Symbol;Acc:MGI:7805235]
16	lncRNA	21057245	21081909	ENSMUSG00000116843	MGI:6215246	Gm49750	predicted gene, 49750 [Source:MGI Symbol;Acc:MGI:6215246]
16	lncRNA	21066244	21115622	ENSMUSG00000096918	MGI:4439787	Gm16863	predicted gene, 16863 [Source:MGI Symbol;Acc:MGI:4439787]
16	lncRNA	21083898	21090711	ENSMUSG00000130455	MGI:7803497	Gm60222	predicted gene, 60222 [Source:MGI Symbol;Acc:MGI:7803497]
16	lncRNA	21129303	21149480	ENSMUSG00000134551	MGI:7804275	Gm60614	predicted gene, 60614 [Source:MGI Symbol;Acc:MGI:7804275]
16	lncRNA	21140249	21152139	ENSMUSG00000121787			MAGE family member F1 [Source:NCBI gene (formerly Entrezgene);Acc:76222]
16	lncRNA	21152105	21158921	ENSMUSG00000120420	MGI:7337138	Gm56612	predicted gene, 56612 [Source:MGI Symbol;Acc:MGI:7337138]
16	lncRNA	21172798	21188491	ENSMUSG00000122490	MGI:7802507	Gm59723	predicted gene, 59723 [Source:MGI Symbol;Acc:MGI:7802507]
16	lncRNA	21177282	21214274	ENSMUSG00000122447			novel transcript
16	lncRNA	21210108	21217490	ENSMUSG00000122534	MGI:5595309	Gm36150	predicted gene, 36150 [Source:MGI Symbol;Acc:MGI:5595309]
16	lncRNA	21240234	21241878	ENSMUSG00000138453	MGI:6722913	Gm53939	predicted gene, 53939 [Source:MGI Symbol;Acc:MGI:6722913]
16	lncRNA	21329875	21346379	ENSMUSG00000138790	MGI:7804961	Gm60958	predicted gene, 60958 [Source:MGI Symbol;Acc:MGI:7804961]
16	miRNA	8441027	8441134	ENSMUSG00002075670	MGI:6846536	Gm55031	predicted gene, 55031 [Source:MGI Symbol;Acc:MGI:6846536]

16	miRNA	10091614	10091685	ENSMUSG00002076434	MGI:6846586	Gm55056	predicted gene, 55056 [Source:MGI Symbol;Acc:MGI:6846586]
16	miRNA	10783588	10783713	ENSMUSG00002075232	MGI:6846702	Gm55114	predicted gene, 55114 [Source:MGI Symbol;Acc:MGI:6846702]
16	miRNA	10961989	10962045	ENSMUSG00002076173	MGI:6846709	Gm55118	predicted gene, 55118 [Source:MGI Symbol;Acc:MGI:6846709]
16	miRNA	11072232	11072309	ENSMUSG00000088544	MGI:3836984	Mir1945	microRNA 1945 [Source:MGI Symbol;Acc:MGI:3836984]
16	miRNA	13267387	13267465	ENSMUSG00000076972	MGI:3718458	Mir193b	microRNA 193b [Source:MGI Symbol;Acc:MGI:3718458]
16	miRNA	13271704	13271790	ENSMUSG00000065449	MGI:3619371	Mir365-1	microRNA 365-1 [Source:MGI Symbol;Acc:MGI:3619371]
16	miRNA	13288944	13289044	ENSMUSG00000099214	MGI:5531065	Mir6365	microRNA 6365 [Source:MGI Symbol;Acc:MGI:5531065]
16	miRNA	13637096	13637184	ENSMUSG00002075895	MGI:6846797	Gm55162	predicted gene, 55162 [Source:MGI Symbol;Acc:MGI:6846797]
16	miRNA	13946430	13946495	ENSMUSG00002076089	MGI:6845248	Gm54384	predicted gene, 54384 [Source:MGI Symbol;Acc:MGI:6845248]
16	miRNA	13977490	13977556	ENSMUSG00000070074	MGI:3619421	Mir484	microRNA 484 [Source:MGI Symbol;Acc:MGI:3619421]
16	miRNA	16941925	16942006	ENSMUSG00000065572	MGI:3618716	Mir130b	microRNA 130b [Source:MGI Symbol;Acc:MGI:3618716]
16	miRNA	16942264	16942360	ENSMUSG00000076288	MGI:3691602	Mir301b	microRNA 301b [Source:MGI Symbol;Acc:MGI:3691602]
16	miRNA	17982953	17983034	ENSMUSG00000099293	MGI:5531102	Mir6366	microRNA 6366 [Source:MGI Symbol;Acc:MGI:5531102]
16	miRNA	18102103	18102181	ENSMUSG00000080452	MGI:3836985	Mir1306	microRNA 1306 [Source:MGI Symbol;Acc:MGI:3836985]
16	miRNA	18102414	18102501	ENSMUSG00000093246	MGI:5454349	Mir3618	microRNA 3618 [Source:MGI Symbol;Acc:MGI:5454349]
16	miRNA	18145265	18145329	ENSMUSG00000065464	MGI:2676849	Mir185	microRNA 185 [Source:MGI Symbol;Acc:MGI:2676849]
16	miRNA	18179300	18179370	ENSMUSG00002075123	MGI:6847211	Gm55370	predicted gene, 55370 [Source:MGI Symbol;Acc:MGI:6847211]
16	miRNA	19138436	19138545	ENSMUSG00002075049	MGI:6845949	Gm54736	predicted gene, 54736 [Source:MGI Symbol;Acc:MGI:6845949]

16	miRNA	19702549	19702650	ENSMUSG00002075497	MGI:6845973	Gm54748	predicted gene, 54748 [Source:MGI Symbol;Acc:MGI:6845973]
16	miRNA	20423202	20423286	ENSMUSG00000080669	MGI:3764925	Mir1224	microRNA 1224 [Source:MGI Symbol;Acc:MGI:3764925]
16	miRNA	21074763	21074816	ENSMUSG00000098400	MGI:5530799	Mir7680	microRNA 7680 [Source:MGI Symbol;Acc:MGI:5530799]
16	misc_RNA	8021839	8022112	ENSMUSG00002075614	MGI:6846520	Gm55023	predicted gene, 55023 [Source:MGI Symbol;Acc:MGI:6846520]
16	misc_RNA	8267362	8267650	ENSMUSG00002076758	MGI:6846522	Gm55024	predicted gene, 55024 [Source:MGI Symbol;Acc:MGI:6846522]
16	misc_RNA	20264403	20264677	ENSMUSG00002076542	MGI:6846033	Gm54778	predicted gene, 54778 [Source:MGI Symbol;Acc:MGI:6846033]
16	processed_pseudogene	7526600	7526930	ENSMUSG00000115989	MGI:6155234	Gm49533	predicted gene, 49533 [Source:MGI Symbol;Acc:MGI:6155234]
16	processed_pseudogene	7921238	7921652	ENSMUSG00000116151	MGI:6155237	Gm49535	predicted gene, 49535 [Source:MGI Symbol;Acc:MGI:6155237]
16	processed_pseudogene	8441050	8441584	ENSMUSG00000083505	MGI:3648278	Gm7541	predicted gene 7541 [Source:MGI Symbol;Acc:MGI:3648278]
16	processed_pseudogene	8443073	8443181	ENSMUSG00000116095	MGI:6155257	Gm49549	predicted gene, 49549 [Source:MGI Symbol;Acc:MGI:6155257]
16	processed_pseudogene	8739924	8740253	ENSMUSG00000068674	MGI:3641838	Gm10247	predicted gene 10247 [Source:MGI Symbol;Acc:MGI:3641838]
16	processed_pseudogene	8813952	8814260	ENSMUSG00000116352	MGI:6155096	Gm49447	predicted gene, 49447 [Source:MGI Symbol;Acc:MGI:6155096]
16	processed_pseudogene	8922667	8925634	ENSMUSG00000116348	MGI:5010907	Gm18722	predicted gene, 18722 [Source:MGI Symbol;Acc:MGI:5010907]
16	processed_pseudogene	9862829	9863594	ENSMUSG00000098100	MGI:3645867	Gm7572	predicted gene 7572 [Source:MGI Symbol;Acc:MGI:3645867]

16	processed_pseudogene	9863623	9863829	ENSMUSG00000098170	MGI:3782063	Gm3890	predicted gene 3890 [Source:MGI Symbol;Acc:MGI:3782063]
16	processed_pseudogene	10056030	10056380	ENSMUSG00000116187	MGI:6155112	Gm49456	predicted gene, 49456 [Source:MGI Symbol;Acc:MGI:6155112]
16	processed_pseudogene	10619245	10619577	ENSMUSG00000071671	MGI:3642690	Gm10343	predicted gene 10343 [Source:MGI Symbol;Acc:MGI:3642690]
16	processed_pseudogene	10854538	10855195	ENSMUSG00000115911	MGI:3782093	Gm3919	predicted gene 3919 [Source:MGI Symbol;Acc:MGI:3782093]
16	processed_pseudogene	11002997	11003963	ENSMUSG00000080790	MGI:3643470	Gm7638	predicted gene 7638 [Source:MGI Symbol;Acc:MGI:3643470]
16	processed_pseudogene	11008118	11009129	ENSMUSG00000081052	MGI:3644967	Gm6305	predicted gene 6305 [Source:MGI Symbol;Acc:MGI:3644967]
16	processed_pseudogene	11312685	11314170	ENSMUSG00000082653	MGI:3801942	Gm15897	predicted gene 15897 [Source:MGI Symbol;Acc:MGI:3801942]
16	processed_pseudogene	12198994	12199366	ENSMUSG00000116403	MGI:5011756	Gm19571	predicted gene, 19571 [Source:MGI Symbol;Acc:MGI:5011756]
16	processed_pseudogene	12226147	12227041	ENSMUSG00000116013	MGI:2146727	C87114	expressed sequence C87114 [Source:MGI Symbol;Acc:MGI:2146727]
16	processed_pseudogene	12577896	12579230	ENSMUSG00000091416	MGI:3646646	Gm6327	predicted gene 6327 [Source:MGI Symbol;Acc:MGI:3646646]
16	processed_pseudogene	12768645	12769327	ENSMUSG00000116170	MGI:3648790	Gm6332	predicted gene 6332 [Source:MGI Symbol;Acc:MGI:3648790]
16	processed_pseudogene	13828626	13829580	ENSMUSG00000082554	MGI:3802043	Gm15806	predicted gene 15806 [Source:MGI Symbol;Acc:MGI:3802043]

16	processed_pseudogene	13855598	13856725	ENSMUSG00000082277	MGI:3802044	Gm15807	predicted gene 15807 [Source:MGI Symbol;Acc:MGI:3802044]
16	processed_pseudogene	13885267	13885870	ENSMUSG00000083536	MGI:3802045	Gm15808	predicted gene 15808 [Source:MGI Symbol;Acc:MGI:3802045]
16	processed_pseudogene	14176296	14176761	ENSMUSG00000116025	MGI:6155210	Gm49517	predicted gene, 49517 [Source:MGI Symbol;Acc:MGI:6155210]
16	processed_pseudogene	14608281	14608875	ENSMUSG00000116400	MGI:6155231	Gm49530	predicted gene, 49530 [Source:MGI Symbol;Acc:MGI:6155231]
16	processed_pseudogene	14818186	14818353	ENSMUSG00000116317	MGI:6155238	Gm49536	predicted gene, 49536 [Source:MGI Symbol;Acc:MGI:6155238]
16	processed_pseudogene	15034723	15035192	ENSMUSG00000069315	MGI:3644153	Gm7731	predicted gene 7731 [Source:MGI Symbol;Acc:MGI:3644153]
16	processed_pseudogene	15036708	15039372	ENSMUSG00000116213	MGI:3647383	Gm5404	predicted gene 5404 [Source:MGI Symbol;Acc:MGI:3647383]
16	processed_pseudogene	15244015	15245068	ENSMUSG00000080704	MGI:3645805	Gm6031	predicted gene 6031 [Source:MGI Symbol;Acc:MGI:3645805]
16	processed_pseudogene	15269868	15270626	ENSMUSG00000116423	MGI:3642962	Gm7085	predicted gene 7085 [Source:MGI Symbol;Acc:MGI:3642962]
16	processed_pseudogene	15405229	15405923	ENSMUSG00000116386	MGI:6155240	Gm49538	predicted gene, 49538 [Source:MGI Symbol;Acc:MGI:6155240]
16	processed_pseudogene	15426971	15427379	ENSMUSG00000116140	MGI:3646851	Gm7748	predicted gene 7748 [Source:MGI Symbol;Acc:MGI:3646851]
16	processed_pseudogene	15568787	15569056	ENSMUSG00000116118	MGI:6155202	Gm49511	predicted gene, 49511 [Source:MGI Symbol;Acc:MGI:6155202]

16	processed_pseudogene	15689693	15690023	ENSMUSG00000116102	MGI:5012267	Gm20082	predicted gene, 20082 [Source:MGI Symbol;Acc:MGI:5012267]
16	processed_pseudogene	15917462	15917975	ENSMUSG00000116385	MGI:5010519	Gm18334	predicted gene, 18334 [Source:MGI Symbol;Acc:MGI:5010519]
16	processed_pseudogene	16093467	16093811	ENSMUSG00000115882	MGI:3648103	Gm5481	predicted gene 5481 [Source:MGI Symbol;Acc:MGI:3648103]
16	processed_pseudogene	16197076	16198072	ENSMUSG00000116192	MGI:5011054	Gm18869	predicted gene, 18869 [Source:MGI Symbol;Acc:MGI:5011054]
16	processed_pseudogene	16200523	16200736	ENSMUSG00000118629	MGI:6388916	Gm53024	predicted gene, 53024 [Source:MGI Symbol;Acc:MGI:6388916]
16	processed_pseudogene	16360187	16360606	ENSMUSG00000089861	MGI:3801880	Gm16139	predicted gene 16139 [Source:MGI Symbol;Acc:MGI:3801880]
16	processed_pseudogene	16666715	16667562	ENSMUSG00000116729	MGI:6215076	Gm49644	predicted gene, 49644 [Source:MGI Symbol;Acc:MGI:6215076]
16	processed_pseudogene	16670179	16671721	ENSMUSG00000116676	MGI:3647546	Gm5768	predicted gene 5768 [Source:MGI Symbol;Acc:MGI:3647546]
16	processed_pseudogene	16750725	16750959	ENSMUSG00000116703	MGI:5826177	Gm46540	predicted gene, 46540 [Source:MGI Symbol;Acc:MGI:5826177]
16	processed_pseudogene	16786473	16786932	ENSMUSG00000116598	MGI:3647845	Gm6438	predicted gene 6438 [Source:MGI Symbol;Acc:MGI:3647845]
16	processed_pseudogene	16899863	16900555	ENSMUSG00000055497	MGI:3708610	Gm9974	predicted gene 9974 [Source:MGI Symbol;Acc:MGI:3708610]
16	processed_pseudogene	16955192	16955545	ENSMUSG00000116694	MGI:3644482	Gm6440	predicted gene 6440 [Source:MGI Symbol;Acc:MGI:3644482]

16	processed_pseudogene	17520791	17521455	ENSMUSG00000116561	MGI:5010520	Gm18335	predicted gene, 18335 [Source:MGI Symbol;Acc:MGI:5010520]
16	processed_pseudogene	17522207	17522964	ENSMUSG00000116994	MGI:6215133	Gm49684	predicted gene, 49684 [Source:MGI Symbol;Acc:MGI:6215133]
16	processed_pseudogene	17529532	17529969	ENSMUSG00000059150	MGI:3644695	Rpl26-ps4	ribosomal protein L26, pseudogene 4 [Source:MGI Symbol;Acc:MGI:3644695]
16	processed_pseudogene	17532725	17533003	ENSMUSG00000116696	MGI:6215132	Gm49683	predicted gene, 49683 [Source:MGI Symbol;Acc:MGI:6215132]
16	processed_pseudogene	17543240	17543549	ENSMUSG00000116783	MGI:3645583	Gm6048	predicted gene 6048 [Source:MGI Symbol;Acc:MGI:3645583]
16	processed_pseudogene	17552033	17552550	ENSMUSG00000116936	MGI:3646992	Bod1-ps	biorientation of chromosomes in cell division 1, pseudogene [Source:MGI Symbol;Acc:MGI:3646992]
16	processed_pseudogene	17632892	17634185	ENSMUSG00000116777	MGI:1343185	Zfp520-ps	zinc finger protein 520, pseudogene [Source:MGI Symbol;Acc:MGI:1343185]
16	processed_pseudogene	18500087	18500966	ENSMUSG00000116825	MGI:3643238	Rps2-ps7	ribosomal protein S2, pseudogene 7 [Source:MGI Symbol;Acc:MGI:3643238]
16	processed_pseudogene	18756151	18756439	ENSMUSG00000082130	MGI:3802098	Gm15799	predicted gene 15799 [Source:MGI Symbol;Acc:MGI:3802098]
16	processed_pseudogene	18763008	18763415	ENSMUSG00000083658	MGI:3802099	Gm15798	predicted gene 15798 [Source:MGI Symbol;Acc:MGI:3802099]
16	processed_pseudogene	18836805	18837452	ENSMUSG00000105028	MGI:5010932	Gm18747	predicted gene, 18747 [Source:MGI Symbol;Acc:MGI:5010932]
16	processed_pseudogene	18846982	18847404	ENSMUSG00000060811	MGI:3641695	Gm10088	predicted gene 10088 [Source:MGI Symbol;Acc:MGI:3641695]

16	processed_pseudogene	18980177	18986796	ENSMUSG00000106444	MGI:5010933	Gm18748	predicted gene, 18748 [Source:MGI Symbol;Acc:MGI:5010933]
16	processed_pseudogene	19450652	19451092	ENSMUSG00000116790	MGI:6215003	Gm49596	predicted gene, 49596 [Source:MGI Symbol;Acc:MGI:6215003]
16	processed_pseudogene	19542062	19542728	ENSMUSG00000097945	MGI:5009985	Gm17799	predicted gene, 17799 [Source:MGI Symbol;Acc:MGI:5009985]
16	processed_pseudogene	19671205	19671517	ENSMUSG00000116980	MGI:6215004	Gm49597	predicted gene, 49597 [Source:MGI Symbol;Acc:MGI:6215004]
16	processed_pseudogene	20384327	20384778	ENSMUSG00000116942	MGI:6215236	Gm49744	predicted gene, 49744 [Source:MGI Symbol;Acc:MGI:6215236]
16	processed_pseudogene	20390160	20390432	ENSMUSG00000116538	MGI:6215239	Gm49746	predicted gene, 49746 [Source:MGI Symbol;Acc:MGI:6215239]
16	processed_pseudogene	20401859	20402174	ENSMUSG00000116808	MGI:5012349	Gm20164	predicted gene, 20164 [Source:MGI Symbol;Acc:MGI:5012349]
16	processed_pseudogene	20482504	20483806	ENSMUSG00000090319	MGI:3782646	Gm4462	predicted gene 4462 [Source:MGI Symbol;Acc:MGI:3782646]
16	processed_pseudogene	20615977	20616523	ENSMUSG00000116776	MGI:5826181	Gm46544	predicted gene, 46544 [Source:MGI Symbol;Acc:MGI:5826181]
16	processed_pseudogene	20640895	20641390	ENSMUSG00000099764	MGI:3645627	Rps10-ps2	ribosomal protein S10, pseudogene 2 [Source:MGI Symbol;Acc:MGI:3645627]
16	processed_pseudogene	20779377	20779892	ENSMUSG00000116732	MGI:5010954	Gm18769	predicted gene, 18769 [Source:MGI Symbol;Acc:MGI:5010954]
16	processed_pseudogene	20840078	20840592	ENSMUSG00000116592	MGI:5010955	Gm18770	predicted gene, 18770 [Source:MGI Symbol;Acc:MGI:5010955]

16	processed_pseudogene	20898420	20899416	ENSMUSG00000086936	MGI:3780104	Gm9697	predicted gene 9697 [Source:MGI Symbol;Acc:MGI:3780104]
16	processed_pseudogene	20940580	20941273	ENSMUSG00000116731	MGI:5010956	Gm18771	predicted gene, 18771 [Source:MGI Symbol;Acc:MGI:5010956]
16	protein_coding	5124665	7229391	ENSMUSG00000008658	MGI:1926224	Rbfox1	RNA binding protein, fox-1 homolog (C. elegans) 1 [Source:MGI Symbol;Acc:MGI:1926224]
16	protein_coding	8227139	8243000	ENSMUSG00000022715	MGI:1921970	Tmem114	transmembrane protein 114 [Source:MGI Symbol;Acc:MGI:1921970]
16	protein_coding	8288652	8308548	ENSMUSG00000039345	MGI:2384301	Mettl22	methyltransferase 22, Kin17 lysine [Source:MGI Symbol;Acc:MGI:2384301]
16	protein_coding	8331293	8439432	ENSMUSG00000057880	MGI:2443582	Abat	4-aminobutyrate aminotransferase [Source:MGI Symbol;Acc:MGI:2443582]
16	protein_coding	8451093	8455576	ENSMUSG00000043140	MGI:1913940	Tmem186	transmembrane protein 186 [Source:MGI Symbol;Acc:MGI:1913940]
16	protein_coding	8455538	8480331	ENSMUSG00000022711	MGI:1859214	Pmm2	phosphomannomutase 2 [Source:MGI Symbol;Acc:MGI:1859214]
16	protein_coding	8476444	8490019	ENSMUSG00000008393	MGI:1196368	Carhsp1	calcium regulated heat stable protein 1 [Source:MGI Symbol;Acc:MGI:1196368]
16	protein_coding	8498634	8501775	ENSMUSG00000107252	MGI:3643507	Litafd	LITAF domain containing [Source:MGI Symbol;Acc:MGI:3643507]
16	protein_coding	8507459	8610172	ENSMUSG00000022710	MGI:2182061	Usp7	ubiquitin specific peptidase 7 [Source:MGI Symbol;Acc:MGI:2182061]
16	protein_coding	8647964	8676786	ENSMUSG00000022507	MGI:1916303	Hapstr1	HUWE1 associated protein modifying stress responses [Source:MGI Symbol;Acc:MGI:1916303]

16	protein_coding	9385762	9813424	ENSMUSG00000059003	MGI:95820	Grin2a	glutamate receptor, ionotropic, NMDA2A (epsilon 1) [Source:MGI Symbol;Acc:MGI:95820]
16	protein_coding	9988090	9992775	ENSMUSG00000039209	MGI:1915422	Rpl39l	ribosomal protein L39-like [Source:MGI Symbol;Acc:MGI:1915422]
16	protein_coding	10010576	10069342	ENSMUSG00000039200	MGI:1922579	Atf7ip2	activating transcription factor 7 interacting protein 2 [Source:MGI Symbol;Acc:MGI:1922579]
16	protein_coding	10099613	10131832	ENSMUSG00000022505	MGI:1098726	Emp2	epithelial membrane protein 2 [Source:MGI Symbol;Acc:MGI:1098726]
16	protein_coding	10175812	10213354	ENSMUSG00000039179	MGI:1917676	Tekt5	tektin 5 [Source:MGI Symbol;Acc:MGI:1917676]
16	protein_coding	10229812	10242292	ENSMUSG00000022503	MGI:1347073	Nubp1	nucleotide binding protein 1 [Source:MGI Symbol;Acc:MGI:1347073]
16	protein_coding	10238421	10265226	ENSMUSG00000050908	MGI:3665441	Tvp23a	trans-golgi network vesicle protein 23A [Source:MGI Symbol;Acc:MGI:3665441]
16	protein_coding	10297923	10346282	ENSMUSG00000022504	MGI:108445	Ciita	class II transactivator [Source:MGI Symbol;Acc:MGI:108445]
16	protein_coding	10320395	10360918	ENSMUSG00000038055	MGI:1926236	Dexi	dexamethasone-induced transcript [Source:MGI Symbol;Acc:MGI:1926236]
16	protein_coding	10363203	10562742	ENSMUSG00000068663	MGI:1921624	Clec16a	C-type lectin domain family 16, member A [Source:MGI Symbol;Acc:MGI:1921624]
16	protein_coding	10600104	10603400	ENSMUSG00000038037	MGI:1354910	Socs1	suppressor of cytokine signaling 1 [Source:MGI Symbol;Acc:MGI:1354910]
16	protein_coding	10605800	10606524	ENSMUSG00000043050	MGI:98785	Tnp2	transition protein 2 [Source:MGI Symbol;Acc:MGI:98785]
16	protein_coding	10608369	10608778	ENSMUSG00000050058	MGI:106601	Prm3	protamine 3 [Source:MGI Symbol;Acc:MGI:106601]
16	protein_coding	10609244	10613998	ENSMUSG00000038015	MGI:97766	Prm2	protamine 2 [Source:MGI Symbol;Acc:MGI:97766]

16	protein_coding	10614190	10623703	ENSMUSG00000022501	MGI:97765	Prm1	protamine 1 [Source:MGI Symbol;Acc:MGI:97765]
16	protein_coding	10652923	10710830	ENSMUSG00000037991	MGI:2685383	Rmi2	RecQ mediated genome instability 2 [Source:MGI Symbol;Acc:MGI:2685383]
16	protein_coding	10777139	10884021	ENSMUSG00000022500	MGI:1929512	Litaf	LPS-induced TN factor [Source:MGI Symbol;Acc:MGI:1929512]
16	protein_coding	10878809	10892849	ENSMUSG00000037972	MGI:1276549	Snn	stannin [Source:MGI Symbol;Acc:MGI:1276549]
16	protein_coding	10892775	10952514	ENSMUSG00000022498	MGI:1923620	Txndc11	thioredoxin domain containing 11 [Source:MGI Symbol;Acc:MGI:1923620]
16	protein_coding	10954456	10994257	ENSMUSG00000037965	MGI:2445044	Zc3h7a	zinc finger CCCH type containing 7 A [Source:MGI Symbol;Acc:MGI:2445044]
16	protein_coding	11010834	11021195	ENSMUSG00000005846	MGI:1913659	Rsl1d1	ribosomal L1 domain containing 1 [Source:MGI Symbol;Acc:MGI:1913659]
16	protein_coding	11037156	11072189	ENSMUSG00000062203	MGI:1316728	Gspt1	G1 to S phase transition 1 [Source:MGI Symbol;Acc:MGI:1316728]
16	protein_coding	11131676	11137938	ENSMUSG00000022496	MGI:1343050	Tnfrsf17	tumor necrosis factor receptor superfamily, member 17 [Source:MGI Symbol;Acc:MGI:1343050]
16	protein_coding	11140772	11573336	ENSMUSG00000071669	MGI:1921728	Snx29	sorting nexin 29 [Source:MGI Symbol;Acc:MGI:1921728]
16	protein_coding	11621585	11727309	ENSMUSG00000065979	MGI:2443300	Cpped1	calcineurin-like phosphoesterase domain containing 1 [Source:MGI Symbol;Acc:MGI:2443300]
16	protein_coding	11801977	12088766	ENSMUSG00000022494	MGI:1919805	Shisa9	shisa family member 9 [Source:MGI Symbol;Acc:MGI:1919805]
16	protein_coding	12927548	12968481	ENSMUSG00000022545	MGI:1354163	Ercc4	excision repair cross-complementing rodent repair deficiency, complementation group 4 [Source:MGI Symbol;Acc:MGI:1354163]

16	protein_coding	13074345	13235393	ENSMUSG00000009569	MGI:3050795	Mrtfb	myocardin related transcription factor B [Source:MGI Symbol;Acc:MGI:3050795]
16	protein_coding	13355824	13486034	ENSMUSG00000022685	MGI:1921358	Parn	poly(A)-specific ribonuclease (deadenylation nuclease) [Source:MGI Symbol;Acc:MGI:1921358]
16	protein_coding	13489722	13521476	ENSMUSG00000022684	MGI:1914368	Bfar	bifunctional apoptosis regulator [Source:MGI Symbol;Acc:MGI:1914368]
16	protein_coding	13532921	13548847	ENSMUSG00000022683	MGI:1347522	Pla2g10	phospholipase A2, group X [Source:MGI Symbol;Acc:MGI:1347522]
16	protein_coding	13572430	13576401	ENSMUSG00000118559	MGI:3045309	A930007A09Rik	RIKEN cDNA A930007A09 gene [Source:MGI Symbol;Acc:MGI:3045309]
16	protein_coding	13598572	13632703	ENSMUSG00000022682	MGI:1925255	Rrn3	RRN3 RNA polymerase I transcription factor homolog (yeast) [Source:MGI Symbol;Acc:MGI:1925255]
16	protein_coding	13636709	13653315	ENSMUSG00000022681	MGI:108471	Ntan1	N-terminal Asn amidase [Source:MGI Symbol;Acc:MGI:108471]
16	protein_coding	13651012	13720995	ENSMUSG00000022680	MGI:1920909	Pdxdc1	pyridoxal-dependent decarboxylase domain containing 1 [Source:MGI Symbol;Acc:MGI:1920909]
16	protein_coding	13721025	13767483	ENSMUSG00000022679	MGI:2135951	Mpv17l	Mpv17 transgene, kidney disease mutant-like [Source:MGI Symbol;Acc:MGI:2135951]
16	protein_coding	13799563	13804752	ENSMUSG00000065968	MGI:1921732	Ifitm7	interferon induced transmembrane protein 7 [Source:MGI Symbol;Acc:MGI:1921732]
16	protein_coding	13804468	13919364	ENSMUSG00000044117	MGI:1914504	Bmerb1	bMERB domain containing 1 [Source:MGI Symbol;Acc:MGI:1914504]
16	protein_coding	13927037	13981215	ENSMUSG00000060657	MGI:2444505	Marf1	meiosis regulator and mRNA stability 1 [Source:MGI Symbol;Acc:MGI:2444505]

16	protein_coding	13981139	14010792	ENSMUSG00000022678	MGI:1914453	Nde1	nudE neurodevelopment protein 1 [Source:MGI Symbol;Acc:MGI:1914453]
16	protein_coding	14012399	14109236	ENSMUSG00000018830	MGI:102643	Myh11	myosin, heavy polypeptide 11, smooth muscle [Source:MGI Symbol;Acc:MGI:102643]
16	protein_coding	14117108	14135269	ENSMUSG00000022677	MGI:1913336	Cep20	centrosomal protein 20 [Source:MGI Symbol;Acc:MGI:1913336]
16	protein_coding	14179422	14293601	ENSMUSG00000023088	MGI:102676	Abcc1	ATP-binding cassette, sub-family C member 1 [Source:MGI Symbol;Acc:MGI:102676]
16	protein_coding	14523716	14527249	ENSMUSG00000022676	MGI:1096393	Snai2	snail family zinc finger 2 [Source:MGI Symbol;Acc:MGI:1096393]
16	protein_coding	14724470	14799235	ENSMUSG00000068617	MGI:1914043	Clxn	calaxin [Source:MGI Symbol;Acc:MGI:1914043]
16	protein_coding	15369941	15413637	ENSMUSG00000022674	MGI:1917870	Ube2v2	ubiquitin-conjugating enzyme E2 variant 2 [Source:MGI Symbol;Acc:MGI:1917870]
16	protein_coding	15441761	15455264	ENSMUSG00000022673	MGI:103199	Mcm4	minichromosome maintenance complex component 4 [Source:MGI Symbol;Acc:MGI:103199]
16	protein_coding	15455730	15660099	ENSMUSG00000022672	MGI:104779	Prkdc	protein kinase, DNA activated, catalytic polypeptide [Source:MGI Symbol;Acc:MGI:104779]
16	protein_coding	15666305	15681233	ENSMUSG00000022671	MGI:1922845	Mzt2	mitotic spindle organizing protein 2 [Source:MGI Symbol;Acc:MGI:1922845]
16	protein_coding	15705150	15708895	ENSMUSG00000071637	MGI:103573	Cebpd	CCAAT/enhancer binding protein delta [Source:MGI Symbol;Acc:MGI:103573]
16	protein_coding	15707088	15964715	ENSMUSG00000041974	MGI:1924834	Spidr	scaffolding protein involved in DNA repair [Source:MGI Symbol;Acc:MGI:1924834]
16	protein_coding	16031182	16090576	ENSMUSG00000041957	MGI:1914701	Pkp2	plakophilin 2 [Source:MGI Symbol;Acc:MGI:1914701]

16	protein_coding	16120829	16127504	ENSMUSG00000022792	MGI:1917370	Yars2	tyrosyl-tRNA synthetase 2 (mitochondrial) [Source:MGI Symbol;Acc:MGI:1917370]
16	protein_coding	16130094	16176823	ENSMUSG00000022789	MGI:1921256	Dnm1l	dynamin 1-like [Source:MGI Symbol;Acc:MGI:1921256]
16	protein_coding	16234781	16418413	ENSMUSG00000022788	MGI:2183747	Fgd4	FYVE, RhoGEF and PH domain containing 4 [Source:MGI Symbol;Acc:MGI:2183747]
16	protein_coding	16490092	16494269	ENSMUSG00000048101	MGI:109316	Or7a40	olfactory receptor family 7 subfamily A member 40 [Source:MGI Symbol;Acc:MGI:109316]
16	protein_coding	16570880	16647320	ENSMUSG00000022783	MGI:1354388	Spag6l	sperm associated antigen 6-like [Source:MGI Symbol;Acc:MGI:1354388]
16	protein_coding	16678535	16681849	ENSMUSG00000075370	MGI:96529	Igll1	immunoglobulin lambda-like polypeptide 1 [Source:MGI Symbol;Acc:MGI:96529]
16	protein_coding	16686267	16688707	ENSMUSG00000059305	MGI:98936	Vpreb1a	V-set pre-B cell surrogate light chain 1A [Source:MGI Symbol;Acc:MGI:98936]
16	protein_coding	16688600	16710854	ENSMUSG00000022779	MGI:1333803	Top3b	topoisomerase (DNA) III beta [Source:MGI Symbol;Acc:MGI:1333803]
16	protein_coding	16714333	16745228	ENSMUSG00000026181	MGI:1918464	Ppm1f	protein phosphatase 1F (PP2C domain containing) [Source:MGI Symbol;Acc:MGI:1918464]
16	protein_coding	16801246	16865317	ENSMUSG00000063358	MGI:1346858	Mapk1	mitogen-activated protein kinase 1 [Source:MGI Symbol;Acc:MGI:1346858]
16	protein_coding	16887560	16904909	ENSMUSG00000022773	MGI:1913303	Ypel1	yippee like 1 [Source:MGI Symbol;Acc:MGI:1913303]
16	protein_coding	16904419	16929121	ENSMUSG00000022771	MGI:2447857	Ppil2	peptidylprolyl isomerase (cyclophilin)-like 2 [Source:MGI Symbol;Acc:MGI:2447857]
16	protein_coding	16931262	16943031	ENSMUSG00000049916	MGI:1917708	2610318N02Rik	RIKEN cDNA 2610318N02 gene [Source:MGI Symbol;Acc:MGI:1917708]

16	protein_coding	16948002	16950247	ENSMUSG00000022769	MGI:2149842	Sdf2l1	stromal cell-derived factor 2-like 1 [Source:MGI Symbol;Acc:MGI:2149842]
16	protein_coding	16956928	16965093	ENSMUSG00000022768	MGI:1924122	Ccdc116	coiled-coil domain containing 116 [Source:MGI Symbol;Acc:MGI:1924122]
16	protein_coding	16962485	16978565	ENSMUSG00000041774	MGI:1916351	Ydjc	YdjC homolog (bacterial) [Source:MGI Symbol;Acc:MGI:1916351]
16	protein_coding	16969877	17020513	ENSMUSG00000038965	MGI:109240	Ube2l3	ubiquitin-conjugating enzyme E2L 3 [Source:MGI Symbol;Acc:MGI:109240]
16	protein_coding	17026467	17031846	ENSMUSG00000071636	MGI:2685449	Rimbp3	RIMS binding protein 3 [Source:MGI Symbol;Acc:MGI:2685449]
16	protein_coding	17051436	17081294	ENSMUSG00000050240	MGI:1929869	Hic2	hypermethylated in cancer 2 [Source:MGI Symbol;Acc:MGI:1929869]
16	protein_coding	17093941	17101093	ENSMUSG00000055692	MGI:107238	Tmem191	transmembrane protein 191 [Source:MGI Symbol;Acc:MGI:107238]
16	protein_coding	17098215	17224178	ENSMUSG00000041720	MGI:2448506	Pi4ka	phosphatidylinositol 4-kinase alpha [Source:MGI Symbol;Acc:MGI:2448506]
16	protein_coding	17149235	17161439	ENSMUSG00000022766	MGI:96051	Serpind1	serine (or cysteine) peptidase inhibitor, clade D, member 1 [Source:MGI Symbol;Acc:MGI:96051]
16	protein_coding	17223850	17248691	ENSMUSG00000022765	MGI:1914724	Snap29	synaptosomal-associated protein 29 [Source:MGI Symbol;Acc:MGI:1914724]
16	protein_coding	17269851	17305298	ENSMUSG00000006134	MGI:104686	Crkl	v-crk avian sarcoma virus CT10 oncogene homolog-like [Source:MGI Symbol;Acc:MGI:104686]
16	protein_coding	17307475	17325349	ENSMUSG00000022763	MGI:1919418	Aifm3	apoptosis-inducing factor, mitochondrion-associated 3 [Source:MGI Symbol;Acc:MGI:1919418]
16	protein_coding	17326552	17344197	ENSMUSG00000022761	MGI:1914113	Lztr1	leucine-zipper-like transcriptional regulator, 1 [Source:MGI Symbol;Acc:MGI:1914113]

16	protein_coding	17345846	17349000	ENSMUSG00000022760	MGI:1916259	Thap7	THAP domain containing 7 [Source:MGI Symbol;Acc:MGI:1916259]
16	protein_coding	17362329	17379111	ENSMUSG00000022759	MGI:1921935	Lrrc74b	leucine rich repeat containing 74B [Source:MGI Symbol;Acc:MGI:1921935]
16	protein_coding	17379749	17395664	ENSMUSG00000022758	MGI:1337113	P2rx6	purinergic receptor P2X, ligand-gated ion channel, 6 [Source:MGI Symbol;Acc:MGI:1337113]
16	protein_coding	17389882	17401078	ENSMUSG00000022756	MGI:2146512	Slc7a4	solute carrier family 7 (cationic amino acid transporter, y+ system), member 4 [Source:MGI Symbol;Acc:MGI:2146512]
16	protein_coding	17437218	17462692	ENSMUSG00000005899	MGI:1924876	Smpd4	sphingomyelin phosphodiesterase 4 [Source:MGI Symbol;Acc:MGI:1924876]
16	protein_coding	17464334	17468602	ENSMUSG00000041617	MGI:1919565	Ccdc74a	coiled-coil domain containing 74A [Source:MGI Symbol;Acc:MGI:1919565]
16	protein_coding	17469072	17550755	ENSMUSG00000012114	MGI:2137379	Med15	mediator complex subunit 15 [Source:MGI Symbol;Acc:MGI:2137379]
16	protein_coding	17577482	17611246	ENSMUSG00000022750	MGI:1337995	Klhl22	kelch-like 22 [Source:MGI Symbol;Acc:MGI:1337995]
16	protein_coding	17615146	17626157	ENSMUSG00000012017	MGI:1858430	Scarf2	scavenger receptor class F, member 2 [Source:MGI Symbol;Acc:MGI:1858430]
16	protein_coding	17650985	17652872	ENSMUSG00000116652	MGI:3045365	Fam246a	family with sequence similarity 246 member A [Source:MGI Symbol;Acc:MGI:3045365]
16	protein_coding	17653140	17656228	ENSMUSG00000090236	MGI:1931324	Car15	carbonic anhydrase 15 [Source:MGI Symbol;Acc:MGI:1931324]
16	protein_coding	17653141	17709535	ENSMUSG00000092470	MGI:5141983	Gm20518	predicted gene 20518 [Source:MGI Symbol;Acc:MGI:5141983]
16	protein_coding	17657346	17716426	ENSMUSG00000003166	MGI:892866	Dgcr2	DiGeorge syndrome critical region gene 2 [Source:MGI Symbol;Acc:MGI:892866]

16	protein_coding	17712087	17715786	ENSMUSG00000041566	MGI:1347557	Tssk1	testis-specific serine kinase 1 [Source:MGI Symbol;Acc:MGI:1347557]
16	protein_coding	17716501	17717888	ENSMUSG00000045521	MGI:1347559	Tssk2	testis-specific serine kinase 2 [Source:MGI Symbol;Acc:MGI:1347559]
16	protein_coding	17718573	17729212	ENSMUSG00000003527	MGI:107854	Ess2	ess-2 splicing factor [Source:MGI Symbol;Acc:MGI:107854]
16	protein_coding	17730971	17732923	ENSMUSG00000022738	MGI:892006	Gsc2	goosecoid homebox 2 [Source:MGI Symbol;Acc:MGI:892006]
16	protein_coding	17743087	17746083	ENSMUSG00000003528	MGI:1345283	Slc25a1	solute carrier family 25 (mitochondrial carrier, citrate transporter), member 1 [Source:MGI Symbol;Acc:MGI:1345283]
16	protein_coding	17798292	17799137	ENSMUSG00000059280	MGI:98937	Vpreb1b	V-set pre-B cell surrogate light chain 1B [Source:MGI Symbol;Acc:MGI:98937]
16	protein_coding	17870724	17889496	ENSMUSG00000003531	MGI:1202877	Dgcr6	DiGeorge syndrome critical region gene 6 [Source:MGI Symbol;Acc:MGI:1202877]
16	protein_coding	17878221	17908067	ENSMUSG00000003526	MGI:97770	Prodh	proline dehydrogenase [Source:MGI Symbol;Acc:MGI:97770]
16	protein_coding	17945506	17970272	ENSMUSG00000043811	MGI:2136886	Rtn4r	reticulon 4 receptor [Source:MGI Symbol;Acc:MGI:2136886]
16	protein_coding	18035743	18038212	ENSMUSG00000090777	MGI:3647803	Ccdc188	coiled-coil domain containing 188 [Source:MGI Symbol;Acc:MGI:3647803]
16	protein_coding	18038617	18053000	ENSMUSG00000060166	MGI:1338012	Zdhhc8	zinc finger, DHHC domain containing 8 [Source:MGI Symbol;Acc:MGI:1338012]
16	protein_coding	18057648	18066596	ENSMUSG00000005732	MGI:96269	Ranbp1	RAN binding protein 1 [Source:MGI Symbol;Acc:MGI:96269]
16	protein_coding	18063079	18076185	ENSMUSG00000116925	MGI:6215291	Gm49776	predicted gene, 49776 [Source:MGI Symbol;Acc:MGI:6215291]
16	protein_coding	18066543	18072636	ENSMUSG00000022721	MGI:96270	Trmt2a	TRM2 tRNA methyltransferase 2A [Source:MGI Symbol;Acc:MGI:96270]

16	protein_coding	18071812	18107110	ENSMUSG00000022718	MGI:2151114	Dgcr8	DGCR8, microprocessor complex subunit [Source:MGI Symbol;Acc:MGI:2151114]
16	protein_coding	18118689	18165967	ENSMUSG00000013539	MGI:101825	Tango2	transport and golgi organization 2 [Source:MGI Symbol;Acc:MGI:101825]
16	protein_coding	18166046	18225826	ENSMUSG00000118669	MGI:109620	Arvcf	armadillo repeat gene deleted in velocardiofacial syndrome [Source:MGI Symbol;Acc:MGI:109620]
16	protein_coding	18225636	18245602	ENSMUSG00000000326	MGI:88470	Comt	catechol-O-methyltransferase [Source:MGI Symbol;Acc:MGI:88470]
16	protein_coding	18245134	18297823	ENSMUSG00000075704	MGI:1347023	Txnrd2	thioredoxin reductase 2 [Source:MGI Symbol;Acc:MGI:1347023]
16	protein_coding	18317463	18385429	ENSMUSG00000000884	MGI:1338057	Gnb1l	guanine nucleotide binding protein (G protein), beta polypeptide 1-like [Source:MGI Symbol;Acc:MGI:1338057]
16	protein_coding	18399729	18409421	ENSMUSG00000009097	MGI:98493	Tbx1	T-box 1 [Source:MGI Symbol;Acc:MGI:98493]
16	protein_coding	18439067	18441153	ENSMUSG00000050761	MGI:107852	Gp1bb	glycoprotein Ib, beta polypeptide [Source:MGI Symbol;Acc:MGI:107852]
16	protein_coding	18439216	18445177	ENSMUSG00000116594	MGI:6215011	Gm49601	predicted gene, 49601 [Source:MGI Symbol;Acc:MGI:6215011]
16	protein_coding	18439252	18448704	ENSMUSG00000072214	MGI:1195461	Septin5	septin 5 [Source:MGI Symbol;Acc:MGI:1195461]
16	protein_coding	18595597	18597012	ENSMUSG00000041378	MGI:1276112	Cldn5	claudin 5 [Source:MGI Symbol;Acc:MGI:1276112]
16	protein_coding	18599197	18630737	ENSMUSG00000000028	MGI:1338073	Cdc45	cell division cycle 45 [Source:MGI Symbol;Acc:MGI:1338073]
16	protein_coding	18630529	18654011	ENSMUSG00000005262	MGI:109353	Ufd1	ubiquitin recognition factor in ER-associated degradation 1 [Source:MGI Symbol;Acc:MGI:109353]
16	protein_coding	18655328	18658910	ENSMUSG00000071632	MGI:1919557	2510002D24Rik	RIKEN cDNA 2510002D24 gene [Source:MGI Symbol;Acc:MGI:1919557]
16	protein_coding	18655334	18774997	ENSMUSG00000099908	MGI:5579245	Gm28539	predicted gene 28539 [Source:MGI Symbol;Acc:MGI:5579245]

16	protein_coding	18690768	18695612	ENSMUSG00000022706	MGI:1332635	Mrpl40	mitochondrial ribosomal protein L40 [Source:MGI Symbol;Acc:MGI:1332635]
16	protein_coding	18695787	18789059	ENSMUSG00000022702	MGI:99430	Hira	histone cell cycle regulator [Source:MGI Symbol;Acc:MGI:99430]
16	protein_coding	19102854	19132814	ENSMUSG00000050742	MGI:3029998	Or2m12	olfactory receptor family 2 subfamily M member 12 [Source:MGI Symbol;Acc:MGI:3029998]
16	protein_coding	19224994	19241884	ENSMUSG00000050158	MGI:3029999	Or2m13	olfactory receptor family 2 subfamily M member 13 [Source:MGI Symbol;Acc:MGI:3029999]
16	protein_coding	19302444	19308632	ENSMUSG00000056822	MGI:3030000	Or2l13	olfactory receptor family 2 subfamily L member 13 [Source:MGI Symbol;Acc:MGI:3030000]
16	protein_coding	19328308	19341016	ENSMUSG00000045341	MGI:3030001	Or2l5	olfactory receptor family 2 subfamily L member 5 [Source:MGI Symbol;Acc:MGI:3030001]
16	protein_coding	19348024	19354305	ENSMUSG00000061361	MGI:3030002	Or2l13b	olfactory receptor family 2 subfamily L member 13B [Source:MGI Symbol;Acc:MGI:3030002]
16	protein_coding	19383639	19390559	ENSMUSG00000068535	MGI:3030003	Or2aj4	olfactory receptor family 2 subfamily AJ member 4 [Source:MGI Symbol;Acc:MGI:3030003]
16	protein_coding	19424446	19430632	ENSMUSG00000062245	MGI:3030004	Or2aj5	olfactory receptor family 2 subfamily AJ member 5 [Source:MGI Symbol;Acc:MGI:3030004]
16	protein_coding	19442680	19448666	ENSMUSG00000060480	MGI:3030005	Or2aj6	olfactory receptor family 2 subfamily AJ member 6 [Source:MGI Symbol;Acc:MGI:3030005]
16	protein_coding	19472128	19525125	ENSMUSG00000041247	MGI:2441659	Lamp3	lysosomal-associated membrane protein 3 [Source:MGI Symbol;Acc:MGI:2441659]
16	protein_coding	19578958	19591503	ENSMUSG00000022686	MGI:2137302	B3gnt5	UDP-GlcNAc:betaGal beta-1,3-N-acetylglucosaminyltransferase 5 [Source:MGI Symbol;Acc:MGI:2137302]

16	protein_coding	19695317	19703024	ENSMUSG00000075330	MGI:1915412	A930003A15Rik	RIKEN cDNA A930003A15 gene [Source:MGI Symbol;Acc:MGI:1915412]
16	protein_coding	19765246	19801787	ENSMUSG00000043008	MGI:2686922	Klhl6	kelch-like 6 [Source:MGI Symbol;Acc:MGI:2686922]
16	protein_coding	19916292	19947971	ENSMUSG00000062901	MGI:1923035	Klhl24	kelch-like 24 [Source:MGI Symbol;Acc:MGI:1923035]
16	protein_coding	19959813	20051323	ENSMUSG00000041215	MGI:2447762	Yeats2	YEATS domain containing 2 [Source:MGI Symbol;Acc:MGI:2447762]
16	protein_coding	20052053	20060108	ENSMUSG00000041205	MGI:3607784	Map6d1	MAP6 domain containing 1 [Source:MGI Symbol;Acc:MGI:3607784]
16	protein_coding	20098568	20121137	ENSMUSG00000033918	MGI:1277152	Parl	presenilin associated, rhomboid-like [Source:MGI Symbol;Acc:MGI:1277152]
16	protein_coding	20127137	20144154	ENSMUSG00000022818	MGI:3644957	Cyp2ab1	cytochrome P450, family 2, subfamily ab, polypeptide 1 [Source:MGI Symbol;Acc:MGI:3644957]
16	protein_coding	20150053	20245144	ENSMUSG00000022822	MGI:1351644	Abcc5	ATP-binding cassette, sub-family C member 5 [Source:MGI Symbol;Acc:MGI:1351644]
16	protein_coding	20317567	20328073	ENSMUSG00000003235	MGI:2446176	Eif2b5	eukaryotic translation initiation factor 2B, subunit 5 epsilon [Source:MGI Symbol;Acc:MGI:2446176]
16	protein_coding	20335732	20352760	ENSMUSG00000003233	MGI:108100	Dvl3	dishevelled segment polarity protein 3 [Source:MGI Symbol;Acc:MGI:108100]
16	protein_coding	20354228	20363659	ENSMUSG00000022841	MGI:1298405	Ap2m1	adaptor-related protein complex 2, mu 1 subunit [Source:MGI Symbol;Acc:MGI:1298405]
16	protein_coding	20367327	20380129	ENSMUSG00000003234	MGI:1351656	Abcf3	ATP-binding cassette, sub-family F member 3 [Source:MGI Symbol;Acc:MGI:1351656]
16	protein_coding	20408221	20424127	ENSMUSG00000046613	MGI:2681859	Vwa5b2	von Willebrand factor A domain containing 5B2 [Source:MGI Symbol;Acc:MGI:2681859]

16	protein_coding	20424124	20430485	ENSMUSG00000033809	MGI:1098592	Alg3	ALG3 alpha-1,3- mannosyltransferase [Source:MGI Symbol;Acc:MGI:1098592]
16	protein_coding	20430343	20437761	ENSMUSG00000115219	MGI:5903914	Eef1akmt4	EEF1A lysine methyltransferase 4 [Source:MGI Symbol;Acc:MGI:5903914]
16	protein_coding	20430360	20464670	ENSMUSG00000115293	MGI:6121518	Eef1ece2	Eef1akmt4-endothelin converting enzyme 2 readthrough [Source:MGI Symbol;Acc:MGI:6121518]
16	protein_coding	20437965	20440037	ENSMUSG00000051146	MGI:1920297	Camk2n2	calcium/calmodulin-dependent protein kinase II inhibitor 2 [Source:MGI Symbol;Acc:MGI:1920297]
16	protein_coding	20448578	20465235	ENSMUSG00000022842	MGI:1101356	Ece2	endothelin converting enzyme 2 [Source:MGI Symbol;Acc:MGI:1101356]
16	protein_coding	20470402	20482164	ENSMUSG00000006998	MGI:1096584	Psmd2	proteasome (prosome, macropain) 26S subunit, non-ATPase, 2 [Source:MGI Symbol;Acc:MGI:1096584]
16	protein_coding	20487063	20511634	ENSMUSG00000045983	MGI:2384784	Eif4g1	eukaryotic translation initiation factor 4, gamma 1 [Source:MGI Symbol;Acc:MGI:2384784]
16	protein_coding	20511991	20521798	ENSMUSG00000050821	MGI:1925658	Fam131a	family with sequence similarity 131, member A [Source:MGI Symbol;Acc:MGI:1925658]
16	protein_coding	20521714	20536496	ENSMUSG00000022843	MGI:105061	Clcn2	chloride channel, voltage-sensitive 2 [Source:MGI Symbol;Acc:MGI:105061]
16	protein_coding	20536415	20541017	ENSMUSG00000021018	MGI:2384309	Polr2h	polymerase (RNA) II (DNA directed) polypeptide H [Source:MGI Symbol;Acc:MGI:2384309]
16	protein_coding	20543204	20553261	ENSMUSG00000022847	MGI:101875	Thpo	thrombopoietin [Source:MGI Symbol;Acc:MGI:101875]
16	protein_coding	20551877	20561134	ENSMUSG00000006958	MGI:1313268	Chrd	chordin [Source:MGI Symbol;Acc:MGI:1313268]
16	protein_coding	20971405	20972750	ENSMUSG00000090356	MGI:1913811	Teddm3	transmembrane epididymal family member 3 [Source:MGI Symbol;Acc:MGI:1913811]

16	protein_coding	21023505	21042055	ENSMUSG00000005958	MGI:104770	Ephb3	Eph receptor B3 [Source:MGI Symbol;Acc:MGI:104770]
16	protein_coding	21241868	21463430	ENSMUSG00000033653	MGI:2146407	Vps8	VPS8 CORVET complex subunit [Source:MGI Symbol;Acc:MGI:2146407]
16	protein_coding	21467795	21513415	ENSMUSG00000043391	MGI:1919440	2510009E07Rik	RIKEN cDNA 2510009E07 gene [Source:MGI Symbol;Acc:MGI:1919440]
16	rRNA	8690943	8691035	ENSMUSG00002075441	MGI:6846556	Gm55041	predicted gene, 55041 [Source:MGI Symbol;Acc:MGI:6846556]
16	rRNA	13787212	13787321	ENSMUSG00002076461	MGI:6845244	Gm54382	predicted gene, 54382 [Source:MGI Symbol;Acc:MGI:6845244]
16	rRNA	15828764	15828873	ENSMUSG00002076768	MGI:6845344	Gm54432	predicted gene, 54432 [Source:MGI Symbol;Acc:MGI:6845344]
16	snoRNA	8220678	8220821	ENSMUSG00000077152	MGI:5454356	Gm24579	predicted gene, 24579 [Source:MGI Symbol;Acc:MGI:5454356]
16	snoRNA	8298524	8298605	ENSMUSG00000077557	MGI:5453483	Gm23706	predicted gene, 23706 [Source:MGI Symbol;Acc:MGI:5453483]
16	snoRNA	13349292	13349415	ENSMUSG00000077488	MGI:5455053	Gm25276	predicted gene, 25276 [Source:MGI Symbol;Acc:MGI:5455053]
16	snoRNA	18058581	18058706	ENSMUSG00000080364	MGI:5455554	Gm25777	predicted gene, 25777 [Source:MGI Symbol;Acc:MGI:5455554]
16	snoRNA	19126449	19126574	ENSMUSG00000089036	MGI:5451935	Gm22158	predicted gene, 22158 [Source:MGI Symbol;Acc:MGI:5451935]
16	snoRNA	19229908	19230039	ENSMUSG00000077172	MGI:5455539	Gm25762	predicted gene, 25762 [Source:MGI Symbol;Acc:MGI:5455539]
16	snoRNA	20502989	20503063	ENSMUSG00000077239	MGI:3819548	Snord66	small nucleolar RNA, C/D box 66 [Source:MGI Symbol;Acc:MGI:3819548]
16	snRNA	8154857	8154960	ENSMUSG00000089593	MGI:5455936	Gm26159	predicted gene, 26159 [Source:MGI Symbol;Acc:MGI:5455936]
16	snRNA	8584033	8584140	ENSMUSG00000084737	MGI:5455582	Gm25805	predicted gene, 25805 [Source:MGI Symbol;Acc:MGI:5455582]
16	snRNA	10055782	10055884	ENSMUSG00000089563	MGI:5454444	Gm24667	predicted gene, 24667 [Source:MGI Symbol;Acc:MGI:5454444]

16	snRNA	10812250	10812433	ENSMUSG00002075097	MGI:6846704	Gm55115	predicted gene, 55115 [Source:MGI Symbol;Acc:MGI:6846704]
16	snRNA	10984969	10985131	ENSMUSG00000064565	MGI:5453922	Gm24145	predicted gene, 24145 [Source:MGI Symbol;Acc:MGI:5453922]
16	snRNA	14200671	14200737	ENSMUSG00000089106	MGI:5452893	Gm23116	predicted gene, 23116 [Source:MGI Symbol;Acc:MGI:5452893]
16	snRNA	14625027	14625133	ENSMUSG00000118928	MGI:5455324	Gm25547	predicted gene, 25547 [Source:MGI Symbol;Acc:MGI:5455324]
16	snRNA	16823971	16824065	ENSMUSG00002076351	MGI:6845440	Gm54480	predicted gene, 54480 [Source:MGI Symbol;Acc:MGI:6845440]
16	snRNA	17023656	17023762	ENSMUSG00000119426	MGI:5451906	Gm22129	predicted gene, 22129 [Source:MGI Symbol;Acc:MGI:5451906]
16	snRNA	17159770	17159876	ENSMUSG00000065331	MGI:5454704	Gm24927	predicted gene, 24927 [Source:MGI Symbol;Acc:MGI:5454704]
16	snRNA	17300947	17301013	ENSMUSG00002076579	MGI:6845504	Gm54512	predicted gene, 54512 [Source:MGI Symbol;Acc:MGI:6845504]
16	snRNA	18173480	18173627	ENSMUSG00002075609	MGI:6847209	Gm55369	predicted gene, 55369 [Source:MGI Symbol;Acc:MGI:6847209]
16	snRNA	20564058	20564222	ENSMUSG00000065372	MGI:5452518	Gm22741	predicted gene, 22741 [Source:MGI Symbol;Acc:MGI:5452518]
16	TEC	17739907	17741083	ENSMUSG00000103149	MGI:1858439	AA914427	EST AA914427 [Source:MGI Symbol;Acc:MGI:1858439]
16	TEC	18300806	18305295	ENSMUSG00000116842	MGI:6214991	Gm49588	predicted gene, 49588 [Source:MGI Symbol;Acc:MGI:6214991]
16	TEC	19050083	19053552	ENSMUSG00000105128	MGI:5663007	Gm42870	predicted gene 42870 [Source:MGI Symbol;Acc:MGI:5663007]
16	TEC	19062275	19063584	ENSMUSG00000105160	MGI:2444617	A530030E21Rik	RIKEN cDNA A530030E21 gene [Source:MGI Symbol;Acc:MGI:2444617]
16	transcribed_processed_pseudogene	10864768	10866154	ENSMUSG00000116274	MGI:5010909	Gm18724	predicted gene, 18724 [Source:MGI Symbol;Acc:MGI:5010909]

16	transcribed_ processed_p seudogene	13495005	13495835	ENSMUSG00000079737	MGI:1913848	Pphln1-ps1	periphilin 1, pseudogene 1 [Source:MGI Symbol;Acc:MGI:1913848]
16	transcribed_ processed_p seudogene	16183048	16184021	ENSMUSG00000098037	MGI:3645843	Gm7765	predicted gene 7765 [Source:MGI Symbol;Acc:MGI:3645843]
16	transcribed_ processed_p seudogene	16637613	16637987	ENSMUSG00000116648	MGI:3645866	Rpl31-ps12	ribosomal protein L31, pseudogene 1 2 [Source:MGI Symbol;Acc:MGI:3645866]
16	transcribed_ processed_p seudogene	19995477	19995762	ENSMUSG00000084838	MGI:3704461	Gm10241	predicted pseudogene 10241 [Source:MGI Symbol;Acc:MGI:3704461]
16	transcribed_ processed_p seudogene	20364668	20364950	ENSMUSG00000080797	MGI:3642834	Gm15760	predicted gene 15760 [Source:MGI Symbol;Acc:MGI:3642834]
16	transcribed_ processed_p seudogene	20639131	20639453	ENSMUSG00000116721	MGI:3779946	Gm9536	predicted gene 9536 [Source:MGI Symbol;Acc:MGI:3779946]
16	transcribed_ processed_p seudogene	21151396	21151879	ENSMUSG00000116632	MGI:1923472	Magef1	MAGE family member F1 [Source:MGI Symbol;Acc:MGI:1923472]

16	transcribed_unitary_pseudogene	10679619	10756228	ENSMUSG00000117309	MGI:6270684	Gm49960	predicted gene, 49960 [Source:MGI Symbol;Acc:MGI:6270684]
16	transcribed_unitary_pseudogene	18319883	18320924	ENSMUSG00000086965	MGI:3833934	Rtl10	retrotransposon Gag like 10 [Source:MGI Symbol;Acc:MGI:3833934]
16	transcribed_unprocessed_pseudogene	20307538	20309897	ENSMUSG00000093381	MGI:5313062	Gm20615	predicted gene 20615 [Source:MGI Symbol;Acc:MGI:5313062]
16	transcribed_unprocessed_pseudogene	20747719	20748603	ENSMUSG00000116659	MGI:3644103	Gm6551	predicted gene 6551 [Source:MGI Symbol;Acc:MGI:3644103]
16	transcribed_unprocessed_pseudogene	20880963	20881844	ENSMUSG00000116710	MGI:3779606	Gm6557	predicted gene 6557 [Source:MGI Symbol;Acc:MGI:3779606]
16	unprocessed_pseudogene	13737291	13739263	ENSMUSG00000089818	MGI:3802178	Gm15950	predicted gene 15950 [Source:MGI Symbol;Acc:MGI:3802178]
16	unprocessed_pseudogene	14297762	14301610	ENSMUSG00000116439	MGI:6155229	Gm49528	predicted gene, 49528 [Source:MGI Symbol;Acc:MGI:6155229]

16	unprocessed_pseudogene	14319964	14327085	ENSMUSG00000090808	MGI:2686604	Gm1758	predicted gene 1758 [Source:MGI Symbol;Acc:MGI:2686604]
16	unprocessed_pseudogene	16490254	16490374	ENSMUSG00000108454	MGI:5753508	Gm44932	predicted gene 44932 [Source:MGI Symbol;Acc:MGI:5753508]
16	unprocessed_pseudogene	20254788	20262132	ENSMUSG00000093649	MGI:5313061	Gm20614	predicted gene 20614 [Source:MGI Symbol;Acc:MGI:5313061]

Supplementary Table 4. Protein-coding genes located within ±10 Mb of pathology peak QTL on chromosome 5 (peak~98.3Mb)

Chr	type	start	stop	gene_id	mgi_type	gene_symbol	description
5	protein_coding	88032888	88042033	ENSMUSG00000009580	MGI:1916842	Odam	odontogenic, ameloblast asssociated [Source:MGI Symbol;Acc:MGI:1916842]
5	protein_coding	88056638	88062030	ENSMUSG00000105888	MGI:3512757	Fdcsp	follicular dendritic cell secreted protein [Source:MGI Symbol;Acc:MGI:3512757]
5	protein_coding	88073438	88080524	ENSMUSG00000001622	MGI:107461	Csn3	casein kappa [Source:MGI Symbol;Acc:MGI:107461]
5	protein_coding	88117318	88120729	ENSMUSG00000007457	MGI:1921498	2310003L06Rik	RIKEN cDNA 2310003L06 gene [Source:MGI Symbol;Acc:MGI:1921498]
5	protein_coding	88117376	88156136	ENSMUSG00000100704	MGI:5579140	Gm28434	predicted gene 28434 [Source:MGI Symbol;Acc:MGI:5579140]
5	protein_coding	88127298	88129403	ENSMUSG00000007907	MGI:1918227	Cabs1	calcium binding protein, spermatid specific 1 [Source:MGI Symbol;Acc:MGI:1918227]
5	protein_coding	88150408	88156393	ENSMUSG00000029280	MGI:102763	Smr3a	submaxillary gland androgen regulated protein 3A [Source:MGI Symbol;Acc:MGI:102763]
5	protein_coding	88234415	88256912	ENSMUSG00000029281	MGI:102762	Smr2	submaxillary gland androgen regulated protein 2 [Source:MGI Symbol;Acc:MGI:102762]
5	protein_coding	88416778	88430694	ENSMUSG00000090302	MGI:3646267	Smr2l	submaxillary gland androgen regulated protein 2 like [Source:MGI Symbol;Acc:MGI:3646267]
5	protein_coding	88465171	88476673	ENSMUSG00000064156	MGI:107496	Prol1	proline rich, lacrimal 1 [Source:MGI Symbol;Acc:MGI:107496]
5	protein_coding	88523967	88533775	ENSMUSG00000029282	MGI:1918671	Amtn	amelotin [Source:MGI Symbol;Acc:MGI:1918671]
5	protein_coding	88603850	88616390	ENSMUSG00000029288	MGI:104655	Ambn	ameloblastin [Source:MGI Symbol;Acc:MGI:104655]
5	protein_coding	88635834	88653908	ENSMUSG00000029286	MGI:1333772	Enam	enamelin [Source:MGI Symbol;Acc:MGI:1333772]
5	protein_coding	88667668	88675750	ENSMUSG00000067149	MGI:96493	Jchain	immunoglobulin joining chain [Source:MGI Symbol;Acc:MGI:96493]
5	protein_coding	88702321	88703949	ENSMUSG00000070697	MGI:1919230	Utp3	UTP3 small subunit processome component [Source:MGI Symbol;Acc:MGI:1919230]

5	protein_coding	88712899	88799251	ENSMUSG00000029291	MGI:106484	Rufy3	RUN and FYVE domain containing 3 [Source:MGI Symbol;Acc:MGI:106484]
5	protein_coding	88807307	88824030	ENSMUSG00000044221	MGI:106479	Grsf1	G-rich RNA sequence binding factor 1 [Source:MGI Symbol;Acc:MGI:106479]
5	protein_coding	88868714	88912079	ENSMUSG00000006262	MGI:1915723	Mob1b	MOB kinase activator 1B [Source:MGI Symbol;Acc:MGI:1915723]
5	protein_coding	88912855	88931140	ENSMUSG00000029366	MGI:102726	Dck	deoxycytidine kinase [Source:MGI Symbol;Acc:MGI:102726]
5	protein_coding	89034345	89387512	ENSMUSG00000060961	MGI:1927555	Slc4a4	solute carrier family 4 (anion exchanger), member 4 [Source:MGI Symbol;Acc:MGI:1927555]
5	protein_coding	89565381	89605757	ENSMUSG00000035540	MGI:95669	Gc	vitamin D binding protein [Source:MGI Symbol;Acc:MGI:95669]
5	protein_coding	89675288	89731599	ENSMUSG00000035528	MGI:1860130	Npffr2	neuropeptide FF receptor 2 [Source:MGI Symbol;Acc:MGI:1860130]
5	protein_coding	89824946	90031193	ENSMUSG00000043635	MGI:3045353	Adamts3	ADAM metallopeptidase with thrombospondin type 1 motif 3 [Source:MGI Symbol;Acc:MGI:3045353]
5	protein_coding	90362583	90371860	ENSMUSG00000035505	MGI:2448532	Cox18	cytochrome c oxidase assembly protein 18 [Source:MGI Symbol;Acc:MGI:2448532]
5	protein_coding	90375025	90514436	ENSMUSG00000055204	MGI:1932101	Ankrd17	ankyrin repeat domain 17 [Source:MGI Symbol;Acc:MGI:1932101]
5	protein_coding	90608756	90624461	ENSMUSG00000029368	MGI:87991	Alb	albumin [Source:MGI Symbol;Acc:MGI:87991]
5	protein_coding	90638596	90656766	ENSMUSG00000054932	MGI:87951	Afp	alpha fetoprotein [Source:MGI Symbol;Acc:MGI:87951]
5	protein_coding	90666791	90701402	ENSMUSG00000029369	MGI:2429409	Afm	afamin [Source:MGI Symbol;Acc:MGI:2429409]
5	protein_coding	90708966	90745730	ENSMUSG00000070690	MGI:1923342	Albfm1	albumin superfamily member 1 [Source:MGI Symbol;Acc:MGI:1923342]
5	protein_coding	90750935	90788516	ENSMUSG00000029370	MGI:1920496	Rassf6	Ras association (RalGDS/AF-6) domain family member 6 [Source:MGI Symbol;Acc:MGI:1920496]
5	protein_coding	90907219	90909483	ENSMUSG00000029371	MGI:1096868	Cxcl5	C-X-C motif chemokine ligand 5 [Source:MGI Symbol;Acc:MGI:1096868]

5	protein_coding	90916377	90917922	ENSMUSG00000029372	MGI:1888712	Ppbp	pro-platelet basic protein [Source:MGI Symbol;Acc:MGI:1888712]
5	protein_coding	90920294	90921242	ENSMUSG00000029373	MGI:1888711	Pf4	platelet factor 4 [Source:MGI Symbol;Acc:MGI:1888711]
5	protein_coding	90933962	90937459	ENSMUSG00000029379	MGI:3037818	Cxcl3	C-X-C motif chemokine ligand 3 [Source:MGI Symbol;Acc:MGI:3037818]
5	protein_coding	90942393	90950926	ENSMUSG00000029375	MGI:1339941	Cxcl15	C-X-C motif chemokine ligand 15 [Source:MGI Symbol;Acc:MGI:1339941]
5	protein_coding	91039100	91040974	ENSMUSG00000029380	MGI:108068	Cxcl1	C-X-C motif chemokine ligand 1 [Source:MGI Symbol;Acc:MGI:108068]
5	protein_coding	91051730	91053797	ENSMUSG00000058427	MGI:1340094	Cxcl2	C-X-C motif chemokine ligand 2 [Source:MGI Symbol;Acc:MGI:1340094]
5	protein_coding	91078976	91169227	ENSMUSG00000029376	MGI:1915871	Mthfd2l	methylenetetrahydrofolate dehydrogenase (NADP+ dependent) 2-like [Source:MGI Symbol;Acc:MGI:1915871]
5	protein_coding	91175323	91183074	ENSMUSG00000035020	MGI:1919170	Epgn	epithelial mitogen [Source:MGI Symbol;Acc:MGI:1919170]
5	protein_coding	91222481	91241505	ENSMUSG00000029377	MGI:107508	Ereg	epiregulin [Source:MGI Symbol;Acc:MGI:107508]
5	protein_coding	91287458	91296291	ENSMUSG00000029378	MGI:88068	Areg	amphiregulin [Source:MGI Symbol;Acc:MGI:88068]
5	protein_coding	91505120	91550853	ENSMUSG00000082361	MGI:99439	Btc	betacellulin, epidermal growth factor family member [Source:MGI Symbol;Acc:MGI:99439]
5	protein_coding	91665474	91774753	ENSMUSG00000034981	MGI:2443349	Parm1	prostate androgen-regulated mucin-like protein 1 [Source:MGI Symbol;Acc:MGI:2443349]
5	protein_coding	92096763	92110927	ENSMUSG00000029397	MGI:1915348	Rchy1	ring finger and CHY zinc finger domain containing 1 [Source:MGI Symbol;Acc:MGI:1915348]
5	protein_coding	92135334	92143179	ENSMUSG00000096035	MGI:2685891	Odaph	odontogenesis associated phosphoprotein [Source:MGI Symbol;Acc:MGI:2685891]
5	protein_coding	92153933	92191742	ENSMUSG00000029403	MGI:1858227	Cdkl2	cyclin dependent kinase like 2 [Source:MGI Symbol;Acc:MGI:1858227]
5	protein_coding	92200005	92231578	ENSMUSG00000029405	MGI:2442040	G3bp2	G3BP stress granule assembly factor 2 [Source:MGI Symbol;Acc:MGI:2442040]

5	protein_coding	92285797	92350657	ENSMUSG00000029407	MGI:1929095	Uso1	USO1 vesicle docking factor [Source:MGI Symbol;Acc:MGI:1929095]
5	protein_coding	92357752	92363267	ENSMUSG00000029409	MGI:1930915	U90926	cDNA sequence U90926 [Source:MGI Symbol;Acc:MGI:1930915]
5	protein_coding	92374538	92404137	ENSMUSG00000029410	MGI:1342304	Ppef2	protein phosphatase, EF hand calcium-binding domain 2 [Source:MGI Symbol;Acc:MGI:1342304]
5	protein_coding	92405518	92426029	ENSMUSG00000029413	MGI:1914361	Naaa	N-acylethanolamine acid amidase [Source:MGI Symbol;Acc:MGI:1914361]
5	protein_coding	92431869	92458338	ENSMUSG00000029415	MGI:2140779	Sdad1	SDA1 domain containing 1 [Source:MGI Symbol;Acc:MGI:2140779]
5	protein_coding	92469206	92475938	ENSMUSG00000029417	MGI:1352449	Cxcl9	C-X-C motif chemokine ligand 9 [Source:MGI Symbol;Acc:MGI:1352449]
5	protein_coding	92479686	92562487	ENSMUSG00000034842	MGI:1202729	Art3	ADP-ribosyltransferase 3 [Source:MGI Symbol;Acc:MGI:1202729]
5	protein_coding	92494497	92496748	ENSMUSG00000034855	MGI:1352450	Cxcl10	C-X-C motif chemokine ligand 10 [Source:MGI Symbol;Acc:MGI:1352450]
5	protein_coding	92507403	92513344	ENSMUSG00000060183	MGI:1860203	Cxcl11	chemokine (C-X-C motif) ligand 11 [Source:MGI Symbol;Acc:MGI:1860203]
5	protein_coding	92563399	92583078	ENSMUSG00000034826	MGI:1920460	Nup54	nucleoporin 54 [Source:MGI Symbol;Acc:MGI:1920460]
5	protein_coding	92589173	92654692	ENSMUSG00000029426	MGI:1196458	Scarb2	scavenger receptor class B, member 2 [Source:MGI Symbol;Acc:MGI:1196458]
5	protein_coding	92702928	92739138	ENSMUSG00000057068	MGI:2686227	Fam47e	family with sequence similarity 47, member E [Source:MGI Symbol;Acc:MGI:2686227]
5	protein_coding	92750900	92754438	ENSMUSG00000047963	MGI:1261768	Stbd1	starch binding domain 1 [Source:MGI Symbol;Acc:MGI:1261768]
5	protein_coding	92755813	92823130	ENSMUSG00000050050	MGI:2444555	Ccdc158	coiled-coil domain containing 158 [Source:MGI Symbol;Acc:MGI:2444555]
5	protein_coding	92831294	93113177	ENSMUSG00000029381	MGI:1351655	Shroom3	shroom family member 3 [Source:MGI Symbol;Acc:MGI:1351655]
5	protein_coding	93188982	93192881	ENSMUSG00000045314	MGI:1925338	Sowahb	sosondowah ankyrin repeat domain family member B [Source:MGI Symbol;Acc:MGI:1925338]
5	protein_coding	93241296	93324306	ENSMUSG00000058013	MGI:1277214	Septin11	septin 11 [Source:MGI Symbol;Acc:MGI:1277214]

5	protein_coding	93329792	93354354	ENSMUSG00000063015	MGI:1341077	Ccni	cyclin I [Source:MGI Symbol;Acc:MGI:1341077]
5	protein_coding	93354377	93361334	ENSMUSG00000029384	MGI:1922860	2010109A12Rik	RIKEN cDNA 2010109A12 gene [Source:MGI Symbol;Acc:MGI:1922860]
5	protein_coding	93415116	93424090	ENSMUSG00000029385	MGI:1095734	Ccng2	cyclin G2 [Source:MGI Symbol;Acc:MGI:1095734]
5	protein_coding	93629688	93688059	ENSMUSG00000096044	MGI:3704103	Pramel33	PRAME like 33 [Source:MGI Symbol;Acc:MGI:3704103]
5	protein_coding	93783056	93819410	ENSMUSG00000070686	MGI:2141341	Pramel34	PRAME like 34 [Source:MGI Symbol;Acc:MGI:2141341]
5	protein_coding	93991623	94003966	ENSMUSG00000107392	MGI:3704104	Pramel35	PRAME like 35 [Source:MGI Symbol;Acc:MGI:3704104]
5	protein_coding	94072636	94076911	ENSMUSG00000096230	MGI:3704105	Pramel36	PRAME like 36 [Source:MGI Symbol;Acc:MGI:3704105]
5	protein_coding	94214310	94218404	ENSMUSG00000072822	MGI:2681870	Pramel37	PRAME like 37 [Source:MGI Symbol;Acc:MGI:2681870]
5	protein_coding	94224244	94228794	ENSMUSG00000072821	MGI:3647817	Pramel53	PRAME like 53 [Source:MGI Symbol;Acc:MGI:3647817]
5	protein_coding	94304436	94369394	ENSMUSG00000096259	MGI:3781282	Pramel38	PRAME like 38 [Source:MGI Symbol;Acc:MGI:3781282]
5	protein_coding	94460534	94465114	ENSMUSG00000095718	MGI:3779601	Pramel40	PRAME like 40 [Source:MGI Symbol;Acc:MGI:3779601]
5	protein_coding	94591865	94596410	ENSMUSG00000074011	MGI:3779756	Pramel41	PRAME like 41 [Source:MGI Symbol;Acc:MGI:3779756]
5	protein_coding	94674146	94694282	ENSMUSG00000095074	MGI:3781318	Pramel42	PRAME like 42 [Source:MGI Symbol;Acc:MGI:3781318]
5	protein_coding	94759928	94764201	ENSMUSG00000095503	MGI:3781326	Pramel43	PRAME like 43 [Source:MGI Symbol;Acc:MGI:3781326]
5	protein_coding	94770074	94774605	ENSMUSG00000094195	MGI:3779602	Pramel44	PRAME like 44 [Source:MGI Symbol;Acc:MGI:3779602]
5	protein_coding	95013295	95033247	ENSMUSG00000095954	MGI:3781362	Pramel56	PRAME like 56 [Source:MGI Symbol;Acc:MGI:3781362]
5	protein_coding	95254563	95259017	ENSMUSG00000092166	MGI:3646599	Pramel45	PRAME like 45 [Source:MGI Symbol;Acc:MGI:3646599]
5	protein_coding	95330483	95342825	ENSMUSG00000095996	MGI:2141041	Pramel60	PRAME like 60 [Source:MGI Symbol;Acc:MGI:2141041]

5	protein_coding	95416352	95420627	ENSMUSG00000096066	MGI:3704250	Pramel46	PRAME like 46 [Source:MGI Symbol;Acc:MGI:3704250]
5	protein_coding	95453816	95491776	ENSMUSG00000079424	MGI:3781437	Pramel47	PRAME like 47 [Source:MGI Symbol;Acc:MGI:3781437]
5	protein_coding	95604665	95633446	ENSMUSG00000070677	MGI:1261918	Pramel48	PRAME like 48 [Source:MGI Symbol;Acc:MGI:1261918]
5	protein_coding	95658617	95670261	ENSMUSG00000079423	MGI:3781464	Pramel57	PRAME like 57 [Source:MGI Symbol;Acc:MGI:3781464]
5	protein_coding	95738458	95742734	ENSMUSG00000094043	MGI:3779772	Pramel49	PRAME like 49 [Source:MGI Symbol;Acc:MGI:3779772]
5	protein_coding	95879648	95884032	ENSMUSG00000096139	MGI:3704107	Pramel50	PRAME like 50 [Source:MGI Symbol;Acc:MGI:3704107]
5	protein_coding	95889873	95894414	ENSMUSG00000072814	MGI:3648891	Pramel54	PRAME like 54 [Source:MGI Symbol;Acc:MGI:3648891]
5	protein_coding	95923729	95952089	ENSMUSG00000072813	MGI:3615333	Pramel55	PRAME like 55 [Source:MGI Symbol;Acc:MGI:3615333]
5	protein_coding	96104810	96108927	ENSMUSG00000023078	MGI:1888499	Cxcl13	C-X-C motif chemokine ligand 13 [Source:MGI Symbol;Acc:MGI:1888499]
5	protein_coding	96218192	96312030	ENSMUSG00000034724	MGI:2443154	Cnot6l	CCR4-NOT transcription complex, subunit 6-like [Source:MGI Symbol;Acc:MGI:2443154]
5	protein_coding	96357352	96414586	ENSMUSG00000029486	MGI:2137202	Mrpl1	mitochondrial ribosomal protein L1 [Source:MGI Symbol;Acc:MGI:2137202]
5	protein_coding	96521814	96932587	ENSMUSG00000034687	MGI:2385368	Fras1	Fraser extracellular matrix complex subunit 1 [Source:MGI Symbol;Acc:MGI:2385368]
5	protein_coding	96941198	96993825	ENSMUSG00000029484	MGI:1201378	Anxa3	annexin A3 [Source:MGI Symbol;Acc:MGI:1201378]
5	protein_coding	97145548	97239726	ENSMUSG00000034663	MGI:2155456	Bmp2k	BMP2 inducible kinase [Source:MGI Symbol;Acc:MGI:2155456]
5	protein_coding	97230188	97259455	ENSMUSG00000055725	MGI:2679683	Paqr3	progestin and adipoQ receptor family member III [Source:MGI Symbol;Acc:MGI:2679683]
5	protein_coding	97530057	97540238	ENSMUSG00000046000	MGI:2141314	Naa11	N(alpha)-acetyltransferase 11, NatA catalytic subunit [Source:MGI Symbol;Acc:MGI:2141314]

5	protein_coding	97603001	97604880	ENSMUSG00000050553	MGI:1329027	Gk2	glycerol kinase 2 [Source:MGI Symbol;Acc:MGI:1329027]
5	protein_coding	98030642	98178902	ENSMUSG00000029338	MGI:1919164	Antxr2	anthrax toxin receptor 2 [Source:MGI Symbol;Acc:MGI:1919164]
5	protein_coding	98315057	98336850	ENSMUSG00000035456	MGI:1924880	Prdm8	PR domain containing 8 [Source:MGI Symbol;Acc:MGI:1924880]
5	protein_coding	98402043	98424889	ENSMUSG00000029337	MGI:95519	Fgf5	fibroblast growth factor 5 [Source:MGI Symbol;Acc:MGI:95519]
5	protein_coding	98477163	98949906	ENSMUSG00000057816	MGI:1916571	Cfap299	cilia and flagella associated protein 299 [Source:MGI Symbol;Acc:MGI:1916571]
5	protein_coding	99002274	99032255	ENSMUSG00000029335	MGI:88179	Bmp3	bone morphogenetic protein 3 [Source:MGI Symbol;Acc:MGI:88179]
5	protein_coding	99077632	99185210	ENSMUSG00000029334	MGI:108173	Prkg2	protein kinase, cGMP-dependent, type II [Source:MGI Symbol;Acc:MGI:108173]
5	protein_coding	99365285	99876924	ENSMUSG00000089809	MGI:2443755	Rasgef1b	RasGEF domain family, member 1B [Source:MGI Symbol;Acc:MGI:2443755]
5	protein_coding	1.00E+08	1.00E+08	ENSMUSG00000105078	MGI:5595070	Vamp9	vesicle-associated membrane protein 9 [Source:MGI Symbol;Acc:MGI:5595070]
5	protein_coding	1.00E+08	1.00E+08	ENSMUSG00000000568	MGI:101947	Hnrnpd	heterogeneous nuclear ribonucleoprotein D [Source:MGI Symbol;Acc:MGI:101947]
5	protein_coding	1.00E+08	1.00E+08	ENSMUSG00000029328	MGI:1355299	Hnrnpdl	heterogeneous nuclear ribonucleoprotein D-like [Source:MGI Symbol;Acc:MGI:1355299]
5	protein_coding	1.00E+08	1.00E+08	ENSMUSG00000029326	MGI:1915120	Enoph1	enolase-phosphatase 1 [Source:MGI Symbol;Acc:MGI:1915120]
5	protein_coding	1.00E+08	1.00E+08	ENSMUSG00000050640	MGI:3041258	Tmem150c	transmembrane protein 150C [Source:MGI Symbol;Acc:MGI:3041258]
5	protein_coding	1.01E+08	1.01E+08	ENSMUSG00000035325	MGI:1916412	Sec31a	SEC31 homolog A, COPII coat complex component [Source:MGI Symbol;Acc:MGI:1916412]
5	protein_coding	1.01E+08	1.01E+08	ENSMUSG00000118665	MGI:2140902	Lin54	lin-54 DREAM MuvB core complex component [Source:MGI Symbol;Acc:MGI:2140902]
5	protein_coding	1.01E+08	1.01E+08	ENSMUSG00000035297	MGI:1349414	Cops4	COP9 signalosome subunit 4 [Source:MGI Symbol;Acc:MGI:1349414]

5	protein_coding	1.01E+08	1.01E+08	ENSMUSG00000029322	MGI:2445289	Plac8	placenta-specific 8 [Source:MGI Symbol;Acc:MGI:2445289]
5	protein_coding	1.01E+08	1.01E+08	ENSMUSG00000029319	MGI:1919133	Coq2	coenzyme Q2 4-hydroxybenzoate polyprenyltransferase [Source:MGI Symbol;Acc:MGI:1919133]
5	protein_coding	1.01E+08	1.01E+08	ENSMUSG00000035273	MGI:1343124	Hpse	heparanase [Source:MGI Symbol;Acc:MGI:1343124]
5	protein_coding	1.01E+08	1.01E+08	ENSMUSG00000035266	MGI:2176740	Helq	helicase, POLQ-like [Source:MGI Symbol;Acc:MGI:2176740]
5	protein_coding	1.01E+08	1.01E+08	ENSMUSG00000016833	MGI:1915985	Mrps18c	mitochondrial ribosomal protein S18C [Source:MGI Symbol;Acc:MGI:1915985]
5	protein_coding	1.01E+08	1.01E+08	ENSMUSG00000035234	MGI:1917931	Abraxas1	BRCA1 A complex subunit [Source:MGI Symbol;Acc:MGI:1917931]
5	protein_coding	1.01E+08	1.01E+08	ENSMUSG00000029314	MGI:3603816	Gpat3	glycerol-3-phosphate acyltransferase 3 [Source:MGI Symbol;Acc:MGI:3603816]
5	protein_coding	1.02E+08	1.02E+08	ENSMUSG00000035187	MGI:1206039	Nkx6-1	NK6 homeobox 1 [Source:MGI Symbol;Acc:MGI:1206039]
5	protein_coding	1.02E+08	1.02E+08	ENSMUSG00000029330	MGI:1921846	Cds1	CDP-diacylglycerol synthase 1 [Source:MGI Symbol;Acc:MGI:1921846]
5	protein_coding	1.02E+08	1.02E+08	ENSMUSG00000043940	MGI:1096875	Wdfy3	WD repeat and FYVE domain containing 3 [Source:MGI Symbol;Acc:MGI:1096875]
5	protein_coding	1.03E+08	1.03E+08	ENSMUSG00000057315	MGI:1922647	Arhgap24	Rho GTPase activating protein 24 [Source:MGI Symbol;Acc:MGI:1922647]
5	protein_coding	1.03E+08	1.03E+08	ENSMUSG00000046709	MGI:1346863	Mapk10	mitogen-activated protein kinase 10 [Source:MGI Symbol;Acc:MGI:1346863]
5	protein_coding	1.04E+08	1.04E+08	ENSMUSG00000034573	MGI:103293	Ptpn13	protein tyrosine phosphatase, non-receptor type 13 [Source:MGI Symbol;Acc:MGI:103293]
5	protein_coding	1.04E+08	1.04E+08	ENSMUSG00000029321	MGI:1923000	Slc10a6	solute carrier family 10 (sodium/bile acid cotransporter family), member 6 [Source:MGI Symbol;Acc:MGI:1923000]
5	protein_coding	1.04E+08	1.04E+08	ENSMUSG00000029320	MGI:1921468	1700016H13Rik	RIKEN cDNA 1700016H13 gene [Source:MGI Symbol;Acc:MGI:1921468]
5	protein_coding	1.04E+08	1.04E+08	ENSMUSG00000029313	MGI:1100819	Aff1	AF4/FMR2 family, member 1 [Source:MGI Symbol;Acc:MGI:1100819]

5	protein_coding	1.04E+08	1.04E+08	ENSMUSG00000029312	MGI:2179430	Klhl8	kelch-like 8 [Source:MGI Symbol;Acc:MGI:2179430]
5	protein_coding	1.04E+08	1.04E+08	ENSMUSG00000034528	MGI:2140804	Hsd17b13	hydroxysteroid (17-beta) dehydrogenase 13 [Source:MGI Symbol;Acc:MGI:2140804]
5	protein_coding	1.04E+08	1.04E+08	ENSMUSG00000029311	MGI:2149821	Hsd17b11	hydroxysteroid (17-beta) dehydrogenase 11 [Source:MGI Symbol;Acc:MGI:2149821]
5	protein_coding	1.04E+08	1.04E+08	ENSMUSG00000029310	MGI:1921417	Nudt9	nudix hydrolase 9 [Source:MGI Symbol;Acc:MGI:1921417]
5	protein_coding	1.04E+08	1.04E+08	ENSMUSG00000091034	MGI:4937294	Scpppq1	secretory calcium-binding phosphoprotein proline-glutamine rich 1 [Source:MGI Symbol;Acc:MGI:4937294]
5	protein_coding	1.04E+08	1.04E+08	ENSMUSG00000029309	MGI:108110	Sparcl1	SPARC-like 1 [Source:MGI Symbol;Acc:MGI:108110]
5	protein_coding	1.04E+08	1.04E+08	ENSMUSG00000053268	MGI:109172	Dspp	dentin sialophosphoprotein [Source:MGI Symbol;Acc:MGI:109172]
5	protein_coding	1.04E+08	1.04E+08	ENSMUSG00000029307	MGI:94910	Dmp1	dentin matrix protein 1 [Source:MGI Symbol;Acc:MGI:94910]
5	protein_coding	1.04E+08	1.04E+08	ENSMUSG00000029306	MGI:96389	Ibsp	integrin binding sialoprotein [Source:MGI Symbol;Acc:MGI:96389]
5	protein_coding	1.04E+08	1.04E+08	ENSMUSG00000053863	MGI:2137384	Mepe	matrix extracellular phosphoglycoprotein with ASARM motif (bone) [Source:MGI Symbol;Acc:MGI:2137384]
5	protein_coding	1.05E+08	1.05E+08	ENSMUSG00000029304	MGI:98389	Spp1	secreted phosphoprotein 1 [Source:MGI Symbol;Acc:MGI:98389]
5	protein_coding	1.05E+08	1.05E+08	ENSMUSG00000034462	MGI:1099818	Pkd2	polycystin 2, transient receptor potential cation channel [Source:MGI Symbol;Acc:MGI:1099818]
5	protein_coding	1.05E+08	1.05E+08	ENSMUSG00000097392	MGI:3040669	Thoc2l	THO complex subunit 2-like [Source:MGI Symbol;Acc:MGI:3040669]
5	protein_coding	1.05E+08	1.05E+08	ENSMUSG00000120166	MGI:1916873	Zfp33b	zinc finger protein 33B [Source:MGI Symbol;Acc:MGI:1916873]
5	protein_coding	1.05E+08	1.05E+08	ENSMUSG00000072774	MGI:2441896	Zfp951	zinc finger protein 951 [Source:MGI Symbol;Acc:MGI:2441896]

5	protein_coding	1.05E+08	1.05E+08	ENSMUSG00000029299	MGI:1351624	Abcg3	ATP binding cassette subfamily G member 3 [Source:MGI Symbol;Acc:MGI:1351624]
5	protein_coding	1.05E+08	1.05E+08	ENSMUSG00000034438	MGI:1923324	Gbp8	guanylate-binding protein 8 [Source:MGI Symbol;Acc:MGI:1923324]
5	protein_coding	1.05E+08	1.05E+08	ENSMUSG00000029298	MGI:3605620	Gbp9	guanylate-binding protein 9 [Source:MGI Symbol;Acc:MGI:3605620]
5	protein_coding	1.05E+08	1.05E+08	ENSMUSG00000079363	MGI:97072	Gbp4	guanylate binding protein 4 [Source:MGI Symbol;Acc:MGI:97072]
5	protein_coding	1.05E+08	1.05E+08	ENSMUSG00000079362	MGI:5663439	Gm43302	predicted gene 43302 [Source:MGI Symbol;Acc:MGI:5663439]
5	protein_coding	1.05E+08	1.05E+08	ENSMUSG00000105096	MGI:4359647	Gbp10	guanylate-binding protein 10 [Source:MGI Symbol;Acc:MGI:4359647]
5	protein_coding	1.05E+08	1.05E+08	ENSMUSG00000104713	MGI:2140937	Gbp6	guanylate binding protein 6 [Source:MGI Symbol;Acc:MGI:2140937]
5	protein_coding	1.05E+08	1.05E+08	ENSMUSG00000092021	MGI:3646307	Gbp11	guanylate binding protein 11 [Source:MGI Symbol;Acc:MGI:3646307]
5	protein_coding	1.06E+08	1.06E+08	ENSMUSG00000070639	MGI:2141353	Lrrc8b	leucine rich repeat containing 8 family, member B [Source:MGI Symbol;Acc:MGI:2141353]
5	protein_coding	1.06E+08	1.06E+08	ENSMUSG00000054720	MGI:2140839	Lrrc8c	leucine rich repeat containing 8 family, member C [Source:MGI Symbol;Acc:MGI:2140839]
5	protein_coding	1.06E+08	1.06E+08	ENSMUSG00000046079	MGI:1922368	Lrrc8d	leucine rich repeat containing 8D [Source:MGI Symbol;Acc:MGI:1922368]
5	protein_coding	1.06E+08	1.06E+08	ENSMUSG00000029290	MGI:1927246	Zfp326	zinc finger protein 326 [Source:MGI Symbol;Acc:MGI:1927246]
5	protein_coding	1.07E+08	1.07E+08	ENSMUSG00000034384	MGI:1859314	Barhl2	BarH like homeobox 2 [Source:MGI Symbol;Acc:MGI:1859314]
5	protein_coding	1.07E+08	1.07E+08	ENSMUSG00000049606	MGI:1277212	Zfp644	zinc finger protein 644 [Source:MGI Symbol;Acc:MGI:1277212]
5	protein_coding	1.07E+08	1.07E+08	ENSMUSG00000043410	MGI:3036246	Hfm1	HFM1, ATP-dependent DNA helicase homolog [Source:MGI Symbol;Acc:MGI:3036246]
5	protein_coding	1.07E+08	1.07E+08	ENSMUSG00000029283	MGI:1309511	Cdc7	cell division cycle 7 [Source:MGI Symbol;Acc:MGI:1309511]

5	protein_coding	1.07E+08	1.07E+08	ENSMUSG00000029287	MGI:104637	Tgfbr3	transforming growth factor, beta receptor III [Source:MGI Symbol;Acc:MGI:104637]
5	protein_coding	1.07E+08	1.08E+08	ENSMUSG00000029279	MGI:1891374	Brdt	bromodomain, testis-specific [Source:MGI Symbol;Acc:MGI:1891374]
5	protein_coding	1.08E+08	1.08E+08	ENSMUSG00000033805	MGI:2686228	Ephx4	epoxide hydrolase 4 [Source:MGI Symbol;Acc:MGI:2686228]
5	protein_coding	1.08E+08	1.08E+08	ENSMUSG00000033794	MGI:1918152	Lpcat2b	lysophosphatidylcholine acyltransferase 2B [Source:MGI Symbol;Acc:MGI:1918152]
5	protein_coding	1.08E+08	1.08E+08	ENSMUSG00000111375	MGI:3646208	Btbd8	BTB domain containing 8 [Source:MGI Symbol;Acc:MGI:3646208]
5	protein_coding	1.08E+08	1.08E+08	ENSMUSG00000106631	MGI:5662806	Gm42669	predicted gene 42669 [Source:MGI Symbol;Acc:MGI:5662806]
5	protein_coding	1.08E+08	1.08E+08	ENSMUSG00000089798	MGI:1923671	1700028K03Rik	RIKEN cDNA 1700028K03 gene [Source:MGI Symbol;Acc:MGI:1923671]
5	protein_coding	1.08E+08	1.08E+08	ENSMUSG00000029276	MGI:2141180	Glmn	glomulin, FKBP associated protein [Source:MGI Symbol;Acc:MGI:2141180]
5	protein_coding	1.08E+08	1.08E+08	ENSMUSG00000033773	MGI:2141142	Rpap2	RNA polymerase II associated protein 2 [Source:MGI Symbol;Acc:MGI:2141142]
5	protein_coding	1.08E+08	1.08E+08	ENSMUSG00000029275	MGI:103170	Gfi1	growth factor independent 1 transcription repressor [Source:MGI Symbol;Acc:MGI:103170]
5	protein_coding	1.08E+08	1.08E+08	ENSMUSG00000011831	MGI:104736	Evi5	ecotropic viral integration site 5 [Source:MGI Symbol;Acc:MGI:104736]
5	protein_coding	1.08E+08	1.08E+08	ENSMUSG00000063447	MGI:1920568	Ube2d2b	ubiquitin-conjugating enzyme E2D 2B [Source:MGI Symbol;Acc:MGI:1920568]
5	protein_coding	1.08E+08	1.08E+08	ENSMUSG00000058558	MGI:102854	Rpl5	ribosomal protein L5 [Source:MGI Symbol;Acc:MGI:102854]
5	protein_coding	1.08E+08	1.08E+08	ENSMUSG00000029270	MGI:1914516	Dipk1a	divergent protein kinase domain 1A [Source:MGI Symbol;Acc:MGI:1914516]
5	protein_coding	1.08E+08	1.08E+08	ENSMUSG00000029267	MGI:105050	Mtf2	metal response element binding transcription factor 2 [Source:MGI Symbol;Acc:MGI:105050]
5	protein_coding	1.08E+08	1.08E+08	ENSMUSG00000063406	MGI:1921586	Tmed5	transmembrane p24 trafficking protein 5 [Source:MGI Symbol;Acc:MGI:1921586]

5	protein_coding	1.08E+08	1.08E+08	ENSMUSG00000056531	MGI:1922974	Ccdc18	coiled-coil domain containing 18 [Source:MGI Symbol;Acc:MGI:1922974]
5	protein_coding	1.08E+08	1.08E+08	ENSMUSG00000029265	MGI:1100515	Dr1	down-regulator of transcription 1 [Source:MGI Symbol;Acc:MGI:1100515]
5	protein_coding	1.08E+08	1.08E+08	ENSMUSG00000029263	MGI:3576484	Pigg	phosphatidylinositol glycan anchor biosynthesis, class G [Source:MGI Symbol;Acc:MGI:3576484]

Supplementary Table 5. All annotated genes within the 95% Bayesian credible interval for the pathology QTL on chromosome 5

Chr	type	start	stop	gene_id	mgi_type	gene_symbol	description
5	lncRNA	41161921	41172181	ENSMUSG00000130164	MGI:7826136	Gm71684	predicted gene, 71684 [Source:MGI Symbol;Acc:MGI:7826136]
5	lncRNA	41274860	41358312	ENSMUSG00000132413	MGI:7826738	Gm71986	predicted gene, 71986 [Source:MGI Symbol;Acc:MGI:7826738]
5	lncRNA	41350377	41406253	ENSMUSG00000132446	MGI:7826782	Gm72008	predicted gene, 72008 [Source:MGI Symbol;Acc:MGI:7826782]
5	lncRNA	41765931	41771759	ENSMUSG00000133947	MGI:5594539	Gm35380	predicted gene, 35380 [Source:MGI Symbol;Acc:MGI:5594539]
5	lncRNA	41771474	41774854	ENSMUSG00000133992	MGI:7827302	Gm72268	predicted gene, 72268 [Source:MGI Symbol;Acc:MGI:7827302]
5	lncRNA	41864464	41864958	ENSMUSG00000134029	MGI:7827314	Gm72274	predicted gene, 72274 [Source:MGI Symbol;Acc:MGI:7827314]
5	lncRNA	41865579	41893410	ENSMUSG00000133906	MGI:7827252	Gm72243	predicted gene, 72243 [Source:MGI Symbol;Acc:MGI:7827252]
5	lncRNA	42001664	42010131	ENSMUSG00000131938	MGI:7826668	Gm71951	predicted gene, 71951 [Source:MGI Symbol;Acc:MGI:7826668]
5	lncRNA	42045334	42374141	ENSMUSG00000067285	MGI:3648966	Gm16223	predicted gene 16223 [Source:MGI Symbol;Acc:MGI:3648966]
5	lncRNA	42397049	42400594	ENSMUSG00000135933	MGI:7827798	Gm72517	predicted gene, 72517 [Source:MGI Symbol;Acc:MGI:7827798]
5	lncRNA	42590654	42658143	ENSMUSG00000122969	MGI:7825344	Gm71283	predicted gene, 71283 [Source:MGI Symbol;Acc:MGI:7825344]
5	lncRNA	42693920	42733451	ENSMUSG00000142458	MGI:7829582	Gm73412	predicted gene, 73412 [Source:MGI Symbol;Acc:MGI:7829582]
5	lncRNA	42752178	42843407	ENSMUSG00000135200	MGI:6723610	Gm54295	predicted gene, 54295 [Source:MGI Symbol;Acc:MGI:6723610]
5	lncRNA	42929725	42949087	ENSMUSG00000131751	MGI:7826620	Gm71927	predicted gene, 71927 [Source:MGI Symbol;Acc:MGI:7826620]
5	lncRNA	43120883	43279559	ENSMUSG00000120713	MGI:7338483	Gm57285	predicted gene, 57285 [Source:MGI Symbol;Acc:MGI:7338483]
5	lncRNA	43183885	43195679	ENSMUSG00000107193	MGI:5663837	Gm43700	predicted gene 43700 [Source:MGI Symbol;Acc:MGI:5663837]
5	lncRNA	43197080	43201404	ENSMUSG00000106501	MGI:5662848	Gm42711	predicted gene 42711 [Source:MGI Symbol;Acc:MGI:5662848]

5	lncRNA	43206900	43217584	ENSMUSG00000125363	MGI:7825611	Gm71419	predicted gene, 71419 [Source:MGI Symbol;Acc:MGI:7825611]
5	lncRNA	43208242	43287854	ENSMUSG00000125280	MGI:7825603	Gm71415	predicted gene, 71415 [Source:MGI Symbol;Acc:MGI:7825603]
5	lncRNA	43230866	43232064	ENSMUSG00000125404	MGI:7825619	Gm71423	predicted gene, 71423 [Source:MGI Symbol;Acc:MGI:7825619]
5	lncRNA	43306203	43392804	ENSMUSG00000085720	MGI:3648234	Gm7854	predicted gene 7854 [Source:MGI Symbol;Acc:MGI:3648234]
5	lncRNA	43388856	43390471	ENSMUSG00000120663	MGI:7338471	Gm57279	predicted gene, 57279 [Source:MGI Symbol;Acc:MGI:7338471]
5	lncRNA	43454674	43461022	ENSMUSG00000137961	MGI:7828504	Gm72871	predicted gene, 72871 [Source:MGI Symbol;Acc:MGI:7828504]
5	lncRNA	43496505	43511399	ENSMUSG00000137893	MGI:7828492	Gm72865	predicted gene, 72865 [Source:MGI Symbol;Acc:MGI:7828492]
5	lncRNA	43508808	43514761	ENSMUSG00000104846	MGI:5663157	Gm43020	predicted gene 43020 [Source:MGI Symbol;Acc:MGI:5663157]
5	lncRNA	43533790	43536980	ENSMUSG00000107244	MGI:5663836	Gm43699	predicted gene 43699 [Source:MGI Symbol;Acc:MGI:5663836]
5	lncRNA	43579093	43580575	ENSMUSG00000140860	MGI:7829236	Gm73239	predicted gene, 73239 [Source:MGI Symbol;Acc:MGI:7829236]
5	lncRNA	43628035	43633199	ENSMUSG00000104691	MGI:5663158	Gm43021	predicted gene 43021 [Source:MGI Symbol;Acc:MGI:5663158]
5	lncRNA	43707271	43759073	ENSMUSG00000087290	MGI:3801874	Gm15866	predicted gene 15866 [Source:MGI Symbol;Acc:MGI:3801874]
5	lncRNA	43942993	43943494	ENSMUSG00000133185	MGI:7827010	Gm72122	predicted gene, 72122 [Source:MGI Symbol;Acc:MGI:7827010]
5	lncRNA	43965047	43966085	ENSMUSG00000104701	MGI:5662692	Gm42555	predicted gene 42555 [Source:MGI Symbol;Acc:MGI:5662692]
5	lncRNA	44018654	44021803	ENSMUSG00000133570	MGI:7827162	Gm72198	predicted gene, 72198 [Source:MGI Symbol;Acc:MGI:7827162]
5	lncRNA	44028865	44029335	ENSMUSG00000133608	MGI:7827164	Gm72199	predicted gene, 72199 [Source:MGI Symbol;Acc:MGI:7827164]
5	lncRNA	44033372	44034646	ENSMUSG00000086479	MGI:3801812	Gm16014	predicted gene 16014 [Source:MGI Symbol;Acc:MGI:3801812]
5	lncRNA	44055929	44105704	ENSMUSG00000087281	MGI:3801811	Gm16015	predicted gene 16015 [Source:MGI Symbol;Acc:MGI:3801811]

5	lncRNA	44144508	44162382	ENSMUSG00000139432	MGI:7828900	Gm73071	predicted gene, 73071 [Source:MGI Symbol;Acc:MGI:7828900]
5	lncRNA	44179180	44182741	ENSMUSG00000139558	MGI:7828932	Gm73087	predicted gene, 73087 [Source:MGI Symbol;Acc:MGI:7828932]
5	lncRNA	44225540	44248196	ENSMUSG00000105264	MGI:5662853	Gm42716	predicted gene 42716 [Source:MGI Symbol;Acc:MGI:5662853]
5	lncRNA	44257784	44265916	ENSMUSG00000072962	MGI:3648398	Gm16401	predicted gene 16401 [Source:MGI Symbol;Acc:MGI:3648398]
5	lncRNA	44263044	44271634	ENSMUSG00000139480	MGI:5595059	Gm35900	predicted gene, 35900 [Source:MGI Symbol;Acc:MGI:5595059]
5	lncRNA	44293962	44294263	ENSMUSG00000105353	MGI:5662565	Gm42428	predicted gene 42428 [Source:MGI Symbol;Acc:MGI:5662565]
5	lncRNA	44377025	44381345	ENSMUSG00000105503	MGI:5663119	Gm42982	predicted gene 42982 [Source:MGI Symbol;Acc:MGI:5663119]
5	lncRNA	44383929	44440444	ENSMUSG00000104784	MGI:5663121	Gm42984	predicted gene 42984 [Source:MGI Symbol;Acc:MGI:5663121]
5	lncRNA	44390266	44391130	ENSMUSG00000136242	MGI:7827904	Gm72570	predicted gene, 72570 [Source:MGI Symbol;Acc:MGI:7827904]
5	lncRNA	44417198	44419558	ENSMUSG00000136206	MGI:7827878	Gm72557	predicted gene, 72557 [Source:MGI Symbol;Acc:MGI:7827878]
5	lncRNA	44496607	44511899	ENSMUSG00000124218	MGI:5623181	Gm40296	predicted gene, 40296 [Source:MGI Symbol;Acc:MGI:5623181]
5	lncRNA	44570134	44589024	ENSMUSG00000104800	MGI:5595176	Gm36017	predicted gene, 36017 [Source:MGI Symbol;Acc:MGI:5595176]
5	lncRNA	44589098	44593903	ENSMUSG00000133239	MGI:5623182	Gm40297	predicted gene, 40297 [Source:MGI Symbol;Acc:MGI:5623182]
5	lncRNA	44798826	44835480	ENSMUSG00000104894	MGI:5663644	Gm43507	predicted gene 43507 [Source:MGI Symbol;Acc:MGI:5663644]
5	lncRNA	44858218	44864232	ENSMUSG00000105224	MGI:3781542	Gm3364	predicted gene 3364 [Source:MGI Symbol;Acc:MGI:3781542]
5	lncRNA	45022196	45034887	ENSMUSG00000106656	MGI:5662887	Gm42750	predicted gene 42750 [Source:MGI Symbol;Acc:MGI:5662887]
5	lncRNA	45075134	45085881	ENSMUSG00000122500	MGI:5595235	Gm36076	predicted gene, 36076 [Source:MGI Symbol;Acc:MGI:5595235]
5	lncRNA	45123525	45136147	ENSMUSG00000062496	MGI:1923033	4930431F12Rik	RIKEN cDNA 4930431F12 gene [Source:MGI Symbol;Acc:MGI:1923033]

							expressed [Source:MGI
5	lncRNA	45166513	45581744	ENSMUSG00000029092	MGI:1277236	D5Ertd615e	Symbol;Acc:MGI:1277236]
5	lncRNA	45320381	45332525	ENSMUSG00000139739	MGI:5826549	Gm46912	predicted gene, 46912 [Source:MGI Symbol;Acc:MGI:5826549]
5	lncRNA	45577426	45605217	ENSMUSG00000126761	MGI:7825753	Gm71490	predicted gene, 71490 [Source:MGI Symbol;Acc:MGI:7825753]
5	lncRNA	45591731	45607480	ENSMUSG00000105255	MGI:5648986	Gm42413	predicted gene, 42413 [Source:MGI Symbol;Acc:MGI:5648986]
5	lncRNA	45626025	45650599	ENSMUSG00000125577	MGI:7825639	Gm71433	predicted gene, 71433 [Source:MGI Symbol;Acc:MGI:7825639]
5	lncRNA	45796041	45802811	ENSMUSG00000097825	MGI:2441755	9630001P10Rik	RIKEN cDNA 9630001P10 gene [Source:MGI Symbol;Acc:MGI:2441755]
5	lncRNA	45815993	45820078	ENSMUSG00000130071	MGI:7826118	Gm71675	predicted gene, 71675 [Source:MGI Symbol;Acc:MGI:7826118]
5	lncRNA	46013440	46016107	ENSMUSG00000130050	MGI:7826114	Gm71672	predicted gene, 71672 [Source:MGI Symbol;Acc:MGI:7826114]
5	lncRNA	46084153	46089779	ENSMUSG00000106602	MGI:1925356	4930405L22Rik	RIKEN cDNA 4930405L22 gene [Source:MGI Symbol;Acc:MGI:1925356]
5	lncRNA	46505008	46572771	ENSMUSG00000140766			predicted pseudogene 7931 [Source:NCBI gene (formerly Entrezgene);Acc:100503799]
5	lncRNA	47138572	47146867	ENSMUSG00000124320	MGI:7825519	Gm71372	predicted gene, 71372 [Source:MGI Symbol;Acc:MGI:7825519]
5	lncRNA	47344395	47376820	ENSMUSG00000126799	MGI:7825755	Gm71491	predicted gene, 71491 [Source:MGI Symbol;Acc:MGI:7825755]
5	lncRNA	47547347	47549721	ENSMUSG00000133558	MGI:7827158	Gm72196	predicted gene, 72196 [Source:MGI Symbol;Acc:MGI:7827158]
5	lncRNA	47938737	48086939	ENSMUSG00000128126	MGI:7825857	Gm71542	predicted gene, 71542 [Source:MGI Symbol;Acc:MGI:7825857]
5	lncRNA	48145374	48163071	ENSMUSG00000092309	MGI:1922330	4930518C09Rik	RIKEN cDNA 4930518C09 gene [Source:MGI Symbol;Acc:MGI:1922330]
5	lncRNA	48163099	48165280	ENSMUSG00000092559	MGI:5141940	Gm20475	predicted gene 20475 [Source:MGI Symbol;Acc:MGI:5141940]
5	lncRNA	48748231	48757336	ENSMUSG00000093502	MGI:5313147	Gm20700	predicted gene 20700 [Source:MGI Symbol;Acc:MGI:5313147]
5	lncRNA	48956749	48973648	ENSMUSG00000134568	MGI:7827460	Gm72347	predicted gene, 72347 [Source:MGI Symbol;Acc:MGI:7827460]

5	lncRNA	49370218	49474233	ENSMUSG00000141583	MGI:7829400	Gm73321	predicted gene, 73321 [Source:MGI Symbol;Acc:MGI:7829400]
5	lncRNA	49474259	49532743	ENSMUSG00000134740	MGI:7827500	Gm72367	predicted gene, 72367 [Source:MGI Symbol;Acc:MGI:7827500]
5	lncRNA	49720350	49859749	ENSMUSG00000129598	MGI:7826001	Gm71615	predicted gene, 71615 [Source:MGI Symbol;Acc:MGI:7826001]
5	lncRNA	49762811	49772775	ENSMUSG00000129635	MGI:5623184	Gm40299	predicted gene, 40299 [Source:MGI Symbol;Acc:MGI:5623184]
5	lncRNA	50217951	50230629	ENSMUSG00000126135	MGI:5595861	Gm36702	predicted gene, 36702 [Source:MGI Symbol;Acc:MGI:5595861]
5	lncRNA	50308644	50365877	ENSMUSG00000104870	MGI:1921215	4930448I18Rik	RIKEN cDNA 4930448I18 gene [Source:MGI Symbol;Acc:MGI:1921215]
5	lncRNA	50426239	50675024	ENSMUSG00000126829			novel transcript
5	lncRNA	50439215	50513096	ENSMUSG00000126863	MGI:7825759	Gm71493	predicted gene, 71493 [Source:MGI Symbol;Acc:MGI:7825759]
5	lncRNA	50557102	50564135	ENSMUSG00000126932	MGI:7825771	Gm71499	predicted gene, 71499 [Source:MGI Symbol;Acc:MGI:7825771]
5	lncRNA	50606004	50701876	ENSMUSG00000105423	MGI:5595973	Gm36814	predicted gene, 36814 [Source:MGI Symbol;Acc:MGI:5595973]
5	lncRNA	50700780	50980956	ENSMUSG00000105320	MGI:5663153	Gm43016	predicted gene 43016 [Source:MGI Symbol;Acc:MGI:5663153]
5	lncRNA	50995647	50996695	ENSMUSG00000126009	MGI:7825675	Gm71451	predicted gene, 71451 [Source:MGI Symbol;Acc:MGI:7825675]
5	lncRNA	51100591	51101634	ENSMUSG00000122533	MGI:7825273	Gm71246	predicted gene, 71246 [Source:MGI Symbol;Acc:MGI:7825273]
5	lncRNA	51124509	51202427	ENSMUSG00000124531	MGI:7825539	Gm71383	predicted gene, 71383 [Source:MGI Symbol;Acc:MGI:7825539]
5	lncRNA	51435063	51446696	ENSMUSG00000138939	MGI:5589083	Gm29924	predicted gene, 29924 [Source:MGI Symbol;Acc:MGI:5589083]
5	lncRNA	51720599	51782721	ENSMUSG00000129179	MGI:7825961	Gm71594	predicted gene, 71594 [Source:MGI Symbol;Acc:MGI:7825961]
5	lncRNA	52088024	52091945	ENSMUSG00000130279	MGI:7826186	Gm71709	predicted gene, 71709 [Source:MGI Symbol;Acc:MGI:7826186]
5	lncRNA	52193982	52198110	ENSMUSG00000125968	MGI:7825671	Gm71449	predicted gene, 71449 [Source:MGI Symbol;Acc:MGI:7825671]

5	lncRNA	52269346	52270573	ENSMUSG00000133149	MGI:7826992	Gm72113	predicted gene, 72113 [Source:MGI Symbol;Acc:MGI:7826992]
5	lncRNA	52271803	52273045	ENSMUSG00000126882	MGI:7825763	Gm71495	predicted gene, 71495 [Source:MGI Symbol;Acc:MGI:7825763]
5	lncRNA	52272010	52289297	ENSMUSG00000126817	MGI:5623187	Gm40302	predicted gene, 40302 [Source:MGI Symbol;Acc:MGI:5623187]
5	lncRNA	52273209	52275868	ENSMUSG00000104847	MGI:5663314	Gm43177	predicted gene 43177 [Source:MGI Symbol;Acc:MGI:5663314]
5	lncRNA	52285971	52286585	ENSMUSG00000126918	MGI:7825767	Gm71497	predicted gene, 71497 [Source:MGI Symbol;Acc:MGI:7825767]
5	lncRNA	52347984	52496385	ENSMUSG00000097145	MGI:3041212	9230114K14Rik	RIKEN cDNA 9230114K14 gene [Source:MGI Symbol;Acc:MGI:3041212]
5	lncRNA	52348205	52349529	ENSMUSG00000124543	MGI:7825541	Gm71384	predicted gene, 71384 [Source:MGI Symbol;Acc:MGI:7825541]
5	lncRNA	52357326	52373776	ENSMUSG00000106464	MGI:3041216	C130083M11Rik	RIKEN cDNA C130083M11 gene [Source:MGI Symbol;Acc:MGI:3041216]
5	lncRNA	52499428	52499702	ENSMUSG00000104751	MGI:5663316	Gm43179	predicted gene 43179 [Source:MGI Symbol;Acc:MGI:5663316]
5	lncRNA	52512028	52513002	ENSMUSG00000124928	MGI:7825579	Gm71403	predicted gene, 71403 [Source:MGI Symbol;Acc:MGI:7825579]
5	lncRNA	52598143	52606135	ENSMUSG00000106380	MGI:3781696	Gm3519	predicted gene 3519 [Source:MGI Symbol;Acc:MGI:3781696]
5	lncRNA	52633807	52639338	ENSMUSG00000143439	MGI:7829810	Gm73527	predicted gene, 73527 [Source:MGI Symbol;Acc:MGI:7829810]
5	lncRNA	52696173	52697421	ENSMUSG00000104829	MGI:5663822	Gm43685	predicted gene 43685 [Source:MGI Symbol;Acc:MGI:5663822]
5	lncRNA	52744234	52776414	ENSMUSG00000052295	MGI:2443206	8030423F21Rik	RIKEN cDNA 8030423F21 gene [Source:MGI Symbol;Acc:MGI:2443206]
5	lncRNA	52744861	52748627	ENSMUSG00000122870	MGI:7825334	Gm71278	predicted gene, 71278 [Source:MGI Symbol;Acc:MGI:7825334]
5	lncRNA	52864538	52870164	ENSMUSG00000127317	MGI:7825803	Gm71515	predicted gene, 71515 [Source:MGI Symbol;Acc:MGI:7825803]
5	lncRNA	52898657	52899604	ENSMUSG00000138208	MGI:7828538	Gm72888	predicted gene, 72888 [Source:MGI Symbol;Acc:MGI:7828538]
5	lncRNA	53041447	53044211	ENSMUSG00000125532	MGI:5589400	Gm30241	predicted gene, 30241 [Source:MGI Symbol;Acc:MGI:5589400]

5	lncRNA	53067635	53187870	ENSMUSG00000106538	MGI:5589460	Gm30301	predicted gene, 30301 [Source:MGI Symbol;Acc:MGI:5589460]
5	lncRNA	53080694	53081256	ENSMUSG00000122780	MGI:7825307	Gm71263	predicted gene, 71263 [Source:MGI Symbol;Acc:MGI:7825307]
5	lncRNA	53130322	53157698	ENSMUSG00000106352	MGI:1923204	5033403H07Rik	RIKEN cDNA 5033403H07 gene [Source:MGI Symbol;Acc:MGI:1923204]
5	lncRNA	53205029	53206530	ENSMUSG00000090642	MGI:4938009	Gm17182	predicted gene 17182 [Source:MGI Symbol;Acc:MGI:4938009]
5	lncRNA	53369449	53395987	ENSMUSG00000128101	MGI:5589623	Gm30464	predicted gene, 30464 [Source:MGI Symbol;Acc:MGI:5589623]
5	lncRNA	53396880	53404254	ENSMUSG00000124368	MGI:5589678	Gm30519	predicted gene, 30519 [Source:MGI Symbol;Acc:MGI:5589678]
5	lncRNA	53421512	53424670	ENSMUSG00000142257	MGI:5623188	Gm40303	predicted gene, 40303 [Source:MGI Symbol;Acc:MGI:5623188]
5	lncRNA	53439152	53440851	ENSMUSG00000106981	MGI:5791331	Gm45495	predicted gene 45495 [Source:MGI Symbol;Acc:MGI:5791331]
5	lncRNA	53517669	53529118	ENSMUSG00000106808	MGI:5662882	Gm42745	predicted gene 42745 [Source:MGI Symbol;Acc:MGI:5662882]
5	lncRNA	53556413	53561612	ENSMUSG00000133208	MGI:7827040	Gm72137	predicted gene, 72137 [Source:MGI Symbol;Acc:MGI:7827040]
5	lncRNA	53621108	53626890	ENSMUSG00000125364	MGI:7825613	Gm71420	predicted gene, 71420 [Source:MGI Symbol;Acc:MGI:7825613]
5	lncRNA	53627024	53627431	ENSMUSG00000125448	MGI:7825621	Gm71424	predicted gene, 71424 [Source:MGI Symbol;Acc:MGI:7825621]
5	lncRNA	53676602	53680073	ENSMUSG00000106807	MGI:3641801	Gm10441	predicted gene 10441 [Source:MGI Symbol;Acc:MGI:3641801]
5	lncRNA	53703517	53704753	ENSMUSG00000107111	MGI:5623189	Gm40304	predicted gene, 40304 [Source:MGI Symbol;Acc:MGI:5623189]
5	lncRNA	53828492	53828778	ENSMUSG00000130376	MGI:7826198	Gm71715	predicted gene, 71715 [Source:MGI Symbol;Acc:MGI:7826198]
5	lncRNA	54081392	54087398	ENSMUSG00000132695	MGI:7826824	Gm72029	predicted gene, 72029 [Source:MGI Symbol;Acc:MGI:7826824]
5	lncRNA	54134035	54153958	ENSMUSG00000126689	MGI:5589760	Gm30601	predicted gene, 30601 [Source:MGI Symbol;Acc:MGI:5589760]
5	lncRNA	54302971	54305114	ENSMUSG00000122231	MGI:7825241	Gm71230	predicted gene, 71230 [Source:MGI Symbol;Acc:MGI:7825241]

5	lncRNA	54424223	54671511	ENSMUSG00000105912	MGI:3641803	Gm10440	predicted gene 10440 [Source:MGI Symbol;Acc:MGI:3641803]
5	lncRNA	54521779	54580819	ENSMUSG00000134062	MGI:7827324	Gm72279	predicted gene, 72279 [Source:MGI Symbol;Acc:MGI:7827324]
5	lncRNA	54586049	54598834	ENSMUSG00000134107	MGI:7827366	Gm72300	predicted gene, 72300 [Source:MGI Symbol;Acc:MGI:7827366]
5	lncRNA	54598112	54603067	ENSMUSG00000134141	MGI:7827386	Gm72310	predicted gene, 72310 [Source:MGI Symbol;Acc:MGI:7827386]
5	lncRNA	56169492	56177088	ENSMUSG00000126211	MGI:7825687	Gm71457	predicted gene, 71457 [Source:MGI Symbol;Acc:MGI:7825687]
5	lncRNA	57316575	57317567	ENSMUSG00000138900	MGI:7828752	Gm72996	predicted gene, 72996 [Source:MGI Symbol;Acc:MGI:7828752]
5	lncRNA	57727421	57879729	ENSMUSG00000097216	MGI:2441691	4932441J04Rik	RIKEN cDNA 4932441J04 gene [Source:MGI Symbol;Acc:MGI:2441691]
5	lncRNA	57872321	57873985	ENSMUSG00000140036	MGI:7829030	Gm73136	predicted gene, 73136 [Source:MGI Symbol;Acc:MGI:7829030]
5	lncRNA	58205960	58217711	ENSMUSG00000137396	MGI:7828378	Gm72808	predicted gene, 72808 [Source:MGI Symbol;Acc:MGI:7828378]
5	lncRNA	58383961	58428446	ENSMUSG00000124009	MGI:7825475	Gm71350	predicted gene, 71350 [Source:MGI Symbol;Acc:MGI:7825475]
5	lncRNA	58600579	58610346	ENSMUSG00000105234	MGI:1925413	4930459L07Rik	RIKEN cDNA 4930459L07 gene [Source:MGI Symbol;Acc:MGI:1925413]
5	lncRNA	58610360	58612683	ENSMUSG00000100557	MGI:1919500	1700029E06Rik	RIKEN cDNA 1700029E06 gene [Source:MGI Symbol;Acc:MGI:1919500]
5	lncRNA	58686983	58690013	ENSMUSG00000136713	MGI:7828114	Gm72676	predicted gene, 72676 [Source:MGI Symbol;Acc:MGI:7828114]
5	lncRNA	58735034	58847926	ENSMUSG00000133497	MGI:7827146	Gm72190	predicted gene, 72190 [Source:MGI Symbol;Acc:MGI:7827146]
5	lncRNA	58985311	59042383	ENSMUSG00000129218	MGI:7825963	Gm71595	predicted gene, 71595 [Source:MGI Symbol;Acc:MGI:7825963]
5	lncRNA	59727045	59731585	ENSMUSG00000106634	MGI:5663179	Gm43042	predicted gene 43042 [Source:MGI Symbol;Acc:MGI:5663179]
5	lncRNA	59870661	59901815	ENSMUSG00000138074	MGI:7828518	Gm72878	predicted gene, 72878 [Source:MGI Symbol;Acc:MGI:7828518]
5	lncRNA	60106402	60113597	ENSMUSG00000123790	MGI:7825443	Gm71333	predicted gene, 71333 [Source:MGI Symbol;Acc:MGI:7825443]

5	lncRNA	60116730	60143384	ENSMUSG00000106013	MGI:1918307	4933402J10Rik	RIKEN cDNA 4933402J10 gene [Source:MGI Symbol;Acc:MGI:1918307]
5	lncRNA	60388621	60389202	ENSMUSG00000129251	MGI:7825967	Gm71597	predicted gene, 71597 [Source:MGI Symbol;Acc:MGI:7825967]
5	lncRNA	60468348	60471120	ENSMUSG00000135740	MGI:7827770	Gm72503	predicted gene, 72503 [Source:MGI Symbol;Acc:MGI:7827770]
5	lncRNA	60543093	60560807	ENSMUSG00000136235	MGI:7827898	Gm72567	predicted gene, 72567 [Source:MGI Symbol;Acc:MGI:7827898]
5	lncRNA	61098281	61152246	ENSMUSG00000105903	MGI:1924172	1700014F14Rik	RIKEN cDNA 1700014F14 gene [Source:MGI Symbol;Acc:MGI:1924172]
5	lncRNA	61187951	61196673	ENSMUSG00000133805	MGI:7827228	Gm72231	predicted gene, 72231 [Source:MGI Symbol;Acc:MGI:7827228]
5	lncRNA	61520600	61531385	ENSMUSG00000134597	MGI:7827468	Gm72351	predicted gene, 72351 [Source:MGI Symbol;Acc:MGI:7827468]
5	lncRNA	61722400	61734622	ENSMUSG00000143250	MGI:7829752	Gm73498	predicted gene, 73498 [Source:MGI Symbol;Acc:MGI:7829752]
5	lncRNA	61846082	61849804	ENSMUSG00000124549	MGI:7825545	Gm71386	predicted gene, 71386 [Source:MGI Symbol;Acc:MGI:7825545]
5	lncRNA	61921477	61966679	ENSMUSG00000143342	MGI:5623193	Gm40308	predicted gene, 40308 [Source:MGI Symbol;Acc:MGI:5623193]
5	lncRNA	61964088	61964533	ENSMUSG00000143380	MGI:7829796	Gm73520	predicted gene, 73520 [Source:MGI Symbol;Acc:MGI:7829796]
5	lncRNA	62207719	62231006	ENSMUSG00000138711	MGI:7828672	Gm72956	predicted gene, 72956 [Source:MGI Symbol;Acc:MGI:7828672]
5	lncRNA	62456170	62456705	ENSMUSG00000128227	MGI:7825863	Gm71545	predicted gene, 71545 [Source:MGI Symbol;Acc:MGI:7825863]
5	lncRNA	62711691	62745660	ENSMUSG00000122017	MGI:7825185	Gm71202	predicted gene, 71202 [Source:MGI Symbol;Acc:MGI:7825185]
5	lncRNA	63008684	63025265	ENSMUSG00000124789	MGI:7825563	Gm71395	predicted gene, 71395 [Source:MGI Symbol;Acc:MGI:7825563]
5	lncRNA	63718561	63781615	ENSMUSG00000134846	MGI:5589996	Gm30837	predicted gene, 30837 [Source:MGI Symbol;Acc:MGI:5589996]
5	lncRNA	63960917	63961635	ENSMUSG00000132514	MGI:7826796	Gm72015	predicted gene, 72015 [Source:MGI Symbol;Acc:MGI:7826796]
5	lncRNA	64081244	64082313	ENSMUSG00000087552	MGI:3802175	Gm15819	predicted gene 15819 [Source:MGI Symbol;Acc:MGI:3802175]

5	lncRNA	64187388	64200435	ENSMUSG00000127532	MGI:7825817	Gm71522	predicted gene, 71522 [Source:MGI Symbol;Acc:MGI:7825817]
5	lncRNA	64202316	64215554	ENSMUSG00000106219	MGI:1922007	5830416I19Rik	RIKEN cDNA 5830416I19 gene [Source:MGI Symbol;Acc:MGI:1922007]
5	lncRNA	64306914	64310169	ENSMUSG00000123942	MGI:7825463	Gm71344	predicted gene, 71344 [Source:MGI Symbol;Acc:MGI:7825463]
5	lncRNA	64388000	64388359	ENSMUSG00000132563	MGI:7826802	Gm72018	predicted gene, 72018 [Source:MGI Symbol;Acc:MGI:7826802]
5	lncRNA	64480644	64521876	ENSMUSG00000142621	MGI:7829632	Gm73438	predicted gene, 73438 [Source:MGI Symbol;Acc:MGI:7829632]
5	lncRNA	64500293	64501895	ENSMUSG00000142836	MGI:7829676	Gm73460	predicted gene, 73460 [Source:MGI Symbol;Acc:MGI:7829676]
5	lncRNA	64517664	64665195	ENSMUSG00000105730	MGI:5663975	Gm43838	predicted gene 43838 [Source:MGI Symbol;Acc:MGI:5663975]
5	lncRNA	64531911	64535171	ENSMUSG00000142793	MGI:7829666	Gm73455	predicted gene, 73455 [Source:MGI Symbol;Acc:MGI:7829666]
5	lncRNA	64612029	64630076	ENSMUSG00000100911	MGI:1917249	1700027F09Rik	RIKEN cDNA 1700027F09 gene [Source:MGI Symbol;Acc:MGI:1917249]
5	lncRNA	64635541	64645120	ENSMUSG00000142709	MGI:7829662	Gm73453	predicted gene, 73453 [Source:MGI Symbol;Acc:MGI:7829662]
5	lncRNA	64673246	64673829	ENSMUSG00000129223	MGI:7825965	Gm71596	predicted gene, 71596 [Source:MGI Symbol;Acc:MGI:7825965]
5	lncRNA	64692554	64695172	ENSMUSG00000123933	MGI:5590582	Gm31423	predicted gene, 31423 [Source:MGI Symbol;Acc:MGI:5590582]
5	lncRNA	64751135	64768095	ENSMUSG00000105402	MGI:3781892	Gm3716	predicted gene 3716 [Source:MGI Symbol;Acc:MGI:3781892]
5	lncRNA	64752661	64763260	ENSMUSG00000124498	MGI:7825535	Gm71381	predicted gene, 71381 [Source:MGI Symbol;Acc:MGI:7825535]
5	lncRNA	64790847	64961791	ENSMUSG00000097640	MGI:5012218	Gm20033	predicted gene, 20033 [Source:MGI Symbol;Acc:MGI:5012218]
5	lncRNA	64792704	64794731	ENSMUSG00000105183	MGI:5662701	Gm42564	predicted gene 42564 [Source:MGI Symbol;Acc:MGI:5662701]
5	lncRNA	64793183	64794959	ENSMUSG00000104928	MGI:5662702	Gm42565	predicted gene 42565 [Source:MGI Symbol;Acc:MGI:5662702]
5	lncRNA	64845044	64853943	ENSMUSG00000136537	MGI:7828016	Gm72627	predicted gene, 72627 [Source:MGI Symbol;Acc:MGI:7828016]

5	lncRNA	64850760	64852105	ENSMUSG00000136771	MGI:7828162	Gm72700	predicted gene, 72700 [Source:MGI Symbol;Acc:MGI:7828162]
5	lncRNA	64852804	64855931	ENSMUSG00000097668	MGI:5477255	Gm26761	predicted gene, 26761 [Source:MGI Symbol;Acc:MGI:5477255]
5	lncRNA	64872245	64880507	ENSMUSG00000136576	MGI:7828018	Gm72628	predicted gene, 72628 [Source:MGI Symbol;Acc:MGI:7828018]
5	lncRNA	64898790	64905510	ENSMUSG00000136618	MGI:7828020	Gm72629	predicted gene, 72629 [Source:MGI Symbol;Acc:MGI:7828020]
5	lncRNA	64905617	64912317	ENSMUSG00000136650	MGI:7828080	Gm72659	predicted gene, 72659 [Source:MGI Symbol;Acc:MGI:7828080]
5	lncRNA	64955290	64959712	ENSMUSG00000136685	MGI:7828098	Gm72668	predicted gene, 72668 [Source:MGI Symbol;Acc:MGI:7828098]
5	lncRNA	65006058	65022788	ENSMUSG00000129045	MGI:7825951	Gm71589	predicted gene, 71589 [Source:MGI Symbol;Acc:MGI:7825951]
5	lncRNA	65036877	65046210	ENSMUSG00000126366	MGI:5591048	Gm31889	predicted gene, 31889 [Source:MGI Symbol;Acc:MGI:5591048]
5	lncRNA	65055091	65063183	ENSMUSG00000123189	MGI:7830556	Gm73906	predicted gene, 73906 [Source:MGI Symbol;Acc:MGI:7830556]
5	lncRNA	65056480	65060908	ENSMUSG00000123224	MGI:7825377	Gm71300	predicted gene, 71300 [Source:MGI Symbol;Acc:MGI:7825377]
5	lncRNA	65091094	65093321	ENSMUSG00000140599	MGI:7829202	Gm73222	predicted gene, 73222 [Source:MGI Symbol;Acc:MGI:7829202]
5	lncRNA	65117478	65126843	ENSMUSG00000139765	MGI:7828954	Gm73098	predicted gene, 73098 [Source:MGI Symbol;Acc:MGI:7828954]
5	lncRNA	65205039	65208797	ENSMUSG00000141992	MGI:7829484	Gm73363	predicted gene, 73363 [Source:MGI Symbol;Acc:MGI:7829484]
5	lncRNA	65215935	65229709	ENSMUSG00000125845	MGI:7825653	Gm71440	predicted gene, 71440 [Source:MGI Symbol;Acc:MGI:7825653]
5	lncRNA	65260538	65264934	ENSMUSG00000141465	MGI:7829390	Gm73316	predicted gene, 73316 [Source:MGI Symbol;Acc:MGI:7829390]
5	lncRNA	65493030	65503925	ENSMUSG00000127609	MGI:7825827	Gm71527	predicted gene, 71527 [Source:MGI Symbol;Acc:MGI:7825827]
5	lncRNA	65745059	65748783	ENSMUSG00000107102	MGI:5662863	Gm42726	predicted gene 42726 [Source:MGI Symbol;Acc:MGI:5662863]
5	lncRNA	65917839	65921732	ENSMUSG00000106978	MGI:1921484	N4bp2os	NEDD4 binding protein 2, opposite strand [Source:MGI Symbol;Acc:MGI:1921484]

5	lncRNA	65922212	65922830	ENSMUSG00000137008	MGI:7828262	Gm72750	predicted gene, 72750 [Source:MGI Symbol;Acc:MGI:7828262]
5	lncRNA	65960105	66015649	ENSMUSG00000135056	MGI:3781698	Gm3521	predicted gene 3521 [Source:MGI Symbol;Acc:MGI:3781698]
5	lncRNA	66008368	66008774	ENSMUSG00000135096	MGI:7827602	Gm72419	predicted gene, 72419 [Source:MGI Symbol;Acc:MGI:7827602]
5	lncRNA	66024584	66028133	ENSMUSG00000137580	MGI:7828448	Gm72843	predicted gene, 72843 [Source:MGI Symbol;Acc:MGI:7828448]
5	lncRNA	66041281	66070097	ENSMUSG00000120171	MGI:5591633	Gm32474	predicted gene, 32474 [Source:MGI Symbol;Acc:MGI:5591633]
5	lncRNA	66060763	66073081	ENSMUSG00000120641	MGI:7338467	Gm57277	predicted gene, 57277 [Source:MGI Symbol;Acc:MGI:7338467]
5	lncRNA	66072341	66077025	ENSMUSG00000137541	MGI:5591715	Gm32556	predicted gene, 32556 [Source:MGI Symbol;Acc:MGI:5591715]
5	lncRNA	66086181	66086780	ENSMUSG00000120547	MGI:7338447	Gm57267	predicted gene, 57267 [Source:MGI Symbol;Acc:MGI:7338447]
5	lncRNA	66101760	66113567	ENSMUSG00000122150	MGI:7825215	Gm71217	predicted gene, 71217 [Source:MGI Symbol;Acc:MGI:7825215]
5	lncRNA	66108660	66109554	ENSMUSG00000122193	MGI:7825231	Gm71225	predicted gene, 71225 [Source:MGI Symbol;Acc:MGI:7825231]
5	lncRNA	66136701	66161628	ENSMUSG00000054598	MGI:3041166	9130230L23Rik	RIKEN cDNA 9130230L23 gene [Source:MGI Symbol;Acc:MGI:3041166]
5	lncRNA	66226398	66227339	ENSMUSG00000132593	MGI:7826806	Gm72020	predicted gene, 72020 [Source:MGI Symbol;Acc:MGI:7826806]
5	lncRNA	66234224	66235217	ENSMUSG00000140414	MGI:7829146	Gm73194	predicted gene, 73194 [Source:MGI Symbol;Acc:MGI:7829146]
5	lncRNA	66308366	66345219	ENSMUSG00000097092	MGI:5477219	Gm26725	predicted gene, 26725 [Source:MGI Symbol;Acc:MGI:5477219]
5	lncRNA	66323950	66348679	ENSMUSG00000107014	MGI:1920889	1700126H18Rik	RIKEN cDNA 1700126H18 gene [Source:MGI Symbol;Acc:MGI:1920889]
5	lncRNA	66332380	66333301	ENSMUSG00000133276	MGI:7827094	Gm72164	predicted gene, 72164 [Source:MGI Symbol;Acc:MGI:7827094]
5	lncRNA	66371551	66371888	ENSMUSG00000142467	MGI:7829588	Gm73415	predicted gene, 73415 [Source:MGI Symbol;Acc:MGI:7829588]
5	lncRNA	66371969	66373272	ENSMUSG00000141032	MGI:7829314	Gm73278	predicted gene, 73278 [Source:MGI Symbol;Acc:MGI:7829314]

5	lncRNA	66480691	66492259	ENSMUSG00000143772	MGI:7829966	Gm73605	predicted gene, 73605 [Source:MGI Symbol;Acc:MGI:7829966]
5	lncRNA	66499835	66502882	ENSMUSG00000143812	MGI:7829990	Gm73617	predicted gene, 73617 [Source:MGI Symbol;Acc:MGI:7829990]
5	lncRNA	66563177	66564984	ENSMUSG00000143854	MGI:7830012	Gm73628	predicted gene, 73628 [Source:MGI Symbol;Acc:MGI:7830012]
5	lncRNA	66783832	66833841	ENSMUSG00000087601	MGI:5446771	Uchl1os	[illegible] opposite strand [Source:MGI Symbol;Acc:MGI:5446771]
5	lncRNA	66844671	66847320	ENSMUSG00000143516	MGI:7829870	Gm73557	predicted gene, 73557 [Source:MGI Symbol;Acc:MGI:7829870]
5	lncRNA	66874038	66877305	ENSMUSG00000140006	MGI:1918405	4933424C09Rik	RIKEN cDNA 4933424C09 gene [Source:MGI Symbol;Acc:MGI:1918405]
5	lncRNA	67001309	67002854	ENSMUSG00000124373	MGI:7825527	Gm71377	predicted gene, 71377 [Source:MGI Symbol;Acc:MGI:7825527]
5	lncRNA	67044882	67051495	ENSMUSG00000124297	MGI:7825513	Gm71369	predicted gene, 71369 [Source:MGI Symbol;Acc:MGI:7825513]
5	lncRNA	67063518	67067487	ENSMUSG00000124335	MGI:7825523	Gm71374	predicted gene, 71374 [Source:MGI Symbol;Acc:MGI:7825523]
5	lncRNA	67142434	67142984	ENSMUSG00000085839	MGI:3801730	Gm15949	predicted gene 15949 [Source:MGI Symbol;Acc:MGI:3801730]
5	lncRNA	67146159	67148550	ENSMUSG00000086964	MGI:3801731	Gm15948	predicted gene 15948 [Source:MGI Symbol;Acc:MGI:3801731]
5	lncRNA	67256018	67287479	ENSMUSG00000122852			predicted gene, 33167 [Source:NCBI gene (formerly Entrezgene);Acc:102635966]
5	lncRNA	67378208	67389306	ENSMUSG00000120155	MGI:5592452	Gm33293	predicted gene, 33293 [Source:MGI Symbol;Acc:MGI:5592452]
5	lncRNA	67401114	67418605	ENSMUSG00000107319	MGI:5662807	Gm42670	predicted gene 42670 [Source:MGI Symbol;Acc:MGI:5662807]
5	lncRNA	67517660	67523998	ENSMUSG00000107266	MGI:5663835	Gm43698	predicted gene 43698 [Source:MGI Symbol;Acc:MGI:5663835]
5	lncRNA	67585021	67595333	ENSMUSG00000097840	MGI:5477250	Gm26756	predicted gene, 26756 [Source:MGI Symbol;Acc:MGI:5477250]
5	lncRNA	67601535	67602073	ENSMUSG00000135689	MGI:7827762	Gm72499	predicted gene, 72499 [Source:MGI Symbol;Acc:MGI:7827762]
5	lncRNA	67601952	67614849	ENSMUSG00000135654			novel transcript

5	lncRNA	67621240	67685733	ENSMUSG00000107385	MGI:2444116	C330024D21Rik	RIKEN cDNA C330024D21 gene [Source:MGI Symbol;Acc:MGI:2444116]
5	lncRNA	67663123	67699286	ENSMUSG00000126656	MGI:7825739	Gm71483	predicted gene, 71483 [Source:MGI Symbol;Acc:MGI:7825739]
5	lncRNA	67764028	67765858	ENSMUSG00000139038	MGI:7828790	Gm73015	predicted gene, 73015 [Source:MGI Symbol;Acc:MGI:7828790]
5	lncRNA	67799744	67804119	ENSMUSG00000085363	MGI:3705145	Gm15478	predicted gene 15478 [Source:MGI Symbol;Acc:MGI:3705145]
5	lncRNA	67989308	67991617	ENSMUSG00000138756	MGI:7828690	Gm72965	predicted gene, 72965 [Source:MGI Symbol;Acc:MGI:7828690]
5	lncRNA	68004408	68134426	ENSMUSG00000087261	MGI:3705209	Gm15477	predicted gene 15477 [Source:MGI Symbol;Acc:MGI:3705209]
5	lncRNA	68090947	68100493	ENSMUSG00000106880	MGI:1921115	4930425K10Rik	RIKEN cDNA 4930425K10 gene [Source:MGI Symbol;Acc:MGI:1921115]
5	lncRNA	68833488	68862977	ENSMUSG00000125658	MGI:7825641	Gm71434	predicted gene, 71434 [Source:MGI Symbol;Acc:MGI:7825641]
5	lncRNA	69163318	69165913	ENSMUSG00000128313	MGI:7825879	Gm71553	predicted gene, 71553 [Source:MGI Symbol;Acc:MGI:7825879]
5	lncRNA	69234412	69351194	ENSMUSG00000129031	MGI:7825949	Gm71588	predicted gene, 71588 [Source:MGI Symbol;Acc:MGI:7825949]
5	lncRNA	69870331	69879091	ENSMUSG00000122868	MGI:7825332	Gm71277	predicted gene, 71277 [Source:MGI Symbol;Acc:MGI:7825332]
5	lncRNA	69983472	69984189	ENSMUSG00000128577	MGI:7825921	Gm71574	predicted gene, 71574 [Source:MGI Symbol;Acc:MGI:7825921]
5	lncRNA	70773071	70813819	ENSMUSG00000141687	MGI:7829420	Gm73331	predicted gene, 73331 [Source:MGI Symbol;Acc:MGI:7829420]
5	lncRNA	71253257	71272467	ENSMUSG00000144038	MGI:7830100	Gm73674	predicted gene, 73674 [Source:MGI Symbol;Acc:MGI:7830100]
5	lncRNA	71837607	71858841	ENSMUSG00000105489	MGI:5621447	Gm38562	predicted gene, 38562 [Source:MGI Symbol;Acc:MGI:5621447]
5	lncRNA	72180652	72188828	ENSMUSG00000139447	MGI:7828904	Gm73073	predicted gene, 73073 [Source:MGI Symbol;Acc:MGI:7828904]
5	lncRNA	72188978	72189764	ENSMUSG00000139489	MGI:7828910	Gm73076	predicted gene, 73076 [Source:MGI Symbol;Acc:MGI:7828910]
5	lncRNA	72402768	72419443	ENSMUSG00000142102	MGI:7829496	Gm73369	predicted gene, 73369 [Source:MGI Symbol;Acc:MGI:7829496]

5	lncRNA	72412097	72422093	ENSMUSG00000142140	MGI:7829520	Gm73381	predicted gene, 73381 [Source:MGI Symbol;Acc:MGI:7829520]
5	lncRNA	72473671	72525391	ENSMUSG00000093400	MGI:5313094	Gm20647	predicted gene 20647 [Source:MGI Symbol;Acc:MGI:5313094]
5	lncRNA	72653543	72654447	ENSMUSG00000093771	MGI:3034643	AU023070	expressed sequence AU023070 [Source:MGI Symbol;Acc:MGI:3034643]
5	lncRNA	72660812	72663757	ENSMUSG00000141448	MGI:7829388	Gm73315	predicted gene, 73315 [Source:MGI Symbol;Acc:MGI:7829388]
5	lncRNA	72792930	72793556	ENSMUSG00000143901	MGI:7830026	Gm73636	predicted gene, 73636 [Source:MGI Symbol;Acc:MGI:7830026]
5	lncRNA	72801750	72804483	ENSMUSG00000106136	MGI:1921334	4933408A14Rik	RIKEN cDNA 4933408A14 gene [Source:MGI Symbol;Acc:MGI:1921334]
5	lncRNA	72817429	72836966	ENSMUSG00000135140	MGI:7827610	Gm72423	predicted gene, 72423 [Source:MGI Symbol;Acc:MGI:7827610]
5	lncRNA	72837080	72849347	ENSMUSG00000139316	MGI:7828836	Gm73039	predicted gene, 73039 [Source:MGI Symbol;Acc:MGI:7828836]
5	lncRNA	73064588	73066196	ENSMUSG00000141163	MGI:7829348	Gm73295	predicted gene, 73295 [Source:MGI Symbol;Acc:MGI:7829348]
5	lncRNA	73070894	73071564	ENSMUSG00000132452	MGI:7826788	Gm72011	predicted gene, 72011 [Source:MGI Symbol;Acc:MGI:7826788]
5	lncRNA	73171243	73190770	ENSMUSG00000142109			novel transcript, antisense to Fryl
5	lncRNA	73314344	73315233	ENSMUSG00000086376	MGI:1920735	1700071G01Rik	RIKEN cDNA 1700071G01 gene [Source:MGI Symbol;Acc:MGI:1920735]
5	lncRNA	73367499	73369986	ENSMUSG00000090561	MGI:4938034	Gm17207	predicted gene 17207 [Source:MGI Symbol;Acc:MGI:4938034]
5	lncRNA	73388934	73392244	ENSMUSG00000136166	MGI:7827846	Gm72541	predicted gene, 72541 [Source:MGI Symbol;Acc:MGI:7827846]
5	lncRNA	73413556	73436481	ENSMUSG00000107087	MGI:5593570	Gm34411	predicted gene, 34411 [Source:MGI Symbol;Acc:MGI:5593570]
5	lncRNA	73425716	73449924	ENSMUSG00000100175	MGI:1919526	1700025M24Rik	RIKEN cDNA 1700025M24 gene [Source:MGI Symbol;Acc:MGI:1919526]
5	lncRNA	73439298	73441107	ENSMUSG00000136196	MGI:7827874	Gm72555	predicted gene, 72555 [Source:MGI Symbol;Acc:MGI:7827874]
5	lncRNA	73441541	73445986	ENSMUSG00000136125	MGI:5623201	Gm40316	predicted gene, 40316 [Source:MGI Symbol;Acc:MGI:5623201]

5	lncRNA	73506014	73525734	ENSMUSG00000125783	MGI:5593655	Gm34496	predicted gene, 34496 [Source:MGI Symbol;Acc:MGI:5593655]
5	lncRNA	73535307	73550115	ENSMUSG00000121792	MGI:7828490	Gm72864	predicted gene, 72864 [Source:MGI Symbol;Acc:MGI:7828490]
5	lncRNA	73633700	73638944	ENSMUSG00000137355	MGI:7830260	Gm73755	predicted gene, 73755 [Source:MGI Symbol;Acc:MGI:7830260]
5	lncRNA	73731812	73734265	ENSMUSG00000139088	MGI:7828794	Gm73017	predicted gene, 73017 [Source:MGI Symbol;Acc:MGI:7828794]
5	lncRNA	73806349	73809172	ENSMUSG00000137084	MGI:5594061	Gm34902	predicted gene, 34902 [Source:MGI Symbol;Acc:MGI:5594061]
5	lncRNA	73955125	73962043	ENSMUSG00000126365	MGI:5594119	Gm34960	predicted gene, 34960 [Source:MGI Symbol;Acc:MGI:5594119]
5	lncRNA	74060149	74072798	ENSMUSG00000129782	MGI:7826029	Gm71629	predicted gene, 71629 [Source:MGI Symbol;Acc:MGI:7826029]
5	lncRNA	74070190	74077770	ENSMUSG00000107027	MGI:1916652	1700019F05Rik	RIKEN cDNA 1700019F05 gene [Source:MGI Symbol;Acc:MGI:1916652]
5	lncRNA	74090336	74112514	ENSMUSG00000143520	MGI:7829874	Gm73559	predicted gene, 73559 [Source:MGI Symbol;Acc:MGI:7829874]
5	lncRNA	74102628	74106682	ENSMUSG00000106968	MGI:2141303	C78283	expressed sequence C78283 [Source:MGI Symbol;Acc:MGI:2141303]
5	lncRNA	74174805	74200657	ENSMUSG00000121985			novel transcript
5	lncRNA	74174859	74190341	ENSMUSG00000087250	MGI:1921549	Usp46os1	ubiquitin specific peptidase 46, opposite strand 1 [Source:MGI Symbol;Acc:MGI:1921549]
5	lncRNA	74192929	74200664	ENSMUSG00000086234	MGI:1924246	Usp46os2	ubiquitin specific peptidase 46, opposite strand 2 [Source:MGI Symbol;Acc:MGI:1924246]
5	lncRNA	74241653	74254064	ENSMUSG00000128868	MGI:7825935	Gm71581	predicted gene, 71581 [Source:MGI Symbol;Acc:MGI:7825935]
5	lncRNA	74253357	74284805	ENSMUSG00000106943	MGI:1917286	Dancr	differentiation antagonizing non-protein coding RNA [Source:MGI Symbol;Acc:MGI:1917286]
5	lncRNA	74272040	74279270	ENSMUSG00000128908	MGI:7825939	Gm71583	predicted gene, 71583 [Source:MGI Symbol;Acc:MGI:7825939]
5	lncRNA	74303305	74319238	ENSMUSG00000122053	MGI:7825203	Gm71211	predicted gene, 71211 [Source:MGI Symbol;Acc:MGI:7825203]
5	lncRNA	74322104	74325657	ENSMUSG00000143368	MGI:5591161	Gm32002	predicted gene, 32002 [Source:MGI Symbol;Acc:MGI:5591161]

5	lncRNA	74322227	74322947	ENSMUSG00000143408	MGI:7829806	Gm73525	predicted gene, 73525 [Source:MGI Symbol;Acc:MGI:7829806]
5	lncRNA	74334851	74348935	ENSMUSG00000139163	MGI:7828812	Gm73027	predicted gene, 73027 [Source:MGI Symbol;Acc:MGI:7828812]
5	lncRNA	74477205	74520529	ENSMUSG00000136303	MGI:5591223	Gm32064	predicted gene, 32064 [Source:MGI Symbol;Acc:MGI:5591223]
5	lncRNA	74565860	74595439	ENSMUSG00000136344	MGI:7827936	Gm72587	predicted gene, 72587 [Source:MGI Symbol;Acc:MGI:7827936]
5	lncRNA	74808344	74819091	ENSMUSG00000121106	MGI:5591463	Gm32304	predicted gene, 32304 [Source:MGI Symbol;Acc:MGI:5591463]
5	lncRNA	74830845	74889528	ENSMUSG00000085657	MGI:3802050	Gm15984	predicted gene 15984 [Source:MGI Symbol;Acc:MGI:3802050]
5	lncRNA	74891092	74895116	ENSMUSG00000085497	MGI:3801786	Gm15985	predicted gene 15985 [Source:MGI Symbol;Acc:MGI:3801786]
5	lncRNA	74910543	74915099	ENSMUSG00000106987	MGI:5662712	Gm42575	predicted gene 42575 [Source:MGI Symbol;Acc:MGI:5662712]
5	lncRNA	74913900	74938534	ENSMUSG00000139893	MGI:7828968	Gm73105	predicted gene, 73105 [Source:MGI Symbol;Acc:MGI:7828968]
5	lncRNA	74949995	74957654	ENSMUSG00000131829	MGI:7826636	Gm71935	predicted gene, 71935 [Source:MGI Symbol;Acc:MGI:7826636]
5	lncRNA	75101786	75109368	ENSMUSG00000141831	MGI:6367169	Gm52779	predicted gene, 52779 [Source:MGI Symbol;Acc:MGI:6367169]
5	lncRNA	75109820	75140100	ENSMUSG00000072874	MGI:3644088	Gm6116	predicted gene 6116 [Source:MGI Symbol;Acc:MGI:3644088]
5	lncRNA	75228008	75232822	ENSMUSG00000137041	MGI:7828266	Gm72752	predicted gene, 72752 [Source:MGI Symbol;Acc:MGI:7828266]
5	lncRNA	75230570	75236830	ENSMUSG00000137002	MGI:7828252	Gm72745	predicted gene, 72745 [Source:MGI Symbol;Acc:MGI:7828252]
5	lncRNA	75270966	75307794	ENSMUSG00000107192	MGI:5011768	Gm19583	predicted gene, 19583 [Source:MGI Symbol;Acc:MGI:5011768]
5	lncRNA	75313716	75314314	ENSMUSG00000106760	MGI:5662939	Gm42802	predicted gene 42802 [Source:MGI Symbol;Acc:MGI:5662939]
5	lncRNA	75376805	75377675	ENSMUSG00000142591	MGI:7829630	Gm73437	predicted gene, 73437 [Source:MGI Symbol;Acc:MGI:7829630]
5	lncRNA	75420845	75424125	ENSMUSG00000127495	MGI:7825815	Gm71521	predicted gene, 71521 [Source:MGI Symbol;Acc:MGI:7825815]

5	lncRNA	75484797	75527661	ENSMUSG00000127213	MGI:7830290	Gm73770	predicted gene, 73770 [Source:MGI Symbol;Acc:MGI:7830290]
5	lncRNA	75527165	75528751	ENSMUSG00000127253	MGI:7825801	Gm71514	predicted gene, 71514 [Source:MGI Symbol;Acc:MGI:7825801]
5	lncRNA	75538146	75548435	ENSMUSG00000123059	MGI:7825352	Gm71287	predicted gene, 71287 [Source:MGI Symbol;Acc:MGI:7825352]
5	lncRNA	75538275	75560066	ENSMUSG00000107386	MGI:5662937	Gm42800	predicted gene 42800 [Source:MGI Symbol;Acc:MGI:5662937]
5	lncRNA	75539613	75539827	ENSMUSG00000123097	MGI:7825354	Gm71288	predicted gene, 71288 [Source:MGI Symbol;Acc:MGI:7825354]
5	lncRNA	75671908	75674187	ENSMUSG00000138324	MGI:7828558	Gm72899	predicted gene, 72899 [Source:MGI Symbol;Acc:MGI:7828558]
5	lncRNA	75817395	75835697	ENSMUSG00000138764	MGI:7828692	Gm72966	predicted gene, 72966 [Source:MGI Symbol;Acc:MGI:7828692]
5	lncRNA	75835827	75860719	ENSMUSG00000129581	MGI:5591717	Gm32558	predicted gene, 32558 [Source:MGI Symbol;Acc:MGI:5591717]
5	lncRNA	75888098	75909603	ENSMUSG00000106754	MGI:5663238	Gm43101	predicted gene 43101 [Source:MGI Symbol;Acc:MGI:5663238]
5	lncRNA	75914216	75919546	ENSMUSG00000106726	MGI:5663237	Gm43100	predicted gene 43100 [Source:MGI Symbol;Acc:MGI:5663237]
5	lncRNA	75914261	75914931	ENSMUSG00000143297	MGI:7829760	Gm73502	predicted gene, 73502 [Source:MGI Symbol;Acc:MGI:7829760]
5	lncRNA	75993652	75996547	ENSMUSG00000107061	MGI:5011775	Gm19590	predicted gene, 19590 [Source:MGI Symbol;Acc:MGI:5011775]
5	lncRNA	75996212	76001817	ENSMUSG00000136464	MGI:7827970	Gm72604	predicted gene, 72604 [Source:MGI Symbol;Acc:MGI:7827970]
5	lncRNA	76007093	76009434	ENSMUSG00000137410	MGI:7828386	Gm72812	predicted gene, 72812 [Source:MGI Symbol;Acc:MGI:7828386]
5	lncRNA	76009898	76014165	ENSMUSG00000100936	MGI:5579571	Gm28865	predicted gene 28865 [Source:MGI Symbol;Acc:MGI:5579571]
5	lncRNA	76012661	76041839	ENSMUSG00000139505	MGI:7828920	Gm73081	predicted gene, 73081 [Source:MGI Symbol;Acc:MGI:7828920]
5	lncRNA	76083539	76092833	ENSMUSG00000134112	MGI:7827374	Gm72304	predicted gene, 72304 [Source:MGI Symbol;Acc:MGI:7827374]
5	lncRNA	76138510	76178133	ENSMUSG00000120170	MGI:5591886	Gm32727	predicted gene, 32727 [Source:MGI Symbol;Acc:MGI:5591886]

5	lncRNA	76160044	76168012	ENSMUSG00000126916	MGI:7825765	Gm71496	predicted gene, 71496 [Source:MGI Symbol;Acc:MGI:7825765]
5	lncRNA	76203710	76219434	ENSMUSG00000106756	MGI:5591939	Gm32780	predicted gene, 32780 [Source:MGI Symbol;Acc:MGI:5591939]
5	lncRNA	76222715	76225128	ENSMUSG00000143677	MGI:7829952	Gm73598	predicted gene, 73598 [Source:MGI Symbol;Acc:MGI:7829952]
5	lncRNA	76306112	76331994	ENSMUSG00000137209	MGI:5593097	Gm33938	predicted gene, 33938 [Source:MGI Symbol;Acc:MGI:5593097]
5	lncRNA	76452723	76453374	ENSMUSG00000098449	MGI:3648350	Gm7467	predicted gene 7467 [Source:MGI Symbol;Acc:MGI:3648350]
5	lncRNA	76479085	76489732	ENSMUSG00000087037	MGI:1921871	4930432L08Rik	RIKEN cDNA 4930432L08 gene [Source:MGI Symbol;Acc:MGI:1921871]
5	lncRNA	76605488	76626206	ENSMUSG00000143679	MGI:7829954	Gm73599	predicted gene, 73599 [Source:MGI Symbol;Acc:MGI:7829954]
5	lncRNA	76741593	76768288	ENSMUSG00000124702	MGI:7825559	Gm71393	predicted gene, 71393 [Source:MGI Symbol;Acc:MGI:7825559]
5	lncRNA	76823250	76825017	ENSMUSG00000132122	MGI:7826714	Gm71974	predicted gene, 71974 [Source:MGI Symbol;Acc:MGI:7826714]
5	lncRNA	76955148	76957770	ENSMUSG00000132052	MGI:7826694	Gm71964	predicted gene, 71964 [Source:MGI Symbol;Acc:MGI:7826694]
5	lncRNA	76964249	76978788	ENSMUSG00000132013	MGI:6367246	Gm52818	predicted gene, 52818 [Source:MGI Symbol;Acc:MGI:6367246]
5	lncRNA	76978887	76981092	ENSMUSG00000132086	MGI:7826708	Gm71971	predicted gene, 71971 [Source:MGI Symbol;Acc:MGI:7826708]
5	lncRNA	77022258	77022921	ENSMUSG00000137719	MGI:7828472	Gm72855	predicted gene, 72855 [Source:MGI Symbol;Acc:MGI:7828472]
5	lncRNA	77053362	77063549	ENSMUSG00000137605	MGI:7828454	Gm72846	predicted gene, 72846 [Source:MGI Symbol;Acc:MGI:7828454]
5	lncRNA	77076215	77095605	ENSMUSG00000121495	MGI:7823087	Gm70141	predicted gene, 70141 [Source:MGI Symbol;Acc:MGI:7823087]
5	lncRNA	77108327	77151020	ENSMUSG00000137527	MGI:6723603	Gm54291	predicted gene, 54291 [Source:MGI Symbol;Acc:MGI:6723603]
5	lncRNA	77149301	77151561	ENSMUSG00000137642			novel transcript
5	lncRNA	77150660	77166723	ENSMUSG00000086632	MGI:3696786	1700112J05Rik	RIKEN cDNA 1700112J05 gene [Source:MGI Symbol;Acc:MGI:3696786]

5	lncRNA	77168164	77168929	ENSMUSG00000137680	MGI:7828468	Gm72853	predicted gene, 72853 [Source:MGI Symbol;Acc:MGI:7828468]
5	lncRNA	77206097	77206721	ENSMUSG00000137760	MGI:7828478	Gm72858	predicted gene, 72858 [Source:MGI Symbol;Acc:MGI:7828478]
5	lncRNA	77215347	77219129	ENSMUSG00000127703	MGI:7825835	Gm71531	predicted gene, 71531 [Source:MGI Symbol;Acc:MGI:7825835]
5	lncRNA	77240550	77250160	ENSMUSG00000087541	MGI:3801917	Hopxos	HOP homeobox, opposite strand [Source:MGI Symbol;Acc:MGI:3801917]
5	lncRNA	77263428	77270195	ENSMUSG00000085858	MGI:3801916	Gm15831	predicted gene 15831 [Source:MGI Symbol;Acc:MGI:3801916]
5	lncRNA	77271261	77278216	ENSMUSG00000137325	MGI:7828356	Gm72797	predicted gene, 72797 [Source:MGI Symbol;Acc:MGI:7828356]
5	lncRNA	77274414	77287851	ENSMUSG00000120897	MGI:7338513	Gm57300	predicted gene, 57300 [Source:MGI Symbol;Acc:MGI:7338513]
5	lncRNA	77283791	77288173	ENSMUSG00000106783	MGI:5624990	Chaer1	[illegible] regulator 1 [Source:MGI Symbol;Acc:MGI:5624990]
5	lncRNA	77293927	77307047	ENSMUSG00000130552	MGI:5593757	Gm34598	predicted gene, 34598 [Source:MGI Symbol;Acc:MGI:5593757]
5	lncRNA	77415158	77416490	ENSMUSG00000131889	MGI:7826644	Gm71939	predicted gene, 71939 [Source:MGI Symbol;Acc:MGI:7826644]
5	lncRNA	77505738	77522641	ENSMUSG00000141011	MGI:7829276	Gm73259	predicted gene, 73259 [Source:MGI Symbol;Acc:MGI:7829276]
5	lncRNA	77534435	77541539	ENSMUSG00000106828	MGI:5593807	Gm34648	predicted gene, 34648 [Source:MGI Symbol;Acc:MGI:5593807]
5	lncRNA	77581834	77621066	ENSMUSG00000107232	MGI:1916620	1700017L05Rik	RIKEN cDNA 1700017L05 gene [Source:MGI Symbol;Acc:MGI:1916620]
5	lncRNA	77610344	77613256	ENSMUSG00000143522	MGI:7829878	Gm73561	predicted gene, 73561 [Source:MGI Symbol;Acc:MGI:7829878]
5	lncRNA	77630302	77642268	ENSMUSG00000106985	MGI:5662898	Gm42761	predicted gene 42761 [Source:MGI Symbol;Acc:MGI:5662898]
5	lncRNA	77659041	77659595	ENSMUSG00000121772	MGI:7824207	Gm70707	predicted gene, 70707 [Source:MGI Symbol;Acc:MGI:7824207]
5	lncRNA	77659055	77694753	ENSMUSG00000123203	MGI:7825375	Gm71299	predicted gene, 71299 [Source:MGI Symbol;Acc:MGI:7825375]
5	lncRNA	77661046	77681112	ENSMUSG00000123236	MGI:7825379	Gm71301	predicted gene, 71301 [Source:MGI Symbol;Acc:MGI:7825379]

5	lncRNA	77725236	77728017	ENSMUSG00000129668	MGI:7826011	Gm71620	predicted gene, 71620 [Source:MGI Symbol;Acc:MGI:7826011]
5	lncRNA	77756653	77792554	ENSMUSG00000133181	MGI:7827006	Gm72120	predicted gene, 72120 [Source:MGI Symbol;Acc:MGI:7827006]
5	lncRNA	77787663	77822531	ENSMUSG00000133218	MGI:7827046	Gm72140	predicted gene, 72140 [Source:MGI Symbol;Acc:MGI:7827046]
5	lncRNA	77838851	77840079	ENSMUSG00000123568	MGI:7825419	Gm71321	predicted gene, 71321 [Source:MGI Symbol;Acc:MGI:7825419]
5	lncRNA	77891693	77909996	ENSMUSG00000138944	MGI:7828760	Gm73000	predicted gene, 73000 [Source:MGI Symbol;Acc:MGI:7828760]
5	lncRNA	77986533	78096901	ENSMUSG00000127046			novel transcript
5	lncRNA	78987656	79002547	ENSMUSG00000124166	MGI:7825493	Gm71359	predicted gene, 71359 [Source:MGI Symbol;Acc:MGI:7825493]
5	lncRNA	79002682	79048131	ENSMUSG00000134542	MGI:7827458	Gm72346	predicted gene, 72346 [Source:MGI Symbol;Acc:MGI:7827458]
5	lncRNA	79298740	79299452	ENSMUSG00000129544	MGI:7825989	Gm71609	predicted gene, 71609 [Source:MGI Symbol;Acc:MGI:7825989]
5	lncRNA	79785905	79786318	ENSMUSG00000127094	MGI:7825789	Gm71508	predicted gene, 71508 [Source:MGI Symbol;Acc:MGI:7825789]
5	lncRNA	80797068	80800099	ENSMUSG00000137734	MGI:7828474	Gm72856	predicted gene, 72856 [Source:MGI Symbol;Acc:MGI:7828474]
5	lncRNA	80925626	81002961	ENSMUSG00000128907	MGI:7825937	Gm71582	predicted gene, 71582 [Source:MGI Symbol;Acc:MGI:7825937]
5	lncRNA	81171512	81176796	ENSMUSG00000124141	MGI:7825489	Gm71357	predicted gene, 71357 [Source:MGI Symbol;Acc:MGI:7825489]
5	lncRNA	81701680	81703966	ENSMUSG00000121076	MGI:7338533	Gm57310	predicted gene, 57310 [Source:MGI Symbol;Acc:MGI:7338533]
5	lncRNA	81939752	81946273	ENSMUSG00000087431	MGI:3801819	Gm16054	predicted gene 16054 [Source:MGI Symbol;Acc:MGI:3801819]
5	lncRNA	82057851	82074134	ENSMUSG00000132904	MGI:7826882	Gm72058	predicted gene, 72058 [Source:MGI Symbol;Acc:MGI:7826882]
5	lncRNA	82606825	82611673	ENSMUSG00000136333	MGI:7827932	Gm72585	predicted gene, 72585 [Source:MGI Symbol;Acc:MGI:7827932]
5	lncRNA	82787205	82800131	ENSMUSG00000135173	MGI:7827642	Gm72439	predicted gene, 72439 [Source:MGI Symbol;Acc:MGI:7827642]

5	lncRNA	82818662	82967715	ENSMUSG00000123351	MGI:7825395	Gm71309	predicted gene, 71309 [Source:MGI Symbol;Acc:MGI:7825395]
5	lncRNA	83148035	83160095	ENSMUSG00000128479	MGI:7825903	Gm71565	predicted gene, 71565 [Source:MGI Symbol;Acc:MGI:7825903]
5	lncRNA	83159825	83164649	ENSMUSG00000128511	MGI:7825907	Gm71567	predicted gene, 71567 [Source:MGI Symbol;Acc:MGI:7825907]
5	lncRNA	83920313	83961863	ENSMUSG00000122804	MGI:7825313	Gm71266	predicted gene, 71266 [Source:MGI Symbol;Acc:MGI:7825313]
5	lncRNA	83958251	83961799	ENSMUSG00000122846	MGI:7825327	Gm71273	predicted gene, 71273 [Source:MGI Symbol;Acc:MGI:7825327]
5	lncRNA	83996968	84037431	ENSMUSG00000137987	MGI:7828508	Gm72873	predicted gene, 72873 [Source:MGI Symbol;Acc:MGI:7828508]
5	lncRNA	84037198	84042514	ENSMUSG00000138029	MGI:7828516	Gm72877	predicted gene, 72877 [Source:MGI Symbol;Acc:MGI:7828516]
5	lncRNA	84271075	84272260	ENSMUSG00000138269	MGI:7830314	Gm73784	predicted gene, 73784 [Source:MGI Symbol;Acc:MGI:7830314]
5	lncRNA	84565315	84614936	ENSMUSG00000132128	MGI:7826718	Gm71976	predicted gene, 71976 [Source:MGI Symbol;Acc:MGI:7826718]
5	lncRNA	84576664	84580479	ENSMUSG00000106598	MGI:5663669	Gm43532	predicted gene 43532 [Source:MGI Symbol;Acc:MGI:5663669]
5	lncRNA	84860434	84861906	ENSMUSG00000124137	MGI:7825487	Gm71356	predicted gene, 71356 [Source:MGI Symbol;Acc:MGI:7825487]
5	lncRNA	85848190	85848619	ENSMUSG00000142369	MGI:7829558	Gm73400	predicted gene, 73400 [Source:MGI Symbol;Acc:MGI:7829558]
5	lncRNA	85853867	85859466	ENSMUSG00000133322	MGI:5594410	Gm35251	predicted gene, 35251 [Source:MGI Symbol;Acc:MGI:5594410]
5	lncRNA	85868633	85870610	ENSMUSG00000104878	MGI:5663704	Gm43567	predicted gene 43567 [Source:MGI Symbol;Acc:MGI:5663704]
5	lncRNA	86053129	86057573	ENSMUSG00000138698	MGI:7828670	Gm72955	predicted gene, 72955 [Source:MGI Symbol;Acc:MGI:7828670]
5	lncRNA	86213524	86216687	ENSMUSG00000130557	MGI:7826228	Gm71730	predicted gene, 71730 [Source:MGI Symbol;Acc:MGI:7826228]
5	lncRNA	86214671	86215754	ENSMUSG00000121702	MGI:7824203	Gm70705	predicted gene, 70705 [Source:MGI Symbol;Acc:MGI:7824203]
5	lncRNA	86320884	86321767	ENSMUSG00000132971	MGI:7826904	Gm72069	predicted gene, 72069 [Source:MGI Symbol;Acc:MGI:7826904]

5	lncRNA	86831709	86834499	ENSMUSG00000106507	MGI:5663193	Gm43056	predicted gene 43056 [Source:MGI Symbol;Acc:MGI:5663193]
5	lncRNA	86876758	86907595	ENSMUSG00000089851	MGI:4414999	Gm16579	predicted gene 16579 [Source:MGI Symbol;Acc:MGI:4414999]
5	lncRNA	86876765	86964965	ENSMUSG00000105704	MGI:5663192	Gm43055	predicted gene 43055 [Source:MGI Symbol;Acc:MGI:5663192]
5	lncRNA	86914994	86930892	ENSMUSG00000104937	MGI:5663194	Gm43057	predicted gene 43057 [Source:MGI Symbol;Acc:MGI:5663194]
5	lncRNA	86951343	86952166	ENSMUSG00000124176	MGI:7825497	Gm71361	predicted gene, 71361 [Source:MGI Symbol;Acc:MGI:7825497]
5	lncRNA	87051725	87053341	ENSMUSG00000130987	MGI:7826348	Gm71791	predicted gene, 71791 [Source:MGI Symbol;Acc:MGI:7826348]
5	lncRNA	87093304	87124242	ENSMUSG00000131191	MGI:7826430	Gm71832	predicted gene, 71832 [Source:MGI Symbol;Acc:MGI:7826430]
5	lncRNA	87108370	87109945	ENSMUSG00000131214	MGI:7830368	Gm73811	predicted gene, 73811 [Source:MGI Symbol;Acc:MGI:7830368]
5	lncRNA	87144907	87148517	ENSMUSG00000136916	MGI:7828234	Gm72736	predicted gene, 72736 [Source:MGI Symbol;Acc:MGI:7828234]
5	lncRNA	87166312	87190056	ENSMUSG00000122385	MGI:7825259	Gm71239	predicted gene, 71239 [Source:MGI Symbol;Acc:MGI:7825259]
5	lncRNA	87520452	87527815	ENSMUSG00000122459	MGI:7830372	Gm73813	predicted gene, 73813 [Source:MGI Symbol;Acc:MGI:7830372]
5	lncRNA	87657298	87664390	ENSMUSG00000141278	MGI:7829378	Gm73310	predicted gene, 73310 [Source:MGI Symbol;Acc:MGI:7829378]
5	lncRNA	87903460	87998719	ENSMUSG00000104839	MGI:5662931	Gm42794	predicted gene 42794 [Source:MGI Symbol;Acc:MGI:5662931]
5	lncRNA	87998218	88000872	ENSMUSG00000121715			DAZ interacting protein 1 pseudogene [Source:NCBI gene (formerly Entrezgene);Acc:654494]
5	lncRNA	88055713	88127236	ENSMUSG00000099605	MGI:1920721	1700066N21Rik	RIKEN cDNA 1700066N21 gene [Source:MGI Symbol;Acc:MGI:1920721]
5	lncRNA	88111344	88114211	ENSMUSG00000055961	MGI:2670982	BC051076	cDNA sequence BC051076 [Source:MGI Symbol;Acc:MGI:2670982]
5	lncRNA	88310430	88315615	ENSMUSG00000139380	MGI:7828856	Gm73049	predicted gene, 73049 [Source:MGI Symbol;Acc:MGI:7828856]

5	lncRNA	88344830	88359055	ENSMUSG00000139858			novel transcript
5	lncRNA	88439212	88599170	ENSMUSG00000126970	MGI:1920562	1700041A01Rik	RIKEN cDNA 1700041A01 gene [Source:MGI Symbol;Acc:MGI:1920562]
5	lncRNA	88692315	88712827	ENSMUSG00000120316	MGI:7338457	Gm57272	predicted gene, 57272 [Source:MGI Symbol;Acc:MGI:7338457]
5	lncRNA	88701671	88702091	ENSMUSG00000134393	MGI:7827442	Gm72338	predicted gene, 72338 [Source:MGI Symbol;Acc:MGI:7827442]
5	lncRNA	88728109	88732185	ENSMUSG00000134225	MGI:7827422	Gm72328	predicted gene, 72328 [Source:MGI Symbol;Acc:MGI:7827422]
5	lncRNA	88728253	88728997	ENSMUSG00000134311	MGI:7827436	Gm72335	predicted gene, 72335 [Source:MGI Symbol;Acc:MGI:7827436]
5	lncRNA	88729359	88729963	ENSMUSG00000134350	MGI:7827438	Gm72336	predicted gene, 72336 [Source:MGI Symbol;Acc:MGI:7827438]
5	lncRNA	88824082	88832390	ENSMUSG00000129340	MGI:7825973	Gm71600	predicted gene, 71600 [Source:MGI Symbol;Acc:MGI:7825973]
5	lncRNA	88857948	88868692	ENSMUSG00000121172	MGI:7338561	Gm57324	predicted gene, 57324 [Source:MGI Symbol;Acc:MGI:7338561]
5	lncRNA	88888201	88891403	ENSMUSG00000132815	MGI:7826846	Gm72040	predicted gene, 72040 [Source:MGI Symbol;Acc:MGI:7826846]
5	lncRNA	88941693	88961351	ENSMUSG00000105640	MGI:5663049	Gm42912	predicted gene 42912 [Source:MGI Symbol;Acc:MGI:5663049]
5	lncRNA	89351748	89353489	ENSMUSG00000126258	MGI:7825697	Gm71462	predicted gene, 71462 [Source:MGI Symbol;Acc:MGI:7825697]
5	lncRNA	89356080	89356956	ENSMUSG00000121166	MGI:7338557	Gm57322	predicted gene, 57322 [Source:MGI Symbol;Acc:MGI:7338557]
5	lncRNA	89370386	89371742	ENSMUSG00000130398	MGI:7826210	Gm71721	predicted gene, 71721 [Source:MGI Symbol;Acc:MGI:7826210]
5	lncRNA	89393196	89410563	ENSMUSG00000143172	MGI:7829738	Gm73491	predicted gene, 73491 [Source:MGI Symbol;Acc:MGI:7829738]
5	lncRNA	89425606	89434146	ENSMUSG00000142798	MGI:7829670	Gm73457	predicted gene, 73457 [Source:MGI Symbol;Acc:MGI:7829670]
5	lncRNA	89503065	89589568	ENSMUSG00000137094	MGI:7828294	Gm72766	predicted gene, 72766 [Source:MGI Symbol;Acc:MGI:7828294]
5	lncRNA	89537350	89540646	ENSMUSG00000137136	MGI:7828298	Gm72768	predicted gene, 72768 [Source:MGI Symbol;Acc:MGI:7828298]

5	lncRNA	89540266	89541409	ENSMUSG00000137177	MGI:7828300	Gm72769	predicted gene, 72769 [Source:MGI Symbol;Acc:MGI:7828300]
5	lncRNA	89611209	89647450	ENSMUSG00000126175	MGI:7825685	Gm71456	predicted gene, 71456 [Source:MGI Symbol;Acc:MGI:7825685]
5	lncRNA	89832053	89837636	ENSMUSG00000126217	MGI:7825689	Gm71458	predicted gene, 71458 [Source:MGI Symbol;Acc:MGI:7825689]
5	lncRNA	90099232	90125740	ENSMUSG00000130148	MGI:7826134	Gm71683	predicted gene, 71683 [Source:MGI Symbol;Acc:MGI:7826134]
5	lncRNA	90267684	90298205	ENSMUSG00000135627	MGI:7827746	Gm72491	predicted gene, 72491 [Source:MGI Symbol;Acc:MGI:7827746]
5	lncRNA	90341182	90343029	ENSMUSG00000140834	MGI:7829230	Gm73236	predicted gene, 73236 [Source:MGI Symbol;Acc:MGI:7829230]
5	lncRNA	90344681	90348377	ENSMUSG00000140416	MGI:7829148	Gm73195	predicted gene, 73195 [Source:MGI Symbol;Acc:MGI:7829148]
5	lncRNA	90394656	90436607	ENSMUSG00000130758	MGI:7826284	Gm71758	predicted gene, 71758 [Source:MGI Symbol;Acc:MGI:7826284]
5	lncRNA	90514479	90516352	ENSMUSG00000054945	MGI:3641866	Gm9958	predicted gene 9958 [Source:MGI Symbol;Acc:MGI:3641866]
5	lncRNA	90562326	90587746	ENSMUSG00000123171	MGI:5595158	Gm35999	predicted gene, 35999 [Source:MGI Symbol;Acc:MGI:5595158]
5	lncRNA	90646939	90663790	ENSMUSG00000106788	MGI:5663586	Gm43449	predicted gene 43449 [Source:MGI Symbol;Acc:MGI:5663586]
5	lncRNA	90701058	90736936	ENSMUSG00000106863	MGI:5624994	Gm42109	predicted gene, 42109 [Source:MGI Symbol;Acc:MGI:5624994]
5	lncRNA	90750130	90754368	ENSMUSG00000139329	MGI:7828840	Gm73041	predicted gene, 73041 [Source:MGI Symbol;Acc:MGI:7828840]
5	lncRNA	90796599	90828142	ENSMUSG00000130379	MGI:7826200	Gm71716	predicted gene, 71716 [Source:MGI Symbol;Acc:MGI:7826200]
5	lncRNA	90860787	90876723	ENSMUSG00000141359	MGI:5595243	Gm36084	predicted gene, 36084 [Source:MGI Symbol;Acc:MGI:5595243]
5	lncRNA	90906282	90910220	ENSMUSG00000135043	MGI:7827592	Gm72413	predicted gene, 72413 [Source:MGI Symbol;Acc:MGI:7827592]
5	lncRNA	90926762	90933050	ENSMUSG00000136230	MGI:7827894	Gm72565	predicted gene, 72565 [Source:MGI Symbol;Acc:MGI:7827894]
5	lncRNA	90980187	90983810	ENSMUSG00000107044	MGI:5663223	Gm43086	predicted gene 43086 [Source:MGI Symbol;Acc:MGI:5663223]

5	lncRNA	91007891	91036327	ENSMUSG00000142520	MGI:7829610	Gm73427	predicted gene, 73427 [Source:MGI Symbol;Acc:MGI:7829610]
5	lncRNA	91047056	91051063	ENSMUSG00000140717	MGI:7829222	Gm73232	predicted gene, 73232 [Source:MGI Symbol;Acc:MGI:7829222]
5	lncRNA	91070591	91072764	ENSMUSG00000107218	MGI:5662667	Gm42530	predicted gene 42530 [Source:MGI Symbol;Acc:MGI:5662667]
5	lncRNA	91158799	91162369	ENSMUSG00000131708	MGI:6367248	Gm52819	predicted gene, 52819 [Source:MGI Symbol;Acc:MGI:6367248]
5	lncRNA	91198968	91283840	ENSMUSG00000107350	MGI:5011795	Gm19610	predicted gene, 19610 [Source:MGI Symbol;Acc:MGI:5011795]
5	lncRNA	91399459	91455916	ENSMUSG00000107198	MGI:5011804	Gm19619	predicted gene, 19619 [Source:MGI Symbol;Acc:MGI:5011804]
5	lncRNA	91572101	91597783	ENSMUSG00000133134	MGI:5624995	Gm42110	predicted gene, 42110 [Source:MGI Symbol;Acc:MGI:5624995]
5	lncRNA	91596864	91604421	ENSMUSG00000133170	MGI:7826996	Gm72115	predicted gene, 72115 [Source:MGI Symbol;Acc:MGI:7826996]
5	lncRNA	91838925	91844544	ENSMUSG00000136387	MGI:7827950	Gm72594	predicted gene, 72594 [Source:MGI Symbol;Acc:MGI:7827950]
5	lncRNA	91856334	91889188	ENSMUSG00000129304	MGI:5595381	Gm36222	predicted gene, 36222 [Source:MGI Symbol;Acc:MGI:5595381]
5	lncRNA	91888385	91915778	ENSMUSG00000099468	MGI:5578977	Gm28271	predicted gene 28271 [Source:MGI Symbol;Acc:MGI:5578977]
5	lncRNA	91974673	91992960	ENSMUSG00000136162	MGI:7827844	Gm72540	predicted gene, 72540 [Source:MGI Symbol;Acc:MGI:7827844]
5	lncRNA	91979238	92000481	ENSMUSG00000136120	MGI:5595462	Gm36303	predicted gene, 36303 [Source:MGI Symbol;Acc:MGI:5595462]
5	lncRNA	92009494	92092978	ENSMUSG00000143999	MGI:5595516	Gm36357	predicted gene, 36357 [Source:MGI Symbol;Acc:MGI:5595516]
5	lncRNA	92059257	92061279	ENSMUSG00000107301	MGI:5663825	Gm43688	predicted gene 43688 [Source:MGI Symbol;Acc:MGI:5663825]
5	lncRNA	92110248	92170798	ENSMUSG00000121865			THAP domain containing 6 [Source:NCBI gene (formerly Entrezgene);Acc:381650]
5	lncRNA	92116719	92127032	ENSMUSG00000129673	MGI:7826013	Gm71621	predicted gene, 71621 [Source:MGI Symbol;Acc:MGI:7826013]
5	lncRNA	92190660	92201237	ENSMUSG00000129634	MGI:7826007	Gm71618	predicted gene, 71618 [Source:MGI Symbol;Acc:MGI:7826007]

5	lncRNA	92198199	92198445	ENSMUSG00000129945	MGI:7826102	Gm71666	predicted gene, 71666 [Source:MGI Symbol;Acc:MGI:7826102]
5	lncRNA	92230778	92233700	ENSMUSG00000129828	MGI:7826064	Gm71647	predicted gene, 71647 [Source:MGI Symbol;Acc:MGI:7826064]
5	lncRNA	92232457	92233892	ENSMUSG00000129902	MGI:7826082	Gm71656	predicted gene, 71656 [Source:MGI Symbol;Acc:MGI:7826082]
5	lncRNA	92273407	92285584	ENSMUSG00000129597	MGI:7825999	Gm71614	predicted gene, 71614 [Source:MGI Symbol;Acc:MGI:7825999]
5	lncRNA	92274877	92277972	ENSMUSG00000120423	MGI:2444469	D430040L24Rik	RIKEN cDNA D430040L24 gene [Source:MGI Symbol;Acc:MGI:2444469]
5	lncRNA	92341678	92345320	ENSMUSG00000129758	MGI:7826025	Gm71627	predicted gene, 71627 [Source:MGI Symbol;Acc:MGI:7826025]
5	lncRNA	92358337	92360533	ENSMUSG00000129868	MGI:5624998	Gm42113	predicted gene, 42113 [Source:MGI Symbol;Acc:MGI:5624998]
5	lncRNA	92364403	92372316	ENSMUSG00000129715	MGI:5624997	Gm42112	predicted gene, 42112 [Source:MGI Symbol;Acc:MGI:5624997]
5	lncRNA	92437911	92450619	ENSMUSG00000106698	MGI:5663736	Gm43599	predicted gene 43599 [Source:MGI Symbol;Acc:MGI:5663736]
5	lncRNA	92582183	92583916	ENSMUSG00000131613	MGI:5624999	Gm42114	predicted gene, 42114 [Source:MGI Symbol;Acc:MGI:5624999]
5	lncRNA	92653679	92660306	ENSMUSG00000130185	MGI:7826142	Gm71687	predicted gene, 71687 [Source:MGI Symbol;Acc:MGI:7826142]
5	lncRNA	92690902	92699275	ENSMUSG00000138080	MGI:7828520	Gm72879	predicted gene, 72879 [Source:MGI Symbol;Acc:MGI:7828520]
5	lncRNA	92822888	92824425	ENSMUSG00000139854	MGI:7828962	Gm73102	predicted gene, 73102 [Source:MGI Symbol;Acc:MGI:7828962]
5	lncRNA	92830033	92831611	ENSMUSG00000141749	MGI:7829442	Gm73342	predicted gene, 73342 [Source:MGI Symbol;Acc:MGI:7829442]
5	lncRNA	92830518	92831151	ENSMUSG00000141780	MGI:7829448	Gm73345	predicted gene, 73345 [Source:MGI Symbol;Acc:MGI:7829448]
5	lncRNA	92884064	92885301	ENSMUSG00000092266	MGI:5141965	Gm20500	predicted gene 20500 [Source:MGI Symbol;Acc:MGI:5141965]
5	lncRNA	93065634	93073037	ENSMUSG00000142400	MGI:7829566	Gm73404	predicted gene, 73404 [Source:MGI Symbol;Acc:MGI:7829566]
5	lncRNA	93151472	93167612	ENSMUSG00000140081	MGI:5595923	Gm36764	predicted gene, 36764 [Source:MGI Symbol;Acc:MGI:5595923]

5	lncRNA	93153559	93162449	ENSMUSG00000140123	MGI:7829050	Gm73146	predicted gene, 73146 [Source:MGI Symbol;Acc:MGI:7829050]
5	lncRNA	93174253	93176495	ENSMUSG00000140404	MGI:7829138	Gm73190	predicted gene, 73190 [Source:MGI Symbol;Acc:MGI:7829138]
5	lncRNA	93185357	93186588	ENSMUSG00000128457	MGI:7825899	Gm71563	predicted gene, 71563 [Source:MGI Symbol;Acc:MGI:7825899]
5	lncRNA	93193166	93199034	ENSMUSG00000133837	MGI:7827236	Gm72235	predicted gene, 72235 [Source:MGI Symbol;Acc:MGI:7827236]
5	lncRNA	93225033	93241274	ENSMUSG00000129086	MGI:7825953	Gm71590	predicted gene, 71590 [Source:MGI Symbol;Acc:MGI:7825953]
5	lncRNA	93304425	93309887	ENSMUSG00000122927	MGI:7825338	Gm71280	predicted gene, 71280 [Source:MGI Symbol;Acc:MGI:7825338]
5	lncRNA	93353325	93353458	ENSMUSG00000122968	MGI:7825342	Gm71282	predicted gene, 71282 [Source:MGI Symbol;Acc:MGI:7825342]
5	lncRNA	93365844	93388691	ENSMUSG00000125536	MGI:7825633	Gm71430	predicted gene, 71430 [Source:MGI Symbol;Acc:MGI:7825633]
5	lncRNA	93452242	93454353	ENSMUSG00000134089	MGI:7827340	Gm72287	predicted gene, 72287 [Source:MGI Symbol;Acc:MGI:7827340]
5	lncRNA	93714055	93723875	ENSMUSG00000125231	MGI:7825599	Gm71413	predicted gene, 71413 [Source:MGI Symbol;Acc:MGI:7825599]
5	lncRNA	94168402	94212535	ENSMUSG00000126234	MGI:7825695	Gm71461	predicted gene, 71461 [Source:MGI Symbol;Acc:MGI:7825695]
5	lncRNA	94424246	94449998	ENSMUSG00000130196	MGI:7826144	Gm71688	predicted gene, 71688 [Source:MGI Symbol;Acc:MGI:7826144]
5	lncRNA	94450328	94454655	ENSMUSG00000121888			PRAME like 39, pseudogene [Source:NCBI gene (formerly Entrezgene);Acc:331195]
5	lncRNA	94487608	94532938	ENSMUSG00000121889			PRAME like 52, pseudogene [Source:NCBI gene (formerly Entrezgene);Acc:100554]
5	lncRNA	94553273	94562789	ENSMUSG00000125893	MGI:7825663	Gm71445	predicted gene, 71445 [Source:MGI Symbol;Acc:MGI:7825663]
5	lncRNA	94734291	94759548	ENSMUSG00000129583	MGI:7825997	Gm71613	predicted gene, 71613 [Source:MGI Symbol;Acc:MGI:7825997]
5	lncRNA	94914344	94916550	ENSMUSG00000139846	MGI:7828960	Gm73101	predicted gene, 73101 [Source:MGI Symbol;Acc:MGI:7828960]
5	lncRNA	95073209	95098491	ENSMUSG00000125730	MGI:5625003	Gm42118	predicted gene, 42118 [Source:MGI Symbol;Acc:MGI:5625003]

5	lncRNA	95136457	95171649	ENSMUSG00000121887	MGI:7824219	Gm70713	predicted gene, 70713 [Source:MGI Symbol;Acc:MGI:7824219]
5	lncRNA	95390289	95431161	ENSMUSG00000138988	MGI:7830506	Gm73881	predicted gene, 73881 [Source:MGI Symbol;Acc:MGI:7830506]
5	lncRNA	95751675	95753276	ENSMUSG00000140862	MGI:7830514	Gm73885	predicted gene, 73885 [Source:MGI Symbol;Acc:MGI:7830514]
5	lncRNA	95834235	95874723	ENSMUSG00000127000	MGI:7825775	Gm71501	predicted gene, 71501 [Source:MGI Symbol;Acc:MGI:7825775]
5	lncRNA	96310583	96310919	ENSMUSG00000135924	MGI:7827792	Gm72514	predicted gene, 72514 [Source:MGI Symbol;Acc:MGI:7827792]
5	lncRNA	96351064	96357927	ENSMUSG00000133365	MGI:7827136	Gm72185	predicted gene, 72185 [Source:MGI Symbol;Acc:MGI:7827136]
5	lncRNA	96512180	96521816	ENSMUSG00000135575	MGI:7827738	Gm72487	predicted gene, 72487 [Source:MGI Symbol;Acc:MGI:7827738]
5	lncRNA	96525726	96560691	ENSMUSG00000124901	MGI:7825573	Gm71400	predicted gene, 71400 [Source:MGI Symbol;Acc:MGI:7825573]
5	lncRNA	96604248	96605611	ENSMUSG00000106263	MGI:5592209	Gm33050	predicted gene, 33050 [Source:MGI Symbol;Acc:MGI:5592209]
5	lncRNA	96881997	96887570	ENSMUSG00000106526	MGI:5662741	Gm42604	predicted gene 42604 [Source:MGI Symbol;Acc:MGI:5662741]
5	lncRNA	96963839	96966838	ENSMUSG00000134078	MGI:7827334	Gm72284	predicted gene, 72284 [Source:MGI Symbol;Acc:MGI:7827334]
5	lncRNA	96988999	96989775	ENSMUSG00000134123	MGI:7827376	Gm72305	predicted gene, 72305 [Source:MGI Symbol;Acc:MGI:7827376]
5	lncRNA	96997301	97000805	ENSMUSG00000104955	MGI:1916674	1700016F12Rik	RIKEN cDNA 1700016F12 gene [Source:MGI Symbol;Acc:MGI:1916674]
5	lncRNA	97004518	97005073	ENSMUSG00000123827	MGI:7825449	Gm71336	predicted gene, 71336 [Source:MGI Symbol;Acc:MGI:7825449]
5	lncRNA	97016638	97028257	ENSMUSG00000133145	MGI:7826990	Gm72112	predicted gene, 72112 [Source:MGI Symbol;Acc:MGI:7826990]
5	lncRNA	97018970	97106952	ENSMUSG00000106357	MGI:3648644	Gm8013	predicted gene 8013 [Source:MGI Symbol;Acc:MGI:3648644]
5	lncRNA	97045768	97089184	ENSMUSG00000105283	MGI:5592529	Gm33370	predicted gene, 33370 [Source:MGI Symbol;Acc:MGI:5592529]
5	lncRNA	97104075	97131508	ENSMUSG00000133056	MGI:7826962	Gm72098	predicted gene, 72098 [Source:MGI Symbol;Acc:MGI:7826962]

5	lncRNA	97111030	97111176	ENSMUSG00000133184	MGI:7827008	Gm72121	predicted gene, 72121 [Source:MGI Symbol;Acc:MGI:7827008]
5	lncRNA	97144065	97145223	ENSMUSG00000105006	MGI:3779894	Gm9484	predicted gene 9484 [Source:MGI Symbol;Acc:MGI:3779894]
5	lncRNA	97258944	97357574	ENSMUSG00000104736	MGI:5592768	Gm33609	predicted gene, 33609 [Source:MGI Symbol;Acc:MGI:5592768]
5	lncRNA	97356287	97371487	ENSMUSG00000133503	MGI:6723653	Gm54317	predicted gene, 54317 [Source:MGI Symbol;Acc:MGI:6723653]
5	lncRNA	97411967	97412790	ENSMUSG00000131920	MGI:7826656	Gm71945	predicted gene, 71945 [Source:MGI Symbol;Acc:MGI:7826656]
5	lncRNA	97418130	97419368	ENSMUSG00000131486	MGI:7826538	Gm71886	predicted gene, 71886 [Source:MGI Symbol;Acc:MGI:7826538]
5	lncRNA	97445838	97475349	ENSMUSG00000135690	MGI:7827764	Gm72500	predicted gene, 72500 [Source:MGI Symbol;Acc:MGI:7827764]
5	lncRNA	97449018	97497427	ENSMUSG00000104717	MGI:5593128	Gm33969	predicted gene, 33969 [Source:MGI Symbol;Acc:MGI:5593128]
5	lncRNA	97503355	97511173	ENSMUSG00000141986	MGI:7829476	Gm73359	predicted gene, 73359 [Source:MGI Symbol;Acc:MGI:7829476]
5	lncRNA	97540251	97790042	ENSMUSG00000097516	MGI:1923064	4930467D21Rik	RIKEN cDNA 4930467D21 gene [Source:MGI Symbol;Acc:MGI:1923064]
5	lncRNA	97546428	97547658	ENSMUSG00000125869	MGI:7825659	Gm71443	predicted gene, 71443 [Source:MGI Symbol;Acc:MGI:7825659]
5	lncRNA	97685798	97702355	ENSMUSG00000125831	MGI:7825651	Gm71439	predicted gene, 71439 [Source:MGI Symbol;Acc:MGI:7825651]
5	lncRNA	97848655	97980411	ENSMUSG00000120552	MGI:6723655	Gm54318	predicted gene, 54318 [Source:MGI Symbol;Acc:MGI:6723655]
5	lncRNA	97969825	97987221	ENSMUSG00000137242	MGI:5593256	Gm34097	predicted gene, 34097 [Source:MGI Symbol;Acc:MGI:5593256]
5	lncRNA	98119363	98127015	ENSMUSG00000127655	MGI:7825831	Gm71529	predicted gene, 71529 [Source:MGI Symbol;Acc:MGI:7825831]
5	lncRNA	98164242	98175481	ENSMUSG00000127610	MGI:5593385	Gm34226	predicted gene, 34226 [Source:MGI Symbol;Acc:MGI:5593385]
5	lncRNA	98226134	98228326	ENSMUSG00000143266	MGI:7829754	Gm73499	predicted gene, 73499 [Source:MGI Symbol;Acc:MGI:7829754]
5	lncRNA	98282899	98286917	ENSMUSG00000130319	MGI:5625006	Gm42121	predicted gene, 42121 [Source:MGI Symbol;Acc:MGI:5625006]

5	lncRNA	98330980	98333843	ENSMUSG00000045238	MGI:3696858	A730035I17Rik	RIKEN cDNA A730035I17 gene [Source:MGI Symbol;Acc:MGI:3696858]
5	lncRNA	98473766	98477169	ENSMUSG00000136326	MGI:7827928	Gm72583	predicted gene, 72583 [Source:MGI Symbol;Acc:MGI:7827928]
5	lncRNA	98627618	98661123	ENSMUSG00000136178	MGI:5593634	Gm34475	predicted gene, 34475 [Source:MGI Symbol;Acc:MGI:5593634]
5	lncRNA	98686346	98690045	ENSMUSG00000136289	MGI:7827920	Gm72579	predicted gene, 72579 [Source:MGI Symbol;Acc:MGI:7827920]
5	lncRNA	98701480	98714873	ENSMUSG00000067101	MGI:1922750	1700010H22Rik	RIKEN cDNA 1700010H22 gene [Source:MGI Symbol;Acc:MGI:1922750]
5	lncRNA	98714907	98720731	ENSMUSG00000136251	MGI:5625007	Gm42122	predicted gene, 42122 [Source:MGI Symbol;Acc:MGI:5625007]
5	lncRNA	98981527	99002388	ENSMUSG00000123838	MGI:5593722	Gm34563	predicted gene, 34563 [Source:MGI Symbol;Acc:MGI:5593722]
5	lncRNA	99026039	99039362	ENSMUSG00000122946	MGI:5625008	Gm42123	predicted gene, 42123 [Source:MGI Symbol;Acc:MGI:5625008]
5	lncRNA	99185150	99190769	ENSMUSG00000137970	MGI:7828506	Gm72872	predicted gene, 72872 [Source:MGI Symbol;Acc:MGI:7828506]
5	lncRNA	99186922	99319280	ENSMUSG00000105579	MGI:5663388	Gm43251	predicted gene 43251 [Source:MGI Symbol;Acc:MGI:5663388]
5	lncRNA	99368625	99369114	ENSMUSG00000125182	MGI:7825591	Gm71409	predicted gene, 71409 [Source:MGI Symbol;Acc:MGI:7825591]
5	lncRNA	99421954	99427143	ENSMUSG00000105686	MGI:5594553	Gm35394	predicted gene, 35394 [Source:MGI Symbol;Acc:MGI:5594553]
5	lncRNA	99507200	99507903	ENSMUSG00000129574	MGI:7825993	Gm71611	predicted gene, 71611 [Source:MGI Symbol;Acc:MGI:7825993]
5	lncRNA	99639936	99650712	ENSMUSG00000140090	MGI:7829036	Gm73139	predicted gene, 73139 [Source:MGI Symbol;Acc:MGI:7829036]
5	lncRNA	99656208	99658940	ENSMUSG00000104753	MGI:5594331	Gm35172	predicted gene, 35172 [Source:MGI Symbol;Acc:MGI:5594331]
5	lncRNA	99657729	99658626	ENSMUSG00000090006	MGI:3801791	Gm16227	predicted gene 16227 [Source:MGI Symbol;Acc:MGI:3801791]
5	lncRNA	99765244	99774928	ENSMUSG00000090146	MGI:1921068	4930405H06Rik	RIKEN cDNA 4930405H06 gene [Source:MGI Symbol;Acc:MGI:1921068]
5	lncRNA	99830241	99863867	ENSMUSG00000089743	MGI:3801792	Gm16226	predicted gene 16226 [Source:MGI Symbol;Acc:MGI:3801792]

5	lncRNA	99862493	99869647	ENSMUSG00000140204	MGI:7829064	Gm73153	predicted gene, 73153 [Source:MGI Symbol;Acc:MGI:7829064]
5	lncRNA	99878688	99902070	ENSMUSG00000105218	MGI:5621298	Gm38413	predicted gene, 38413 [Source:MGI Symbol;Acc:MGI:5621298]
5	lncRNA	1.00E+08	1.00E+08	ENSMUSG00000132178	MGI:7826730	Gm71982	predicted gene, 71982 [Source:MGI Symbol;Acc:MGI:7826730]
5	lncRNA	1.00E+08	1.00E+08	ENSMUSG00000141515	MGI:7829396	Gm73319	predicted gene, 73319 [Source:MGI Symbol;Acc:MGI:7829396]
5	lncRNA	1.00E+08	1.00E+08	ENSMUSG00000091623	MGI:4937919	Gm17092	predicted gene 17092 [Source:MGI Symbol;Acc:MGI:4937919]
5	lncRNA	1.00E+08	1.00E+08	ENSMUSG00000126730	MGI:7825749	Gm71488	predicted gene, 71488 [Source:MGI Symbol;Acc:MGI:7825749]
5	lncRNA	1.00E+08	1.00E+08	ENSMUSG00000140370	MGI:7829108	Gm73175	predicted gene, 73175 [Source:MGI Symbol;Acc:MGI:7829108]
5	lncRNA	1.00E+08	1.00E+08	ENSMUSG00000086259	MGI:1923253	Tmem150cos	transmembrane protein 150C, opposite strand [Source:MGI Symbol;Acc:MGI:1923253]
5	lncRNA	1.00E+08	1.00E+08	ENSMUSG00000136233	MGI:7827896	Gm72566	predicted gene, 72566 [Source:MGI Symbol;Acc:MGI:7827896]
5	lncRNA	1.00E+08	1.00E+08	ENSMUSG00000136314	MGI:7827924	Gm72581	predicted gene, 72581 [Source:MGI Symbol;Acc:MGI:7827924]
5	lncRNA	1.00E+08	1.00E+08	ENSMUSG00000100157	MGI:1916821	2310034O05Rik	RIKEN cDNA 2310034O05 gene [Source:MGI Symbol;Acc:MGI:1916821]
5	lncRNA	1.00E+08	1.00E+08	ENSMUSG00000144029	MGI:7830096	Gm73672	predicted gene, 73672 [Source:MGI Symbol;Acc:MGI:7830096]
5	lncRNA	1.00E+08	1.00E+08	ENSMUSG00000121065	MGI:7338529	Gm57308	predicted gene, 57308 [Source:MGI Symbol;Acc:MGI:7338529]
5	lncRNA	1.00E+08	1.00E+08	ENSMUSG00000130915			novel transcript
5	lncRNA	1.00E+08	1.00E+08	ENSMUSG00000131020	MGI:7826350	Gm71792	predicted gene, 71792 [Source:MGI Symbol;Acc:MGI:7826350]
5	lncRNA	1.00E+08	1.00E+08	ENSMUSG00000131094	MGI:5625013	Gm42128	predicted gene, 42128 [Source:MGI Symbol;Acc:MGI:5625013]
5	lncRNA	1.00E+08	1.00E+08	ENSMUSG00000130982	MGI:7826346	Gm71790	predicted gene, 71790 [Source:MGI Symbol;Acc:MGI:7826346]
5	lncRNA	1.00E+08	1.00E+08	ENSMUSG00000130947	MGI:5825550	Gm45913	predicted gene, 45913 [Source:MGI Symbol;Acc:MGI:5825550]

5	lncRNA	1.00E+08	1.00E+08	ENSMUSG00000131052	MGI:7826364	Gm71799	predicted gene, 71799 [Source:MGI Symbol;Acc:MGI:7826364]
5	lncRNA	1.01E+08	1.01E+08	ENSMUSG00000138356	MGI:7828568	Gm72904	predicted gene, 72904 [Source:MGI Symbol;Acc:MGI:7828568]
5	lncRNA	1.01E+08	1.01E+08	ENSMUSG00000138317	MGI:7828556	Gm72898	predicted gene, 72898 [Source:MGI Symbol;Acc:MGI:7828556]
5	lncRNA	1.01E+08	1.01E+08	ENSMUSG00000097772	MGI:1918676	5430416N02Rik	RIKEN cDNA 5430416N02 gene [Source:MGI Symbol;Acc:MGI:1918676]
5	lncRNA	1.01E+08	1.01E+08	ENSMUSG00000131639	MGI:7826588	Gm71911	predicted gene, 71911 [Source:MGI Symbol;Acc:MGI:7826588]
5	lncRNA	1.01E+08	1.01E+08	ENSMUSG00000131182	MGI:7826424	Gm71829	predicted gene, 71829 [Source:MGI Symbol;Acc:MGI:7826424]
5	lncRNA	1.01E+08	1.01E+08	ENSMUSG00000131120	MGI:7826404	Gm71819	predicted gene, 71819 [Source:MGI Symbol;Acc:MGI:7826404]
5	lncRNA	1.01E+08	1.01E+08	ENSMUSG00000131155	MGI:7830460	Gm73858	predicted gene, 73858 [Source:MGI Symbol;Acc:MGI:7830460]
5	lncRNA	1.01E+08	1.01E+08	ENSMUSG00000085269	MGI:3783219	Gm15777	predicted gene 15777 [Source:MGI Symbol;Acc:MGI:3783219]
5	lncRNA	1.01E+08	1.01E+08	ENSMUSG00000141768	MGI:7829444	Gm73343	predicted gene, 73343 [Source:MGI Symbol;Acc:MGI:7829444]
5	lncRNA	1.01E+08	1.01E+08	ENSMUSG00000141699	MGI:5595778	Gm36619	predicted gene, 36619 [Source:MGI Symbol;Acc:MGI:5595778]
5	lncRNA	1.01E+08	1.01E+08	ENSMUSG00000105755	MGI:5663651	Gm43514	predicted gene 43514 [Source:MGI Symbol;Acc:MGI:5663651]
5	lncRNA	1.01E+08	1.01E+08	ENSMUSG00000087611	MGI:1923052	4930458D05Rik	RIKEN cDNA 4930458D05 gene [Source:MGI Symbol;Acc:MGI:1923052]
5	lncRNA	1.01E+08	1.01E+08	ENSMUSG00000141832	MGI:7829460	Gm73351	predicted gene, 73351 [Source:MGI Symbol;Acc:MGI:7829460]
5	lncRNA	1.01E+08	1.01E+08	ENSMUSG00000106255	MGI:5595952	Gm36793	predicted gene, 36793 [Source:MGI Symbol;Acc:MGI:5595952]
5	lncRNA	1.01E+08	1.01E+08	ENSMUSG00000122123	MGI:5595899	Gm36740	predicted gene, 36740 [Source:MGI Symbol;Acc:MGI:5595899]
5	lncRNA	1.01E+08	1.01E+08	ENSMUSG00000126729	MGI:7825747	Gm71487	predicted gene, 71487 [Source:MGI Symbol;Acc:MGI:7825747]
5	lncRNA	1.01E+08	1.01E+08	ENSMUSG00000128974	MGI:7825943	Gm71585	predicted gene, 71585 [Source:MGI Symbol;Acc:MGI:7825943]

5	lncRNA	1.01E+08	1.01E+08	ENSMUSG00000136829	MGI:7828182	Gm72710	predicted gene, 72710 [Source:MGI Symbol;Acc:MGI:7828182]
5	lncRNA	1.01E+08	1.01E+08	ENSMUSG00000124318	MGI:7825517	Gm71371	predicted gene, 71371 [Source:MGI Symbol;Acc:MGI:7825517]
5	lncRNA	1.01E+08	1.01E+08	ENSMUSG00000129567	MGI:7825991	Gm71610	predicted gene, 71610 [Source:MGI Symbol;Acc:MGI:7825991]
5	lncRNA	1.01E+08	1.01E+08	ENSMUSG00000142499	MGI:5596010	Gm36851	predicted gene, 36851 [Source:MGI Symbol;Acc:MGI:5596010]
5	lncRNA	1.02E+08	1.02E+08	ENSMUSG00000143141	MGI:5625016	Gm42131	predicted gene, 42131 [Source:MGI Symbol;Acc:MGI:5625016]
5	lncRNA	1.02E+08	1.02E+08	ENSMUSG00000133793	MGI:5589141	Gm29982	predicted gene, 29982 [Source:MGI Symbol;Acc:MGI:5589141]
5	lncRNA	1.02E+08	1.02E+08	ENSMUSG00000133836	MGI:7827232	Gm72233	predicted gene, 72233 [Source:MGI Symbol;Acc:MGI:7827232]
5	lncRNA	1.02E+08	1.02E+08	ENSMUSG00000092503	MGI:5141949	Gm20484	predicted gene 20484 [Source:MGI Symbol;Acc:MGI:5141949]
5	lncRNA	1.02E+08	1.02E+08	ENSMUSG00000139393	MGI:7828862	Gm73052	predicted gene, 73052 [Source:MGI Symbol;Acc:MGI:7828862]
5	lncRNA	1.02E+08	1.02E+08	ENSMUSG00000105097	MGI:5625018	Gm42133	predicted gene, 42133 [Source:MGI Symbol;Acc:MGI:5625018]
5	lncRNA	1.02E+08	1.02E+08	ENSMUSG00000106164	MGI:1924611	9430085M18Rik	RIKEN cDNA 9430085M18 gene [Source:MGI Symbol;Acc:MGI:1924611]
5	lncRNA	1.02E+08	1.02E+08	ENSMUSG00000122648	MGI:5625019	Gm42134	predicted gene, 42134 [Source:MGI Symbol;Acc:MGI:5625019]
5	lncRNA	1.02E+08	1.02E+08	ENSMUSG00000092354	MGI:5142013	Gm20548	predicted gene 20548 [Source:MGI Symbol;Acc:MGI:5142013]
5	lncRNA	1.02E+08	1.02E+08	ENSMUSG00000131745	MGI:7826618	Gm71926	predicted gene, 71926 [Source:MGI Symbol;Acc:MGI:7826618]
5	lncRNA	1.02E+08	1.02E+08	ENSMUSG00000123489	MGI:7825409	Gm71316	predicted gene, 71316 [Source:MGI Symbol;Acc:MGI:7825409]
5	lncRNA	1.02E+08	1.02E+08	ENSMUSG00000132130	MGI:7826722	Gm71978	predicted gene, 71978 [Source:MGI Symbol;Acc:MGI:7826722]
5	lncRNA	1.02E+08	1.02E+08	ENSMUSG00000128755	MGI:5625020	Gm42135	predicted gene, 42135 [Source:MGI Symbol;Acc:MGI:5625020]
5	lncRNA	1.02E+08	1.02E+08	ENSMUSG00000106492	MGI:5588866	Gm29707	predicted gene, 29707 [Source:MGI Symbol;Acc:MGI:5588866]

5	lncRNA	1.02E+08	1.02E+08	ENSMUSG00000126276	MGI:7825705	Gm71466	predicted gene, 71466 [Source:MGI Symbol;Acc:MGI:7825705]
5	lncRNA	1.02E+08	1.02E+08	ENSMUSG00000138981	MGI:5625021	Gm42136	predicted gene, 42136 [Source:MGI Symbol;Acc:MGI:5625021]
5	lncRNA	1.02E+08	1.02E+08	ENSMUSG00000133687	MGI:5826584	Gm46947	predicted gene, 46947 [Source:MGI Symbol;Acc:MGI:5826584]
5	lncRNA	1.02E+08	1.02E+08	ENSMUSG00000139331	MGI:7828844	Gm73043	predicted gene, 73043 [Source:MGI Symbol;Acc:MGI:7828844]
5	lncRNA	1.02E+08	1.02E+08	ENSMUSG00000137432	MGI:7828396	Gm72817	predicted gene, 72817 [Source:MGI Symbol;Acc:MGI:7828396]
5	lncRNA	1.02E+08	1.02E+08	ENSMUSG00000132860	MGI:7826870	Gm72052	predicted gene, 72052 [Source:MGI Symbol;Acc:MGI:7826870]
5	lncRNA	1.03E+08	1.03E+08	ENSMUSG00000123014	MGI:7825346	Gm71284	predicted gene, 71284 [Source:MGI Symbol;Acc:MGI:7825346]
5	lncRNA	1.03E+08	1.03E+08	ENSMUSG00000123054	MGI:7825350	Gm71286	predicted gene, 71286 [Source:MGI Symbol;Acc:MGI:7825350]
5	lncRNA	1.03E+08	1.03E+08	ENSMUSG00000106586	MGI:1922755	1700013M08Rik	RIKEN cDNA 1700013M08 gene [Source:MGI Symbol;Acc:MGI:1922755]
5	lncRNA	1.03E+08	1.03E+08	ENSMUSG00000143028	MGI:5589555	Gm30396	predicted gene, 30396 [Source:MGI Symbol;Acc:MGI:5589555]
5	lncRNA	1.03E+08	1.03E+08	ENSMUSG00000143109	MGI:7829732	Gm73488	predicted gene, 73488 [Source:MGI Symbol;Acc:MGI:7829732]
5	lncRNA	1.03E+08	1.03E+08	ENSMUSG00000143146	MGI:7829736	Gm73490	predicted gene, 73490 [Source:MGI Symbol;Acc:MGI:7829736]
5	lncRNA	1.03E+08	1.03E+08	ENSMUSG00000122328	MGI:7825251	Gm71235	predicted gene, 71235 [Source:MGI Symbol;Acc:MGI:7825251]
5	lncRNA	1.03E+08	1.04E+08	ENSMUSG00000104956	MGI:1921874	4930429D17Rik	RIKEN cDNA 4930429D17 gene [Source:MGI Symbol;Acc:MGI:1921874]
5	lncRNA	1.03E+08	1.03E+08	ENSMUSG00000105426	MGI:1922769	1700021F02Rik	RIKEN cDNA 1700021F02 gene [Source:MGI Symbol;Acc:MGI:1922769]
5	lncRNA	1.04E+08	1.04E+08	ENSMUSG00000104684	MGI:2441699	5430427N15Rik	RIKEN cDNA 5430427N15 gene [Source:MGI Symbol;Acc:MGI:2441699]
5	lncRNA	1.04E+08	1.04E+08	ENSMUSG00000140589	MGI:5589602	Gm30443	predicted gene, 30443 [Source:MGI Symbol;Acc:MGI:5589602]
5	lncRNA	1.04E+08	1.04E+08	ENSMUSG00000141609	MGI:7829404	Gm73323	predicted gene, 73323 [Source:MGI Symbol;Acc:MGI:7829404]

5	lncRNA	1.04E+08	1.04E+08	ENSMUSG00000104580	MGI:5662757	Gm42620	predicted gene 42620 [Source:MGI Symbol;Acc:MGI:5662757]
5	lncRNA	1.04E+08	1.04E+08	ENSMUSG00000136783	MGI:5625023	Gm42138	predicted gene, 42138 [Source:MGI Symbol;Acc:MGI:5625023]
5	lncRNA	1.04E+08	1.04E+08	ENSMUSG00000122606	MGI:7825279	Gm71249	predicted gene, 71249 [Source:MGI Symbol;Acc:MGI:7825279]
5	lncRNA	1.04E+08	1.04E+08	ENSMUSG00000142430	MGI:7829570	Gm73406	predicted gene, 73406 [Source:MGI Symbol;Acc:MGI:7829570]
5	lncRNA	1.04E+08	1.04E+08	ENSMUSG00000142388	MGI:7829564	Gm73403	predicted gene, 73403 [Source:MGI Symbol;Acc:MGI:7829564]
5	lncRNA	1.04E+08	1.04E+08	ENSMUSG00000130284	MGI:7826190	Gm71711	predicted gene, 71711 [Source:MGI Symbol;Acc:MGI:7826190]
5	lncRNA	1.04E+08	1.04E+08	ENSMUSG00000140976	MGI:6367177	Gm52783	predicted gene, 52783 [Source:MGI Symbol;Acc:MGI:6367177]
5	lncRNA	1.04E+08	1.04E+08	ENSMUSG00000139941	MGI:7828976	Gm73109	predicted gene, 73109 [Source:MGI Symbol;Acc:MGI:7828976]
5	lncRNA	1.04E+08	1.04E+08	ENSMUSG00000097877	MGI:5477197	Gm26703	predicted gene, 26703 [Source:MGI Symbol;Acc:MGI:5477197]
5	lncRNA	1.04E+08	1.04E+08	ENSMUSG00000105615	MGI:5590207	Gm31048	predicted gene, 31048 [Source:MGI Symbol;Acc:MGI:5590207]
5	lncRNA	1.04E+08	1.04E+08	ENSMUSG00000130229	MGI:7826166	Gm71699	predicted gene, 71699 [Source:MGI Symbol;Acc:MGI:7826166]
5	lncRNA	1.04E+08	1.05E+08	ENSMUSG00000128790	MGI:7825931	Gm71579	predicted gene, 71579 [Source:MGI Symbol;Acc:MGI:7825931]
5	lncRNA	1.05E+08	1.05E+08	ENSMUSG00000130295	MGI:7826192	Gm71712	predicted gene, 71712 [Source:MGI Symbol;Acc:MGI:7826192]
5	lncRNA	1.05E+08	1.05E+08	ENSMUSG00000124296	MGI:7825511	Gm71368	predicted gene, 71368 [Source:MGI Symbol;Acc:MGI:7825511]
5	lncRNA	1.05E+08	1.05E+08	ENSMUSG00000124334	MGI:7825521	Gm71373	predicted gene, 71373 [Source:MGI Symbol;Acc:MGI:7825521]
5	lncRNA	1.05E+08	1.05E+08	ENSMUSG00000122026	MGI:7825199	Gm71209	predicted gene, 71209 [Source:MGI Symbol;Acc:MGI:7825199]
5	lncRNA	1.05E+08	1.05E+08	ENSMUSG00000140668	MGI:7829220	Gm73231	predicted gene, 73231 [Source:MGI Symbol;Acc:MGI:7829220]
5	lncRNA	1.05E+08	1.05E+08	ENSMUSG00000134138	MGI:5590414	Gm31255	predicted gene, 31255 [Source:MGI Symbol;Acc:MGI:5590414]

5	lncRNA	1.05E+08	1.05E+08	ENSMUSG00000134259	MGI:7827428	Gm72331	predicted gene, 72331 [Source:MGI Symbol;Acc:MGI:7827428]
5	lncRNA	1.05E+08	1.05E+08	ENSMUSG00000139462	MGI:7828906	Gm73074	predicted gene, 73074 [Source:MGI Symbol;Acc:MGI:7828906]
5	lncRNA	1.05E+08	1.05E+08	ENSMUSG00000134102	MGI:7827352	Gm72293	predicted gene, 72293 [Source:MGI Symbol;Acc:MGI:7827352]
5	lncRNA	1.05E+08	1.05E+08	ENSMUSG00000132192	MGI:7826734	Gm71984	predicted gene, 71984 [Source:MGI Symbol;Acc:MGI:7826734]
5	lncRNA	1.05E+08	1.05E+08	ENSMUSG00000119978	MGI:5590944	Gm31785	predicted gene, 31785 [Source:MGI Symbol;Acc:MGI:5590944]
5	lncRNA	1.06E+08	1.06E+08	ENSMUSG00000134940	MGI:7827556	Gm72395	predicted gene, 72395 [Source:MGI Symbol;Acc:MGI:7827556]
5	lncRNA	1.06E+08	1.06E+08	ENSMUSG00000106438	MGI:5591210	Gm32051	predicted gene, 32051 [Source:MGI Symbol;Acc:MGI:5591210]
5	lncRNA	1.06E+08	1.06E+08	ENSMUSG00000135055	MGI:7827596	Gm72415	predicted gene, 72415 [Source:MGI Symbol;Acc:MGI:7827596]
5	lncRNA	1.06E+08	1.06E+08	ENSMUSG00000135020	MGI:1923077	4930542N06Rik	RIKEN cDNA 4930542N06 gene [Source:MGI Symbol;Acc:MGI:1923077]
5	lncRNA	1.06E+08	1.06E+08	ENSMUSG00000126067			novel transcript
5	lncRNA	1.06E+08	1.06E+08	ENSMUSG00000126110	MGI:7825683	Gm71455	predicted gene, 71455 [Source:MGI Symbol;Acc:MGI:7825683]
5	lncRNA	1.06E+08	1.06E+08	ENSMUSG00000133270	MGI:7827092	Gm72163	predicted gene, 72163 [Source:MGI Symbol;Acc:MGI:7827092]
5	lncRNA	1.06E+08	1.06E+08	ENSMUSG00000133188	MGI:7827012	Gm72123	predicted gene, 72123 [Source:MGI Symbol;Acc:MGI:7827012]
5	lncRNA	1.06E+08	1.06E+08	ENSMUSG00000133309	MGI:7827098	Gm72166	predicted gene, 72166 [Source:MGI Symbol;Acc:MGI:7827098]
5	lncRNA	1.06E+08	1.06E+08	ENSMUSG00000133225	MGI:7827076	Gm72155	predicted gene, 72155 [Source:MGI Symbol;Acc:MGI:7827076]
5	lncRNA	1.06E+08	1.06E+08	ENSMUSG00000085771	MGI:2141339	C230066G23Rik	RIKEN cDNA C230066G23 gene [Source:MGI Symbol;Acc:MGI:2141339]
5	lncRNA	1.06E+08	1.06E+08	ENSMUSG00000143828	MGI:7831190	Gm74226	predicted gene, 74226 [Source:MGI Symbol;Acc:MGI:7831190]
5	lncRNA	1.06E+08	1.06E+08	ENSMUSG00000143792	MGI:7829970	Gm73607	predicted gene, 73607 [Source:MGI Symbol;Acc:MGI:7829970]

5	lncRNA	1.06E+08	1.06E+08	ENSMUSG00000131937	MGI:5591389	Gm32230	predicted gene, 32230 [Source:MGI Symbol;Acc:MGI:5591389]
5	lncRNA	1.06E+08	1.06E+08	ENSMUSG00000120245	MGI:7338425	Gm57256	predicted gene, 57256 [Source:MGI Symbol;Acc:MGI:7338425]
5	lncRNA	1.06E+08	1.06E+08	ENSMUSG00000126152	MGI:7831156	Gm74209	predicted gene, 74209 [Source:MGI Symbol;Acc:MGI:7831156]
5	lncRNA	1.06E+08	1.06E+08	ENSMUSG00000121168	MGI:7338559	Gm57323	predicted gene, 57323 [Source:MGI Symbol;Acc:MGI:7338559]
5	lncRNA	1.06E+08	1.06E+08	ENSMUSG00000126262	MGI:7825699	Gm71463	predicted gene, 71463 [Source:MGI Symbol;Acc:MGI:7825699]
5	lncRNA	1.06E+08	1.06E+08	ENSMUSG00000084878	MGI:3802116	Lrrc8dos	leucine rich repeat containing 8D, opposite strand [Source:MGI Symbol;Acc:MGI:3802116]
5	lncRNA	1.06E+08	1.06E+08	ENSMUSG00000126228	MGI:7825691	Gm71459	predicted gene, 71459 [Source:MGI Symbol;Acc:MGI:7825691]
5	lncRNA	1.06E+08	1.06E+08	ENSMUSG00000123641	MGI:7825425	Gm71324	predicted gene, 71324 [Source:MGI Symbol;Acc:MGI:7825425]
5	lncRNA	1.06E+08	1.06E+08	ENSMUSG00000123599	MGI:7825421	Gm71322	predicted gene, 71322 [Source:MGI Symbol;Acc:MGI:7825421]
5	lncRNA	1.06E+08	1.06E+08	ENSMUSG00000123655	MGI:7825427	Gm71325	predicted gene, 71325 [Source:MGI Symbol;Acc:MGI:7825427]
5	lncRNA	1.06E+08	1.06E+08	ENSMUSG00000106483	MGI:5591895	Gm32736	predicted gene, 32736 [Source:MGI Symbol;Acc:MGI:5591895]
5	lncRNA	1.06E+08	1.06E+08	ENSMUSG00000126268	MGI:7825703	Gm71465	predicted gene, 71465 [Source:MGI Symbol;Acc:MGI:7825703]
5	lncRNA	1.06E+08	1.07E+08	ENSMUSG00000106594	MGI:1921031	4930432H08Rik	RIKEN cDNA 4930432H08 gene [Source:MGI Symbol;Acc:MGI:1921031]
5	lncRNA	1.06E+08	1.06E+08	ENSMUSG00000135775	MGI:7827772	Gm72504	predicted gene, 72504 [Source:MGI Symbol;Acc:MGI:7827772]
5	lncRNA	1.06E+08	1.06E+08	ENSMUSG00000105508	MGI:5592080	Gm32921	predicted gene, 32921 [Source:MGI Symbol;Acc:MGI:5592080]
5	lncRNA	1.06E+08	1.07E+08	ENSMUSG00000105531	MGI:2685681	4930458A03Rik	RIKEN cDNA 4930458A03 gene [Source:MGI Symbol;Acc:MGI:2685681]
5	lncRNA	1.07E+08	1.07E+08	ENSMUSG00000128962	MGI:7825941	Gm71584	predicted gene, 71584 [Source:MGI Symbol;Acc:MGI:7825941]
5	lncRNA	1.07E+08	1.07E+08	ENSMUSG00000129002	MGI:7825947	Gm71587	predicted gene, 71587 [Source:MGI Symbol;Acc:MGI:7825947]

5	lncRNA	1.07E+08	1.07E+08	ENSMUSG00000120527	MGI:5592310	Gm33151	predicted gene, 33151 [Source:MGI Symbol;Acc:MGI:5592310]
5	lncRNA	1.07E+08	1.07E+08	ENSMUSG00000143832	MGI:7830010	Gm73627	predicted gene, 73627 [Source:MGI Symbol;Acc:MGI:7830010]
5	lncRNA	1.07E+08	1.07E+08	ENSMUSG00000097018	MGI:5477366	Gm26872	predicted gene, 26872 [Source:MGI Symbol;Acc:MGI:5477366]
5	lncRNA	1.07E+08	1.07E+08	ENSMUSG00000130270	MGI:7826176	Gm71704	predicted gene, 71704 [Source:MGI Symbol;Acc:MGI:7826176]
5	lncRNA	1.07E+08	1.07E+08	ENSMUSG00000099061	MGI:5547786	Gm28050	predicted gene, 28050 [Source:MGI Symbol;Acc:MGI:5547786]
5	lncRNA	1.07E+08	1.07E+08	ENSMUSG00000131939			novel transcript
5	lncRNA	1.07E+08	1.07E+08	ENSMUSG00000098302	MGI:5547775	Gm28039	predicted gene, 28039 [Source:MGI Symbol;Acc:MGI:5547775]
5	lncRNA	1.07E+08	1.07E+08	ENSMUSG00000101237	MGI:5580170	Gm29464	predicted gene 29464 [Source:MGI Symbol;Acc:MGI:5580170]
5	lncRNA	1.07E+08	1.07E+08	ENSMUSG00000131617	MGI:7826584	Gm71909	predicted gene, 71909 [Source:MGI Symbol;Acc:MGI:7826584]
5	lncRNA	1.07E+08	1.07E+08	ENSMUSG00000126214	MGI:5592486	Gm33327	predicted gene, 33327 [Source:MGI Symbol;Acc:MGI:5592486]
5	lncRNA	1.07E+08	1.07E+08	ENSMUSG00000140743	MGI:7829226	Gm73234	predicted gene, 73234 [Source:MGI Symbol;Acc:MGI:7829226]
5	lncRNA	1.07E+08	1.07E+08	ENSMUSG00000136976	MGI:7828246	Gm72742	predicted gene, 72742 [Source:MGI Symbol;Acc:MGI:7828246]
5	lncRNA	1.07E+08	1.07E+08	ENSMUSG00000124898	MGI:7825571	Gm71399	predicted gene, 71399 [Source:MGI Symbol;Acc:MGI:7825571]
5	lncRNA	1.07E+08	1.07E+08	ENSMUSG00000134103	MGI:5592530	Gm33371	predicted gene, 33371 [Source:MGI Symbol;Acc:MGI:5592530]
5	lncRNA	1.07E+08	1.07E+08	ENSMUSG00000106554	MGI:5592633	Gm33474	predicted gene, 33474 [Source:MGI Symbol;Acc:MGI:5592633]
5	lncRNA	1.07E+08	1.07E+08	ENSMUSG00000123782	MGI:5625031	Gm42146	predicted gene, 42146 [Source:MGI Symbol;Acc:MGI:5625031]
5	lncRNA	1.07E+08	1.07E+08	ENSMUSG00000138859	MGI:7828740	Gm72990	predicted gene, 72990 [Source:MGI Symbol;Acc:MGI:7828740]
5	lncRNA	1.07E+08	1.07E+08	ENSMUSG00000132945	MGI:7826894	Gm72064	predicted gene, 72064 [Source:MGI Symbol;Acc:MGI:7826894]

5	lncRNA	1.08E+08	1.08E+08	ENSMUSG00000125872	MGI:7825661	Gm71444	predicted gene, 71444 [Source:MGI Symbol;Acc:MGI:7825661]
5	lncRNA	1.08E+08	1.08E+08	ENSMUSG00000090558	MGI:4938029	Gm17202	predicted gene 17202 [Source:MGI Symbol;Acc:MGI:4938029]
5	lncRNA	1.08E+08	1.08E+08	ENSMUSG00000125909	MGI:7825665	Gm71446	predicted gene, 71446 [Source:MGI Symbol;Acc:MGI:7825665]
5	lncRNA	1.08E+08	1.08E+08	ENSMUSG00000132608	MGI:7826808	Gm72021	predicted gene, 72021 [Source:MGI Symbol;Acc:MGI:7826808]
5	lncRNA	1.08E+08	1.08E+08	ENSMUSG00000134553	MGI:5593044	Gm33885	predicted gene, 33885 [Source:MGI Symbol;Acc:MGI:5593044]
5	lncRNA	1.08E+08	1.08E+08	ENSMUSG00000085900	MGI:1925693	A930041C12Rik	RIKEN cDNA A930041C12 gene [Source:MGI Symbol;Acc:MGI:1925693]
5	lncRNA	1.08E+08	1.08E+08	ENSMUSG00000133374	MGI:7827138	Gm72186	predicted gene, 72186 [Source:MGI Symbol;Acc:MGI:7827138]
5	lncRNA	1.08E+08	1.08E+08	ENSMUSG00000106333	MGI:1923034	4930428O21Rik	RIKEN cDNA 4930428O21 gene [Source:MGI Symbol;Acc:MGI:1923034]
5	lncRNA	1.08E+08	1.08E+08	ENSMUSG00000090030	MGI:3041214	A430072P03Rik	RIKEN cDNA A430072P03 gene [Source:MGI Symbol;Acc:MGI:3041214]
5	lncRNA	1.08E+08	1.08E+08	ENSMUSG00000123337	MGI:7825391	Gm71307	predicted gene, 71307 [Source:MGI Symbol;Acc:MGI:7825391]
5	lncRNA	1.08E+08	1.08E+08	ENSMUSG00000137433	MGI:7828406	Gm72822	predicted gene, 72822 [Source:MGI Symbol;Acc:MGI:7828406]
5	lncRNA	1.08E+08	1.08E+08	ENSMUSG00000137507	MGI:7828436	Gm72837	predicted gene, 72837 [Source:MGI Symbol;Acc:MGI:7828436]
5	lncRNA	1.08E+08	1.08E+08	ENSMUSG00000137388	MGI:7830274	Gm73762	predicted gene, 73762 [Source:MGI Symbol;Acc:MGI:7830274]
5	lncRNA	1.08E+08	1.08E+08	ENSMUSG00000141797	MGI:7829452	Gm73347	predicted gene, 73347 [Source:MGI Symbol;Acc:MGI:7829452]
5	lncRNA	1.08E+08	1.08E+08	ENSMUSG00000123424	MGI:7830606	Gm73931	predicted gene, 73931 [Source:MGI Symbol;Acc:MGI:7830606]
5	lncRNA	1.09E+08	1.09E+08	ENSMUSG00000104904	MGI:1924810	9330198I05Rik	RIKEN cDNA 9330198I05 gene [Source:MGI Symbol;Acc:MGI:1924810]
5	lncRNA	1.09E+08	1.09E+08	ENSMUSG00000106304	MGI:5662655	Gm42518	predicted gene 42518 [Source:MGI Symbol;Acc:MGI:5662655]
5	lncRNA	1.09E+08	1.09E+08	ENSMUSG00000072769	MGI:3642823	Gm10419	predicted gene 10419 [Source:MGI Symbol;Acc:MGI:3642823]

5	lncRNA	1.09E+08	1.09E+08	ENSMUSG00000120483	MGI:7338441	Gm57264	predicted gene, 57264 [Source:MGI Symbol;Acc:MGI:7338441]
5	lncRNA	1.09E+08	1.09E+08	ENSMUSG00000121853	MGI:7830664	Gm73960	predicted gene, 73960 [Source:MGI Symbol;Acc:MGI:7830664]
5	lncRNA	1.09E+08	1.09E+08	ENSMUSG00000128538	MGI:7830734	Gm73995	predicted gene, 73995 [Source:MGI Symbol;Acc:MGI:7830734]
5	lncRNA	1.09E+08	1.09E+08	ENSMUSG00000142352	MGI:7830736	Gm73996	predicted gene, 73996 [Source:MGI Symbol;Acc:MGI:7830736]
5	lncRNA	1.09E+08	1.09E+08	ENSMUSG00000133269	MGI:7830740	Gm73998	predicted gene, 73998 [Source:MGI Symbol;Acc:MGI:7830740]
5	lncRNA	1.09E+08	1.09E+08	ENSMUSG00000133224	MGI:7827074	Gm72154	predicted gene, 72154 [Source:MGI Symbol;Acc:MGI:7827074]
5	lncRNA	1.09E+08	1.09E+08	ENSMUSG00000138567	MGI:5625036	Gm42151	predicted gene, 42151 [Source:MGI Symbol;Acc:MGI:5625036]
5	lncRNA	1.09E+08	1.09E+08	ENSMUSG00000101762	MGI:1920545	1700047L14Rik	RIKEN cDNA 1700047L14 gene [Source:MGI Symbol;Acc:MGI:1920545]
5	lncRNA	1.09E+08	1.09E+08	ENSMUSG00000093479	MGI:5313076	Gm20629	predicted gene 20629 [Source:MGI Symbol;Acc:MGI:5313076]
5	lncRNA	1.09E+08	1.09E+08	ENSMUSG00000129648	MGI:7826009	Gm71619	predicted gene, 71619 [Source:MGI Symbol;Acc:MGI:7826009]
5	lncRNA	1.10E+08	1.10E+08	ENSMUSG00000124061	MGI:6364910	Gm50598	predicted gene, 50598 [Source:MGI Symbol;Acc:MGI:6364910]
5	lncRNA	1.10E+08	1.10E+08	ENSMUSG00000134481	MGI:7827446	Gm72340	predicted gene, 72340 [Source:MGI Symbol;Acc:MGI:7827446]
5	lncRNA	1.10E+08	1.10E+08	ENSMUSG00000136256	MGI:7827908	Gm72573	predicted gene, 72573 [Source:MGI Symbol;Acc:MGI:7827908]
5	lncRNA	1.10E+08	1.10E+08	ENSMUSG00000136293	MGI:7830534	Gm73895	predicted gene, 73895 [Source:MGI Symbol;Acc:MGI:7830534]
5	lncRNA	1.10E+08	1.10E+08	ENSMUSG00000121595			RIKEN cDNA 4930522L14 gene [Source:NCBI gene (formerly Entrezgene);Acc:100041734]
5	lncRNA	1.10E+08	1.10E+08	ENSMUSG00000120227	MGI:7338503	Gm57295	predicted gene, 57295 [Source:MGI Symbol;Acc:MGI:7338503]
5	lncRNA	1.10E+08	1.10E+08	ENSMUSG00000133173	MGI:5594248	Gm35089	predicted gene, 35089 [Source:MGI Symbol;Acc:MGI:5594248]
5	lncRNA	1.10E+08	1.10E+08	ENSMUSG00000097140	MGI:5477273	Gm26779	predicted gene, 26779 [Source:MGI Symbol;Acc:MGI:5477273]

5	lncRNA	1.10E+08	1.10E+08	ENSMUSG00000133215	MGI:5477302	Gm26808	predicted gene, 26808 [Source:MGI Symbol;Acc:MGI:5477302]
5	lncRNA	1.10E+08	1.10E+08	ENSMUSG00000097863	MGI:1922653	1010001B22Rik	RIKEN cDNA 1010001B22 gene [Source:MGI Symbol;Acc:MGI:1922653]
5	lncRNA	1.10E+08	1.10E+08	ENSMUSG00000123077	MGI:6723685	Gm54334	predicted gene, 54334 [Source:MGI Symbol;Acc:MGI:6723685]
5	lncRNA	1.10E+08	1.10E+08	ENSMUSG00000086247	MGI:3783229	Gm15787	predicted gene 15787 [Source:MGI Symbol;Acc:MGI:3783229]
5	lncRNA	1.10E+08	1.10E+08	ENSMUSG00000104720	MGI:5663275	Gm43138	predicted gene 43138 [Source:MGI Symbol;Acc:MGI:5663275]
5	lncRNA	1.10E+08	1.10E+08	ENSMUSG00000104970	MGI:3704253	5930411N13Rik	RIKEN cDNA 5930411N13 gene [Source:MGI Symbol;Acc:MGI:3704253]
5	lncRNA	1.10E+08	1.10E+08	ENSMUSG00000127072	MGI:7825785	Gm71506	predicted gene, 71506 [Source:MGI Symbol;Acc:MGI:7825785]
5	lncRNA	1.10E+08	1.10E+08	ENSMUSG00000087439	MGI:3783230	Gm15788	predicted gene 15788 [Source:MGI Symbol;Acc:MGI:3783230]
5	lncRNA	1.10E+08	1.10E+08	ENSMUSG00000085606	MGI:3783234	Gm15792	predicted gene 15792 [Source:MGI Symbol;Acc:MGI:3783234]
5	lncRNA	1.10E+08	1.10E+08	ENSMUSG00000132550	MGI:7826798	Gm72016	predicted gene, 72016 [Source:MGI Symbol;Acc:MGI:7826798]
5	lncRNA	1.11E+08	1.11E+08	ENSMUSG00000105482	MGI:5662733	Gm42596	predicted gene 42596 [Source:MGI Symbol;Acc:MGI:5662733]
5	lncRNA	1.11E+08	1.11E+08	ENSMUSG00000048215	MGI:2445162	A630023P12Rik	RIKEN cDNA A630023P12 gene [Source:MGI Symbol;Acc:MGI:2445162]
5	lncRNA	1.11E+08	1.11E+08	ENSMUSG00000138464	MGI:7828604	Gm72922	predicted gene, 72922 [Source:MGI Symbol;Acc:MGI:7828604]
5	lncRNA	1.11E+08	1.11E+08	ENSMUSG00000134164	MGI:7827390	Gm72312	predicted gene, 72312 [Source:MGI Symbol;Acc:MGI:7827390]
5	lncRNA	1.11E+08	1.11E+08	ENSMUSG00000105161	MGI:5662732	Gm42595	predicted gene 42595 [Source:MGI Symbol;Acc:MGI:5662732]
5	lncRNA	1.11E+08	1.11E+08	ENSMUSG00000086401	MGI:3783008	Gm15559	predicted gene 15559 [Source:MGI Symbol;Acc:MGI:3783008]
5	lncRNA	1.11E+08	1.11E+08	ENSMUSG00000128201	MGI:5477009	Gm26515	predicted gene, 26515 [Source:MGI Symbol;Acc:MGI:5477009]
5	lncRNA	1.11E+08	1.11E+08	ENSMUSG00000132579	MGI:7826804	Gm72019	predicted gene, 72019 [Source:MGI Symbol;Acc:MGI:7826804]

5	lncRNA	1.11E+08	1.11E+08	ENSMUSG00000131984	MGI:7826684	Gm71959	predicted gene, 71959 [Source:MGI Symbol;Acc:MGI:7826684]
5	lncRNA	1.11E+08	1.11E+08	ENSMUSG00000133651	MGI:7827166	Gm72200	predicted gene, 72200 [Source:MGI Symbol;Acc:MGI:7827166]
5	lncRNA	1.11E+08	1.11E+08	ENSMUSG00000140868	MGI:5594787	Gm35628	predicted gene, 35628 [Source:MGI Symbol;Acc:MGI:5594787]
5	lncRNA	1.11E+08	1.11E+08	ENSMUSG00000141983	MGI:7829474	Gm73358	predicted gene, 73358 [Source:MGI Symbol;Acc:MGI:7829474]
5	lncRNA	1.12E+08	1.12E+08	ENSMUSG00000104559	MGI:5663255	Gm43118	predicted gene 43118 [Source:MGI Symbol;Acc:MGI:5663255]
5	lncRNA	1.12E+08	1.12E+08	ENSMUSG00000120465	MGI:7338437	Gm57262	predicted gene, 57262 [Source:MGI Symbol;Acc:MGI:7338437]
5	lncRNA	1.12E+08	1.12E+08	ENSMUSG00000132416	MGI:7826740	Gm71987	predicted gene, 71987 [Source:MGI Symbol;Acc:MGI:7826740]
5	lncRNA	1.12E+08	1.12E+08	ENSMUSG00000141147	MGI:5625040	Gm42155	predicted gene, 42155 [Source:MGI Symbol;Acc:MGI:5625040]
5	lncRNA	1.12E+08	1.12E+08	ENSMUSG00000123711	MGI:5625039	Gm42154	predicted gene, 42154 [Source:MGI Symbol;Acc:MGI:5625039]
5	lncRNA	1.12E+08	1.12E+08	ENSMUSG00000144040	MGI:7830108	Gm73679	predicted gene, 73679 [Source:MGI Symbol;Acc:MGI:7830108]
5	lncRNA	1.12E+08	1.12E+08	ENSMUSG00000120047	MGI:5595308	Cirpil	cardiac ischemia reperfusion associated p53 interacting lncRNA [Source:MGI Symbol;Acc:MGI:5595308]
5	lncRNA	1.12E+08	1.12E+08	ENSMUSG00000134002	MGI:5595382	Gm36223	predicted gene, 36223 [Source:MGI Symbol;Acc:MGI:5595382]
5	lncRNA	1.12E+08	1.12E+08	ENSMUSG00000141595	MGI:7829402	Gm73322	predicted gene, 73322 [Source:MGI Symbol;Acc:MGI:7829402]
5	lncRNA	1.12E+08	1.12E+08	ENSMUSG00000141634	MGI:7829414	Gm73328	predicted gene, 73328 [Source:MGI Symbol;Acc:MGI:7829414]
5	lncRNA	1.12E+08	1.12E+08	ENSMUSG00000134952	MGI:5595433	Gm36274	predicted gene, 36274 [Source:MGI Symbol;Acc:MGI:5595433]
5	lncRNA	1.12E+08	1.12E+08	ENSMUSG00000052848	MGI:3041189	C130026L21Rik	RIKEN cDNA C130026L21 gene [Source:MGI Symbol;Acc:MGI:3041189]
5	lncRNA	1.12E+08	1.12E+08	ENSMUSG00000104833	MGI:5662626	Gm42489	predicted gene 42489 [Source:MGI Symbol;Acc:MGI:5662626]

5	lncRNA	1.12E+08	1.12E+08	ENSMUSG00000125147	MGI:7825585	Gm71406	predicted gene, 71406 [Source:MGI Symbol;Acc:MGI:7825585]
5	lncRNA	1.12E+08	1.12E+08	ENSMUSG00000122221	MGI:5595545	Gm36386	predicted gene, 36386 [Source:MGI Symbol;Acc:MGI:5595545]
5	lncRNA	1.12E+08	1.12E+08	ENSMUSG00000122261	MGI:5595593	Gm36434	predicted gene, 36434 [Source:MGI Symbol;Acc:MGI:5595593]
5	lncRNA	1.12E+08	1.12E+08	ENSMUSG00000122182	MGI:7825227	Gm71223	predicted gene, 71223 [Source:MGI Symbol;Acc:MGI:7825227]
5	lncRNA	1.12E+08	1.12E+08	ENSMUSG00000123321	MGI:7825387	Gm71305	predicted gene, 71305 [Source:MGI Symbol;Acc:MGI:7825387]
5	lncRNA	1.12E+08	1.12E+08	ENSMUSG00000123283	MGI:7825383	Gm71303	predicted gene, 71303 [Source:MGI Symbol;Acc:MGI:7825383]
5	lncRNA	1.12E+08	1.12E+08	ENSMUSG00000123154	MGI:7825360	Gm71291	predicted gene, 71291 [Source:MGI Symbol;Acc:MGI:7825360]
5	lncRNA	1.12E+08	1.12E+08	ENSMUSG00000142550	MGI:7829624	Gm73434	predicted gene, 73434 [Source:MGI Symbol;Acc:MGI:7829624]
5	lncRNA	1.12E+08	1.12E+08	ENSMUSG00000128486	MGI:5595640	Gm36481	predicted gene, 36481 [Source:MGI Symbol;Acc:MGI:5595640]
5	lncRNA	1.12E+08	1.12E+08	ENSMUSG00000085919	MGI:3801872	Gm16019	predicted gene 16019 [Source:MGI Symbol;Acc:MGI:3801872]
5	lncRNA	1.12E+08	1.12E+08	ENSMUSG00000085271	MGI:2685527	E130006D01Rik	RIKEN cDNA E130006D01 gene [Source:MGI Symbol;Acc:MGI:2685527]
5	lncRNA	1.12E+08	1.12E+08	ENSMUSG00000105376	MGI:5595694	Gm36535	predicted gene, 36535 [Source:MGI Symbol;Acc:MGI:5595694]
5	lncRNA	1.12E+08	1.12E+08	ENSMUSG00000131805	MGI:7826634	Gm71934	predicted gene, 71934 [Source:MGI Symbol;Acc:MGI:7826634]
5	lncRNA	1.12E+08	1.12E+08	ENSMUSG00000141916	MGI:7829468	Gm73355	predicted gene, 73355 [Source:MGI Symbol;Acc:MGI:7829468]
5	lncRNA	1.12E+08	1.12E+08	ENSMUSG00000128353	MGI:5589094	Gm29935	predicted gene, 29935 [Source:MGI Symbol;Acc:MGI:5589094]
5	lncRNA	1.12E+08	1.12E+08	ENSMUSG00000106060	MGI:5662625	Gm42488	predicted gene 42488 [Source:MGI Symbol;Acc:MGI:5662625]
5	lncRNA	1.12E+08	1.12E+08	ENSMUSG00000128427	MGI:7825889	Gm71558	predicted gene, 71558 [Source:MGI Symbol;Acc:MGI:7825889]
5	lncRNA	1.12E+08	1.12E+08	ENSMUSG00000128317	MGI:5596039	Gm36880	predicted gene, 36880 [Source:MGI Symbol;Acc:MGI:5596039]

5	lncRNA	1.12E+08	1.12E+08	ENSMUSG00000128392	MGI:5625041	Gm42156	predicted gene, 42156 [Source:MGI Symbol;Acc:MGI:5625041]
5	lncRNA	1.12E+08	1.12E+08	ENSMUSG00000128275	MGI:7825869	Gm71548	predicted gene, 71548 [Source:MGI Symbol;Acc:MGI:7825869]
5	lncRNA	1.12E+08	1.12E+08	ENSMUSG00000128237	MGI:7825865	Gm71546	predicted gene, 71546 [Source:MGI Symbol;Acc:MGI:7825865]
5	lncRNA	1.12E+08	1.12E+08	ENSMUSG00000131045	MGI:5589191	Gm30032	predicted gene, 30032 [Source:MGI Symbol;Acc:MGI:5589191]
5	lncRNA	1.12E+08	1.12E+08	ENSMUSG00000131083	MGI:7826374	Gm71804	predicted gene, 71804 [Source:MGI Symbol;Acc:MGI:7826374]
5	lncRNA	1.12E+08	1.12E+08	ENSMUSG00000139603	MGI:7828942	Gm73092	predicted gene, 73092 [Source:MGI Symbol;Acc:MGI:7828942]
5	lncRNA	1.12E+08	1.12E+08	ENSMUSG00000104538	MGI:1922890	1700016B01Rik	RIKEN cDNA 1700016B01 gene [Source:MGI Symbol;Acc:MGI:1922890]
5	lncRNA	1.12E+08	1.12E+08	ENSMUSG00000139184	MGI:7828820	Gm73031	predicted gene, 73031 [Source:MGI Symbol;Acc:MGI:7828820]
5	lncRNA	1.12E+08	1.12E+08	ENSMUSG00000133363	MGI:7827134	Gm72184	predicted gene, 72184 [Source:MGI Symbol;Acc:MGI:7827134]
5	lncRNA	1.12E+08	1.12E+08	ENSMUSG00000101683	MGI:1922819	1700028D13Rik	RIKEN cDNA 1700028D13 gene [Source:MGI Symbol;Acc:MGI:1922819]
5	lncRNA	1.12E+08	1.12E+08	ENSMUSG00000097767	MGI:2444886	Miat	myocardial infarction associated transcript (non-protein coding) [Source:MGI Symbol;Acc:MGI:2444886]
5	lncRNA	1.12E+08	1.12E+08	ENSMUSG00000097986	MGI:5504068	Gm26953	predicted gene, 26953 [Source:MGI Symbol;Acc:MGI:5504068]
5	lncRNA	1.12E+08	1.12E+08	ENSMUSG00000133381	MGI:7827140	Gm72187	predicted gene, 72187 [Source:MGI Symbol;Acc:MGI:7827140]
5	lncRNA	1.12E+08	1.12E+08	ENSMUSG00000138023	MGI:5826578	Gm46941	predicted gene, 46941 [Source:MGI Symbol;Acc:MGI:5826578]
5	lncRNA	1.13E+08	1.13E+08	ENSMUSG00000093637	MGI:5313083	Gm20636	predicted gene 20636 [Source:MGI Symbol;Acc:MGI:5313083]
5	lncRNA	1.13E+08	1.13E+08	ENSMUSG00000130751	MGI:7826280	Gm71756	predicted gene, 71756 [Source:MGI Symbol;Acc:MGI:7826280]
5	lncRNA	1.13E+08	1.13E+08	ENSMUSG00000124762	MGI:7825561	Gm71394	predicted gene, 71394 [Source:MGI Symbol;Acc:MGI:7825561]

5	lncRNA	1.13E+08	1.13E+08	ENSMUSG00000135548	MGI:7827732	Gm72484	predicted gene, 72484 [Source:MGI Symbol;Acc:MGI:7827732]
5	lncRNA	1.13E+08	1.13E+08	ENSMUSG00000124512	MGI:7825537	Gm71382	predicted gene, 71382 [Source:MGI Symbol;Acc:MGI:7825537]
5	lncRNA	1.13E+08	1.13E+08	ENSMUSG00000124546	MGI:7825543	Gm71385	predicted gene, 71385 [Source:MGI Symbol;Acc:MGI:7825543]
5	lncRNA	1.13E+08	1.13E+08	ENSMUSG00000097962	MGI:1914575	1700034G24Rik	RIKEN cDNA 1700034G24 gene [Source:MGI Symbol;Acc:MGI:1914575]
5	lncRNA	1.13E+08	1.13E+08	ENSMUSG00000098027	MGI:5504119	Gm27004	predicted gene, 27004 [Source:MGI Symbol;Acc:MGI:5504119]
5	lncRNA	1.13E+08	1.13E+08	ENSMUSG00000122359	MGI:7825255	Gm71237	predicted gene, 71237 [Source:MGI Symbol;Acc:MGI:7825255]
5	lncRNA	1.13E+08	1.13E+08	ENSMUSG00000104807	MGI:5663002	Gm42865	predicted gene 42865 [Source:MGI Symbol;Acc:MGI:5663002]
5	lncRNA	1.13E+08	1.13E+08	ENSMUSG00000127191	MGI:7825797	Gm71512	predicted gene, 71512 [Source:MGI Symbol;Acc:MGI:7825797]
5	lncRNA	1.13E+08	1.13E+08	ENSMUSG00000098230	MGI:1914610	1700095B10Rik	RIKEN cDNA 1700095B10 gene [Source:MGI Symbol;Acc:MGI:1914610]
5	lncRNA	1.13E+08	1.13E+08	ENSMUSG00000129505	MGI:7825985	Gm71606	predicted gene, 71606 [Source:MGI Symbol;Acc:MGI:7825985]
5	lncRNA	1.13E+08	1.13E+08	ENSMUSG00000134035	MGI:7827316	Gm72275	predicted gene, 72275 [Source:MGI Symbol;Acc:MGI:7827316]
5	lncRNA	1.13E+08	1.13E+08	ENSMUSG00000135925	MGI:7827794	Gm72515	predicted gene, 72515 [Source:MGI Symbol;Acc:MGI:7827794]
5	lncRNA	1.13E+08	1.13E+08	ENSMUSG00000136353	MGI:7827946	Gm72592	predicted gene, 72592 [Source:MGI Symbol;Acc:MGI:7827946]
5	lncRNA	1.13E+08	1.13E+08	ENSMUSG00000137004	MGI:7828260	Gm72749	predicted gene, 72749 [Source:MGI Symbol;Acc:MGI:7828260]
5	lncRNA	1.13E+08	1.13E+08	ENSMUSG00000141774	MGI:1926036	4833439F03Rik	RIKEN cDNA 4833439F03 gene [Source:MGI Symbol;Acc:MGI:1926036]
5	lncRNA	1.13E+08	1.13E+08	ENSMUSG00000106659	MGI:5625046	Gm42161	predicted gene, 42161 [Source:MGI Symbol;Acc:MGI:5625046]
5	lncRNA	1.13E+08	1.13E+08	ENSMUSG00000127988	MGI:7825843	Gm71535	predicted gene, 71535 [Source:MGI Symbol;Acc:MGI:7825843]
5	lncRNA	1.13E+08	1.13E+08	ENSMUSG00000138601	MGI:7828646	Gm72943	predicted gene, 72943 [Source:MGI Symbol;Acc:MGI:7828646]

5	lncRNA	1.13E+08	1.13E+08	ENSMUSG00000141062	MGI:7829336	Gm73289	predicted gene, 73289 [Source:MGI Symbol;Acc:MGI:7829336]
5	lncRNA	1.13E+08	1.13E+08	ENSMUSG00000104705	MGI:1921047	4930405N21Rik	RIKEN cDNA 4930405N21 gene [Source:MGI Symbol;Acc:MGI:1921047]
5	lncRNA	1.13E+08	1.13E+08	ENSMUSG00000105174	MGI:5663490	Gm43353	predicted gene 43353 [Source:MGI Symbol;Acc:MGI:5663490]
5	lncRNA	1.13E+08	1.13E+08	ENSMUSG00000143806	MGI:7829986	Gm73615	predicted gene, 73615 [Source:MGI Symbol;Acc:MGI:7829986]
5	lncRNA	1.13E+08	1.14E+08	ENSMUSG00000126830	MGI:1921478	1700016C19Rik	RIKEN cDNA 1700016C19 gene [Source:MGI Symbol;Acc:MGI:1921478]
5	lncRNA	1.14E+08	1.14E+08	ENSMUSG00000126864	MGI:7825761	Gm71494	predicted gene, 71494 [Source:MGI Symbol;Acc:MGI:7825761]
5	lncRNA	1.14E+08	1.14E+08	ENSMUSG00000053873	MGI:1891321	Aym1	activator of yeast meiotic promoters 1 [Source:MGI Symbol;Acc:MGI:1891321]
5	lncRNA	1.14E+08	1.14E+08	ENSMUSG00000133155	MGI:7826994	Gm72114	predicted gene, 72114 [Source:MGI Symbol;Acc:MGI:7826994]
5	lncRNA	1.14E+08	1.14E+08	ENSMUSG00000143804	MGI:7829980	Gm73612	predicted gene, 73612 [Source:MGI Symbol;Acc:MGI:7829980]
5	lncRNA	1.14E+08	1.14E+08	ENSMUSG00000140157	MGI:7829054	Gm73148	predicted gene, 73148 [Source:MGI Symbol;Acc:MGI:7829054]
5	lncRNA	1.14E+08	1.14E+08	ENSMUSG00000139134	MGI:7828798	Gm73019	predicted gene, 73019 [Source:MGI Symbol;Acc:MGI:7828798]
5	lncRNA	1.14E+08	1.14E+08	ENSMUSG00000131097	MGI:5589971	Gm30812	predicted gene, 30812 [Source:MGI Symbol;Acc:MGI:5589971]
5	lncRNA	1.14E+08	1.14E+08	ENSMUSG00000131138	MGI:7826418	Gm71826	predicted gene, 71826 [Source:MGI Symbol;Acc:MGI:7826418]
5	lncRNA	1.14E+08	1.14E+08	ENSMUSG00000120761	MGI:7338541	Gm57314	predicted gene, 57314 [Source:MGI Symbol;Acc:MGI:7338541]
5	lncRNA	1.14E+08	1.14E+08	ENSMUSG00000126419	MGI:7825721	Gm71474	predicted gene, 71474 [Source:MGI Symbol;Acc:MGI:7825721]
5	lncRNA	1.14E+08	1.14E+08	ENSMUSG00000126378	MGI:7825719	Gm71473	predicted gene, 71473 [Source:MGI Symbol;Acc:MGI:7825719]
5	lncRNA	1.14E+08	1.14E+08	ENSMUSG00000126337	MGI:7825715	Gm71471	predicted gene, 71471 [Source:MGI Symbol;Acc:MGI:7825715]
5	lncRNA	1.14E+08	1.14E+08	ENSMUSG00000129427	MGI:7825977	Gm71602	predicted gene, 71602 [Source:MGI Symbol;Acc:MGI:7825977]

5	lncRNA	1.14E+08	1.14E+08	ENSMUSG00000085713	MGI:3783178	Gm15736	predicted gene 15736 [Source:MGI Symbol;Acc:MGI:3783178]
5	lncRNA	1.14E+08	1.14E+08	ENSMUSG00000134876	MGI:7827550	Gm72392	predicted gene, 72392 [Source:MGI Symbol;Acc:MGI:7827550]
5	lncRNA	1.14E+08	1.14E+08	ENSMUSG00000125163	MGI:7825589	Gm71408	predicted gene, 71408 [Source:MGI Symbol;Acc:MGI:7825589]
5	lncRNA	1.14E+08	1.14E+08	ENSMUSG00000125195	MGI:7825595	Gm71411	predicted gene, 71411 [Source:MGI Symbol;Acc:MGI:7825595]
5	lncRNA	1.14E+08	1.14E+08	ENSMUSG00000042184	MGI:1920726	1700069L16Rik	RIKEN cDNA 1700069L16 gene [Source:MGI Symbol;Acc:MGI:1920726]
5	lncRNA	1.14E+08	1.14E+08	ENSMUSG00000133974	MGI:7827298	Gm72266	predicted gene, 72266 [Source:MGI Symbol;Acc:MGI:7827298]
5	lncRNA	1.14E+08	1.14E+08	ENSMUSG00000135715	MGI:5624889	Gm42004	predicted gene, 42004 [Source:MGI Symbol;Acc:MGI:5624889]
5	lncRNA	1.14E+08	1.14E+08	ENSMUSG00000130821	MGI:7826300	Gm71766	predicted gene, 71766 [Source:MGI Symbol;Acc:MGI:7826300]
5	lncRNA	1.14E+08	1.14E+08	ENSMUSG00000090458	MGI:4937949	Gm17122	predicted gene 17122 [Source:MGI Symbol;Acc:MGI:4937949]
5	lncRNA	1.14E+08	1.14E+08	ENSMUSG00000123600	MGI:7825423	Gm71323	predicted gene, 71323 [Source:MGI Symbol;Acc:MGI:7825423]
5	lncRNA	1.14E+08	1.14E+08	ENSMUSG00000105664	MGI:5662934	Gm42797	predicted gene 42797 [Source:MGI Symbol;Acc:MGI:5662934]
5	lncRNA	1.14E+08	1.14E+08	ENSMUSG00000123190	MGI:7825366	Gm71294	predicted gene, 71294 [Source:MGI Symbol;Acc:MGI:7825366]
5	lncRNA	1.14E+08	1.14E+08	ENSMUSG00000140843	MGI:7829232	Gm73237	predicted gene, 73237 [Source:MGI Symbol;Acc:MGI:7829232]
5	lncRNA	1.14E+08	1.14E+08	ENSMUSG00000140808	MGI:7829228	Gm73235	predicted gene, 73235 [Source:MGI Symbol;Acc:MGI:7829228]
5	lncRNA	1.14E+08	1.14E+08	ENSMUSG00000127137	MGI:7825793	Gm71510	predicted gene, 71510 [Source:MGI Symbol;Acc:MGI:7825793]
5	lncRNA	1.15E+08	1.15E+08	ENSMUSG00000122018	MGI:5593881	Gm34722	predicted gene, 34722 [Source:MGI Symbol;Acc:MGI:5593881]
5	lncRNA	1.15E+08	1.15E+08	ENSMUSG00000143223	MGI:7829748	Gm73496	predicted gene, 73496 [Source:MGI Symbol;Acc:MGI:7829748]
5	lncRNA	1.15E+08	1.15E+08	ENSMUSG00000137482	MGI:7828430	Gm72834	predicted gene, 72834 [Source:MGI Symbol;Acc:MGI:7828430]

5	lncRNA	1.15E+08	1.15E+08	ENSMUSG00000140498	MGI:7829156	Gm73199	predicted gene, 73199 [Source:MGI Symbol;Acc:MGI:7829156]
5	lncRNA	1.15E+08	1.15E+08	ENSMUSG00000086302	MGI:3650747	Gm13790	predicted gene 13790 [Source:MGI Symbol;Acc:MGI:3650747]
5	lncRNA	1.15E+08	1.15E+08	ENSMUSG00000127419	MGI:7825809	Gm71518	predicted gene, 71518 [Source:MGI Symbol;Acc:MGI:7825809]
5	lncRNA	1.15E+08	1.15E+08	ENSMUSG00000127459	MGI:7825811	Gm71519	predicted gene, 71519 [Source:MGI Symbol;Acc:MGI:7825811]
5	lncRNA	1.15E+08	1.15E+08	ENSMUSG00000140177	MGI:7829060	Gm73151	predicted gene, 73151 [Source:MGI Symbol;Acc:MGI:7829060]
5	lncRNA	1.15E+08	1.15E+08	ENSMUSG00000122765	MGI:5594003	Gm34844	predicted gene, 34844 [Source:MGI Symbol;Acc:MGI:5594003]
5	lncRNA	1.15E+08	1.15E+08	ENSMUSG00000127492	MGI:7825813	Gm71520	predicted gene, 71520 [Source:MGI Symbol;Acc:MGI:7825813]
5	lncRNA	1.15E+08	1.15E+08	ENSMUSG00000141988	MGI:7829480	Gm73361	predicted gene, 73361 [Source:MGI Symbol;Acc:MGI:7829480]
5	lncRNA	1.15E+08	1.15E+08	ENSMUSG00000139305	MGI:5594090	Gm34931	predicted gene, 34931 [Source:MGI Symbol;Acc:MGI:5594090]
5	lncRNA	1.15E+08	1.15E+08	ENSMUSG00000139348	MGI:7828852	Gm73047	predicted gene, 73047 [Source:MGI Symbol;Acc:MGI:7828852]
5	lncRNA	1.15E+08	1.15E+08	ENSMUSG00000107340	MGI:5662926	Gm42789	predicted gene 42789 [Source:MGI Symbol;Acc:MGI:5662926]
5	lncRNA	1.15E+08	1.15E+08	ENSMUSG00000092183	MGI:1914892	4930515G01Rik	RIKEN cDNA 4930515G01 gene [Source:MGI Symbol;Acc:MGI:1914892]
5	lncRNA	1.15E+08	1.15E+08	ENSMUSG00000133025	MGI:7826956	Gm72095	predicted gene, 72095 [Source:MGI Symbol;Acc:MGI:7826956]
5	lncRNA	1.15E+08	1.15E+08	ENSMUSG00000133116	MGI:7826968	Gm72101	predicted gene, 72101 [Source:MGI Symbol;Acc:MGI:7826968]
5	lncRNA	1.15E+08	1.15E+08	ENSMUSG00000132938	MGI:7826888	Gm72061	predicted gene, 72061 [Source:MGI Symbol;Acc:MGI:7826888]
5	lncRNA	1.15E+08	1.15E+08	ENSMUSG00000132979	MGI:7826908	Gm72071	predicted gene, 72071 [Source:MGI Symbol;Acc:MGI:7826908]
5	lncRNA	1.15E+08	1.15E+08	ENSMUSG00000131648	MGI:7826602	Gm71918	predicted gene, 71918 [Source:MGI Symbol;Acc:MGI:7826602]
5	lncRNA	1.15E+08	1.15E+08	ENSMUSG00000107108	MGI:3641854	Gm9936	predicted gene 9936 [Source:MGI Symbol;Acc:MGI:3641854]

5	lncRNA	1.15E+08	1.15E+08	ENSMUSG00000135230	MGI:7827654	Gm72445	predicted gene, 72445 [Source:MGI Symbol;Acc:MGI:7827654]
5	lncRNA	1.15E+08	1.15E+08	ENSMUSG00000087477	MGI:3650351	Gm13822	predicted gene 13822 [Source:MGI Symbol;Acc:MGI:3650351]
5	lncRNA	1.15E+08	1.15E+08	ENSMUSG00000122036	MGI:7825201	Gm71210	predicted gene, 71210 [Source:MGI Symbol;Acc:MGI:7825201]
5	lncRNA	1.15E+08	1.15E+08	ENSMUSG00000138573	MGI:7828632	Gm72936	predicted gene, 72936 [Source:MGI Symbol;Acc:MGI:7828632]
5	lncRNA	1.15E+08	1.15E+08	ENSMUSG00000129685	MGI:7826015	Gm71622	predicted gene, 71622 [Source:MGI Symbol;Acc:MGI:7826015]
5	lncRNA	1.15E+08	1.15E+08	ENSMUSG00000086054	MGI:3652225	Hnf1aos1	HNF1 homeobox A, opposite strand 1 [Source:MGI Symbol;Acc:MGI:3652225]
5	lncRNA	1.15E+08	1.15E+08	ENSMUSG00000086140	MGI:3652220	Hnf1aos2	HNF1 homeobox A, opposite strand 2 [Source:MGI Symbol;Acc:MGI:3652220]
5	lncRNA	1.15E+08	1.15E+08	ENSMUSG00000129727	MGI:7826021	Gm71625	predicted gene, 71625 [Source:MGI Symbol;Acc:MGI:7826021]
5	lncRNA	1.15E+08	1.15E+08	ENSMUSG00000121818			ribosomal protein L37, retrotransposed [Source:NCBI gene (formerly Entrezgene);Acc:100502825]
5	lncRNA	1.15E+08	1.15E+08	ENSMUSG00000136454	MGI:7827964	Gm72601	predicted gene, 72601 [Source:MGI Symbol;Acc:MGI:7827964]
5	lncRNA	1.15E+08	1.15E+08	ENSMUSG00000120554	MGI:7338449	Gm57268	predicted gene, 57268 [Source:MGI Symbol;Acc:MGI:7338449]
5	lncRNA	1.15E+08	1.15E+08	ENSMUSG00000128459	MGI:7825901	Gm71564	predicted gene, 71564 [Source:MGI Symbol;Acc:MGI:7825901]
5	lncRNA	1.15E+08	1.15E+08	ENSMUSG00000086368	MGI:3651141	Gm13830	predicted gene 13830 [Source:MGI Symbol;Acc:MGI:3651141]
5	lncRNA	1.15E+08	1.15E+08	ENSMUSG00000121115	MGI:7338553	Gm57320	predicted gene, 57320 [Source:MGI Symbol;Acc:MGI:7338553]
5	lncRNA	1.15E+08	1.15E+08	ENSMUSG00000107288	MGI:1925175	A430102K17Rik	RIKEN cDNA A430102K17 gene [Source:MGI Symbol;Acc:MGI:1925175]
5	lncRNA	1.15E+08	1.16E+08	ENSMUSG00000086352	MGI:1921042	4930401G09Rik	RIKEN cDNA 4930401G09 gene [Source:MGI Symbol;Acc:MGI:1921042]
5	lncRNA	1.15E+08	1.16E+08	ENSMUSG00000087292	MGI:3650708	Gm13832	predicted gene 13832 [Source:MGI Symbol;Acc:MGI:3650708]

5	lncRNA	1.16E+08	1.16E+08	ENSMUSG00000124350			[Source:NCBI gene (formerly Entrezgene);Acc:102639568]
5	lncRNA	1.16E+08	1.16E+08	ENSMUSG00000107353	MGI:1925404	4930430O22Rik	RIKEN cDNA 4930430O22 gene [Source:MGI Symbol;Acc:MGI:1925404]
5	lncRNA	1.16E+08	1.16E+08	ENSMUSG00000127019	MGI:7825779	Gm71503	predicted gene, 71503 [Source:MGI Symbol;Acc:MGI:7825779]
5	lncRNA	1.16E+08	1.16E+08	ENSMUSG00000120846	MGI:7338509	Gm57298	predicted gene, 57298 [Source:MGI Symbol;Acc:MGI:7338509]
5	lncRNA	1.16E+08	1.16E+08	ENSMUSG00000121040	MGI:7338523	Gm57305	predicted gene, 57305 [Source:MGI Symbol;Acc:MGI:7338523]
5	lncRNA	1.16E+08	1.16E+08	ENSMUSG00000132950	MGI:7826896	Gm72065	predicted gene, 72065 [Source:MGI Symbol;Acc:MGI:7826896]
5	lncRNA	1.16E+08	1.16E+08	ENSMUSG00000126088	MGI:7830410	Gm73833	predicted gene, 73833 [Source:MGI Symbol;Acc:MGI:7830410]
5	lncRNA	1.16E+08	1.16E+08	ENSMUSG00000130738	MGI:7826278	Gm71755	predicted gene, 71755 [Source:MGI Symbol;Acc:MGI:7826278]
5	lncRNA	1.16E+08	1.16E+08	ENSMUSG00000139020	MGI:7828780	Gm73010	predicted gene, 73010 [Source:MGI Symbol;Acc:MGI:7828780]
5	lncRNA	1.16E+08	1.16E+08	ENSMUSG00000086849	MGI:3649479	Gm13840	predicted gene 13840 [Source:MGI Symbol;Acc:MGI:3649479]
5	lncRNA	1.16E+08	1.16E+08	ENSMUSG00000140383	MGI:7829134	Gm73188	predicted gene, 73188 [Source:MGI Symbol;Acc:MGI:7829134]
5	lncRNA	1.16E+08	1.16E+08	ENSMUSG00000107121	MGI:1913373	1110006O24Rik	RIKEN cDNA 1110006O24 gene [Source:MGI Symbol;Acc:MGI:1913373]
5	lncRNA	1.16E+08	1.16E+08	ENSMUSG00000135244	MGI:7827656	Gm72446	predicted gene, 72446 [Source:MGI Symbol;Acc:MGI:7827656]
5	lncRNA	1.16E+08	1.16E+08	ENSMUSG00000125952	MGI:7825669	Gm71448	predicted gene, 71448 [Source:MGI Symbol;Acc:MGI:7825669]
5	lncRNA	1.16E+08	1.16E+08	ENSMUSG00000125719	MGI:7825645	Gm71436	predicted gene, 71436 [Source:MGI Symbol;Acc:MGI:7825645]
5	lncRNA	1.16E+08	1.16E+08	ENSMUSG00000134680	MGI:5624893	Gm42008	predicted gene, 42008 [Source:MGI Symbol;Acc:MGI:5624893]
5	lncRNA	1.16E+08	1.16E+08	ENSMUSG00000120953	MGI:5595344	Gm36185	predicted gene, 36185 [Source:MGI Symbol;Acc:MGI:5595344]
5	lncRNA	1.16E+08	1.16E+08	ENSMUSG00000085376	MGI:3705279	Gm14508	predicted gene 14508 [Source:MGI Symbol;Acc:MGI:3705279]

5	lncRNA	1.16E+08	1.16E+08	ENSMUSG00000084992	MGI:3650892	Gm13842	predicted gene 13842 [Source:MGI Symbol;Acc:MGI:3650892]
5	lncRNA	1.16E+08	1.16E+08	ENSMUSG00000131869	MGI:7826640	Gm71937	predicted gene, 71937 [Source:MGI Symbol;Acc:MGI:7826640]
5	lncRNA	1.16E+08	1.16E+08	ENSMUSG00000127595	MGI:7825825	Gm71526	predicted gene, 71526 [Source:MGI Symbol;Acc:MGI:7825825]
5	lncRNA	1.16E+08	1.16E+08	ENSMUSG00000086655	MGI:2444781	C330018A13Rik	RIKEN cDNA C330018A13 gene [Source:MGI Symbol;Acc:MGI:2444781]
5	lncRNA	1.16E+08	1.16E+08	ENSMUSG00000086122	MGI:3705240	Gm14507	predicted gene 14507 [Source:MGI Symbol;Acc:MGI:3705240]
5	lncRNA	1.16E+08	1.16E+08	ENSMUSG00000086055	MGI:3650972	Gm13837	predicted gene 13837 [Source:MGI Symbol;Acc:MGI:3650972]
5	lncRNA	1.16E+08	1.16E+08	ENSMUSG00000131570	MGI:7826574	Gm71904	predicted gene, 71904 [Source:MGI Symbol;Acc:MGI:7826574]
5	lncRNA	1.16E+08	1.16E+08	ENSMUSG00000087124	MGI:3650513	Gm13839	predicted gene 13839 [Source:MGI Symbol;Acc:MGI:3650513]
5	lncRNA	1.16E+08	1.16E+08	ENSMUSG00000084921	MGI:3649377	Gm13838	predicted gene 13838 [Source:MGI Symbol;Acc:MGI:3649377]
5	lncRNA	1.16E+08	1.16E+08	ENSMUSG00000086715	MGI:1925096	B230112J18Rik	RIKEN cDNA B230112J18 gene [Source:MGI Symbol;Acc:MGI:1925096]
5	lncRNA	1.17E+08	1.17E+08	ENSMUSG00000126742	MGI:7825751	Gm71489	predicted gene, 71489 [Source:MGI Symbol;Acc:MGI:7825751]
5	lncRNA	1.17E+08	1.17E+08	ENSMUSG00000086945	MGI:1922553	4930562A09Rik	RIKEN cDNA 4930562A09 gene [Source:MGI Symbol;Acc:MGI:1922553]
5	lncRNA	1.17E+08	1.17E+08	ENSMUSG00000133941	MGI:7827262	Gm72248	predicted gene, 72248 [Source:MGI Symbol;Acc:MGI:7827262]
5	lncRNA	1.17E+08	1.17E+08	ENSMUSG00000134017	MGI:5624894	Gm42009	predicted gene, 42009 [Source:MGI Symbol;Acc:MGI:5624894]
5	lncRNA	1.17E+08	1.17E+08	ENSMUSG00000134063	MGI:7827330	Gm72282	predicted gene, 72282 [Source:MGI Symbol;Acc:MGI:7827330]
5	lncRNA	1.17E+08	1.17E+08	ENSMUSG00000086219	MGI:1924048	Srrm4os	serine/arginine repetitive matrix 4, opposite strand [Source:MGI Symbol;Acc:MGI:1924048]
5	lncRNA	1.17E+08	1.17E+08	ENSMUSG00000134106	MGI:7827362	Gm72298	predicted gene, 72298 [Source:MGI Symbol;Acc:MGI:7827362]
5	lncRNA	1.17E+08	1.17E+08	ENSMUSG00000106914	MGI:5662991	Gm42854	predicted gene 42854 [Source:MGI Symbol;Acc:MGI:5662991]

5	lncRNA	1.17E+08	1.17E+08	ENSMUSG00000132062	MGI:7826700	Gm71967	predicted gene, 71967 [Source:MGI Symbol;Acc:MGI:7826700]
5	lncRNA	1.17E+08	1.17E+08	ENSMUSG00000132023	MGI:7826688	Gm71961	predicted gene, 71961 [Source:MGI Symbol;Acc:MGI:7826688]
5	lncRNA	1.17E+08	1.17E+08	ENSMUSG00000106909	MGI:5663259	Gm43122	predicted gene 43122 [Source:MGI Symbol;Acc:MGI:5663259]
5	lncRNA	1.17E+08	1.17E+08	ENSMUSG00000128674	MGI:7825923	Gm71575	predicted gene, 71575 [Source:MGI Symbol;Acc:MGI:7825923]
5	lncRNA	1.17E+08	1.17E+08	ENSMUSG00000128640	MGI:5589002	Gm29843	predicted gene, 29843 [Source:MGI Symbol;Acc:MGI:5589002]
5	lncRNA	1.17E+08	1.17E+08	ENSMUSG00000107237	MGI:5589085	Gm29926	predicted gene, 29926 [Source:MGI Symbol;Acc:MGI:5589085]
5	lncRNA	1.17E+08	1.17E+08	ENSMUSG00000128718	MGI:7825929	Gm71578	predicted gene, 71578 [Source:MGI Symbol;Acc:MGI:7825929]
5	lncRNA	1.17E+08	1.17E+08	ENSMUSG00000128565	MGI:7825917	Gm71572	predicted gene, 71572 [Source:MGI Symbol;Acc:MGI:7825917]
5	lncRNA	1.17E+08	1.17E+08	ENSMUSG00000133049	MGI:7826960	Gm72097	predicted gene, 72097 [Source:MGI Symbol;Acc:MGI:7826960]
5	lncRNA	1.17E+08	1.17E+08	ENSMUSG00000130385	MGI:7826208	Gm71720	predicted gene, 71720 [Source:MGI Symbol;Acc:MGI:7826208]
5	lncRNA	1.17E+08	1.17E+08	ENSMUSG00000131824	MGI:5624895	Gm42010	predicted gene, 42010 [Source:MGI Symbol;Acc:MGI:5624895]
5	lncRNA	1.17E+08	1.17E+08	ENSMUSG00000128116	MGI:7825855	Gm71541	predicted gene, 71541 [Source:MGI Symbol;Acc:MGI:7825855]
5	lncRNA	1.17E+08	1.17E+08	ENSMUSG00000139383	MGI:7828860	Gm73051	predicted gene, 73051 [Source:MGI Symbol;Acc:MGI:7828860]
5	lncRNA	1.17E+08	1.17E+08	ENSMUSG00000129200	MGI:5589131	Gm29972	predicted gene, 29972 [Source:MGI Symbol;Acc:MGI:5589131]
5	lncRNA	1.17E+08	1.17E+08	ENSMUSG00000129242	MGI:5589187	Gm30028	predicted gene, 30028 [Source:MGI Symbol;Acc:MGI:5589187]
5	lncRNA	1.17E+08	1.17E+08	ENSMUSG00000129278	MGI:7825969	Gm71598	predicted gene, 71598 [Source:MGI Symbol;Acc:MGI:7825969]
5	lncRNA	1.17E+08	1.17E+08	ENSMUSG00000086047	MGI:1925671	9530046B11Rik	RIKEN cDNA 9530046B11 gene [Source:MGI Symbol;Acc:MGI:1925671]
5	lncRNA	1.17E+08	1.17E+08	ENSMUSG00000120368	MGI:7338439	Gm57263	predicted gene, 57263 [Source:MGI Symbol;Acc:MGI:7338439]

5	lncRNA	1.17E+08	1.17E+08	ENSMUSG00000136822	MGI:7828180	Gm72709	predicted gene, 72709 [Source:MGI Symbol;Acc:MGI:7828180]
5	lncRNA	1.17E+08	1.17E+08	ENSMUSG00000126795	MGI:6367266	Gm52828	predicted gene, 52828 [Source:MGI Symbol;Acc:MGI:6367266]
5	lncRNA	1.17E+08	1.17E+08	ENSMUSG00000126840	MGI:7825757	Gm71492	predicted gene, 71492 [Source:MGI Symbol;Acc:MGI:7825757]
5	lncRNA	1.17E+08	1.17E+08	ENSMUSG00000097163	MGI:3039587	BC051077	cDNA sequence BC051077 [Source:MGI Symbol;Acc:MGI:3039587]
5	lncRNA	1.17E+08	1.17E+08	ENSMUSG00000072688	MGI:3704255	Gm10399	predicted gene 10399 [Source:MGI Symbol;Acc:MGI:3704255]
5	lncRNA	1.17E+08	1.17E+08	ENSMUSG00000122690	MGI:5624897	Gm42012	predicted gene, 42012 [Source:MGI Symbol;Acc:MGI:5624897]
5	lncRNA	1.18E+08	1.18E+08	ENSMUSG00000086075	MGI:3783171	Gm15728	predicted gene 15728 [Source:MGI Symbol;Acc:MGI:3783171]
5	lncRNA	1.18E+08	1.18E+08	ENSMUSG00000120827	MGI:6367268	Gm52829	predicted gene, 52829 [Source:MGI Symbol;Acc:MGI:6367268]
5	lncRNA	1.18E+08	1.18E+08	ENSMUSG00000128985	MGI:7825945	Gm71586	predicted gene, 71586 [Source:MGI Symbol;Acc:MGI:7825945]
5	lncRNA	1.18E+08	1.18E+08	ENSMUSG00000138250	MGI:7828548	Gm72894	predicted gene, 72894 [Source:MGI Symbol;Acc:MGI:7828548]
5	lncRNA	1.18E+08	1.18E+08	ENSMUSG00000126651	MGI:7825737	Gm71482	predicted gene, 71482 [Source:MGI Symbol;Acc:MGI:7825737]
5	lncRNA	1.18E+08	1.18E+08	ENSMUSG00000123983	MGI:7825465	Gm71345	predicted gene, 71345 [Source:MGI Symbol;Acc:MGI:7825465]
5	lncRNA	1.18E+08	1.18E+08	ENSMUSG00000128859	MGI:7831172	Gm74217	predicted gene, 74217 [Source:MGI Symbol;Acc:MGI:7831172]
5	lncRNA	1.18E+08	1.18E+08	ENSMUSG00000137379	MGI:5589519	Gm30360	predicted gene, 30360 [Source:MGI Symbol;Acc:MGI:5589519]
5	lncRNA	1.18E+08	1.18E+08	ENSMUSG00000136798	MGI:7828174	Gm72706	predicted gene, 72706 [Source:MGI Symbol;Acc:MGI:7828174]
5	lncRNA	1.18E+08	1.18E+08	ENSMUSG00000098072	MGI:5504110	Gm26995	predicted gene, 26995 [Source:MGI Symbol;Acc:MGI:5504110]
5	lncRNA	1.18E+08	1.18E+08	ENSMUSG00000029360	MGI:3704256	Gm9754	predicted gene 9754 [Source:MGI Symbol;Acc:MGI:3704256]
5	lncRNA	1.18E+08	1.18E+08	ENSMUSG00000139919	MGI:7828972	Gm73107	predicted gene, 73107 [Source:MGI Symbol;Acc:MGI:7828972]

5	lncRNA	1.18E+08	1.18E+08	ENSMUSG00000141016	MGI:7829290	Gm73266	predicted gene, 73266 [Source:MGI Symbol;Acc:MGI:7829290]
5	lncRNA	1.18E+08	1.18E+08	ENSMUSG00000143393	MGI:7829804	Gm73524	predicted gene, 73524 [Source:MGI Symbol;Acc:MGI:7829804]
5	lncRNA	1.18E+08	1.18E+08	ENSMUSG00000141505	MGI:7829394	Gm73318	predicted gene, 73318 [Source:MGI Symbol;Acc:MGI:7829394]
5	lncRNA	1.18E+08	1.18E+08	ENSMUSG00000140965	MGI:7829266	Gm73254	predicted gene, 73254 [Source:MGI Symbol;Acc:MGI:7829266]
5	lncRNA	1.18E+08	1.18E+08	ENSMUSG00000122569	MGI:5826579	Gm46942	predicted gene, 46942 [Source:MGI Symbol;Acc:MGI:5826579]
5	lncRNA	1.18E+08	1.19E+08	ENSMUSG00000128713	MGI:7825925	Gm71576	predicted gene, 71576 [Source:MGI Symbol;Acc:MGI:7825925]
5	lncRNA	1.19E+08	1.19E+08	ENSMUSG00000141024	MGI:5589763	Gm30604	predicted gene, 30604 [Source:MGI Symbol;Acc:MGI:5589763]
5	lncRNA	1.19E+08	1.19E+08	ENSMUSG00000106812	MGI:5579269	Gm28563	predicted gene 28563 [Source:MGI Symbol;Acc:MGI:5579269]
5	lncRNA	1.19E+08	1.19E+08	ENSMUSG00000138714	MGI:7828674	Gm72957	predicted gene, 72957 [Source:MGI Symbol;Acc:MGI:7828674]
5	lncRNA	1.19E+08	1.19E+08	ENSMUSG00000127080	MGI:7825787	Gm71507	predicted gene, 71507 [Source:MGI Symbol;Acc:MGI:7825787]
5	lncRNA	1.19E+08	1.19E+08	ENSMUSG00000126021	MGI:5589905	Gm30746	predicted gene, 30746 [Source:MGI Symbol;Acc:MGI:5589905]
5	lncRNA	1.19E+08	1.19E+08	ENSMUSG00000130380	MGI:5826558	Gm46921	predicted gene, 46921 [Source:MGI Symbol;Acc:MGI:5826558]
5	lncRNA	1.19E+08	1.19E+08	ENSMUSG00000121735	MGI:7826194	Gm71713	predicted gene, 71713 [Source:MGI Symbol;Acc:MGI:7826194]
5	lncRNA	1.19E+08	1.19E+08	ENSMUSG00000128021	MGI:7825847	Gm71537	predicted gene, 71537 [Source:MGI Symbol;Acc:MGI:7825847]
5	lncRNA	1.19E+08	1.19E+08	ENSMUSG00000120779	MGI:7338499	Gm57293	predicted gene, 57293 [Source:MGI Symbol;Acc:MGI:7338499]
5	lncRNA	1.19E+08	1.19E+08	ENSMUSG00000130938	MGI:7826312	Gm71773	predicted gene, 71773 [Source:MGI Symbol;Acc:MGI:7826312]
5	lncRNA	1.19E+08	1.19E+08	ENSMUSG00000139964	MGI:7828978	Gm73110	predicted gene, 73110 [Source:MGI Symbol;Acc:MGI:7828978]
5	lncRNA	1.19E+08	1.19E+08	ENSMUSG00000134410	MGI:7827444	Gm72339	predicted gene, 72339 [Source:MGI Symbol;Acc:MGI:7827444]

5	lncRNA	1.19E+08	1.19E+08	ENSMUSG00000135281	MGI:7827668	Gm72452	predicted gene, 72452 [Source:MGI Symbol;Acc:MGI:7827668]
5	lncRNA	1.19E+08	1.19E+08	ENSMUSG00000126319	MGI:7825713	Gm71470	predicted gene, 71470 [Source:MGI Symbol;Acc:MGI:7825713]
5	lncRNA	1.19E+08	1.19E+08	ENSMUSG00000123304	MGI:5624900	Gm42015	predicted gene, 42015 [Source:MGI Symbol;Acc:MGI:5624900]
5	lncRNA	1.19E+08	1.19E+08	ENSMUSG00000132475	MGI:7826790	Gm72012	predicted gene, 72012 [Source:MGI Symbol;Acc:MGI:7826790]
5	lncRNA	1.19E+08	1.19E+08	ENSMUSG00000132438	MGI:7826778	Gm72006	predicted gene, 72006 [Source:MGI Symbol;Acc:MGI:7826778]
5	lncRNA	1.19E+08	1.19E+08	ENSMUSG00000106708	MGI:5663919	Gm43782	predicted gene 43782 [Source:MGI Symbol;Acc:MGI:5663919]
5	lncRNA	1.19E+08	1.19E+08	ENSMUSG00000106745	MGI:5663479	Gm43342	predicted gene 43342 [Source:MGI Symbol;Acc:MGI:5663479]
5	lncRNA	1.19E+08	1.19E+08	ENSMUSG00000107144	MGI:1921877	4930413E15Rik	RIKEN cDNA 4930413E15 gene [Source:MGI Symbol;Acc:MGI:1921877]
5	lncRNA	1.19E+08	1.19E+08	ENSMUSG00000142875	MGI:5590054	Gm30895	predicted gene, 30895 [Source:MGI Symbol;Acc:MGI:5590054]
5	lncRNA	1.19E+08	1.19E+08	ENSMUSG00000142923	MGI:5590193	Gm31034	predicted gene, 31034 [Source:MGI Symbol;Acc:MGI:5590193]
5	lncRNA	1.19E+08	1.19E+08	ENSMUSG00000143941	MGI:7830054	Gm73650	predicted gene, 73650 [Source:MGI Symbol;Acc:MGI:7830054]
5	lncRNA	1.19E+08	1.19E+08	ENSMUSG00000100573	MGI:1920727	1700081H04Rik	RIKEN cDNA 1700081H04 gene [Source:MGI Symbol;Acc:MGI:1920727]
5	lncRNA	1.19E+08	1.19E+08	ENSMUSG00000140902	MGI:7829242	Gm73242	predicted gene, 73242 [Source:MGI Symbol;Acc:MGI:7829242]
5	lncRNA	1.19E+08	1.19E+08	ENSMUSG00000140937	MGI:5590288	Gm31129	predicted gene, 31129 [Source:MGI Symbol;Acc:MGI:5590288]
5	lncRNA	1.19E+08	1.19E+08	ENSMUSG00000107334	MGI:3647618	Gm7538	predicted gene 7538 [Source:MGI Symbol;Acc:MGI:3647618]
5	lncRNA	1.19E+08	1.19E+08	ENSMUSG00000137673	MGI:7828464	Gm72851	predicted gene, 72851 [Source:MGI Symbol;Acc:MGI:7828464]
5	lncRNA	1.19E+08	1.19E+08	ENSMUSG00000137711	MGI:7828470	Gm72854	predicted gene, 72854 [Source:MGI Symbol;Acc:MGI:7828470]
5	lncRNA	1.19E+08	1.19E+08	ENSMUSG00000140558	MGI:7829190	Gm73216	predicted gene, 73216 [Source:MGI Symbol;Acc:MGI:7829190]

5	lncRNA	1.19E+08	1.19E+08	ENSMUSG00000133340	MGI:5590349	Gm31190	predicted gene, 31190 [Source:MGI Symbol;Acc:MGI:5590349]
5	lncRNA	1.19E+08	1.19E+08	ENSMUSG00000141471	MGI:7829392	Gm73317	predicted gene, 73317 [Source:MGI Symbol;Acc:MGI:7829392]
5	lncRNA	1.19E+08	1.19E+08	ENSMUSG00000120005	MGI:7338421	Gm57254	predicted gene, 57254 [Source:MGI Symbol;Acc:MGI:7338421]
5	lncRNA	1.19E+08	1.19E+08	ENSMUSG00000141549	MGI:5624901	Gm42016	predicted gene, 42016 [Source:MGI Symbol;Acc:MGI:5624901]
5	lncRNA	1.20E+08	1.20E+08	ENSMUSG00000143531	MGI:7829882	Gm73563	predicted gene, 73563 [Source:MGI Symbol;Acc:MGI:7829882]
5	lncRNA	1.20E+08	1.20E+08	ENSMUSG00000139730	MGI:7828944	Gm73093	predicted gene, 73093 [Source:MGI Symbol;Acc:MGI:7828944]
5	lncRNA	1.20E+08	1.20E+08	ENSMUSG00000106938	MGI:5590473	Gm31314	predicted gene, 31314 [Source:MGI Symbol;Acc:MGI:5590473]
5	lncRNA	1.20E+08	1.20E+08	ENSMUSG00000087516	MGI:3780472	Tbx3os1	T-box 3, opposite strand 1 [Source:MGI Symbol;Acc:MGI:3780472]
5	lncRNA	1.20E+08	1.20E+08	ENSMUSG00000085708	MGI:3801955	Gm16063	predicted gene 16063 [Source:MGI Symbol;Acc:MGI:3801955]
5	lncRNA	1.20E+08	1.20E+08	ENSMUSG00000140110	MGI:7829040	Gm73141	predicted gene, 73141 [Source:MGI Symbol;Acc:MGI:7829040]
5	lncRNA	1.20E+08	1.20E+08	ENSMUSG00000086847	MGI:3801954	Tbx3os2	T-box 3, opposite strand 2 [Source:MGI Symbol;Acc:MGI:3801954]
5	lncRNA	1.20E+08	1.20E+08	ENSMUSG00000127058	MGI:7825783	Gm71505	predicted gene, 71505 [Source:MGI Symbol;Acc:MGI:7825783]
5	lncRNA	1.20E+08	1.20E+08	ENSMUSG00000120759	MGI:7338493	Gm57290	predicted gene, 57290 [Source:MGI Symbol;Acc:MGI:7338493]
5	lncRNA	1.20E+08	1.20E+08	ENSMUSG00000106795	MGI:5663187	Gm43050	predicted gene 43050 [Source:MGI Symbol;Acc:MGI:5663187]
5	lncRNA	1.20E+08	1.20E+08	ENSMUSG00000132877	MGI:7826876	Gm72055	predicted gene, 72055 [Source:MGI Symbol;Acc:MGI:7826876]
5	lncRNA	1.20E+08	1.20E+08	ENSMUSG00000107084	MGI:5663406	Gm43269	predicted gene 43269 [Source:MGI Symbol;Acc:MGI:5663406]
5	lncRNA	1.20E+08	1.20E+08	ENSMUSG00000122811	MGI:5590932	Gm31773	predicted gene, 31773 [Source:MGI Symbol;Acc:MGI:5590932]
5	lncRNA	1.20E+08	1.20E+08	ENSMUSG00000107063	MGI:5621439	Gm38554	predicted gene, 38554 [Source:MGI Symbol;Acc:MGI:5621439]

5	lncRNA	1.20E+08	1.20E+08	ENSMUSG00000125535	MGI:7825631	Gm71429	predicted gene, 71429 [Source:MGI Symbol;Acc:MGI:7825631]
5	lncRNA	1.20E+08	1.20E+08	ENSMUSG00000132866	MGI:7826872	Gm72053	predicted gene, 72053 [Source:MGI Symbol;Acc:MGI:7826872]
5	lncRNA	1.20E+08	1.20E+08	ENSMUSG00000097368	MGI:3708725	Gm10390	predicted gene 10390 [Source:MGI Symbol;Acc:MGI:3708725]
5	lncRNA	1.20E+08	1.20E+08	ENSMUSG00000122430	MGI:7825265	Gm71242	predicted gene, 71242 [Source:MGI Symbol;Acc:MGI:7825265]
5	lncRNA	1.20E+08	1.20E+08	ENSMUSG00000122511	MGI:7825271	Gm71245	predicted gene, 71245 [Source:MGI Symbol;Acc:MGI:7825271]
5	lncRNA	1.20E+08	1.20E+08	ENSMUSG00000135011	MGI:7827568	Gm72401	predicted gene, 72401 [Source:MGI Symbol;Acc:MGI:7827568]
5	lncRNA	1.20E+08	1.20E+08	ENSMUSG00000140328	MGI:7829102	Gm73172	predicted gene, 73172 [Source:MGI Symbol;Acc:MGI:7829102]
5	lncRNA	1.20E+08	1.20E+08	ENSMUSG00000136990	MGI:7828250	Gm72744	predicted gene, 72744 [Source:MGI Symbol;Acc:MGI:7828250]
5	lncRNA	1.20E+08	1.21E+08	ENSMUSG00000107141	MGI:5662791	Gm42654	predicted gene 42654 [Source:MGI Symbol;Acc:MGI:5662791]
5	lncRNA	1.21E+08	1.21E+08	ENSMUSG00000098649	MGI:5521042	Lhx5as1	LIM homeobox 5, antisense 1 [Source:MGI Symbol;Acc:MGI:5521042]
5	lncRNA	1.21E+08	1.21E+08	ENSMUSG00000121101	MGI:7338535	Gm57311	predicted gene, 57311 [Source:MGI Symbol;Acc:MGI:7338535]
5	lncRNA	1.21E+08	1.21E+08	ENSMUSG00000086165	MGI:3783131	Gm15690	predicted gene 15690 [Source:MGI Symbol;Acc:MGI:3783131]
5	lncRNA	1.21E+08	1.21E+08	ENSMUSG00000142125	MGI:7829516	Gm73379	predicted gene, 73379 [Source:MGI Symbol;Acc:MGI:7829516]
5	lncRNA	1.21E+08	1.21E+08	ENSMUSG00000107191	MGI:5663716	Gm43579	predicted gene 43579 [Source:MGI Symbol;Acc:MGI:5663716]
5	lncRNA	1.21E+08	1.21E+08	ENSMUSG00000134485	MGI:7827452	Gm72343	predicted gene, 72343 [Source:MGI Symbol;Acc:MGI:7827452]
5	lncRNA	1.21E+08	1.21E+08	ENSMUSG00000089830	MGI:3783191	Gm15749	predicted gene 15749 [Source:MGI Symbol;Acc:MGI:3783191]
5	lncRNA	1.21E+08	1.21E+08	ENSMUSG00000128151	MGI:7825859	Gm71543	predicted gene, 71543 [Source:MGI Symbol;Acc:MGI:7825859]
5	lncRNA	1.21E+08	1.21E+08	ENSMUSG00000142087	MGI:7829494	Gm73368	predicted gene, 73368 [Source:MGI Symbol;Acc:MGI:7829494]

5	lncRNA	1.21E+08	1.21E+08	ENSMUSG00000107179	MGI:5663206	Gm43069	predicted gene 43069 [Source:MGI Symbol;Acc:MGI:5663206]
5	lncRNA	1.21E+08	1.21E+08	ENSMUSG00000141431	MGI:5591344	Gm32185	predicted gene, 32185 [Source:MGI Symbol;Acc:MGI:5591344]
5	lncRNA	1.21E+08	1.21E+08	ENSMUSG00000128029	MGI:7825849	Gm71538	predicted gene, 71538 [Source:MGI Symbol;Acc:MGI:7825849]
5	lncRNA	1.21E+08	1.21E+08	ENSMUSG00000143877	MGI:7830018	Gm73631	predicted gene, 73631 [Source:MGI Symbol;Acc:MGI:7830018]
5	lncRNA	1.21E+08	1.21E+08	ENSMUSG00000126312	MGI:7825711	Gm71469	predicted gene, 71469 [Source:MGI Symbol;Acc:MGI:7825711]
5	lncRNA	1.21E+08	1.21E+08	ENSMUSG00000126344	MGI:7825717	Gm71472	predicted gene, 71472 [Source:MGI Symbol;Acc:MGI:7825717]
5	lncRNA	1.21E+08	1.21E+08	ENSMUSG00000142544	MGI:7829620	Gm73432	predicted gene, 73432 [Source:MGI Symbol;Acc:MGI:7829620]
5	lncRNA	1.21E+08	1.21E+08	ENSMUSG00000120570	MGI:7338555	Gm57321	predicted gene, 57321 [Source:MGI Symbol;Acc:MGI:7338555]
5	lncRNA	1.22E+08	1.22E+08	ENSMUSG00000104985	MGI:5663054	Gm42917	predicted gene 42917 [Source:MGI Symbol;Acc:MGI:5663054]
5	lncRNA	1.22E+08	1.22E+08	ENSMUSG00000106209	MGI:5663055	Gm42918	predicted gene 42918 [Source:MGI Symbol;Acc:MGI:5663055]
5	lncRNA	1.22E+08	1.22E+08	ENSMUSG00000131095	MGI:7826388	Gm71811	predicted gene, 71811 [Source:MGI Symbol;Acc:MGI:7826388]
5	lncRNA	1.22E+08	1.22E+08	ENSMUSG00000126057	MGI:7825677	Gm71452	predicted gene, 71452 [Source:MGI Symbol;Acc:MGI:7825677]
5	lncRNA	1.22E+08	1.22E+08	ENSMUSG00000120376	MGI:7338527	Gm57307	predicted gene, 57307 [Source:MGI Symbol;Acc:MGI:7338527]
5	lncRNA	1.22E+08	1.22E+08	ENSMUSG00000130280	MGI:7826188	Gm71710	predicted gene, 71710 [Source:MGI Symbol;Acc:MGI:7826188]
5	lncRNA	1.22E+08	1.22E+08	ENSMUSG00000130243	MGI:7826168	Gm71700	predicted gene, 71700 [Source:MGI Symbol;Acc:MGI:7826168]
5	lncRNA	1.22E+08	1.22E+08	ENSMUSG00000120053	MGI:1925708	1700064N11Rik	RIKEN cDNA 1700064N11 gene [Source:MGI Symbol;Acc:MGI:1925708]
5	lncRNA	1.22E+08	1.22E+08	ENSMUSG00000089729	MGI:4414972	Gm16552	predicted gene 16552 [Source:MGI Symbol;Acc:MGI:4414972]
5	lncRNA	1.22E+08	1.22E+08	ENSMUSG00000122020	MGI:7825187	Gm71203	predicted gene, 71203 [Source:MGI Symbol;Acc:MGI:7825187]

5	lncRNA	1.22E+08	1.22E+08	ENSMUSG00000104872	MGI:1913571	1700008B11Rik	RIKEN cDNA 1700008B11 gene [Source:MGI Symbol;Acc:MGI:1913571]
5	lncRNA	1.22E+08	1.22E+08	ENSMUSG00000125798	MGI:7825647	Gm71437	predicted gene, 71437 [Source:MGI Symbol;Acc:MGI:7825647]
5	lncRNA	1.22E+08	1.22E+08	ENSMUSG00000140369	MGI:7829106	Gm73174	predicted gene, 73174 [Source:MGI Symbol;Acc:MGI:7829106]
5	lncRNA	1.22E+08	1.22E+08	ENSMUSG00000140332	MGI:5591581	Gm32422	predicted gene, 32422 [Source:MGI Symbol;Acc:MGI:5591581]
5	lncRNA	1.22E+08	1.22E+08	ENSMUSG00000131264	MGI:7826460	Gm71847	predicted gene, 71847 [Source:MGI Symbol;Acc:MGI:7826460]
5	lncRNA	1.22E+08	1.22E+08	ENSMUSG00000125571	MGI:7825637	Gm71432	predicted gene, 71432 [Source:MGI Symbol;Acc:MGI:7825637]
5	lncRNA	1.22E+08	1.22E+08	ENSMUSG00000105795	MGI:3782144	Gm3970	predicted gene 3970 [Source:MGI Symbol;Acc:MGI:3782144]
5	lncRNA	1.22E+08	1.22E+08	ENSMUSG00000087386	MGI:3783081	Gm15637	predicted gene 15637 [Source:MGI Symbol;Acc:MGI:3783081]
5	lncRNA	1.22E+08	1.22E+08	ENSMUSG00000138489	MGI:6367183	Gm52786	predicted gene, 52786 [Source:MGI Symbol;Acc:MGI:6367183]
5	lncRNA	1.22E+08	1.22E+08	ENSMUSG00000123198	MGI:7825373	Gm71298	predicted gene, 71298 [Source:MGI Symbol;Acc:MGI:7825373]
5	lncRNA	1.22E+08	1.22E+08	ENSMUSG00000120313	MGI:7338479	Gm57283	predicted gene, 57283 [Source:MGI Symbol;Acc:MGI:7338479]
5	lncRNA	1.22E+08	1.22E+08	ENSMUSG00000123162	MGI:7825362	Gm71292	predicted gene, 71292 [Source:MGI Symbol;Acc:MGI:7825362]
5	lncRNA	1.22E+08	1.22E+08	ENSMUSG00000123828	MGI:7825451	Gm71338	predicted gene, 71338 [Source:MGI Symbol;Acc:MGI:7825451]
5	lncRNA	1.22E+08	1.22E+08	ENSMUSG00000123867	MGI:7825453	Gm71339	predicted gene, 71339 [Source:MGI Symbol;Acc:MGI:7825453]
5	lncRNA	1.22E+08	1.22E+08	ENSMUSG00000143197	MGI:7829746	Gm73495	predicted gene, 73495 [Source:MGI Symbol;Acc:MGI:7829746]
5	lncRNA	1.22E+08	1.22E+08	ENSMUSG00000134979	MGI:7827560	Gm72397	predicted gene, 72397 [Source:MGI Symbol;Acc:MGI:7827560]
5	lncRNA	1.22E+08	1.22E+08	ENSMUSG00000125496	MGI:1920843	1700112N08Rik	RIKEN cDNA 1700112N08 gene [Source:MGI Symbol;Acc:MGI:1920843]
5	lncRNA	1.22E+08	1.22E+08	ENSMUSG00000125538	MGI:7825635	Gm71431	predicted gene, 71431 [Source:MGI Symbol;Acc:MGI:7825635]

5	lncRNA	1.22E+08	1.22E+08	ENSMUSG00000134697	MGI:7827492	Gm72363	predicted gene, 72363 [Source:MGI Symbol;Acc:MGI:7827492]
5	lncRNA	1.23E+08	1.23E+08	ENSMUSG00000122024	MGI:7825189	Gm71204	predicted gene, 71204 [Source:MGI Symbol;Acc:MGI:7825189]
5	lncRNA	1.23E+08	1.23E+08	ENSMUSG00000120495	MGI:7338443	Gm57265	predicted gene, 57265 [Source:MGI Symbol;Acc:MGI:7338443]
5	lncRNA	1.23E+08	1.23E+08	ENSMUSG00000105895	MGI:5662966	Gm42829	predicted gene 42829 [Source:MGI Symbol;Acc:MGI:5662966]
5	lncRNA	1.23E+08	1.23E+08	ENSMUSG00000133548	MGI:7827156	Gm72195	predicted gene, 72195 [Source:MGI Symbol;Acc:MGI:7827156]
5	lncRNA	1.23E+08	1.23E+08	ENSMUSG00000141355	MGI:7829382	Gm73312	predicted gene, 73312 [Source:MGI Symbol;Acc:MGI:7829382]
5	lncRNA	1.23E+08	1.23E+08	ENSMUSG00000120573	MGI:7338495	Gm57291	predicted gene, 57291 [Source:MGI Symbol;Acc:MGI:7338495]
5	lncRNA	1.23E+08	1.23E+08	ENSMUSG00000130488	MGI:1920518	1700040P09Rik	RIKEN cDNA 1700040P09 gene [Source:MGI Symbol;Acc:MGI:1920518]
5	lncRNA	1.23E+08	1.23E+08	ENSMUSG00000105430	MGI:5663438	Gm43301	predicted gene 43301 [Source:MGI Symbol;Acc:MGI:5663438]
5	lncRNA	1.23E+08	1.23E+08	ENSMUSG00000130609	MGI:7826250	Gm71741	predicted gene, 71741 [Source:MGI Symbol;Acc:MGI:7826250]
5	lncRNA	1.23E+08	1.23E+08	ENSMUSG00000132189	MGI:7826732	Gm71983	predicted gene, 71983 [Source:MGI Symbol;Acc:MGI:7826732]
5	lncRNA	1.23E+08	1.23E+08	ENSMUSG00000141260	MGI:5592147	Gm32988	predicted gene, 32988 [Source:MGI Symbol;Acc:MGI:5592147]
5	lncRNA	1.23E+08	1.23E+08	ENSMUSG00000129930	MGI:7826088	Gm71659	predicted gene, 71659 [Source:MGI Symbol;Acc:MGI:7826088]
5	lncRNA	1.23E+08	1.23E+08	ENSMUSG00000127177	MGI:7825795	Gm71511	predicted gene, 71511 [Source:MGI Symbol;Acc:MGI:7825795]
5	lncRNA	1.23E+08	1.23E+08	ENSMUSG00000122493	MGI:7825269	Gm71244	predicted gene, 71244 [Source:MGI Symbol;Acc:MGI:7825269]
5	lncRNA	1.23E+08	1.23E+08	ENSMUSG00000140346	MGI:7829104	Gm73173	predicted gene, 73173 [Source:MGI Symbol;Acc:MGI:7829104]
5	lncRNA	1.23E+08	1.23E+08	ENSMUSG00000136699	MGI:7828106	Gm72672	predicted gene, 72672 [Source:MGI Symbol;Acc:MGI:7828106]
5	lncRNA	1.23E+08	1.23E+08	ENSMUSG00000120987	MGI:5592203	Gm33044	predicted gene, 33044 [Source:MGI Symbol;Acc:MGI:5592203]

5	lncRNA	1.23E+08	1.23E+08	ENSMUSG00000142508	MGI:5592277	Gm33118	predicted gene, 33118 [Source:MGI Symbol;Acc:MGI:5592277]
5	lncRNA	1.23E+08	1.23E+08	ENSMUSG00000056735	MGI:1924414	A930024E05Rik	RIKEN cDNA A930024E05 gene [Source:MGI Symbol;Acc:MGI:1924414]
5	lncRNA	1.23E+08	1.23E+08	ENSMUSG00000105311	MGI:5753150	Gm44574	predicted gene 44574 [Source:MGI Symbol;Acc:MGI:5753150]
5	lncRNA	1.23E+08	1.23E+08	ENSMUSG00000142552	MGI:7829626	Gm73435	predicted gene, 73435 [Source:MGI Symbol;Acc:MGI:7829626]
5	lncRNA	1.23E+08	1.23E+08	ENSMUSG00000122704	MGI:7825301	Gm71260	predicted gene, 71260 [Source:MGI Symbol;Acc:MGI:7825301]
5	lncRNA	1.23E+08	1.23E+08	ENSMUSG00000106555	MGI:3780646	Gm2479	predicted gene 2479 [Source:MGI Symbol;Acc:MGI:3780646]
5	lncRNA	1.23E+08	1.23E+08	ENSMUSG00000131184	MGI:5624908	Gm42023	predicted gene, 42023 [Source:MGI Symbol;Acc:MGI:5624908]
5	lncRNA	1.23E+08	1.23E+08	ENSMUSG00000131154	MGI:7826420	Gm71827	predicted gene, 71827 [Source:MGI Symbol;Acc:MGI:7826420]
5	lncRNA	1.23E+08	1.23E+08	ENSMUSG00000141270	MGI:7829376	Gm73309	predicted gene, 73309 [Source:MGI Symbol;Acc:MGI:7829376]
5	lncRNA	1.23E+08	1.23E+08	ENSMUSG00000090086	MGI:2140910	AI480526	expressed sequence AI480526 [Source:MGI Symbol;Acc:MGI:2140910]
5	lncRNA	1.23E+08	1.23E+08	ENSMUSG00000104156	MGI:5611330	Gm38102	predicted gene, 38102 [Source:MGI Symbol;Acc:MGI:5611330]
5	lncRNA	1.23E+08	1.23E+08	ENSMUSG00000104606	MGI:5663546	Gm43409	predicted gene 43409 [Source:MGI Symbol;Acc:MGI:5663546]
5	lncRNA	1.23E+08	1.23E+08	ENSMUSG00000087063	MGI:3801821	Gm15857	predicted gene 15857 [Source:MGI Symbol;Acc:MGI:3801821]
5	lncRNA	1.23E+08	1.23E+08	ENSMUSG00000087336	MGI:3801778	Gm15860	predicted gene 15860 [Source:MGI Symbol;Acc:MGI:3801778]
5	lncRNA	1.24E+08	1.24E+08	ENSMUSG00000104830	MGI:1923368	5830487J09Rik	RIKEN cDNA 5830487J09 gene [Source:MGI Symbol;Acc:MGI:1923368]
5	lncRNA	1.24E+08	1.24E+08	ENSMUSG00000086700	MGI:3783189	Gm15747	predicted gene 15747 [Source:MGI Symbol;Acc:MGI:3783189]
5	lncRNA	1.24E+08	1.24E+08	ENSMUSG00000106636	MGI:5663950	Gm43813	predicted gene 43813 [Source:MGI Symbol;Acc:MGI:5663950]
5	lncRNA	1.24E+08	1.24E+08	ENSMUSG00000086753	MGI:3783193	Gm15751	predicted gene 15751 [Source:MGI Symbol;Acc:MGI:3783193]

5	lncRNA	1.24E+08	1.24E+08	ENSMUSG00000135480	MGI:7827704	Gm72470	predicted gene, 72470 [Source:MGI Symbol;Acc:MGI:7827704]
5	lncRNA	1.24E+08	1.24E+08	ENSMUSG00000125294	MGI:7825607	Gm71417	predicted gene, 71417 [Source:MGI Symbol;Acc:MGI:7825607]
5	lncRNA	1.24E+08	1.24E+08	ENSMUSG00000142870	MGI:7829678	Gm73461	predicted gene, 73461 [Source:MGI Symbol;Acc:MGI:7829678]
5	lncRNA	1.24E+08	1.24E+08	ENSMUSG00000140043	MGI:7829032	Gm73137	predicted gene, 73137 [Source:MGI Symbol;Acc:MGI:7829032]
5	lncRNA	1.24E+08	1.24E+08	ENSMUSG00000142808	MGI:7829672	Gm73458	predicted gene, 73458 [Source:MGI Symbol;Acc:MGI:7829672]
5	lncRNA	1.24E+08	1.24E+08	ENSMUSG00000106261	MGI:5593245	Gm34086	predicted gene, 34086 [Source:MGI Symbol;Acc:MGI:5593245]
5	lncRNA	1.24E+08	1.24E+08	ENSMUSG00000128052	MGI:7825851	Gm71539	predicted gene, 71539 [Source:MGI Symbol;Acc:MGI:7825851]
5	lncRNA	1.24E+08	1.24E+08	ENSMUSG00000104818	MGI:5663798	Gm43661	predicted gene 43661 [Source:MGI Symbol;Acc:MGI:5663798]
5	lncRNA	1.24E+08	1.24E+08	ENSMUSG00000126658	MGI:7825741	Gm71484	predicted gene, 71484 [Source:MGI Symbol;Acc:MGI:7825741]
5	lncRNA	1.24E+08	1.24E+08	ENSMUSG00000128265	MGI:7825867	Gm71547	predicted gene, 71547 [Source:MGI Symbol;Acc:MGI:7825867]
5	lncRNA	1.24E+08	1.24E+08	ENSMUSG00000085433	MGI:3802119	Gm16001	predicted gene 16001 [Source:MGI Symbol;Acc:MGI:3802119]
5	lncRNA	1.24E+08	1.24E+08	ENSMUSG00000132124	MGI:7826716	Gm71975	predicted gene, 71975 [Source:MGI Symbol;Acc:MGI:7826716]
5	lncRNA	1.24E+08	1.24E+08	ENSMUSG00000089842	MGI:3840147	Pitpnm2os2	phosphatidylinositol transfer protein, membrane-associated 2, opposite strand 2 [Source:MGI Symbol;Acc:MGI:3840147]
5	lncRNA	1.24E+08	1.24E+08	ENSMUSG00000133564	MGI:7827160	Gm72197	predicted gene, 72197 [Source:MGI Symbol;Acc:MGI:7827160]
5	lncRNA	1.24E+08	1.24E+08	ENSMUSG00000090220	MGI:1923177	Pitpnm2os1	phosphatidylinositol transfer protein, membrane-associated 2, opposite strand 1 [Source:MGI Symbol;Acc:MGI:1923177]
5	lncRNA	1.24E+08	1.24E+08	ENSMUSG00000105759	MGI:5662562	Gm42425	predicted gene 42425 [Source:MGI Symbol;Acc:MGI:5662562]
5	lncRNA	1.25E+08	1.25E+08	ENSMUSG00000126538	MGI:7831160	Gm74211	predicted gene, 74211 [Source:MGI Symbol;Acc:MGI:7831160]

5	lncRNA	1.25E+08	1.25E+08	ENSMUSG00000136903	MGI:7828228	Gm72733	predicted gene, 72733 [Source:MGI Symbol;Acc:MGI:7828228]
5	lncRNA	1.25E+08	1.25E+08	ENSMUSG00000144115	MGI:7830126	Gm73688	predicted gene, 73688 [Source:MGI Symbol;Acc:MGI:7830126]
5	lncRNA	1.25E+08	1.25E+08	ENSMUSG00000144077	MGI:7830124	Gm73687	predicted gene, 73687 [Source:MGI Symbol;Acc:MGI:7830124]
5	lncRNA	1.25E+08	1.25E+08	ENSMUSG00000144148	MGI:6367189	Gm52789	predicted gene, 52789 [Source:MGI Symbol;Acc:MGI:6367189]
5	lncRNA	1.25E+08	1.25E+08	ENSMUSG00000144043	MGI:7830116	Gm73683	predicted gene, 73683 [Source:MGI Symbol;Acc:MGI:7830116]
5	lncRNA	1.25E+08	1.25E+08	ENSMUSG00000144003	MGI:7830082	Gm73664	predicted gene, 73664 [Source:MGI Symbol;Acc:MGI:7830082]
5	lncRNA	1.25E+08	1.25E+08	ENSMUSG00000106082	MGI:3605803	4930404A12Rik	RIKEN cDNA 4930404A12 gene [Source:MGI Symbol;Acc:MGI:3605803]
5	lncRNA	1.25E+08	1.25E+08	ENSMUSG00000105583	MGI:5663914	Gm43777	predicted gene 43777 [Source:MGI Symbol;Acc:MGI:5663914]
5	lncRNA	1.25E+08	1.25E+08	ENSMUSG00000125796	MGI:5623206	Gm40321	predicted gene, 40321 [Source:MGI Symbol;Acc:MGI:5623206]
5	lncRNA	1.25E+08	1.25E+08	ENSMUSG00000126937	MGI:5591688	Gm32529	predicted gene, 32529 [Source:MGI Symbol;Acc:MGI:5591688]
5	lncRNA	1.25E+08	1.25E+08	ENSMUSG00000105604	MGI:5662975	Gm42838	predicted gene 42838 [Source:MGI Symbol;Acc:MGI:5662975]
5	lncRNA	1.25E+08	1.25E+08	ENSMUSG00000106663	MGI:5662976	Gm42839	predicted gene 42839 [Source:MGI Symbol;Acc:MGI:5662976]
5	lncRNA	1.25E+08	1.25E+08	ENSMUSG00000138261	MGI:7828552	Gm72896	predicted gene, 72896 [Source:MGI Symbol;Acc:MGI:7828552]
5	lncRNA	1.25E+08	1.25E+08	ENSMUSG00000105039	MGI:5591744	Gm32585	predicted gene, 32585 [Source:MGI Symbol;Acc:MGI:5591744]
5	lncRNA	1.25E+08	1.25E+08	ENSMUSG00000131642	MGI:7826598	Gm71916	predicted gene, 71916 [Source:MGI Symbol;Acc:MGI:7826598]
5	lncRNA	1.25E+08	1.25E+08	ENSMUSG00000131568	MGI:7826572	Gm71903	predicted gene, 71903 [Source:MGI Symbol;Acc:MGI:7826572]
5	lncRNA	1.25E+08	1.25E+08	ENSMUSG00000131271	MGI:5591860	Gm32701	predicted gene, 32701 [Source:MGI Symbol;Acc:MGI:5591860]
5	lncRNA	1.25E+08	1.25E+08	ENSMUSG00000131428	MGI:7831178	Gm74220	predicted gene, 74220 [Source:MGI Symbol;Acc:MGI:7831178]

5	lncRNA	1.25E+08	1.25E+08	ENSMUSG00000131347	MGI:7826504	Gm71869	predicted gene, 71869 [Source:MGI Symbol;Acc:MGI:7826504]
5	lncRNA	1.25E+08	1.25E+08	ENSMUSG00000131305	MGI:7826496	Gm71865	predicted gene, 71865 [Source:MGI Symbol;Acc:MGI:7826496]
5	lncRNA	1.25E+08	1.25E+08	ENSMUSG00000131385	MGI:7826506	Gm71870	predicted gene, 71870 [Source:MGI Symbol;Acc:MGI:7826506]
5	lncRNA	1.25E+08	1.25E+08	ENSMUSG00000104932	MGI:5623208	Gm40323	predicted gene, 40323 [Source:MGI Symbol;Acc:MGI:5623208]
5	lncRNA	1.25E+08	1.25E+08	ENSMUSG00000131467	MGI:7826510	Gm71872	predicted gene, 71872 [Source:MGI Symbol;Acc:MGI:7826510]
5	lncRNA	1.25E+08	1.25E+08	ENSMUSG00000072612	MGI:3647829	Gm10382	predicted gene 10382 [Source:MGI Symbol;Acc:MGI:3647829]
5	lncRNA	1.25E+08	1.25E+08	ENSMUSG00000131682	MGI:7826606	Gm71920	predicted gene, 71920 [Source:MGI Symbol;Acc:MGI:7826606]
5	lncRNA	1.25E+08	1.25E+08	ENSMUSG00000120357	MGI:7338455	Gm57271	predicted gene, 57271 [Source:MGI Symbol;Acc:MGI:7338455]
5	lncRNA	1.25E+08	1.25E+08	ENSMUSG00000120435	MGI:7338463	Gm57275	predicted gene, 57275 [Source:MGI Symbol;Acc:MGI:7338463]
5	lncRNA	1.25E+08	1.25E+08	ENSMUSG00000136838	MGI:7828190	Gm72714	predicted gene, 72714 [Source:MGI Symbol;Acc:MGI:7828190]
5	lncRNA	1.25E+08	1.25E+08	ENSMUSG00000132160	MGI:7826724	Gm71979	predicted gene, 71979 [Source:MGI Symbol;Acc:MGI:7826724]
5	lncRNA	1.26E+08	1.26E+08	ENSMUSG00000130692	MGI:7826260	Gm71746	predicted gene, 71746 [Source:MGI Symbol;Acc:MGI:7826260]
5	lncRNA	1.26E+08	1.26E+08	ENSMUSG00000130651	MGI:7830302	Gm73778	predicted gene, 73778 [Source:MGI Symbol;Acc:MGI:7830302]
5	lncRNA	1.26E+08	1.26E+08	ENSMUSG00000130725	MGI:7826262	Gm71747	predicted gene, 71747 [Source:MGI Symbol;Acc:MGI:7826262]
5	lncRNA	1.26E+08	1.26E+08	ENSMUSG00000122318	MGI:5623210	Gm40325	predicted gene, 40325 [Source:MGI Symbol;Acc:MGI:5623210]
5	lncRNA	1.26E+08	1.26E+08	ENSMUSG00000133223	MGI:7827072	Gm72153	predicted gene, 72153 [Source:MGI Symbol;Acc:MGI:7827072]
5	lncRNA	1.26E+08	1.26E+08	ENSMUSG00000133268	MGI:7827090	Gm72162	predicted gene, 72162 [Source:MGI Symbol;Acc:MGI:7827090]
5	lncRNA	1.26E+08	1.26E+08	ENSMUSG00000131744	MGI:7826616	Gm71925	predicted gene, 71925 [Source:MGI Symbol;Acc:MGI:7826616]

5	lncRNA	1.26E+08	1.26E+08	ENSMUSG00000129713	MGI:7826019	Gm71624	predicted gene, 71624 [Source:MGI Symbol;Acc:MGI:7826019]
5	lncRNA	1.26E+08	1.26E+08	ENSMUSG00000129756	MGI:5623212	Gm40327	predicted gene, 40327 [Source:MGI Symbol;Acc:MGI:5623212]
5	lncRNA	1.26E+08	1.26E+08	ENSMUSG00000129792	MGI:7826031	Gm71630	predicted gene, 71630 [Source:MGI Symbol;Acc:MGI:7826031]
5	lncRNA	1.26E+08	1.26E+08	ENSMUSG00000129826	MGI:7830304	Gm73779	predicted gene, 73779 [Source:MGI Symbol;Acc:MGI:7830304]
5	lncRNA	1.26E+08	1.26E+08	ENSMUSG00000142010	MGI:5623213	Gm40328	predicted gene, 40328 [Source:MGI Symbol;Acc:MGI:5623213]
5	lncRNA	1.26E+08	1.26E+08	ENSMUSG00000131107	MGI:7826394	Gm71814	predicted gene, 71814 [Source:MGI Symbol;Acc:MGI:7826394]
5	miRNA	42048685	42048766	ENSMUSG00000098264	MGI:5530900	Mir6414	microRNA 6414 [Source:MGI Symbol;Acc:MGI:5530900]
5	miRNA	48381284	48381393	ENSMUSG00000065603	MGI:3618751	Mir218-1	microRNA 218-1 [Source:MGI Symbol;Acc:MGI:3618751]
5	miRNA	50410104	50410210	ENSMUSG00000099124	MGI:5530705	Mir8117	microRNA 8117 [Source:MGI Symbol;Acc:MGI:5530705]
5	miRNA	51380930	51381016	ENSMUSG00002076511	MGI:6848422	Gm55981	predicted gene, 55981 [Source:MGI Symbol;Acc:MGI:6848422]
5	miRNA	52293244	52293359	ENSMUSG00000098726	MGI:5530716	Mir6417	microRNA 6417 [Source:MGI Symbol;Acc:MGI:5530716]
5	miRNA	64258773	64258850	ENSMUSG00002076449	MGI:6848597	Gm56069	predicted gene, 56069 [Source:MGI Symbol;Acc:MGI:6848597]
5	miRNA	64264750	64264845	ENSMUSG00002076429	MGI:6848599	Gm56070	predicted gene, 56070 [Source:MGI Symbol;Acc:MGI:6848599]
5	miRNA	64804976	64805092	ENSMUSG00002074850	MGI:6848635	Gm56088	predicted gene, 56088 [Source:MGI Symbol;Acc:MGI:6848635]
5	miRNA	65127661	65127738	ENSMUSG00000077042	MGI:3718549	Mir574	microRNA 574 [Source:MGI Symbol;Acc:MGI:3718549]
5	miRNA	66146388	66146461	ENSMUSG00002076702	MGI:6848728	Gm56135	predicted gene, 56135 [Source:MGI Symbol;Acc:MGI:6848728]
5	miRNA	67211022	67211135	ENSMUSG00002075048	MGI:6848788	Gm56165	predicted gene, 56165 [Source:MGI Symbol;Acc:MGI:6848788]
5	miRNA	67789168	67789262	ENSMUSG00002076306	MGI:6846111	Gm54817	predicted gene, 54817 [Source:MGI Symbol;Acc:MGI:6846111]

5	miRNA	69498126	69498231	ENSMUSG00002076624	MGI:6846134	Gm54829	predicted gene, 54829 [Source:MGI Symbol;Acc:MGI:6846134]
5	miRNA	75337492	75337565	ENSMUSG00000099060	MGI:5531242	Mir7025	microRNA 7025 [Source:MGI Symbol;Acc:MGI:5531242]
5	miRNA	76070487	76070587	ENSMUSG00002075712	MGI:6847257	Gm55393	predicted gene, 55393 [Source:MGI Symbol;Acc:MGI:6847257]
5	miRNA	77420604	77420685	ENSMUSG00000093061	MGI:4950415	Mir5098	microRNA 5098 [Source:MGI Symbol;Acc:MGI:4950415]
5	miRNA	81883774	81883862	ENSMUSG00002074859	MGI:6848072	Gm55803	predicted gene, 55803 [Source:MGI Symbol;Acc:MGI:6848072]
5	miRNA	82946791	82946912	ENSMUSG00000080586	MGI:3783361	Mir1187	microRNA 1187 [Source:MGI Symbol;Acc:MGI:3783361]
5	miRNA	83612641	83612723	ENSMUSG00002076941	MGI:6848080	Gm55807	predicted gene, 55807 [Source:MGI Symbol;Acc:MGI:6848080]
5	miRNA	87743761	87743852	ENSMUSG00000106074	MGI:4950408	Mir3969	microRNA 3969 [Source:MGI Symbol;Acc:MGI:4950408]
5	miRNA	90944045	90944114	ENSMUSG00002076065	MGI:6848412	Gm55976	predicted gene, 55976 [Source:MGI Symbol;Acc:MGI:6848412]
5	miRNA	92606416	92606515	ENSMUSG00000099160	MGI:5531096	Mir6415	microRNA 6415 [Source:MGI Symbol;Acc:MGI:5531096]
5	miRNA	92936310	92936421	ENSMUSG00000089268	MGI:3837206	Mir1961	microRNA 1961 [Source:MGI Symbol;Acc:MGI:3837206]
5	miRNA	98623415	98623523	ENSMUSG00000105814	MGI:3629657	Mir703	microRNA 703 [Source:MGI Symbol;Acc:MGI:3629657]
5	miRNA	1.01E+08	1.01E+08	ENSMUSG00002076564	MGI:6849020	Gm56281	predicted gene, 56281 [Source:MGI Symbol;Acc:MGI:6849020]
5	miRNA	1.04E+08	1.04E+08	ENSMUSG00000093690	MGI:5453643	Mir5619	microRNA 5619 [Source:MGI Symbol;Acc:MGI:5453643]
5	miRNA	1.04E+08	1.04E+08	ENSMUSG00002076521	MGI:6846437	Gm54981	predicted gene, 54981 [Source:MGI Symbol;Acc:MGI:6846437]
5	miRNA	1.07E+08	1.07E+08	ENSMUSG00002075799	MGI:6846624	Gm55075	predicted gene, 55075 [Source:MGI Symbol;Acc:MGI:6846624]
5	miRNA	1.11E+08	1.11E+08	ENSMUSG00000099284	MGI:5531370	Mir7026	microRNA 7026 [Source:MGI Symbol;Acc:MGI:5531370]
5	miRNA	1.11E+08	1.11E+08	ENSMUSG00000076163	MGI:3629658	Mir701	microRNA 701 [Source:MGI Symbol;Acc:MGI:3629658]

5	miRNA	1.13E+08	1.13E+08	ENSMUSG00000105778	MGI:5562765	Mir7229	microRNA 7229 [Source:MGI Symbol;Acc:MGI:5562765]
5	miRNA	1.13E+08	1.13E+08	ENSMUSG00000104840	MGI:5562766	Mir7230	microRNA 7230 [Source:MGI Symbol;Acc:MGI:5562766]
5	miRNA	1.15E+08	1.15E+08	ENSMUSG00000098731	MGI:5531213	Mir7027	microRNA 7027 [Source:MGI Symbol;Acc:MGI:5531213]
5	miRNA	1.15E+08	1.15E+08	ENSMUSG00000098656	MGI:5531056	Mir7028	microRNA 7028 [Source:MGI Symbol;Acc:MGI:5531056]
5	miRNA	1.15E+08	1.15E+08	ENSMUSG00000098343	MGI:5531232	Mir6240	microRNA 6240 [Source:MGI Symbol;Acc:MGI:5531232]
5	miRNA	1.16E+08	1.16E+08	ENSMUSG00000099047	MGI:5530675	Mir7029	microRNA 7029 [Source:MGI Symbol;Acc:MGI:5530675]
5	miRNA	1.16E+08	1.16E+08	ENSMUSG00000099254	MGI:5530649	Mir7030	microRNA 7030 [Source:MGI Symbol;Acc:MGI:5530649]
5	miRNA	1.19E+08	1.19E+08	ENSMUSG00002075849	MGI:6848605	Gm56073	predicted gene, 56073 [Source:MGI Symbol;Acc:MGI:6848605]
5	miRNA	1.20E+08	1.20E+08	ENSMUSG00002075229	MGI:6848665	Gm56103	predicted gene, 56103 [Source:MGI Symbol;Acc:MGI:6848665]
5	miRNA	1.21E+08	1.21E+08	ENSMUSG00002076672	MGI:6848705	Gm56123	predicted gene, 56123 [Source:MGI Symbol;Acc:MGI:6848705]
5	miRNA	1.22E+08	1.22E+08	ENSMUSG00000099063	MGI:5531241	Mir7031	microRNA 7031 [Source:MGI Symbol;Acc:MGI:5531241]
5	miRNA	1.23E+08	1.23E+08	ENSMUSG00000099324	MGI:5531394	Mir8115	microRNA 8115 [Source:MGI Symbol;Acc:MGI:5531394]
5	miRNA	1.24E+08	1.24E+08	ENSMUSG00000098951	MGI:5530729	Mir7647	microRNA 7647 [Source:MGI Symbol;Acc:MGI:5530729]
5	miRNA	1.24E+08	1.24E+08	ENSMUSG00000098454	MGI:5530691	Mir7032	microRNA 7032 [Source:MGI Symbol;Acc:MGI:5530691]
5	miRNA	1.25E+08	1.25E+08	ENSMUSG00002076658	MGI:6848996	Gm56269	predicted gene, 56269 [Source:MGI Symbol;Acc:MGI:6848996]
5	miRNA	1.26E+08	1.26E+08	ENSMUSG00002076951	MGI:6848100	Gm55817	predicted gene, 55817 [Source:MGI Symbol;Acc:MGI:6848100]
5	misc_RNA	66483083	66483405	ENSMUSG00002076874	MGI:6848760	Gm56151	predicted gene, 56151 [Source:MGI Symbol;Acc:MGI:6848760]
5	misc_RNA	79786662	79786956	ENSMUSG00002075735	MGI:6847501	Gm55516	predicted gene, 55516 [Source:MGI Symbol;Acc:MGI:6847501]

5	misc_RNA	89829775	89830098	ENSMUSG00002075998	MGI:6848366	Gm55953	predicted gene, 55953 [Source:MGI Symbol;Acc:MGI:6848366]
5	misc_RNA	92130564	92130839	ENSMUSG00002075701	MGI:6848420	Gm55980	predicted gene, 55980 [Source:MGI Symbol;Acc:MGI:6848420]
5	misc_RNA	1.01E+08	1.01E+08	ENSMUSG00002076301	MGI:6849012	Gm56277	predicted gene, 56277 [Source:MGI Symbol;Acc:MGI:6849012]
5	misc_RNA	1.01E+08	1.01E+08	ENSMUSG00002076403	MGI:6849014	Gm56278	predicted gene, 56278 [Source:MGI Symbol;Acc:MGI:6849014]
5	misc_RNA	1.01E+08	1.01E+08	ENSMUSG00002075475	MGI:6849016	Gm56279	predicted gene, 56279 [Source:MGI Symbol;Acc:MGI:6849016]
5	misc_RNA	1.01E+08	1.01E+08	ENSMUSG00002075511	MGI:6849018	Gm56280	predicted gene, 56280 [Source:MGI Symbol;Acc:MGI:6849018]
5	misc_RNA	1.04E+08	1.04E+08	ENSMUSG00002075219	MGI:6846311	Gm54918	predicted gene, 54918 [Source:MGI Symbol;Acc:MGI:6846311]
5	misc_RNA	1.10E+08	1.10E+08	ENSMUSG00002075066	MGI:6847414	Gm55472	predicted gene, 55472 [Source:MGI Symbol;Acc:MGI:6847414]
5	misc_RNA	1.11E+08	1.11E+08	ENSMUSG00002076336	MGI:6847805	Gm55669	predicted gene, 55669 [Source:MGI Symbol;Acc:MGI:6847805]
5	misc_RNA	1.11E+08	1.11E+08	ENSMUSG00002075664	MGI:6847807	Gm55670	predicted gene, 55670 [Source:MGI Symbol;Acc:MGI:6847807]
5	misc_RNA	1.12E+08	1.12E+08	ENSMUSG00002074930	MGI:6847823	Gm55678	predicted gene, 55678 [Source:MGI Symbol;Acc:MGI:6847823]
5	misc_RNA	1.12E+08	1.12E+08	ENSMUSG00002076466	MGI:6847825	Gm55679	predicted gene, 55679 [Source:MGI Symbol;Acc:MGI:6847825]
5	misc_RNA	1.12E+08	1.12E+08	ENSMUSG00002075414	MGI:6847827	Gm55680	predicted gene, 55680 [Source:MGI Symbol;Acc:MGI:6847827]
5	misc_RNA	1.12E+08	1.12E+08	ENSMUSG00002075709	MGI:6847837	Gm55685	predicted gene, 55685 [Source:MGI Symbol;Acc:MGI:6847837]
5	misc_RNA	1.18E+08	1.18E+08	ENSMUSG00002075291	MGI:6848601	Gm56071	predicted gene, 56071 [Source:MGI Symbol;Acc:MGI:6848601]
5	misc_RNA	1.24E+08	1.24E+08	ENSMUSG00002075826	MGI:6847579	Gm55555	predicted gene, 55555 [Source:MGI Symbol;Acc:MGI:6847579]
5	processed_pseudogene	41499466	41500274	ENSMUSG00000106895	MGI:3645925	Gm4754	predicted gene 4754 [Source:MGI Symbol;Acc:MGI:3645925]

5	processed_pseudogene	41578919	41579387	ENSMUSG00000107135	MGI:5663943	Gm43806	predicted gene 43806 [Source:MGI Symbol;Acc:MGI:5663943]
5	processed_pseudogene	42010653	42011544	ENSMUSG00000107259	MGI:3645526	Gm5298	predicted gene 5298 [Source:MGI Symbol;Acc:MGI:3645526]
5	processed_pseudogene	42091740	42093015	ENSMUSG00000106884	MGI:5012341	Gm20156	predicted gene, 20156 [Source:MGI Symbol;Acc:MGI:5012341]
5	processed_pseudogene	42657324	42657757	ENSMUSG00000106813	MGI:3644423	Gm7181	predicted gene 7181 [Source:MGI Symbol;Acc:MGI:3644423]
5	processed_pseudogene	43120747	43121952	ENSMUSG00000106622	MGI:3648967	Gm5554	predicted gene 5554 [Source:MGI Symbol;Acc:MGI:3648967]
5	processed_pseudogene	44074095	44075856	ENSMUSG00000093859	MGI:3647603	Gm7882	predicted gene 7882 [Source:MGI Symbol;Acc:MGI:3647603]
5	processed_pseudogene	44512203	44514096	ENSMUSG00000106394	MGI:5662835	Gm42698	predicted gene 42698 [Source:MGI Symbol;Acc:MGI:5662835]
5	processed_pseudogene	44563299	44563935	ENSMUSG00000104923	MGI:3645504	Gm5865	predicted gene 5865 [Source:MGI Symbol;Acc:MGI:3645504]
5	processed_pseudogene	47791844	47792648	ENSMUSG00000106402	MGI:5010009	Gm17824	predicted gene, 17824 [Source:MGI Symbol;Acc:MGI:5010009]
5	processed_pseudogene	47857187	47857634	ENSMUSG00000105616	MGI:3781188	Gm3010	predicted gene 3010 [Source:MGI Symbol;Acc:MGI:3781188]
5	processed_pseudogene	48132347	48132677	ENSMUSG00000105994	MGI:5663564	Gm43427	predicted gene 43427 [Source:MGI Symbol;Acc:MGI:5663564]
5	processed_pseudogene	48823247	48824611	ENSMUSG00000093748	MGI:5011216	Gm19031	predicted gene, 19031 [Source:MGI Symbol;Acc:MGI:5011216]

5	processed_pseudogene	49581184	49581496	ENSMUSG00000093490	MGI:5012117	Gm19932	predicted gene, 19932 [Source:MGI Symbol;Acc:MGI:5012117]
5	processed_pseudogene	49709500	49710610	ENSMUSG00000083477	MGI:3648970	Gm5555	predicted pseudogene 5555 [Source:MGI Symbol;Acc:MGI:3648970]
5	processed_pseudogene	50057348	50059644	ENSMUSG00000105540	MGI:3649096	Gm7988	predicted gene 7988 [Source:MGI Symbol;Acc:MGI:3649096]
5	processed_pseudogene	51078684	51079248	ENSMUSG00000105273	MGI:5663152	Gm43015	predicted gene 43015 [Source:MGI Symbol;Acc:MGI:5663152]
5	processed_pseudogene	51209723	51210203	ENSMUSG00000106267	MGI:5623204	Gm40319	predicted gene, 40319 [Source:MGI Symbol;Acc:MGI:5623204]
5	processed_pseudogene	52739416	52740388	ENSMUSG00000095078	MGI:3645339	Gm5866	predicted gene 5866 [Source:MGI Symbol;Acc:MGI:3645339]
5	processed_pseudogene	53074961	53076551	ENSMUSG00000105054	MGI:5663801	Gm43664	predicted gene 43664 [Source:MGI Symbol;Acc:MGI:5663801]
5	processed_pseudogene	54251345	54252422	ENSMUSG00000107105	MGI:3801941	Gm15820	predicted gene 15820 [Source:MGI Symbol;Acc:MGI:3801941]
5	processed_pseudogene	54727247	54735354	ENSMUSG00000106529	MGI:5010636	Gm18451	predicted gene, 18451 [Source:MGI Symbol;Acc:MGI:5010636]
5	processed_pseudogene	54809310	54809463	ENSMUSG00000105143	MGI:3645115	Gm8069	predicted pseudogene 8069 [Source:MGI Symbol;Acc:MGI:3645115]
5	processed_pseudogene	55153962	55154790	ENSMUSG00000106113	MGI:5663916	Gm43779	predicted gene 43779 [Source:MGI Symbol;Acc:MGI:5663916]
5	processed_pseudogene	55740987	55741692	ENSMUSG00000105384	MGI:5663533	Gm43396	predicted gene 43396 [Source:MGI Symbol;Acc:MGI:5663533]

5	processed_pseudogene	55741836	55742142	ENSMUSG00000105551	MGI:5663336	Gm43199	predicted gene 43199 [Source:MGI Symbol;Acc:MGI:5663336]
5	processed_pseudogene	55781532	55782933	ENSMUSG00000083902	MGI:3801944	Tent2-ps1	terminal nucleotidyltransferase 2, pseudogene 1 [Source:MGI Symbol;Acc:MGI:3801944]
5	processed_pseudogene	55879959	55880763	ENSMUSG00000106335	MGI:5012408	Gm20223	predicted gene, 20223 [Source:MGI Symbol;Acc:MGI:5012408]
5	processed_pseudogene	56314680	56314992	ENSMUSG00000105685	MGI:5663452	Gm43315	predicted gene 43315 [Source:MGI Symbol;Acc:MGI:5663452]
5	processed_pseudogene	57386608	57387957	ENSMUSG00000104739	MGI:3781461	Gm3283	predicted gene 3283 [Source:MGI Symbol;Acc:MGI:3781461]
5	processed_pseudogene	57418614	57419349	ENSMUSG00000104560	MGI:3648794	Gm8115	predicted gene 8115 [Source:MGI Symbol;Acc:MGI:3648794]
5	processed_pseudogene	57513177	57514620	ENSMUSG00000105291	MGI:5011086	Gm18901	predicted gene, 18901 [Source:MGI Symbol;Acc:MGI:5011086]
5	processed_pseudogene	57617502	57618132	ENSMUSG00000104892	MGI:3644221	Gm6615	predicted gene 6615 [Source:MGI Symbol;Acc:MGI:3644221]
5	processed_pseudogene	57765911	57766409	ENSMUSG00000051116	MGI:3645325	Gm8121	predicted pseudogene 8121 [Source:MGI Symbol;Acc:MGI:3645325]
5	processed_pseudogene	58306965	58307186	ENSMUSG00000105525	MGI:5663113	Gm42976	predicted gene 42976 [Source:MGI Symbol;Acc:MGI:5663113]
5	processed_pseudogene	59157643	59158336	ENSMUSG00000106509	MGI:5663531	Gm43394	predicted gene 43394 [Source:MGI Symbol;Acc:MGI:5663531]
5	processed_pseudogene	59210942	59211895	ENSMUSG00000094696	MGI:3644251	Gm6632	predicted pseudogene 6632 [Source:MGI Symbol;Acc:MGI:3644251]

5	processed_pseudogene	59669308	59669686	ENSMUSG00000105038	MGI:5663181	Gm43044	predicted gene 43044 [Source:MGI Symbol;Acc:MGI:5663181]
5	processed_pseudogene	59935255	59935666	ENSMUSG00000104889	MGI:5663180	Gm43043	predicted gene 43043 [Source:MGI Symbol;Acc:MGI:5663180]
5	processed_pseudogene	60182239	60182672	ENSMUSG00000106250	MGI:5663529	Gm43392	predicted gene 43392 [Source:MGI Symbol;Acc:MGI:5663529]
5	processed_pseudogene	60183033	60183226	ENSMUSG00000106170	MGI:5663530	Gm43393	predicted gene 43393 [Source:MGI Symbol;Acc:MGI:5663530]
5	processed_pseudogene	60333259	60333892	ENSMUSG00000106042	MGI:5662998	Gm42861	predicted gene 42861 [Source:MGI Symbol;Acc:MGI:5662998]
5	processed_pseudogene	60388826	60389555	ENSMUSG00000087034	MGI:1333788	Cbfa2t2-ps1	CBFA2/RUNX1 translocation partner 2, pseudogene 1 [Source:MGI Symbol;Acc:MGI:1333788]
5	processed_pseudogene	60735973	60736393	ENSMUSG00000106548	MGI:5663527	Gm43390	predicted gene 43390 [Source:MGI Symbol;Acc:MGI:5663527]
5	processed_pseudogene	60831660	60833451	ENSMUSG00000105486	MGI:3781550	Gm3372	predicted gene 3372 [Source:MGI Symbol;Acc:MGI:3781550]
5	processed_pseudogene	60979002	60979770	ENSMUSG00000104775	MGI:3643331	Gm8182	predicted gene 8182 [Source:MGI Symbol;Acc:MGI:3643331]
5	processed_pseudogene	61495239	61496076	ENSMUSG00000106370	MGI:5663519	Gm43382	predicted gene 43382 [Source:MGI Symbol;Acc:MGI:5663519]
5	processed_pseudogene	61759913	61760381	ENSMUSG00000105675	MGI:5663520	Gm43383	predicted gene 43383 [Source:MGI Symbol;Acc:MGI:5663520]
5	processed_pseudogene	61874373	61875009	ENSMUSG00000105573	MGI:5663521	Gm43384	predicted gene 43384 [Source:MGI Symbol;Acc:MGI:5663521]

5	processed_pseudogene	62161563	62162178	ENSMUSG00000106530	MGI:5662567	Gm42430	predicted gene 42430 [Source:MGI Symbol;Acc:MGI:5662567]
5	processed_pseudogene	63851397	63851856	ENSMUSG00000090138	MGI:3781863	Gm3687	predicted gene 3687 [Source:MGI Symbol;Acc:MGI:3781863]
5	processed_pseudogene	64147731	64148214	ENSMUSG00000106076	MGI:5663977	Gm43840	predicted gene 43840 [Source:MGI Symbol;Acc:MGI:5663977]
5	processed_pseudogene	64347376	64348330	ENSMUSG00000083435	MGI:3801976	Gm15824	predicted gene 15824 [Source:MGI Symbol;Acc:MGI:3801976]
5	processed_pseudogene	64409899	64410568	ENSMUSG00000081143	MGI:3801858	Gm15823	predicted gene 15823 [Source:MGI Symbol;Acc:MGI:3801858]
5	processed_pseudogene	64477599	64478097	ENSMUSG00000083100	MGI:3648725	Gm6044	predicted gene 6044 [Source:MGI Symbol;Acc:MGI:3648725]
5	processed_pseudogene	66355255	66358669	ENSMUSG00000084108	MGI:3783236	Gm15794	predicted gene 15794 [Source:MGI Symbol;Acc:MGI:3783236]
5	processed_pseudogene	66514261	66514528	ENSMUSG00000106910	MGI:3826595	Gm16273	predicted gene 16273 [Source:MGI Symbol;Acc:MGI:3826595]
5	processed_pseudogene	67047709	67048219	ENSMUSG00000082420	MGI:3643656	Gm6517	predicted gene 6517 [Source:MGI Symbol;Acc:MGI:3643656]
5	processed_pseudogene	68145870	68146350	ENSMUSG00000107257	MGI:5663165	Gm43028	predicted gene 43028 [Source:MGI Symbol;Acc:MGI:5663165]
5	processed_pseudogene	68434815	68435139	ENSMUSG00000107289	MGI:5663163	Gm43026	predicted gene 43026 [Source:MGI Symbol;Acc:MGI:5663163]
5	processed_pseudogene	69618495	69619086	ENSMUSG00000043484	MGI:3644922	Gm5867	predicted gene 5867 [Source:MGI Symbol;Acc:MGI:3644922]

5	processed_pseudogene	70088868	70089151	ENSMUSG00000106284	MGI:5663276	Gm43139	predicted gene 43139 [Source:MGI Symbol;Acc:MGI:5663276]
5	processed_pseudogene	70710951	70711564	ENSMUSG00000106613	MGI:5662789	Gm42652	predicted gene 42652 [Source:MGI Symbol;Acc:MGI:5662789]
5	processed_pseudogene	72190061	72190831	ENSMUSG00000080736	MGI:3783062	Gm15617	predicted gene 15617 [Source:MGI Symbol;Acc:MGI:3783062]
5	processed_pseudogene	72204548	72204922	ENSMUSG00000082705	MGI:3783061	Gm15616	predicted gene 15616 [Source:MGI Symbol;Acc:MGI:3783061]
5	processed_pseudogene	72329160	72329631	ENSMUSG00000105016	MGI:5662706	Gm42569	predicted gene 42569 [Source:MGI Symbol;Acc:MGI:5662706]
5	processed_pseudogene	72566787	72567300	ENSMUSG00000093644	MGI:5011745	Gm19560	predicted gene, 19560 [Source:MGI Symbol;Acc:MGI:5011745]
5	processed_pseudogene	72729517	72729775	ENSMUSG00000105865	MGI:5593303	Gm34144	predicted gene, 34144 [Source:MGI Symbol;Acc:MGI:5593303]
5	processed_pseudogene	72883405	72883714	ENSMUSG00000105089	MGI:5663854	Gm43717	predicted gene 43717 [Source:MGI Symbol;Acc:MGI:5663854]
5	processed_pseudogene	73554142	73555156	ENSMUSG00000106958	MGI:5595119	Gm35960	predicted gene, 35960 [Source:MGI Symbol;Acc:MGI:5595119]
5	processed_pseudogene	75043996	75044896	ENSMUSG00000106806	MGI:5010091	Gm17906	predicted gene, 17906 [Source:MGI Symbol;Acc:MGI:5010091]
5	processed_pseudogene	75209140	75210697	ENSMUSG00000107162	MGI:5010778	Gm18593	predicted gene, 18593 [Source:MGI Symbol;Acc:MGI:5010778]
5	processed_pseudogene	75269296	75269584	ENSMUSG00000106900	MGI:3780209	Gm2040	predicted gene 2040 [Source:MGI Symbol;Acc:MGI:3780209]

5	processed_pseudogene	75407104	75407431	ENSMUSG00000106704	MGI:5662936	Gm42799	predicted gene 42799 [Source:MGI Symbol;Acc:MGI:5662936]
5	processed_pseudogene	76221072	76221423	ENSMUSG00000107020	MGI:3645584	Gm6051	predicted gene 6051 [Source:MGI Symbol;Acc:MGI:3645584]
5	processed_pseudogene	76380459	76380917	ENSMUSG00000106831	MGI:894296	Ube2n-ps1	ubiquitin-conjugating enzyme E2N, pseudogene 1 [Source:MGI Symbol;Acc:MGI:894296]
5	processed_pseudogene	76558201	76558352	ENSMUSG00000107160	MGI:5662803	Gm42666	predicted gene 42666 [Source:MGI Symbol;Acc:MGI:5662803]
5	processed_pseudogene	76598548	76599621	ENSMUSG00000106982	MGI:5662802	Gm42665	predicted gene 42665 [Source:MGI Symbol;Acc:MGI:5662802]
5	processed_pseudogene	76889920	76890360	ENSMUSG00000083874	MGI:3643474	Gm7494	predicted gene 7494 [Source:MGI Symbol;Acc:MGI:3643474]
5	processed_pseudogene	77375225	77375797	ENSMUSG00000107034	MGI:5011245	Gm19060	predicted gene, 19060 [Source:MGI Symbol;Acc:MGI:5011245]
5	processed_pseudogene	77466502	77466784	ENSMUSG00000107238	MGI:5662895	Gm42758	predicted gene 42758 [Source:MGI Symbol;Acc:MGI:5662895]
5	processed_pseudogene	78056971	78057307	ENSMUSG00000106960	MGI:5662810	Gm42673	predicted gene 42673 [Source:MGI Symbol;Acc:MGI:5662810]
5	processed_pseudogene	78621732	78622582	ENSMUSG00000044058	MGI:2384077	Mthfr-ps1	5,10-methylenetetrahydrofolate reductase, pseudogene 1 [Source:MGI Symbol;Acc:MGI:2384077]
5	processed_pseudogene	78688375	78688738	ENSMUSG00000107309	MGI:5663369	Gm43232	predicted gene 43232 [Source:MGI Symbol;Acc:MGI:5663369]
5	processed_pseudogene	79588044	79588061	ENSMUSG00000107308	MGI:5663371	Gm43234	predicted gene 43234 [Source:MGI Symbol;Acc:MGI:5663371]

5	processed_pseudogene	79655248	79656358	ENSMUSG00000107183	MGI:5010637	Gm18452	predicted gene, 18452 [Source:MGI Symbol;Acc:MGI:5010637]
5	processed_pseudogene	81048597	81049470	ENSMUSG00000083829	MGI:3780369	Gm2199	predicted gene 2199 [Source:MGI Symbol;Acc:MGI:3780369]
5	processed_pseudogene	82484698	82485496	ENSMUSG00000106926	MGI:3644615	Rpl7-ps7	ribosomal protein L7, pseudogene 7 [Source:MGI Symbol;Acc:MGI:3644615]
5	processed_pseudogene	83188485	83188851	ENSMUSG00000106606	MGI:5663354	Gm43217	predicted gene 43217 [Source:MGI Symbol;Acc:MGI:5663354]
5	processed_pseudogene	83447432	83448299	ENSMUSG00000081822	MGI:3783070	Gm15626	predicted gene 15626 [Source:MGI Symbol;Acc:MGI:3783070]
5	processed_pseudogene	84766565	84767678	ENSMUSG00000105658	MGI:5663668	Gm43531	predicted gene 43531 [Source:MGI Symbol;Acc:MGI:5663668]
5	processed_pseudogene	85014038	85014704	ENSMUSG00000106389	MGI:5434361	Gm21006	predicted gene, 21006 [Source:MGI Symbol;Acc:MGI:5434361]
5	processed_pseudogene	86153266	86153476	ENSMUSG00000106225	MGI:5663701	Gm43564	predicted gene 43564 [Source:MGI Symbol;Acc:MGI:5663701]
5	processed_pseudogene	87110235	87110423	ENSMUSG00000118539	MGI:6388938	Gm53044	predicted gene, 53044 [Source:MGI Symbol;Acc:MGI:6388938]
5	processed_pseudogene	87145018	87146274	ENSMUSG00000104802	MGI:3648808	Gm5869	predicted gene 5869 [Source:MGI Symbol;Acc:MGI:3648808]
5	processed_pseudogene	87162019	87163078	ENSMUSG00000105315	MGI:5010820	Gm18635	predicted gene, 18635 [Source:MGI Symbol;Acc:MGI:5010820]
5	processed_pseudogene	87262393	87262468	ENSMUSG00000106502	MGI:5662932	Gm42795	predicted gene 42795 [Source:MGI Symbol;Acc:MGI:5662932]

5	processed_pseudogene	87339481	87341032	ENSMUSG00000106330	MGI:5663506	Gm43369	predicted gene 43369 [Source:MGI Symbol;Acc:MGI:5663506]
5	processed_pseudogene	87517662	87518342	ENSMUSG00000104664	MGI:5594729	Idi1-ps2	isopentenyl-diphosphate delta isomerase, pseudogene 2 [Source:MGI Symbol;Acc:MGI:5594729]
5	processed_pseudogene	87748930	87749338	ENSMUSG00000106217	MGI:5663505	Gm43368	predicted gene 43368 [Source:MGI Symbol;Acc:MGI:5663505]
5	processed_pseudogene	87751561	87753475	ENSMUSG00000105246	MGI:5434398	Gm21043	predicted gene, 21043 [Source:MGI Symbol;Acc:MGI:5434398]
5	processed_pseudogene	87884965	87885069	ENSMUSG00000105796	MGI:5662982	Gm42845	predicted gene 42845 [Source:MGI Symbol;Acc:MGI:5662982]
5	processed_pseudogene	88210375	88212232	ENSMUSG00000105905	MGI:3646978	Gm7709	predicted gene 7709 [Source:MGI Symbol;Acc:MGI:3646978]
5	processed_pseudogene	88218805	88219183	ENSMUSG00000104640	MGI:5662760	Gm42623	predicted gene 42623 [Source:MGI Symbol;Acc:MGI:5662760]
5	processed_pseudogene	88292563	88292917	ENSMUSG00000105807	MGI:5662759	Gm42622	predicted gene 42622 [Source:MGI Symbol;Acc:MGI:5662759]
5	processed_pseudogene	88379209	88381099	ENSMUSG00000106593	MGI:3779657	Gm7048	predicted gene 7048 [Source:MGI Symbol;Acc:MGI:3779657]
5	processed_pseudogene	88571074	88571413	ENSMUSG00000106633	MGI:5663568	Gm43431	predicted gene 43431 [Source:MGI Symbol;Acc:MGI:5663568]
5	processed_pseudogene	88575672	88576217	ENSMUSG00000105013	MGI:3644419	Gm7721	predicted gene 7721 [Source:MGI Symbol;Acc:MGI:3644419]
5	processed_pseudogene	88692895	88693015	ENSMUSG00000106322	MGI:5663569	Gm43432	predicted gene 43432 [Source:MGI Symbol;Acc:MGI:5663569]

5	processed_pseudogene	88887106	88887588	ENSMUSG00000095524	MGI:3783124	Gm15682	predicted gene 15682 [Source:MGI Symbol;Acc:MGI:3783124]
5	processed_pseudogene	88951453	88953082	ENSMUSG00000106305	MGI:5663048	Gm42911	predicted gene 42911 [Source:MGI Symbol;Acc:MGI:5663048]
5	processed_pseudogene	88985869	88987258	ENSMUSG00000082389	MGI:2663191	Mkrn1-ps1	makorin, ring finger protein 1, pseudogene 1 [Source:MGI Symbol;Acc:MGI:2663191]
5	processed_pseudogene	89647011	89647836	ENSMUSG00000106534	MGI:3646411	Gm6366	predicted gene 6366 [Source:MGI Symbol;Acc:MGI:3646411]
5	processed_pseudogene	90280088	90280534	ENSMUSG00000043618	MGI:3643585	Eif5al3-ps	eukaryotic translation initiation factor 5A-like 3, pseudogene [Source:MGI Symbol;Acc:MGI:3643585]
5	processed_pseudogene	90706708	90707278	ENSMUSG00000106705	MGI:3780770	Gm2602	predicted gene 2602 [Source:MGI Symbol;Acc:MGI:3780770]
5	processed_pseudogene	90898384	90898697	ENSMUSG00000107072	MGI:5663227	Gm43090	predicted gene 43090 [Source:MGI Symbol;Acc:MGI:5663227]
5	processed_pseudogene	91885564	91885938	ENSMUSG00000107261	MGI:3643840	Trmt112-ps1	tRNA methyltransferase 11-2, pseudogene 1 [Source:MGI Symbol;Acc:MGI:3643840]
5	processed_pseudogene	91936457	91937340	ENSMUSG00000107337	MGI:5663733	Gm43596	predicted gene 43596 [Source:MGI Symbol;Acc:MGI:5663733]
5	processed_pseudogene	91954438	91954932	ENSMUSG00000096726	MGI:3644616	Gm5558	predicted gene 5558 [Source:MGI Symbol;Acc:MGI:3644616]
5	processed_pseudogene	92288153	92288788	ENSMUSG00000084111	MGI:3783151	Gm15710	predicted gene 15710 [Source:MGI Symbol;Acc:MGI:3783151]
5	processed_pseudogene	92511763	92513370	ENSMUSG00000082068	MGI:3780842	Gm2673	predicted gene 2673 [Source:MGI Symbol;Acc:MGI:3780842]

5	processed_pseudogene	93151690	93152263	ENSMUSG00000106930	MGI:3648082	Gm6450	predicted gene 6450 [Source:MGI Symbol;Acc:MGI:3648082]
5	processed_pseudogene	93343657	93344038	ENSMUSG00000083700	MGI:3781164	Gm2986	predicted gene 2986 [Source:MGI Symbol;Acc:MGI:3781164]
5	processed_pseudogene	93538461	93540950	ENSMUSG00000053208	MGI:3648817	Kat2b-ps	K(lysine) acetyltransferase 2B, pseudogene [Source:MGI Symbol;Acc:MGI:3648817]
5	processed_pseudogene	93854333	93854591	ENSMUSG00000106954	MGI:5663363	Gm43226	predicted gene 43226 [Source:MGI Symbol;Acc:MGI:5663363]
5	processed_pseudogene	96009573	96010574	ENSMUSG00000096617	MGI:3779498	Gm5559	predicted gene 5559 [Source:MGI Symbol;Acc:MGI:3779498]
5	processed_pseudogene	96122099	96123482	ENSMUSG00000107092	MGI:3647399	Gm7993	predicted gene 7993 [Source:MGI Symbol;Acc:MGI:3647399]
5	processed_pseudogene	96321799	96322209	ENSMUSG00000090468	MGI:3781012	Gm2840	predicted gene 2840 [Source:MGI Symbol;Acc:MGI:3781012]
5	processed_pseudogene	97412687	97413578	ENSMUSG00000067161	MGI:3647980	Gm5560	predicted pseudogene 5560 [Source:MGI Symbol;Acc:MGI:3647980]
5	processed_pseudogene	98204652	98204962	ENSMUSG00000105117	MGI:5662897	Gm42760	predicted gene 42760 [Source:MGI Symbol;Acc:MGI:5662897]
5	processed_pseudogene	98223162	98223777	ENSMUSG00000105441	MGI:3647081	Gm8041	predicted gene 8041 [Source:MGI Symbol;Acc:MGI:3647081]
5	processed_pseudogene	98301040	98302035	ENSMUSG00000098073	MGI:3648505	Gm8048	predicted gene 8048 [Source:MGI Symbol;Acc:MGI:3648505]
5	processed_pseudogene	98601606	98601986	ENSMUSG00000106273	MGI:5663904	Gm43767	predicted gene 43767 [Source:MGI Symbol;Acc:MGI:5663904]

5	processed_pseudogene	99169662	99170081	ENSMUSG00000089659	MGI:3781646	Gm3470	predicted gene 3470 [Source:MGI Symbol;Acc:MGI:3781646]
5	processed_pseudogene	99758484	99759292	ENSMUSG00000089627	MGI:3801790	Gm16228	predicted gene 16228 [Source:MGI Symbol;Acc:MGI:3801790]
5	processed_pseudogene	99987392	99987849	ENSMUSG00000104731	MGI:5663386	Gm43249	predicted gene 43249 [Source:MGI Symbol;Acc:MGI:5663386]
5	processed_pseudogene	1.01E+08	1.01E+08	ENSMUSG00000105181	MGI:5011805	Gm19620	predicted gene, 19620 [Source:MGI Symbol;Acc:MGI:5011805]
5	processed_pseudogene	1.01E+08	1.01E+08	ENSMUSG00000105965	MGI:109499	Cycs-ps2	cytochrome c, pseudogene 2 [Source:MGI Symbol;Acc:MGI:109499]
5	processed_pseudogene	1.02E+08	1.02E+08	ENSMUSG00000106610	MGI:5663240	Gm43103	predicted gene 43103 [Source:MGI Symbol;Acc:MGI:5663240]
5	processed_pseudogene	1.02E+08	1.02E+08	ENSMUSG00000105088	MGI:5010163	Gm17978	predicted gene, 17978 [Source:MGI Symbol;Acc:MGI:5010163]
5	processed_pseudogene	1.02E+08	1.02E+08	ENSMUSG00000106189	MGI:5663070	Gm42933	predicted gene 42933 [Source:MGI Symbol;Acc:MGI:5663070]
5	processed_pseudogene	1.03E+08	1.03E+08	ENSMUSG00000106295	MGI:5663069	Gm42932	predicted gene 42932 [Source:MGI Symbol;Acc:MGI:5663069]
5	processed_pseudogene	1.03E+08	1.03E+08	ENSMUSG00000105253	MGI:5662659	Gm42522	predicted gene 42522 [Source:MGI Symbol;Acc:MGI:5662659]
5	processed_pseudogene	1.03E+08	1.03E+08	ENSMUSG00000104688	MGI:5010887	Gm18702	predicted gene, 18702 [Source:MGI Symbol;Acc:MGI:5010887]
5	processed_pseudogene	1.04E+08	1.04E+08	ENSMUSG00000084126	MGI:3802079	Gm15844	predicted gene 15844 [Source:MGI Symbol;Acc:MGI:3802079]

5	processed_pseudogene	1.04E+08	1.04E+08	ENSMUSG00000082527	MGI:3643346	Gm8200	predicted gene 8200 [Source:MGI Symbol;Acc:MGI:3643346]
5	processed_pseudogene	1.05E+08	1.05E+08	ENSMUSG00000048334	MGI:3644429	Gm8258	predicted gene 8258 [Source:MGI Symbol;Acc:MGI:3644429]
5	processed_pseudogene	1.05E+08	1.05E+08	ENSMUSG00000104899	MGI:5663992	Gm43855	predicted gene 43855 [Source:MGI Symbol;Acc:MGI:5663992]
5	processed_pseudogene	1.05E+08	1.05E+08	ENSMUSG00000106392	MGI:3644057	Gm5870	predicted pseudogene 5870 [Source:MGI Symbol;Acc:MGI:3644057]
5	processed_pseudogene	1.06E+08	1.06E+08	ENSMUSG00000067058	MGI:3642245	Rps15a-ps5	ribosomal protein S15A, pseudogene 5 [Source:MGI Symbol;Acc:MGI:3642245]
5	processed_pseudogene	1.06E+08	1.06E+08	ENSMUSG00000094708	MGI:3708724	Gm10359	predicted gene 10359 [Source:MGI Symbol;Acc:MGI:3708724]
5	processed_pseudogene	1.06E+08	1.06E+08	ENSMUSG00000105074	MGI:5663955	Gm43818	predicted gene 43818 [Source:MGI Symbol;Acc:MGI:5663955]
5	processed_pseudogene	1.06E+08	1.06E+08	ENSMUSG00000105129	MGI:5011751	Gm19566	predicted gene, 19566 [Source:MGI Symbol;Acc:MGI:5011751]
5	processed_pseudogene	1.06E+08	1.06E+08	ENSMUSG00000105600	MGI:3644525	Gm5987	predicted gene 5987 [Source:MGI Symbol;Acc:MGI:3644525]
5	processed_pseudogene	1.06E+08	1.06E+08	ENSMUSG00000106669	MGI:5621318	Gm38433	predicted gene, 38433 [Source:MGI Symbol;Acc:MGI:5621318]
5	processed_pseudogene	1.07E+08	1.07E+08	ENSMUSG00000104604	MGI:5663052	Gm42915	predicted gene 42915 [Source:MGI Symbol;Acc:MGI:5663052]
5	processed_pseudogene	1.07E+08	1.07E+08	ENSMUSG00000106142	MGI:5010122	Gm17937	predicted gene, 17937 [Source:MGI Symbol;Acc:MGI:5010122]

5	processed_pseudogene	1.07E+08	1.07E+08	ENSMUSG00000106301	MGI:3647827	Gm8365	predicted gene 8365 [Source:MGI Symbol;Acc:MGI:3647827]
5	processed_pseudogene	1.07E+08	1.07E+08	ENSMUSG00000084218	MGI:3644272	Gm8145	predicted gene 8145 [Source:MGI Symbol;Acc:MGI:3644272]
5	processed_pseudogene	1.08E+08	1.08E+08	ENSMUSG00000081798	MGI:3644504	Gm8152	predicted gene 8152 [Source:MGI Symbol;Acc:MGI:3644504]
5	processed_pseudogene	1.08E+08	1.08E+08	ENSMUSG00000089628	MGI:3779431	Gm9727	predicted gene 9727 [Source:MGI Symbol;Acc:MGI:3779431]
5	processed_pseudogene	1.08E+08	1.08E+08	ENSMUSG00000083609	MGI:3801785	Gm16007	predicted gene 16007 [Source:MGI Symbol;Acc:MGI:3801785]
5	processed_pseudogene	1.08E+08	1.08E+08	ENSMUSG00000096920	MGI:3780053	Rps19-ps8	ribosomal protein S19, pseudogene 8 [Source:MGI Symbol;Acc:MGI:3780053]
5	processed_pseudogene	1.08E+08	1.08E+08	ENSMUSG00000105554	MGI:3648119	Gm7118	predicted gene 7118 [Source:MGI Symbol;Acc:MGI:3648119]
5	processed_pseudogene	1.08E+08	1.08E+08	ENSMUSG00000105188	MGI:3646277	Gm8190	predicted gene 8190 [Source:MGI Symbol;Acc:MGI:3646277]
5	processed_pseudogene	1.08E+08	1.08E+08	ENSMUSG00000105591	MGI:5663220	Gm43083	predicted gene 43083 [Source:MGI Symbol;Acc:MGI:5663220]
5	processed_pseudogene	1.09E+08	1.09E+08	ENSMUSG00000060730	MGI:3642342	Gm10086	predicted pseudogene 10086 [Source:MGI Symbol;Acc:MGI:3642342]
5	processed_pseudogene	1.09E+08	1.09E+08	ENSMUSG00000093756	MGI:5010779	Gm18594	predicted gene, 18594 [Source:MGI Symbol;Acc:MGI:5010779]
5	processed_pseudogene	1.09E+08	1.09E+08	ENSMUSG00000105808	MGI:5662764	Gm42627	predicted gene 42627 [Source:MGI Symbol;Acc:MGI:5662764]

5	processed_pseudogene	1.09E+08	1.09E+08	ENSMUSG00000105193	MGI:5011156	Gm18971	predicted gene, 18971 [Source:MGI Symbol;Acc:MGI:5011156]
5	processed_pseudogene	1.09E+08	1.09E+08	ENSMUSG00000093725	MGI:5010780	Gm18595	predicted gene, 18595 [Source:MGI Symbol;Acc:MGI:5010780]
5	processed_pseudogene	1.09E+08	1.09E+08	ENSMUSG00000093545	MGI:3644058	Gm5871	predicted gene 5871 [Source:MGI Symbol;Acc:MGI:3644058]
5	processed_pseudogene	1.09E+08	1.09E+08	ENSMUSG00000093543	MGI:3647680	Gm8473	predicted gene 8473 [Source:MGI Symbol;Acc:MGI:3647680]
5	processed_pseudogene	1.10E+08	1.10E+08	ENSMUSG00000105303	MGI:5010740	Gm18555	predicted gene, 18555 [Source:MGI Symbol;Acc:MGI:5010740]
5	processed_pseudogene	1.10E+08	1.10E+08	ENSMUSG00000093688	MGI:3644909	Gm8488	predicted gene 8488 [Source:MGI Symbol;Acc:MGI:3644909]
5	processed_pseudogene	1.10E+08	1.10E+08	ENSMUSG00000094409	MGI:3761467	Vmn2r-ps23	vomeronasal 2, receptor, pseudogene 23 [Source:MGI Symbol;Acc:MGI:3761467]
5	processed_pseudogene	1.10E+08	1.10E+08	ENSMUSG00000049477	MGI:3643536	Gm8508	predicted gene 8508 [Source:MGI Symbol;Acc:MGI:3643536]
5	processed_pseudogene	1.10E+08	1.10E+08	ENSMUSG00000081865	MGI:3705726	Gm15484	predicted gene 15484 [Source:MGI Symbol;Acc:MGI:3705726]
5	processed_pseudogene	1.10E+08	1.10E+08	ENSMUSG00000081256	MGI:3783233	Gm15791	predicted gene 15791 [Source:MGI Symbol;Acc:MGI:3783233]
5	processed_pseudogene	1.11E+08	1.11E+08	ENSMUSG00000105603	MGI:5592155	Gm32996	predicted gene, 32996 [Source:MGI Symbol;Acc:MGI:5592155]
5	processed_pseudogene	1.11E+08	1.11E+08	ENSMUSG00000104602	MGI:5662916	Gm42779	predicted gene 42779 [Source:MGI Symbol;Acc:MGI:5662916]

5	processed_pseudogene	1.11E+08	1.11E+08	ENSMUSG00000081104	MGI:3801755	Gm15989	predicted gene 15989 [Source:MGI Symbol;Acc:MGI:3801755]
5	processed_pseudogene	1.11E+08	1.11E+08	ENSMUSG00000083771	MGI:3801756	Gm15988	predicted gene 15988 [Source:MGI Symbol;Acc:MGI:3801756]
5	processed_pseudogene	1.13E+08	1.13E+08	ENSMUSG00000105406	MGI:5663384	Gm43247	predicted gene 43247 [Source:MGI Symbol;Acc:MGI:5663384]
5	processed_pseudogene	1.14E+08	1.14E+08	ENSMUSG00000105031	MGI:3781688	Gm3511	predicted gene 3511 [Source:MGI Symbol;Acc:MGI:3781688]
5	processed_pseudogene	1.14E+08	1.14E+08	ENSMUSG00000104972	MGI:5012116	Gm19931	predicted gene, 19931 [Source:MGI Symbol;Acc:MGI:5012116]
5	processed_pseudogene	1.14E+08	1.14E+08	ENSMUSG00000106484	MGI:5010018	Gm17833	predicted gene, 17833 [Source:MGI Symbol;Acc:MGI:5010018]
5	processed_pseudogene	1.14E+08	1.14E+08	ENSMUSG00000084243	MGI:3649548	Gm13784	predicted gene 13784 [Source:MGI Symbol;Acc:MGI:3649548]
5	processed_pseudogene	1.15E+08	1.15E+08	ENSMUSG00000082052	MGI:3650308	Ywhaq-ps2	tyrosine 3-monooxygenase/tryptophan 5-monooxygenase activation protein theta, pseudogene 2 [Source:MGI Symbol;Acc:MGI:3650308]
5	processed_pseudogene	1.15E+08	1.15E+08	ENSMUSG00000080707	MGI:3651997	Gm13823	predicted gene 13823 [Source:MGI Symbol;Acc:MGI:3651997]
5	processed_pseudogene	1.15E+08	1.15E+08	ENSMUSG00000086167	MGI:3651518	Gm13827	predicted gene 13827 [Source:MGI Symbol;Acc:MGI:3651518]
5	processed_pseudogene	1.15E+08	1.15E+08	ENSMUSG00000083182	MGI:3651756	Gm13828	predicted gene 13828 [Source:MGI Symbol;Acc:MGI:3651756]

5	processed_pseudogene	1.15E+08	1.15E+08	ENSMUSG00000083875	MGI:3651085	Gm13831	predicted gene 13831 [Source:MGI Symbol;Acc:MGI:3651085]
5	processed_pseudogene	1.15E+08	1.15E+08	ENSMUSG00000082610	MGI:3651367	Gm13829	predicted gene 13829 [Source:MGI Symbol;Acc:MGI:3651367]
5	processed_pseudogene	1.16E+08	1.16E+08	ENSMUSG00000083833	MGI:3650890	Gm13841	predicted gene 13841 [Source:MGI Symbol;Acc:MGI:3650890]
5	processed_pseudogene	1.17E+08	1.17E+08	ENSMUSG00000107369	MGI:3648223	Gstm2-ps1	glutathione S-transferase mu 2 (muscle), pseudogene 1 [Source:MGI Symbol;Acc:MGI:3648223]
5	processed_pseudogene	1.17E+08	1.17E+08	ENSMUSG00000107327	MGI:3648572	Gm7478	predicted gene 7478 [Source:MGI Symbol;Acc:MGI:3648572]
5	processed_pseudogene	1.17E+08	1.17E+08	ENSMUSG00000106829	MGI:3781959	Gm3786	predicted gene 3786 [Source:MGI Symbol;Acc:MGI:3781959]
5	processed_pseudogene	1.17E+08	1.17E+08	ENSMUSG00000081274	MGI:3783170	Gm15727	predicted gene 15727 [Source:MGI Symbol;Acc:MGI:3783170]
5	processed_pseudogene	1.21E+08	1.21E+08	ENSMUSG00000107004	MGI:5662794	Gm42657	predicted gene 42657 [Source:MGI Symbol;Acc:MGI:5662794]
5	processed_pseudogene	1.22E+08	1.22E+08	ENSMUSG00000082760	MGI:3782995	Gm15546	predicted gene 15546 [Source:MGI Symbol;Acc:MGI:3782995]
5	processed_pseudogene	1.22E+08	1.22E+08	ENSMUSG00000082265	MGI:3782996	Gm15547	predicted gene 15547 [Source:MGI Symbol;Acc:MGI:3782996]
5	processed_pseudogene	1.22E+08	1.22E+08	ENSMUSG00000082645	MGI:3705452	Gm15481	predicted gene 15481 [Source:MGI Symbol;Acc:MGI:3705452]
5	processed_pseudogene	1.22E+08	1.22E+08	ENSMUSG00000083909	MGI:3801843	Gm15842	predicted gene 15842 [Source:MGI Symbol;Acc:MGI:3801843]

5	processed_pseudogene	1.23E+08	1.23E+08	ENSMUSG00000079906	MGI:3801824	Gm15846	predicted gene 15846 [Source:MGI Symbol;Acc:MGI:3801824]
5	processed_pseudogene	1.23E+08	1.23E+08	ENSMUSG00000059352	MGI:3645905	Gm10064	predicted gene 10064 [Source:MGI Symbol;Acc:MGI:3645905]
5	processed_pseudogene	1.23E+08	1.23E+08	ENSMUSG00000080836	MGI:3648226	Gm6444	predicted gene 6444 [Source:MGI Symbol;Acc:MGI:3648226]
5	processed_pseudogene	1.23E+08	1.23E+08	ENSMUSG00000094664	MGI:3704258	Rpl35a-ps6	ribosomal protein L35A, pseudogene 6 [Source:MGI Symbol;Acc:MGI:3704258]
5	processed_pseudogene	1.24E+08	1.24E+08	ENSMUSG00000082532	MGI:3783190	Rpl31-ps6	ribosomal protein L31, pseudogene 6 [Source:MGI Symbol;Acc:MGI:3783190]
5	processed_pseudogene	1.25E+08	1.25E+08	ENSMUSG00000082402	MGI:3783066	Gm15621	predicted gene 15621 [Source:MGI Symbol;Acc:MGI:3783066]
5	protein_coding	41782319	41865500	ENSMUSG00000029128	MGI:1917285	Rab28	RAB28, member RAS oncogene family [Source:MGI Symbol;Acc:MGI:1917285]
5	protein_coding	41918826	41921844	ENSMUSG00000049691	MGI:108015	Nkx3-2	NK3 homeobox 2 [Source:MGI Symbol;Acc:MGI:108015]
5	protein_coding	41944881	42001658	ENSMUSG00000061755	MGI:2444804	Bod1l	biorientation of chromosomes in cell division 1-like [Source:MGI Symbol;Acc:MGI:2444804]
5	protein_coding	43390513	43447067	ENSMUSG00000039782	MGI:2442640	Cpeb2	[illegible] protein 2 [Source:MGI Symbol;Acc:MGI:2442640]
5	protein_coding	43672881	43776145	ENSMUSG00000061535	MGI:1925911	C1qtnf7	C1q and tumor necrosis factor related protein 7 [Source:MGI Symbol;Acc:MGI:1925911]
5	protein_coding	43819688	43898314	ENSMUSG00000039765	MGI:1924487	Cc2d2a	coiled-coil and C2 domain containing 2A [Source:MGI Symbol;Acc:MGI:1924487]
5	protein_coding	43901957	43978980	ENSMUSG00000039753	MGI:2152883	Fbxl5	F-box and leucine-rich repeat protein 5 [Source:MGI Symbol;Acc:MGI:2152883]
5	protein_coding	43976227	44001328	ENSMUSG00000029082	MGI:105370	Bst1	bone marrow stromal cell antigen 1 [Source:MGI Symbol;Acc:MGI:105370]
5	protein_coding	44025895	44069717	ENSMUSG00000029084	MGI:107474	Cd38	CD38 antigen [Source:MGI Symbol;Acc:MGI:107474]

5	protein_coding	44070486	44071259	ENSMUSG00000033036	MGI:3645078	Ppihl	peptidyl prolyl isomerase H like [Source:MGI Symbol;Acc:MGI:3645078]
5	protein_coding	44136200	44139121	ENSMUSG00000048373	MGI:1096350	Fgfbp1	fibroblast growth factor binding protein 1 [Source:MGI Symbol;Acc:MGI:1096350]
5	protein_coding	44150962	44259374	ENSMUSG00000029086	MGI:1100886	Prom1	prominin 1 [Source:MGI Symbol;Acc:MGI:1100886]
5	protein_coding	44332496	44383968	ENSMUSG00000046985	MGI:2683537	Tapt1	transformation 1 [Source:MGI Symbol;Acc:MGI:2683537]
5	protein_coding	44629474	44957022	ENSMUSG00000039706	MGI:894670	Ldb2	LIM domain binding 2 [Source:MGI Symbol;Acc:MGI:894670]
5	protein_coding	45591363	45607578	ENSMUSG00000015806	MGI:97836	Qdpr	quinoid dihydropteridine reductase [Source:MGI Symbol;Acc:MGI:97836]
5	protein_coding	45611093	45621491	ENSMUSG00000049530	MGI:3646230	Clrn2	clarin 2 [Source:MGI Symbol;Acc:MGI:3646230]
5	protein_coding	45650716	45670033	ENSMUSG00000039682	MGI:1914238	Lap3	leucine aminopeptidase 3 [Source:MGI Symbol;Acc:MGI:1914238]
5	protein_coding	45677571	45686618	ENSMUSG00000015804	MGI:1914249	Med28	mediator complex subunit 28 [Source:MGI Symbol;Acc:MGI:1914249]
5	protein_coding	45687047	45796956	ENSMUSG00000015879	MGI:2442958	Fam184b	family with sequence similarity 184, member B [Source:MGI Symbol;Acc:MGI:2442958]
5	protein_coding	45827261	45857888	ENSMUSG00000015880	MGI:1930197	Ncapg	non-SMC condensin I complex, subunit G [Source:MGI Symbol;Acc:MGI:1930197]
5	protein_coding	45854523	46014957	ENSMUSG00000015882	MGI:2651932	Lcorl	ligand dependent nuclear receptor corepressor-like [Source:MGI Symbol;Acc:MGI:2651932]
5	protein_coding	48140480	48465075	ENSMUSG00000031558	MGI:1315205	Slit2	slit guidance ligand 2 [Source:MGI Symbol;Acc:MGI:1315205]
5	protein_coding	48485294	48571636	ENSMUSG00000029089	MGI:1917842	5730480H06Rik	RIKEN cDNA 5730480H06 gene [Source:MGI Symbol;Acc:MGI:1917842]
5	protein_coding	48546844	49682249	ENSMUSG00000029088	MGI:1933131	Kcnip4	Kv channel interacting protein 4 [Source:MGI Symbol;Acc:MGI:1933131]
5	protein_coding	50117298	50216348	ENSMUSG00000029090	MGI:1917943	Adgra3	adhesion G protein-coupled receptor A3 [Source:MGI Symbol;Acc:MGI:1917943]
5	protein_coding	51611592	51725068	ENSMUSG00000029167	MGI:1342774	Ppargc1a	peroxisome proliferative activated receptor, gamma, coactivator 1 alpha [Source:MGI Symbol;Acc:MGI:1342774]

5	protein_coding	52307545	52347856	ENSMUSG00000029169	MGI:1099786	Dhx15	DEAH-box helicase 15 [Source:MGI Symbol;Acc:MGI:1099786]
5	protein_coding	52521133	52528760	ENSMUSG00000072941	MGI:103181	Sod3	superoxide dismutase 3, extracellular [Source:MGI Symbol;Acc:MGI:103181]
5	protein_coding	52531993	52628863	ENSMUSG00000045790	MGI:2685293	Ccdc149	coiled-coil domain containing 149 [Source:MGI Symbol;Acc:MGI:2685293]
5	protein_coding	52690859	52723804	ENSMUSG00000039252	MGI:2180196	Lgi2	leucine-rich repeat LGI family, member 2 [Source:MGI Symbol;Acc:MGI:2180196]
5	protein_coding	52797429	52827050	ENSMUSG00000029173	MGI:1098791	Sepsecs	Sep (O-phosphoserine) tRNA:Sec (selenocysteine) tRNA synthase [Source:MGI Symbol;Acc:MGI:1098791]
5	protein_coding	52898916	52926682	ENSMUSG00000029186	MGI:1914323	Pi4k2b	phosphatidylinositol 4-kinase type 2 beta [Source:MGI Symbol;Acc:MGI:1914323]
5	protein_coding	52932751	52982007	ENSMUSG00000029179	MGI:1926046	Zcchc4	zinc finger, CCHC domain containing 4 [Source:MGI Symbol;Acc:MGI:1926046]
5	protein_coding	52991354	53025139	ENSMUSG00000029176	MGI:1098673	Anapc4	anaphase promoting complex subunit 4 [Source:MGI Symbol;Acc:MGI:1098673]
5	protein_coding	53195423	53229006	ENSMUSG00000029188	MGI:1342284	Slc34a2	[illegible] member 2 [Source:MGI Symbol;Acc:MGI:1342284]
5	protein_coding	53264425	53371269	ENSMUSG00000029189	MGI:1916941	Sel1l3	sel-1 suppressor of lin-12-like 3 (C. elegans) [Source:MGI Symbol;Acc:MGI:1916941]
5	protein_coding	53424425	53435882	ENSMUSG00000061461	MGI:1913528	Smim20	small integral membrane protein 20 [Source:MGI Symbol;Acc:MGI:1913528]
5	protein_coding	53623494	53814704	ENSMUSG00000039191	MGI:96522	Rbpj	recombination signal binding protein for immunoglobulin kappa J region [Source:MGI Symbol;Acc:MGI:96522]
5	protein_coding	53855118	53865047	ENSMUSG00000029193	MGI:99478	Cckar	cholecystokinin A receptor [Source:MGI Symbol;Acc:MGI:99478]
5	protein_coding	53966948	54061307	ENSMUSG00000039178	MGI:1914499	Tbc1d19	TBC1 domain family, member 19 [Source:MGI Symbol;Acc:MGI:1914499]
5	protein_coding	54155841	54278399	ENSMUSG00000039156	MGI:2151156	Stim2	stromal interaction molecule 2 [Source:MGI Symbol;Acc:MGI:2151156]
5	protein_coding	57875309	58290572	ENSMUSG00000029108	MGI:1860487	Pcdh7	protocadherin 7 [Source:MGI Symbol;Acc:MGI:1860487]
5	protein_coding	61966159	61968506	ENSMUSG00000089992	MGI:105977	G6pd2	glucose-6-phosphate dehydrogenase 2 [Source:MGI Symbol;Acc:MGI:105977]

5	protein_coding	62759788	62923502	ENSMUSG00000037999	MGI:2684416	Arap2	ArfGAP with RhoGAP domain, ankyrin repeat and PH domain 2 [Source:MGI Symbol;Acc:MGI:2684416]
5	protein_coding	62969017	63045651	ENSMUSG00000090326	MGI:4937018	Dthd1	death domain containing 1 [Source:MGI Symbol;Acc:MGI:4937018]
5	protein_coding	63806445	63967889	ENSMUSG00000090061	MGI:1920464	Nwd2	NACHT and WD repeat domain containing 2 [Source:MGI Symbol;Acc:MGI:1920464]
5	protein_coding	63969706	64056968	ENSMUSG00000060512	MGI:1923511	Pgcka1	PDCD10 and GCKIII kinases associated 1 [Source:MGI Symbol;Acc:MGI:1923511]
5	protein_coding	64066240	64126240	ENSMUSG00000047881	MGI:2140767	Rell1	RELT-like 1 [Source:MGI Symbol;Acc:MGI:2140767]
5	protein_coding	64250293	64285694	ENSMUSG00000029171	MGI:97564	Pgm2	phosphoglucomutase 2 [Source:MGI Symbol;Acc:MGI:97564]
5	protein_coding	64313648	64508829	ENSMUSG00000029174	MGI:1889508	Tbc1d1	TBC1 domain family, member 1 [Source:MGI Symbol;Acc:MGI:1889508]
5	protein_coding	64960731	64990244	ENSMUSG00000029178	MGI:1342773	Klf3	Kruppel-like transcription factor 3 (basic) [Source:MGI Symbol;Acc:MGI:1342773]
5	protein_coding	65082022	65090906	ENSMUSG00000044827	MGI:1341295	Tlr1	toll-like receptor 1 [Source:MGI Symbol;Acc:MGI:1341295]
5	protein_coding	65109374	65117440	ENSMUSG00000051498	MGI:1341296	Tlr6	toll-like receptor 6 [Source:MGI Symbol;Acc:MGI:1341296]
5	protein_coding	65127414	65199229	ENSMUSG00000029185	MGI:1915553	Fam114a1	family with sequence similarity 114, member A1 [Source:MGI Symbol;Acc:MGI:1915553]
5	protein_coding	65211689	65249475	ENSMUSG00000037913	MGI:2685292	Tmem156	transmembrane protein 156 [Source:MGI Symbol;Acc:MGI:2685292]
5	protein_coding	65264882	65325531	ENSMUSG00000054920	MGI:1919028	Klhl5	kelch-like 5 [Source:MGI Symbol;Acc:MGI:1919028]
5	protein_coding	65357039	65417758	ENSMUSG00000037890	MGI:2443231	Wdr19	WD repeat domain 19 [Source:MGI Symbol;Acc:MGI:2443231]
5	protein_coding	65419193	65493013	ENSMUSG00000029191	MGI:97891	Rfc1	replication factor C (activator 1) 1 [Source:MGI Symbol;Acc:MGI:97891]
5	protein_coding	65505657	65541350	ENSMUSG00000029195	MGI:1932466	Klb	klotho beta [Source:MGI Symbol;Acc:MGI:1932466]
5	protein_coding	65545707	65548787	ENSMUSG00000047215	MGI:1298373	Rpl9	ribosomal protein L9 [Source:MGI Symbol;Acc:MGI:1298373]

5	protein_coding	65548840	65568036	ENSMUSG00000029199	MGI:1934604	Lias	lipoic acid synthetase [Source:MGI Symbol;Acc:MGI:1934604]
5	protein_coding	65570564	65593292	ENSMUSG00000029201	MGI:1306785	Ugdh	UDP-glucose dehydrogenase [Source:MGI Symbol;Acc:MGI:1306785]
5	protein_coding	65584770	65650176	ENSMUSG00000105835	MGI:5663689	Gm43552	predicted gene 43552 [Source:MGI Symbol;Acc:MGI:5663689]
5	protein_coding	65604187	65694527	ENSMUSG00000037822	MGI:1915802	Smim14	small integral membrane protein 14 [Source:MGI Symbol;Acc:MGI:1915802]
5	protein_coding	65694576	65756331	ENSMUSG00000029203	MGI:1858216	Ube2k	ubiquitin-conjugating enzyme E2K [Source:MGI Symbol;Acc:MGI:1858216]
5	protein_coding	65763064	65855616	ENSMUSG00000029202	MGI:1918771	Pds5a	PDS5 cohesin associated factor A [Source:MGI Symbol;Acc:MGI:1918771]
5	protein_coding	65920864	65987451	ENSMUSG00000037795	MGI:2684414	N4bp2	NEDD4 binding protein 2 [Source:MGI Symbol;Acc:MGI:2684414]
5	protein_coding	66018556	66054043	ENSMUSG00000029204	MGI:1921984	Rhoh	ras homolog family member H [Source:MGI Symbol;Acc:MGI:1921984]
5	protein_coding	66092264	66134669	ENSMUSG00000029205	MGI:1202403	Chrna9	polypeptide 9 [Source:MGI Symbol;Acc:MGI:1202403]
5	protein_coding	66173892	66330461	ENSMUSG00000070780	MGI:2384294	Rbm47	RNA binding motif protein 47 [Source:MGI Symbol;Acc:MGI:2384294]
5	protein_coding	66417240	66455369	ENSMUSG00000029206	MGI:1918168	Nsun7	NOL1/NOP2/Sun domain family, member 7 [Source:MGI Symbol;Acc:MGI:1918168]
5	protein_coding	66456046	66776127	ENSMUSG00000029207	MGI:108405	Apbb2	member 2 [Source:MGI Symbol;Acc:MGI:108405]
5	protein_coding	66700842	66700919	ENSMUSG00000121580	MGI:7820357	Gm68765	predicted gene, 68765 [Source:MGI Symbol;Acc:MGI:7820357]
5	protein_coding	66833434	66844577	ENSMUSG00000029223	MGI:103149	Uchl1	ubiquitin carboxy-terminal hydrolase L1 [Source:MGI Symbol;Acc:MGI:103149]
5	protein_coding	66903170	67214501	ENSMUSG00000037736	MGI:1924819	Limch1	LIM and calponin homology domains 1 [Source:MGI Symbol;Acc:MGI:1924819]
5	protein_coding	67251742	67256644	ENSMUSG00000012520	MGI:1100882	Phox2b	paired-like homeobox 2b [Source:MGI Symbol;Acc:MGI:1100882]
5	protein_coding	67417908	67448804	ENSMUSG00000037720	MGI:1915128	Tmem33	transmembrane protein 33 [Source:MGI Symbol;Acc:MGI:1915128]
5	protein_coding	67464298	67515786	ENSMUSG00000029221	MGI:1923690	Slc30a9	member 9 [Source:MGI Symbol;Acc:MGI:1923690]

5	protein_coding	67549490	67585653	ENSMUSG00000092060	MGI:3648414	Bend4	BEN domain containing 4 [Source:MGI Symbol;Acc:MGI:3648414]
5	protein_coding	67765225	67780895	ENSMUSG00000050010	MGI:3041225	Shisa3	shisa family member 3 [Source:MGI Symbol;Acc:MGI:3041225]
5	protein_coding	67775483	68004777	ENSMUSG00000037685	MGI:1330848	Atp8a1	ATPase phospholipid transporting 8A1 [Source:MGI Symbol;Acc:MGI:1330848]
5	protein_coding	68189178	68323741	ENSMUSG00000068082	MGI:3577767	Grxcr1	glutaredoxin, cysteine rich 1 [Source:MGI Symbol;Acc:MGI:3577767]
5	protein_coding	69266628	69499022	ENSMUSG00000037653	MGI:2443804	Kctd8	potassium channel tetramerisation domain containing 8 [Source:MGI Symbol;Acc:MGI:2443804]
5	protein_coding	69674014	69699991	ENSMUSG00000029158	MGI:1922831	Yipf7	Yip1 domain family, member 7 [Source:MGI Symbol;Acc:MGI:1922831]
5	protein_coding	69714266	69733316	ENSMUSG00000029208	MGI:2140726	Guf1	GUF1 homolog, GTPase [Source:MGI Symbol;Acc:MGI:2140726]
5	protein_coding	69730451	69749683	ENSMUSG00000029209	MGI:1915230	Gnpda2	glucosamine-6-phosphate deaminase 2 [Source:MGI Symbol;Acc:MGI:1915230]
5	protein_coding	70908390	70999960	ENSMUSG00000001260	MGI:103156	Gabrg1	subunit gamma 1 [Source:MGI Symbol;Acc:MGI:103156]
5	protein_coding	71114940	71253192	ENSMUSG00000000560	MGI:95614	Gabra2	subunit alpha 2 [Source:MGI Symbol;Acc:MGI:95614]
5	protein_coding	71600167	71705548	ENSMUSG00000049387	MGI:1925424	Cox7b2	cytochrome c oxidase subunit 7B2 [Source:MGI Symbol;Acc:MGI:1925424]
5	protein_coding	71727092	71815651	ENSMUSG00000029211	MGI:95616	Gabra4	subunit alpha 4 [Source:MGI Symbol;Acc:MGI:95616]
5	protein_coding	71815456	72306380	ENSMUSG00000029212	MGI:95619	Gabrb1	subunit beta 1 [Source:MGI Symbol;Acc:MGI:95619]
5	protein_coding	72313918	72325532	ENSMUSG00000029213	MGI:1343485	Commd8	COMM domain containing 8 [Source:MGI Symbol;Acc:MGI:1343485]
5	protein_coding	72360672	72456118	ENSMUSG00000046808	MGI:2450125	Atp10d	ATPase, class V, type 10D [Source:MGI Symbol;Acc:MGI:2450125]
5	protein_coding	72457368	72661816	ENSMUSG00000005220	MGI:1349451	Corin	corin, serine peptidase [Source:MGI Symbol;Acc:MGI:1349451]
5	protein_coding	72670644	72717027	ENSMUSG00000072889	MGI:1923646	Nfxl1	nuclear transcription factor, X-box binding-like 1 [Source:MGI Symbol;Acc:MGI:1923646]

5	protein_coding	72734149	72738597	ENSMUSG00000063935	MGI:2180337	Zar1	zygote arrest 1 [Source:MGI Symbol;Acc:MGI:2180337]
5	protein_coding	72738981	72744894	ENSMUSG00000060204	MGI:3645333	Slc10a4-ps	solute carrier family 10 (sodium/bile acid cotransporter family), pseudogene [Source:MGI Symbol;Acc:MGI:3645333]
5	protein_coding	72761039	72801618	ENSMUSG00000067220	MGI:88436	Cnga1	cyclic nucleotide gated channel alpha 1 [Source:MGI Symbol;Acc:MGI:88436]
5	protein_coding	72805138	72828421	ENSMUSG00000067219	MGI:1917951	Nipal1	NIPA-like domain containing 1 [Source:MGI Symbol;Acc:MGI:1917951]
5	protein_coding	72853321	72910120	ENSMUSG00000054892	MGI:102960	Txk	TXK tyrosine kinase [Source:MGI Symbol;Acc:MGI:102960]
5	protein_coding	72913059	73025826	ENSMUSG00000029217	MGI:98662	Tec	tec protein tyrosine kinase [Source:MGI Symbol;Acc:MGI:98662]
5	protein_coding	73071647	73136172	ENSMUSG00000036087	MGI:1923241	Slain2	SLAIN motif family, member 2 [Source:MGI Symbol;Acc:MGI:1923241]
5	protein_coding	73123151	73132045	ENSMUSG00000063656	MGI:3710643	Gm10135	predicted gene 10135 [Source:MGI Symbol;Acc:MGI:3710643]
5	protein_coding	73164226	73170298	ENSMUSG00000029219	MGI:3606480	Slc10a4	solute carrier family 10 (sodium/bile acid cotransporter family), member 4 [Source:MGI Symbol;Acc:MGI:3606480]
5	protein_coding	73177330	73413962	ENSMUSG00000070733	MGI:1919563	Fryl	FRY like transcription coactivator [Source:MGI Symbol;Acc:MGI:1919563]
5	protein_coding	73450127	73471412	ENSMUSG00000029152	MGI:1915345	Ociad1	OCIA domain containing 1 [Source:MGI Symbol;Acc:MGI:1915345]
5	protein_coding	73479542	73498371	ENSMUSG00000029153	MGI:1916377	Ociad2	OCIA domain containing 2 [Source:MGI Symbol;Acc:MGI:1916377]
5	protein_coding	73563418	73610778	ENSMUSG00000029154	MGI:2444131	Cwh43	cell wall biogenesis 43 C-terminal homolog [Source:MGI Symbol;Acc:MGI:2444131]
5	protein_coding	73638343	73718137	ENSMUSG00000051674	MGI:2140972	Dcun1d4	containing 4 [Source:MGI Symbol;Acc:MGI:2140972]
5	protein_coding	73763985	73789869	ENSMUSG00000067206	MGI:2387634	Lrrc66	leucine rich repeat containing 66 [Source:MGI Symbol;Acc:MGI:2387634]
5	protein_coding	73790092	73805133	ENSMUSG00000029156	MGI:1346523	Sgcb	sarcoglycan, beta (dystrophin-associated glycoprotein) [Source:MGI Symbol;Acc:MGI:1346523]

5	protein_coding	73808722	73836855	ENSMUSG00000029155	MGI:1920722	Spata18	spermatogenesis associated 18 [Source:MGI Symbol;Acc:MGI:1920722]
5	protein_coding	74159114	74229092	ENSMUSG00000054814	MGI:1916977	Usp46	ubiquitin specific peptidase 46 [Source:MGI Symbol;Acc:MGI:1916977]
5	protein_coding	74355947	74360142	ENSMUSG00000049907	MGI:1916189	Rasl11b	RAS-like, family 11, member B [Source:MGI Symbol;Acc:MGI:1916189]
5	protein_coding	74365477	74692420	ENSMUSG00000062110	MGI:2443446	Scfd2	Sec1 family domain containing 2 [Source:MGI Symbol;Acc:MGI:2443446]
5	protein_coding	74696110	74759461	ENSMUSG00000029227	MGI:1914149	Fip1l1	factor interacting with PAPOLA and CPSF1 [Source:MGI Symbol;Acc:MGI:1914149]
5	protein_coding	74753108	74863573	ENSMUSG00000029228	MGI:1278335	Lnx1	ligand of numb-protein X 1 [Source:MGI Symbol;Acc:MGI:1278335]
5	protein_coding	75158649	75205435	ENSMUSG00000029229	MGI:1921527	Chic2	cysteine-rich hydrophobic domain 2 [Source:MGI Symbol;Acc:MGI:1921527]
5	protein_coding	75236262	75238554	ENSMUSG00000035946	MGI:95843	Gsx2	GS homeobox 2 [Source:MGI Symbol;Acc:MGI:95843]
5	protein_coding	75312953	75358876	ENSMUSG00000029231	MGI:97530	Pdgfra	[illegible] polypeptide [Source:MGI Symbol;Acc:MGI:97530]
5	protein_coding	75735576	75817382	ENSMUSG00000005672	MGI:96677	Kit	KIT proto-oncogene receptor tyrosine kinase [Source:MGI Symbol;Acc:MGI:96677]
5	protein_coding	76093487	76139118	ENSMUSG00000062960	MGI:96683	Kdr	kinase insert domain protein receptor [Source:MGI Symbol;Acc:MGI:96683]
5	protein_coding	76288118	76303351	ENSMUSG00000029233	MGI:1930252	Srd5a3	steroid 5 alpha-reductase 3 [Source:MGI Symbol;Acc:MGI:1930252]
5	protein_coding	76331727	76357092	ENSMUSG00000029234	MGI:894407	Tmem165	transmembrane protein 165 [Source:MGI Symbol;Acc:MGI:894407]
5	protein_coding	76357715	76452639	ENSMUSG00000029238	MGI:99698	Clock	clock circadian regulator [Source:MGI Symbol;Acc:MGI:99698]
5	protein_coding	76459962	76479003	ENSMUSG00000029235	MGI:1890655	Pdcl2	phosducin-like 2 [Source:MGI Symbol;Acc:MGI:1890655]
5	protein_coding	76481342	76511635	ENSMUSG00000029236	MGI:1860476	Nmu	neuromedin U [Source:MGI Symbol;Acc:MGI:1860476]
5	protein_coding	76631887	76664496	ENSMUSG00000091204	MGI:3647743	Exoc1l	exocyst complex component 1 like [Source:MGI Symbol;Acc:MGI:3647743]
5	protein_coding	76677158	76718141	ENSMUSG00000036435	MGI:2445020	Exoc1	exocyst complex component 1 [Source:MGI Symbol;Acc:MGI:2445020]

5	protein_coding	76736545	76794313	ENSMUSG00000036403	MGI:2681869	Cep135	centrosomal protein 135 [Source:MGI Symbol;Acc:MGI:2681869]
5	protein_coding	76804359	77021392	ENSMUSG00000036377	MGI:2444817	Cracd	capping protein inhibiting regulator of actin [Source:MGI Symbol;Acc:MGI:2444817]
5	protein_coding	77021506	77053361	ENSMUSG00000055923	MGI:2442517	Aasdh	aminoadipate-semialdehyde dehydrogenase [Source:MGI Symbol;Acc:MGI:2442517]
5	protein_coding	77061096	77099425	ENSMUSG00000029246	MGI:2387203	Ppat	[illegible] amidotransferase [Source:MGI Symbol;Acc:MGI:2387203]
5	protein_coding	77099154	77115356	ENSMUSG00000029247	MGI:1914304	Paics	phosphoribosylaminoimidazole carboxylase, phosphoribosylaminoribosylaminoimidazole, succinocarboxamide synthetase [Source:MGI Symbol;Acc:MGI:1914304]
5	protein_coding	77122530	77147784	ENSMUSG00000036323	MGI:1333795	Srp72	signal recognition particle 72 [Source:MGI Symbol;Acc:MGI:1333795]
5	protein_coding	77151902	77158453	ENSMUSG00000063820	MGI:1915496	Arl9	ADP-ribosylation factor-like 9 [Source:MGI Symbol;Acc:MGI:1915496]
5	protein_coding	77163870	77209376	ENSMUSG00000029248	MGI:1919118	Spmap2l	sperm microtubule associated protein 2 like [Source:MGI Symbol;Acc:MGI:1919118]
5	protein_coding	77234835	77262968	ENSMUSG00000059325	MGI:1916782	Hopx	HOP homeobox [Source:MGI Symbol;Acc:MGI:1916782]
5	protein_coding	77352954	77359318	ENSMUSG00000053030	MGI:1917232	Spink2	serine peptidase inhibitor, Kazal type 2 [Source:MGI Symbol;Acc:MGI:1917232]
5	protein_coding	77413338	77434279	ENSMUSG00000029249	MGI:104897	Rest	RE1-silencing transcription factor [Source:MGI Symbol;Acc:MGI:104897]
5	protein_coding	77442029	77457931	ENSMUSG00000036285	MGI:1914306	Noa1	nitric oxide associated 1 [Source:MGI Symbol;Acc:MGI:1914306]
5	protein_coding	77457994	77497171	ENSMUSG00000029250	MGI:2388280	Polr2b	[illegible] polypeptide B [Source:MGI Symbol;Acc:MGI:2388280]
5	protein_coding	77497087	77555888	ENSMUSG00000036256	MGI:1352480	Igfbp7	insulin-like growth factor binding protein 7 [Source:MGI Symbol;Acc:MGI:1352480]
5	protein_coding	81167985	81972980	ENSMUSG00000037605	MGI:2441950	Adgrl3	adhesion G protein-coupled receptor L3 [Source:MGI Symbol;Acc:MGI:2441950]
5	protein_coding	82270255	82272560	ENSMUSG00000099863	MGI:1914577	1700031L13Rik	RIKEN cDNA 1700031L13 gene [Source:MGI Symbol;Acc:MGI:1914577]
5	protein_coding	83425992	83503042	ENSMUSG00000049537	MGI:2444966	Tecrl	trans-2,3-enoyl-CoA reductase-like [Source:MGI Symbol;Acc:MGI:2444966]

5	protein_coding	84202620	84565241	ENSMUSG00000029245	MGI:99654	Epha5	Eph receptor A5 [Source:MGI Symbol;Acc:MGI:99654]
5	protein_coding	86159883	86213442	ENSMUSG00000029253	MGI:99700	Cenpc1	centromere protein C1 [Source:MGI Symbol;Acc:MGI:99700]
5	protein_coding	86219605	86253984	ENSMUSG00000029254	MGI:1926193	Stap1	signal transducing adaptor family member 1 [Source:MGI Symbol;Acc:MGI:1926193]
5	protein_coding	86257146	86320662	ENSMUSG00000035898	MGI:1913894	Uba6	ubiquitin-like modifier activating enzyme 6 [Source:MGI Symbol;Acc:MGI:1913894]
5	protein_coding	86328613	86345760	ENSMUSG00000029255	MGI:95790	Gnrhr	gonadotropin releasing hormone receptor [Source:MGI Symbol;Acc:MGI:95790]
5	protein_coding	86379340	86437167	ENSMUSG00000061184	MGI:3521861	Tmprss11c	transmembrane protease, serine 11c [Source:MGI Symbol;Acc:MGI:3521861]
5	protein_coding	86450076	86521279	ENSMUSG00000061259	MGI:2385221	Tmprss11d	transmembrane protease, serine 11d [Source:MGI Symbol;Acc:MGI:2385221]
5	protein_coding	86558269	86616849	ENSMUSG00000072845	MGI:2684853	Tmprss11a	transmembrane protease, serine 11a [Source:MGI Symbol;Acc:MGI:2684853]
5	protein_coding	86633735	86666459	ENSMUSG00000079451	MGI:2444058	Tmprss11g	transmembrane protease, serine 11g [Source:MGI Symbol;Acc:MGI:2444058]
5	protein_coding	86669757	86780283	ENSMUSG00000048764	MGI:2442348	Tmprss11f	transmembrane protease, serine 11f [Source:MGI Symbol;Acc:MGI:2442348]
5	protein_coding	86805490	86824221	ENSMUSG00000035861	MGI:2442893	Tmprss11b	transmembrane protease, serine 11B [Source:MGI Symbol;Acc:MGI:2442893]
5	protein_coding	86853045	86893674	ENSMUSG00000054537	MGI:3513175	Tmprss11e	transmembrane protease, serine 11e [Source:MGI Symbol;Acc:MGI:3513175]
5	protein_coding	86952080	86984518	ENSMUSG00000035851	MGI:2443713	Ythdc1	YTH domain containing 1 [Source:MGI Symbol;Acc:MGI:2443713]
5	protein_coding	87037626	87054796	ENSMUSG00000029260	MGI:2140962	Ugt2b34	polypeptide B34 [Source:MGI Symbol;Acc:MGI:2140962]
5	protein_coding	87064497	87074389	ENSMUSG00000035836	MGI:1919023	Ugt2b1	polypeptide B1 [Source:MGI Symbol;Acc:MGI:1919023]
5	protein_coding	87148719	87161134	ENSMUSG00000035811	MGI:3576100	Ugt2b35	polypeptide B35 [Source:MGI Symbol;Acc:MGI:3576100]
5	protein_coding	87213786	87240414	ENSMUSG00000070704	MGI:3576103	Ugt2b36	polypeptide B36 [Source:MGI Symbol;Acc:MGI:3576103]
5	protein_coding	87272819	87288177	ENSMUSG00000054630	MGI:98900	Ugt2b5	polypeptide B5 [Source:MGI Symbol;Acc:MGI:98900]

5	protein_coding	87388352	87402663	ENSMUSG00000057425	MGI:2148239	Ugt2b37	polypeptide B37 [Source:MGI Symbol;Acc:MGI:2148239]
5	protein_coding	87472831	87485054	ENSMUSG00000035780	MGI:1919344	Ugt2a3	polypeptide A3 [Source:MGI Symbol;Acc:MGI:1919344]
5	protein_coding	87557801	87572062	ENSMUSG00000061906	MGI:2140794	Ugt2b38	polypeptide B38 [Source:MGI Symbol;Acc:MGI:2140794]
5	protein_coding	87607349	87630119	ENSMUSG00000029268	MGI:3576095	Ugt2a2	polypeptide A2 [Source:MGI Symbol;Acc:MGI:3576095]
5	protein_coding	87607349	87638730	ENSMUSG00000106677	MGI:2149905	Ugt2a1	polypeptide A1 [Source:MGI Symbol;Acc:MGI:2149905]
5	protein_coding	87608099	87634665	ENSMUSG00000107180	MGI:5663775	Gm43638	predicted gene 43638 [Source:MGI Symbol;Acc:MGI:5663775]
5	protein_coding	87661198	87686054	ENSMUSG00000029269	MGI:2136282	Sult1b1	sulfotransferase family 1B, member 1 [Source:MGI Symbol;Acc:MGI:2136282]
5	protein_coding	87702504	87716886	ENSMUSG00000029273	MGI:1926341	Sult1d1	sulfotransferase family 1D, member 1 [Source:MGI Symbol;Acc:MGI:1926341]
5	protein_coding	87723828	87739453	ENSMUSG00000029272	MGI:98431	Sult1e1	sulfotransferase family 1E, member 1 [Source:MGI Symbol;Acc:MGI:98431]
5	protein_coding	87814067	87830437	ENSMUSG00000070702	MGI:88540	Csn1s1	casein alpha s1 [Source:MGI Symbol;Acc:MGI:88540]
5	protein_coding	87840483	87847489	ENSMUSG00000063157	MGI:88541	Csn2	casein beta [Source:MGI Symbol;Acc:MGI:88541]
5	protein_coding	87922426	87936656	ENSMUSG00000061937	MGI:88542	Csn1s2a	casein alpha s2-like A [Source:MGI Symbol;Acc:MGI:88542]
5	protein_coding	87955941	87972285	ENSMUSG00000061388	MGI:105312	Csn1s2b	casein alpha s2-like B [Source:MGI Symbol;Acc:MGI:105312]
5	protein_coding	87973545	87994246	ENSMUSG00000002240	MGI:1921029	Prr27	proline rich 27 [Source:MGI Symbol;Acc:MGI:1921029]
5	protein_coding	88032888	88042033	ENSMUSG00000009580	MGI:1916842	Odam	odontogenic, ameloblast asssociated [Source:MGI Symbol;Acc:MGI:1916842]
5	protein_coding	88056638	88062030	ENSMUSG00000105888	MGI:3512757	Fdcsp	follicular dendritic cell secreted protein [Source:MGI Symbol;Acc:MGI:3512757]
5	protein_coding	88073438	88080524	ENSMUSG00000001622	MGI:107461	Csn3	casein kappa [Source:MGI Symbol;Acc:MGI:107461]
5	protein_coding	88117318	88120729	ENSMUSG00000007457	MGI:1921498	2310003L06Rik	RIKEN cDNA 2310003L06 gene [Source:MGI Symbol;Acc:MGI:1921498]

5	protein_coding	88117376	88156136	ENSMUSG00000100704	MGI:5579140	Gm28434	predicted gene 28434 [Source:MGI Symbol;Acc:MGI:5579140]
5	protein_coding	88127298	88129403	ENSMUSG00000007907	MGI:1918227	Cabs1	calcium binding protein, spermatid specific 1 [Source:MGI Symbol;Acc:MGI:1918227]
5	protein_coding	88150408	88156393	ENSMUSG00000029280	MGI:102763	Smr3a	submaxillary gland androgen regulated protein 3A [Source:MGI Symbol;Acc:MGI:102763]
5	protein_coding	88234415	88256912	ENSMUSG00000029281	MGI:102762	Smr2	submaxillary gland androgen regulated protein 2 [Source:MGI Symbol;Acc:MGI:102762]
5	protein_coding	88416778	88430694	ENSMUSG00000090302	MGI:3646267	Smr2l	submaxillary gland androgen regulated protein 2 like [Source:MGI Symbol;Acc:MGI:3646267]
5	protein_coding	88465171	88476673	ENSMUSG00000064156	MGI:107496	Prol1	proline rich, lacrimal 1 [Source:MGI Symbol;Acc:MGI:107496]
5	protein_coding	88523967	88533775	ENSMUSG00000029282	MGI:1918671	Amtn	amelotin [Source:MGI Symbol;Acc:MGI:1918671]
5	protein_coding	88603850	88616390	ENSMUSG00000029288	MGI:104655	Ambn	ameloblastin [Source:MGI Symbol;Acc:MGI:104655]
5	protein_coding	88635834	88653908	ENSMUSG00000029286	MGI:1333772	Enam	enamelin [Source:MGI Symbol;Acc:MGI:1333772]
5	protein_coding	88667668	88675750	ENSMUSG00000067149	MGI:96493	Jchain	immunoglobulin joining chain [Source:MGI Symbol;Acc:MGI:96493]
5	protein_coding	88702321	88703949	ENSMUSG00000070697	MGI:1919230	Utp3	UTP3 small subunit processome component [Source:MGI Symbol;Acc:MGI:1919230]
5	protein_coding	88712899	88799251	ENSMUSG00000029291	MGI:106484	Rufy3	RUN and FYVE domain containing 3 [Source:MGI Symbol;Acc:MGI:106484]
5	protein_coding	88807307	88824030	ENSMUSG00000044221	MGI:106479	Grsf1	G-rich RNA sequence binding factor 1 [Source:MGI Symbol;Acc:MGI:106479]
5	protein_coding	88868714	88912079	ENSMUSG00000006262	MGI:1915723	Mob1b	MOB kinase activator 1B [Source:MGI Symbol;Acc:MGI:1915723]
5	protein_coding	88912855	88931140	ENSMUSG00000029366	MGI:102726	Dck	deoxycytidine kinase [Source:MGI Symbol;Acc:MGI:102726]
5	protein_coding	89034345	89387512	ENSMUSG00000060961	MGI:1927555	Slc4a4	[illegible] member 4 [Source:MGI Symbol;Acc:MGI:1927555]
5	protein_coding	89565381	89605757	ENSMUSG00000035540	MGI:95669	Gc	vitamin D binding protein [Source:MGI Symbol;Acc:MGI:95669]
5	protein_coding	89675288	89731599	ENSMUSG00000035528	MGI:1860130	Npffr2	neuropeptide FF receptor 2 [Source:MGI Symbol;Acc:MGI:1860130]

5	protein_coding	89824946	90031193	ENSMUSG00000043635	MGI:3045353	Adamts3	type 1 motif 3 [Source:MGI Symbol;Acc:MGI:3045353]
5	protein_coding	90362583	90371860	ENSMUSG00000035505	MGI:2448532	Cox18	cytochrome c oxidase assembly protein 18 [Source:MGI Symbol;Acc:MGI:2448532]
5	protein_coding	90375025	90514436	ENSMUSG00000055204	MGI:1932101	Ankrd17	ankyrin repeat domain 17 [Source:MGI Symbol;Acc:MGI:1932101]
5	protein_coding	90608756	90624461	ENSMUSG00000029368	MGI:87991	Alb	albumin [Source:MGI Symbol;Acc:MGI:87991]
5	protein_coding	90638596	90656766	ENSMUSG00000054932	MGI:87951	Afp	alpha fetoprotein [Source:MGI Symbol;Acc:MGI:87951]
5	protein_coding	90666791	90701402	ENSMUSG00000029369	MGI:2429409	Afm	afamin [Source:MGI Symbol;Acc:MGI:2429409]
5	protein_coding	90708966	90745730	ENSMUSG00000070690	MGI:1923342	Albfm1	albumin superfamily member 1 [Source:MGI Symbol;Acc:MGI:1923342]
5	protein_coding	90750935	90788516	ENSMUSG00000029370	MGI:1920496	Rassf6	member 6 [Source:MGI Symbol;Acc:MGI:1920496]
5	protein_coding	90907219	90909483	ENSMUSG00000029371	MGI:1096868	Cxcl5	C-X-C motif chemokine ligand 5 [Source:MGI Symbol;Acc:MGI:1096868]
5	protein_coding	90916377	90917922	ENSMUSG00000029372	MGI:1888712	Ppbp	pro-platelet basic protein [Source:MGI Symbol;Acc:MGI:1888712]
5	protein_coding	90920294	90921242	ENSMUSG00000029373	MGI:1888711	Pf4	platelet factor 4 [Source:MGI Symbol;Acc:MGI:1888711]
5	protein_coding	90933962	90937459	ENSMUSG00000029379	MGI:3037818	Cxcl3	C-X-C motif chemokine ligand 3 [Source:MGI Symbol;Acc:MGI:3037818]
5	protein_coding	90942393	90950926	ENSMUSG00000029375	MGI:1339941	Cxcl15	C-X-C motif chemokine ligand 15 [Source:MGI Symbol;Acc:MGI:1339941]
5	protein_coding	91039100	91040974	ENSMUSG00000029380	MGI:108068	Cxcl1	C-X-C motif chemokine ligand 1 [Source:MGI Symbol;Acc:MGI:108068]
5	protein_coding	91051730	91053797	ENSMUSG00000058427	MGI:1340094	Cxcl2	C-X-C motif chemokine ligand 2 [Source:MGI Symbol;Acc:MGI:1340094]
5	protein_coding	91078976	91169227	ENSMUSG00000029376	MGI:1915871	Mthfd2l	methylenetetrahydrofolate dehydrogenase (NADP+ dependent) 2-like [Source:MGI Symbol;Acc:MGI:1915871]
5	protein_coding	91175323	91183074	ENSMUSG00000035020	MGI:1919170	Epgn	epithelial mitogen [Source:MGI Symbol;Acc:MGI:1919170]

5	protein_coding	91222481	91241505	ENSMUSG00000029377	MGI:107508	Ereg	epiregulin [Source:MGI Symbol;Acc:MGI:107508]
5	protein_coding	91287458	91296291	ENSMUSG00000029378	MGI:88068	Areg	amphiregulin [Source:MGI Symbol;Acc:MGI:88068]
5	protein_coding	91505120	91550853	ENSMUSG00000082361	MGI:99439	Btc	betacellulin, epidermal growth factor family member [Source:MGI Symbol;Acc:MGI:99439]
5	protein_coding	91665474	91774753	ENSMUSG00000034981	MGI:2443349	Parm1	prostate androgen-regulated mucin-like protein 1 [Source:MGI Symbol;Acc:MGI:2443349]
5	protein_coding	92096763	92110927	ENSMUSG00000029397	MGI:1915348	Rchy1	[illegible] containing 1 [Source:MGI Symbol;Acc:MGI:1915348]
5	protein_coding	92135334	92143179	ENSMUSG00000096035	MGI:2685891	Odaph	odontogenesis associated phosphoprotein [Source:MGI Symbol;Acc:MGI:2685891]
5	protein_coding	92153933	92191742	ENSMUSG00000029403	MGI:1858227	Cdkl2	cyclin dependent kinase like 2 [Source:MGI Symbol;Acc:MGI:1858227]
5	protein_coding	92200005	92231578	ENSMUSG00000029405	MGI:2442040	G3bp2	G3BP stress granule assembly factor 2 [Source:MGI Symbol;Acc:MGI:2442040]
5	protein_coding	92285797	92350657	ENSMUSG00000029407	MGI:1929095	Uso1	USO1 vesicle docking factor [Source:MGI Symbol;Acc:MGI:1929095]
5	protein_coding	92357752	92363267	ENSMUSG00000029409	MGI:1930915	U90926	cDNA sequence U90926 [Source:MGI Symbol;Acc:MGI:1930915]
5	protein_coding	92374538	92404137	ENSMUSG00000029410	MGI:1342304	Ppef2	[illegible] domain 2 [Source:MGI Symbol;Acc:MGI:1342304]
5	protein_coding	92405518	92426029	ENSMUSG00000029413	MGI:1914361	Naaa	N-acylethanolamine acid amidase [Source:MGI Symbol;Acc:MGI:1914361]
5	protein_coding	92431869	92458338	ENSMUSG00000029415	MGI:2140779	Sdad1	SDA1 domain containing 1 [Source:MGI Symbol;Acc:MGI:2140779]
5	protein_coding	92469206	92475938	ENSMUSG00000029417	MGI:1352449	Cxcl9	C-X-C motif chemokine ligand 9 [Source:MGI Symbol;Acc:MGI:1352449]
5	protein_coding	92479686	92562487	ENSMUSG00000034842	MGI:1202729	Art3	ADP-ribosyltransferase 3 [Source:MGI Symbol;Acc:MGI:1202729]
5	protein_coding	92494497	92496748	ENSMUSG00000034855	MGI:1352450	Cxcl10	C-X-C motif chemokine ligand 10 [Source:MGI Symbol;Acc:MGI:1352450]
5	protein_coding	92507403	92513344	ENSMUSG00000060183	MGI:1860203	Cxcl11	chemokine (C-X-C motif) ligand 11 [Source:MGI Symbol;Acc:MGI:1860203]
5	protein_coding	92563399	92583078	ENSMUSG00000034826	MGI:1920460	Nup54	nucleoporin 54 [Source:MGI Symbol;Acc:MGI:1920460]

5	protein_coding	92589173	92654692	ENSMUSG00000029426	MGI:1196458	Scarb2	scavenger receptor class B, member 2 [Source:MGI Symbol;Acc:MGI:1196458]
5	protein_coding	92702928	92739138	ENSMUSG00000057068	MGI:2686227	Fam47e	family with sequence similarity 47, member E [Source:MGI Symbol;Acc:MGI:2686227]
5	protein_coding	92750900	92754438	ENSMUSG00000047963	MGI:1261768	Stbd1	starch binding domain 1 [Source:MGI Symbol;Acc:MGI:1261768]
5	protein_coding	92755813	92823130	ENSMUSG00000050050	MGI:2444555	Ccdc158	coiled-coil domain containing 158 [Source:MGI Symbol;Acc:MGI:2444555]
5	protein_coding	92831294	93113177	ENSMUSG00000029381	MGI:1351655	Shroom3	shroom family member 3 [Source:MGI Symbol;Acc:MGI:1351655]
5	protein_coding	93188982	93192881	ENSMUSG00000045314	MGI:1925338	Sowahb	[illegible] member B [Source:MGI Symbol;Acc:MGI:1925338]
5	protein_coding	93241296	93324306	ENSMUSG00000058013	MGI:1277214	Septin11	septin 11 [Source:MGI Symbol;Acc:MGI:1277214]
5	protein_coding	93329792	93354354	ENSMUSG00000063015	MGI:1341077	Ccni	cyclin I [Source:MGI Symbol;Acc:MGI:1341077]
5	protein_coding	93354377	93361334	ENSMUSG00000029384	MGI:1922860	2010109A12Rik	RIKEN cDNA 2010109A12 gene [Source:MGI Symbol;Acc:MGI:1922860]
5	protein_coding	93415116	93424090	ENSMUSG00000029385	MGI:1095734	Ccng2	cyclin G2 [Source:MGI Symbol;Acc:MGI:1095734]
5	protein_coding	93629688	93688059	ENSMUSG00000096044	MGI:3704103	Pramel33	PRAME like 33 [Source:MGI Symbol;Acc:MGI:3704103]
5	protein_coding	93783056	93819410	ENSMUSG00000070686	MGI:2141341	Pramel34	PRAME like 34 [Source:MGI Symbol;Acc:MGI:2141341]
5	protein_coding	93991623	94003966	ENSMUSG00000107392	MGI:3704104	Pramel35	PRAME like 35 [Source:MGI Symbol;Acc:MGI:3704104]
5	protein_coding	94072636	94076911	ENSMUSG00000096230	MGI:3704105	Pramel36	PRAME like 36 [Source:MGI Symbol;Acc:MGI:3704105]
5	protein_coding	94214310	94218404	ENSMUSG00000072822	MGI:2681870	Pramel37	PRAME like 37 [Source:MGI Symbol;Acc:MGI:2681870]
5	protein_coding	94224244	94228794	ENSMUSG00000072821	MGI:3647817	Pramel53	PRAME like 53 [Source:MGI Symbol;Acc:MGI:3647817]
5	protein_coding	94304436	94369394	ENSMUSG00000096259	MGI:3781282	Pramel38	PRAME like 38 [Source:MGI Symbol;Acc:MGI:3781282]
5	protein_coding	94460534	94465114	ENSMUSG00000095718	MGI:3779601	Pramel40	PRAME like 40 [Source:MGI Symbol;Acc:MGI:3779601]

5	protein_coding	94591865	94596410	ENSMUSG00000074011	MGI:3779756	Pramel41	PRAME like 41 [Source:MGI Symbol;Acc:MGI:3779756]
5	protein_coding	94674146	94694282	ENSMUSG00000095074	MGI:3781318	Pramel42	PRAME like 42 [Source:MGI Symbol;Acc:MGI:3781318]
5	protein_coding	94759928	94764201	ENSMUSG00000095503	MGI:3781326	Pramel43	PRAME like 43 [Source:MGI Symbol;Acc:MGI:3781326]
5	protein_coding	94770074	94774605	ENSMUSG00000094195	MGI:3779602	Pramel44	PRAME like 44 [Source:MGI Symbol;Acc:MGI:3779602]
5	protein_coding	95013295	95033247	ENSMUSG00000095954	MGI:3781362	Pramel56	PRAME like 56 [Source:MGI Symbol;Acc:MGI:3781362]
5	protein_coding	95254563	95259017	ENSMUSG00000092166	MGI:3646599	Pramel45	PRAME like 45 [Source:MGI Symbol;Acc:MGI:3646599]
5	protein_coding	95330483	95342825	ENSMUSG00000095996	MGI:2141041	Pramel60	PRAME like 60 [Source:MGI Symbol;Acc:MGI:2141041]
5	protein_coding	95416352	95420627	ENSMUSG00000096066	MGI:3704250	Pramel46	PRAME like 46 [Source:MGI Symbol;Acc:MGI:3704250]
5	protein_coding	95453816	95491776	ENSMUSG00000079424	MGI:3781437	Pramel47	PRAME like 47 [Source:MGI Symbol;Acc:MGI:3781437]
5	protein_coding	95604665	95633446	ENSMUSG00000070677	MGI:1261918	Pramel48	PRAME like 48 [Source:MGI Symbol;Acc:MGI:1261918]
5	protein_coding	95658617	95670261	ENSMUSG00000079423	MGI:3781464	Pramel57	PRAME like 57 [Source:MGI Symbol;Acc:MGI:3781464]
5	protein_coding	95738458	95742734	ENSMUSG00000094043	MGI:3779772	Pramel49	PRAME like 49 [Source:MGI Symbol;Acc:MGI:3779772]
5	protein_coding	95879648	95884032	ENSMUSG00000096139	MGI:3704107	Pramel50	PRAME like 50 [Source:MGI Symbol;Acc:MGI:3704107]
5	protein_coding	95889873	95894414	ENSMUSG00000072814	MGI:3648891	Pramel54	PRAME like 54 [Source:MGI Symbol;Acc:MGI:3648891]
5	protein_coding	95923729	95952089	ENSMUSG00000072813	MGI:3615333	Pramel55	PRAME like 55 [Source:MGI Symbol;Acc:MGI:3615333]
5	protein_coding	96104810	96108927	ENSMUSG00000023078	MGI:1888499	Cxcl13	C-X-C motif chemokine ligand 13 [Source:MGI Symbol;Acc:MGI:1888499]
5	protein_coding	96218192	96312030	ENSMUSG00000034724	MGI:2443154	Cnot6l	CCR4-NOT transcription complex, subunit 6-like [Source:MGI Symbol;Acc:MGI:2443154]
5	protein_coding	96357352	96414586	ENSMUSG00000029486	MGI:2137202	Mrpl1	mitochondrial ribosomal protein L1 [Source:MGI Symbol;Acc:MGI:2137202]

5	protein_coding	96521814	96932587	ENSMUSG00000034687	MGI:2385368	Fras1	Fraser extracellular matrix complex subunit 1 [Source:MGI Symbol;Acc:MGI:2385368]
5	protein_coding	96941198	96993825	ENSMUSG00000029484	MGI:1201378	Anxa3	annexin A3 [Source:MGI Symbol;Acc:MGI:1201378]
5	protein_coding	97145548	97239726	ENSMUSG00000034663	MGI:2155456	Bmp2k	BMP2 inducible kinase [Source:MGI Symbol;Acc:MGI:2155456]
5	protein_coding	97230188	97259455	ENSMUSG00000055725	MGI:2679683	Paqr3	progestin and adipoQ receptor family member III [Source:MGI Symbol;Acc:MGI:2679683]
5	protein_coding	97530057	97540238	ENSMUSG00000046000	MGI:2141314	Naa11	[illegible] subunit [Source:MGI Symbol;Acc:MGI:2141314]
5	protein_coding	97603001	97604880	ENSMUSG00000050553	MGI:1329027	Gk2	glycerol kinase 2 [Source:MGI Symbol;Acc:MGI:1329027]
5	protein_coding	98030642	98178902	ENSMUSG00000029338	MGI:1919164	Antxr2	anthrax toxin receptor 2 [Source:MGI Symbol;Acc:MGI:1919164]
5	protein_coding	98315057	98336850	ENSMUSG00000035456	MGI:1924880	Prdm8	PR domain containing 8 [Source:MGI Symbol;Acc:MGI:1924880]
5	protein_coding	98402043	98424889	ENSMUSG00000029337	MGI:95519	Fgf5	fibroblast growth factor 5 [Source:MGI Symbol;Acc:MGI:95519]
5	protein_coding	98477163	98949906	ENSMUSG00000057816	MGI:1916571	Cfap299	cilia and flagella associated protein 299 [Source:MGI Symbol;Acc:MGI:1916571]
5	protein_coding	99002274	99032255	ENSMUSG00000029335	MGI:88179	Bmp3	bone morphogenetic protein 3 [Source:MGI Symbol;Acc:MGI:88179]
5	protein_coding	99077632	99185210	ENSMUSG00000029334	MGI:108173	Prkg2	protein kinase, cGMP-dependent, type II [Source:MGI Symbol;Acc:MGI:108173]
5	protein_coding	99365285	99876924	ENSMUSG00000089809	MGI:2443755	Rasgef1b	RasGEF domain family, member 1B [Source:MGI Symbol;Acc:MGI:2443755]
5	protein_coding	1.00E+08	1.00E+08	ENSMUSG00000105078	MGI:5595070	Vamp9	vesicle-associated membrane protein 9 [Source:MGI Symbol;Acc:MGI:5595070]
5	protein_coding	1.00E+08	1.00E+08	ENSMUSG00000000568	MGI:101947	Hnrnpd	heterogeneous nuclear ribonucleoprotein D [Source:MGI Symbol;Acc:MGI:101947]
5	protein_coding	1.00E+08	1.00E+08	ENSMUSG00000029328	MGI:1355299	Hnrnpdl	heterogeneous nuclear ribonucleoprotein D-like [Source:MGI Symbol;Acc:MGI:1355299]
5	protein_coding	1.00E+08	1.00E+08	ENSMUSG00000029326	MGI:1915120	Enoph1	enolase-phosphatase 1 [Source:MGI Symbol;Acc:MGI:1915120]
5	protein_coding	1.00E+08	1.00E+08	ENSMUSG00000050640	MGI:3041258	Tmem150c	transmembrane protein 150C [Source:MGI Symbol;Acc:MGI:3041258]

5	protein_coding	1.01E+08	1.01E+08	ENSMUSG00000035325	MGI:1916412	Sec31a	[illegible] component [Source:MGI Symbol;Acc:MGI:1916412]
5	protein_coding	1.01E+08	1.01E+08	ENSMUSG00000118665	MGI:2140902	Lin54	lin-54 DREAM MuvB core complex component [Source:MGI Symbol;Acc:MGI:2140902]
5	protein_coding	1.01E+08	1.01E+08	ENSMUSG00000035297	MGI:1349414	Cops4	COP9 signalosome subunit 4 [Source:MGI Symbol;Acc:MGI:1349414]
5	protein_coding	1.01E+08	1.01E+08	ENSMUSG00000029322	MGI:2445289	Plac8	placenta-specific 8 [Source:MGI Symbol;Acc:MGI:2445289]
5	protein_coding	1.01E+08	1.01E+08	ENSMUSG00000029319	MGI:1919133	Coq2	coenzyme Q2 4-hydroxybenzoate polyprenyltransferase [Source:MGI Symbol;Acc:MGI:1919133]
5	protein_coding	1.01E+08	1.01E+08	ENSMUSG00000035273	MGI:1343124	Hpse	heparanase [Source:MGI Symbol;Acc:MGI:1343124]
5	protein_coding	1.01E+08	1.01E+08	ENSMUSG00000035266	MGI:2176740	Helq	helicase, POLQ-like [Source:MGI Symbol;Acc:MGI:2176740]
5	protein_coding	1.01E+08	1.01E+08	ENSMUSG00000016833	MGI:1915985	Mrps18c	mitochondrial ribosomal protein S18C [Source:MGI Symbol;Acc:MGI:1915985]
5	protein_coding	1.01E+08	1.01E+08	ENSMUSG00000035234	MGI:1917931	Abraxas1	BRCA1 A complex subunit [Source:MGI Symbol;Acc:MGI:1917931]
5	protein_coding	1.01E+08	1.01E+08	ENSMUSG00000029314	MGI:3603816	Gpat3	glycerol-3-phosphate acyltransferase 3 [Source:MGI Symbol;Acc:MGI:3603816]
5	protein_coding	1.02E+08	1.02E+08	ENSMUSG00000035187	MGI:1206039	Nkx6-1	NK6 homeobox 1 [Source:MGI Symbol;Acc:MGI:1206039]
5	protein_coding	1.02E+08	1.02E+08	ENSMUSG00000029330	MGI:1921846	Cds1	CDP-diacylglycerol synthase 1 [Source:MGI Symbol;Acc:MGI:1921846]
5	protein_coding	1.02E+08	1.02E+08	ENSMUSG00000043940	MGI:1096875	Wdfy3	WD repeat and FYVE domain containing 3 [Source:MGI Symbol;Acc:MGI:1096875]
5	protein_coding	1.03E+08	1.03E+08	ENSMUSG00000057315	MGI:1922647	Arhgap24	Rho GTPase activating protein 24 [Source:MGI Symbol;Acc:MGI:1922647]
5	protein_coding	1.03E+08	1.03E+08	ENSMUSG00000046709	MGI:1346863	Mapk10	mitogen-activated protein kinase 10 [Source:MGI Symbol;Acc:MGI:1346863]
5	protein_coding	1.04E+08	1.04E+08	ENSMUSG00000034573	MGI:103293	Ptpn13	protein tyrosine phosphatase, non-receptor type 13 [Source:MGI Symbol;Acc:MGI:103293]
5	protein_coding	1.04E+08	1.04E+08	ENSMUSG00000029321	MGI:1923000	Slc10a6	solute carrier family 10 (sodium/bile acid cotransporter family), member 6 [Source:MGI Symbol;Acc:MGI:1923000]

5	protein_coding	1.04E+08	1.04E+08	ENSMUSG00000029320	MGI:1921468	1700016H13Rik	RIKEN cDNA 1700016H13 gene [Source:MGI Symbol;Acc:MGI:1921468]
5	protein_coding	1.04E+08	1.04E+08	ENSMUSG00000029313	MGI:1100819	Aff1	AF4/FMR2 family, member 1 [Source:MGI Symbol;Acc:MGI:1100819]
5	protein_coding	1.04E+08	1.04E+08	ENSMUSG00000029312	MGI:2179430	Klhl8	kelch-like 8 [Source:MGI Symbol;Acc:MGI:2179430]
5	protein_coding	1.04E+08	1.04E+08	ENSMUSG00000034528	MGI:2140804	Hsd17b13	hydroxysteroid (17-beta) dehydrogenase 13 [Source:MGI Symbol;Acc:MGI:2140804]
5	protein_coding	1.04E+08	1.04E+08	ENSMUSG00000029311	MGI:2149821	Hsd17b11	hydroxysteroid (17-beta) dehydrogenase 11 [Source:MGI Symbol;Acc:MGI:2149821]
5	protein_coding	1.04E+08	1.04E+08	ENSMUSG00000029310	MGI:1921417	Nudt9	nudix hydrolase 9 [Source:MGI Symbol;Acc:MGI:1921417]
5	protein_coding	1.04E+08	1.04E+08	ENSMUSG00000091034	MGI:4937294	Scpppq1	secretory calcium-binding phosphoprotein proline-glutamine rich 1 [Source:MGI Symbol;Acc:MGI:4937294]
5	protein_coding	1.04E+08	1.04E+08	ENSMUSG00000029309	MGI:108110	Sparcl1	SPARC-like 1 [Source:MGI Symbol;Acc:MGI:108110]
5	protein_coding	1.04E+08	1.04E+08	ENSMUSG00000053268	MGI:109172	Dspp	dentin sialophosphoprotein [Source:MGI Symbol;Acc:MGI:109172]
5	protein_coding	1.04E+08	1.04E+08	ENSMUSG00000029307	MGI:94910	Dmp1	dentin matrix protein 1 [Source:MGI Symbol;Acc:MGI:94910]
5	protein_coding	1.04E+08	1.04E+08	ENSMUSG00000029306	MGI:96389	Ibsp	integrin binding sialoprotein [Source:MGI Symbol;Acc:MGI:96389]
5	protein_coding	1.04E+08	1.04E+08	ENSMUSG00000053863	MGI:2137384	Mepe	matrix extracellular phosphoglycoprotein with ASARM motif (bone) [Source:MGI Symbol;Acc:MGI:2137384]
5	protein_coding	1.05E+08	1.05E+08	ENSMUSG00000029304	MGI:98389	Spp1	secreted phosphoprotein 1 [Source:MGI Symbol;Acc:MGI:98389]
5	protein_coding	1.05E+08	1.05E+08	ENSMUSG00000034462	MGI:1099818	Pkd2	[illegible] channel [Source:MGI Symbol;Acc:MGI:1099818]
5	protein_coding	1.05E+08	1.05E+08	ENSMUSG00000097392	MGI:3040669	Thoc2l	THO complex subunit 2-like [Source:MGI Symbol;Acc:MGI:3040669]
5	protein_coding	1.05E+08	1.05E+08	ENSMUSG00000120166	MGI:1916873	Zfp33b	zinc finger protein 33B [Source:MGI Symbol;Acc:MGI:1916873]
5	protein_coding	1.05E+08	1.05E+08	ENSMUSG00000072774	MGI:2441896	Zfp951	zinc finger protein 951 [Source:MGI Symbol;Acc:MGI:2441896]

5	protein_coding	1.05E+08	1.05E+08	ENSMUSG00000029299	MGI:1351624	Abcg3	ATP binding cassette subfamily G member 3 [Source:MGI Symbol;Acc:MGI:1351624]
5	protein_coding	1.05E+08	1.05E+08	ENSMUSG00000034438	MGI:1923324	Gbp8	guanylate-binding protein 8 [Source:MGI Symbol;Acc:MGI:1923324]
5	protein_coding	1.05E+08	1.05E+08	ENSMUSG00000029298	MGI:3605620	Gbp9	guanylate-binding protein 9 [Source:MGI Symbol;Acc:MGI:3605620]
5	protein_coding	1.05E+08	1.05E+08	ENSMUSG00000079363	MGI:97072	Gbp4	guanylate binding protein 4 [Source:MGI Symbol;Acc:MGI:97072]
5	protein_coding	1.05E+08	1.05E+08	ENSMUSG00000079362	MGI:5663439	Gm43302	predicted gene 43302 [Source:MGI Symbol;Acc:MGI:5663439]
5	protein_coding	1.05E+08	1.05E+08	ENSMUSG00000105096	MGI:4359647	Gbp10	guanylate-binding protein 10 [Source:MGI Symbol;Acc:MGI:4359647]
5	protein_coding	1.05E+08	1.05E+08	ENSMUSG00000104713	MGI:2140937	Gbp6	guanylate binding protein 6 [Source:MGI Symbol;Acc:MGI:2140937]
5	protein_coding	1.05E+08	1.05E+08	ENSMUSG00000092021	MGI:3646307	Gbp11	guanylate binding protein 11 [Source:MGI Symbol;Acc:MGI:3646307]
5	protein_coding	1.06E+08	1.06E+08	ENSMUSG00000070639	MGI:2141353	Lrrc8b	[illegible] member B [Source:MGI Symbol;Acc:MGI:2141353]
5	protein_coding	1.06E+08	1.06E+08	ENSMUSG00000054720	MGI:2140839	Lrrc8c	[illegible] member C [Source:MGI Symbol;Acc:MGI:2140839]
5	protein_coding	1.06E+08	1.06E+08	ENSMUSG00000046079	MGI:1922368	Lrrc8d	leucine rich repeat containing 8D [Source:MGI Symbol;Acc:MGI:1922368]
5	protein_coding	1.06E+08	1.06E+08	ENSMUSG00000029290	MGI:1927246	Zfp326	zinc finger protein 326 [Source:MGI Symbol;Acc:MGI:1927246]
5	protein_coding	1.07E+08	1.07E+08	ENSMUSG00000034384	MGI:1859314	Barhl2	BarH like homeobox 2 [Source:MGI Symbol;Acc:MGI:1859314]
5	protein_coding	1.07E+08	1.07E+08	ENSMUSG00000049606	MGI:1277212	Zfp644	zinc finger protein 644 [Source:MGI Symbol;Acc:MGI:1277212]
5	protein_coding	1.07E+08	1.07E+08	ENSMUSG00000043410	MGI:3036246	Hfm1	HFM1, ATP-dependent DNA helicase homolog [Source:MGI Symbol;Acc:MGI:3036246]
5	protein_coding	1.07E+08	1.07E+08	ENSMUSG00000029283	MGI:1309511	Cdc7	cell division cycle 7 [Source:MGI Symbol;Acc:MGI:1309511]
5	protein_coding	1.07E+08	1.07E+08	ENSMUSG00000029287	MGI:104637	Tgfbr3	transforming growth factor, beta receptor III [Source:MGI Symbol;Acc:MGI:104637]
5	protein_coding	1.07E+08	1.08E+08	ENSMUSG00000029279	MGI:1891374	Brdt	bromodomain, testis-specific [Source:MGI Symbol;Acc:MGI:1891374]

5	protein_coding	1.08E+08	1.08E+08	ENSMUSG00000033805	MGI:2686228	Ephx4	epoxide hydrolase 4 [Source:MGI Symbol;Acc:MGI:2686228]
5	protein_coding	1.08E+08	1.08E+08	ENSMUSG00000033794	MGI:1918152	Lpcat2b	lysophosphatidylcholine acyltransferase 2B [Source:MGI Symbol;Acc:MGI:1918152]
5	protein_coding	1.08E+08	1.08E+08	ENSMUSG00000111375	MGI:3646208	Btbd8	BTB domain containing 8 [Source:MGI Symbol;Acc:MGI:3646208]
5	protein_coding	1.08E+08	1.08E+08	ENSMUSG00000106631	MGI:5662806	Gm42669	predicted gene 42669 [Source:MGI Symbol;Acc:MGI:5662806]
5	protein_coding	1.08E+08	1.08E+08	ENSMUSG00000089798	MGI:1923671	1700028K03Rik	RIKEN cDNA 1700028K03 gene [Source:MGI Symbol;Acc:MGI:1923671]
5	protein_coding	1.08E+08	1.08E+08	ENSMUSG00000029276	MGI:2141180	Glmn	glomulin, FKBP associated protein [Source:MGI Symbol;Acc:MGI:2141180]
5	protein_coding	1.08E+08	1.08E+08	ENSMUSG00000033773	MGI:2141142	Rpap2	RNA polymerase II associated protein 2 [Source:MGI Symbol;Acc:MGI:2141142]
5	protein_coding	1.08E+08	1.08E+08	ENSMUSG00000029275	MGI:103170	Gfi1	[illegible] repressor [Source:MGI Symbol;Acc:MGI:103170]
5	protein_coding	1.08E+08	1.08E+08	ENSMUSG00000011831	MGI:104736	Evi5	ecotropic viral integration site 5 [Source:MGI Symbol;Acc:MGI:104736]
5	protein_coding	1.08E+08	1.08E+08	ENSMUSG00000063447	MGI:1920568	Ube2d2b	ubiquitin-conjugating enzyme E2D 2B [Source:MGI Symbol;Acc:MGI:1920568]
5	protein_coding	1.08E+08	1.08E+08	ENSMUSG00000058558	MGI:102854	Rpl5	ribosomal protein L5 [Source:MGI Symbol;Acc:MGI:102854]
5	protein_coding	1.08E+08	1.08E+08	ENSMUSG00000029270	MGI:1914516	Dipk1a	divergent protein kinase domain 1A [Source:MGI Symbol;Acc:MGI:1914516]
5	protein_coding	1.08E+08	1.08E+08	ENSMUSG00000029267	MGI:105050	Mtf2	metal response element binding transcription factor 2 [Source:MGI Symbol;Acc:MGI:105050]
5	protein_coding	1.08E+08	1.08E+08	ENSMUSG00000063406	MGI:1921586	Tmed5	transmembrane p24 trafficking protein 5 [Source:MGI Symbol;Acc:MGI:1921586]
5	protein_coding	1.08E+08	1.08E+08	ENSMUSG00000056531	MGI:1922974	Ccdc18	coiled-coil domain containing 18 [Source:MGI Symbol;Acc:MGI:1922974]
5	protein_coding	1.08E+08	1.08E+08	ENSMUSG00000029265	MGI:1100515	Dr1	down-regulator of transcription 1 [Source:MGI Symbol;Acc:MGI:1100515]
5	protein_coding	1.08E+08	1.08E+08	ENSMUSG00000029263	MGI:3576484	Pigg	[illegible] biosynthesis, class G [Source:MGI Symbol;Acc:MGI:3576484]
5	protein_coding	1.09E+08	1.09E+08	ENSMUSG00000105867	MGI:5662654	Tmem271rt	transmembrane protein 271, retrotransposed [Source:MGI Symbol;Acc:MGI:5662654]

5	protein_coding	1.09E+08	1.09E+08	ENSMUSG00000029491	MGI:97525	Pde6b	beta polypeptide [Source:MGI Symbol;Acc:MGI:97525]
5	protein_coding	1.09E+08	1.09E+08	ENSMUSG00000050856	MGI:106636	Atp5me	ATP synthase membrane subunit e [Source:MGI Symbol;Acc:MGI:106636]
5	protein_coding	1.09E+08	1.09E+08	ENSMUSG00000029490	MGI:2442629	Slc49a3	solute carrier family 49 member 3 [Source:MGI Symbol;Acc:MGI:2442629]
5	protein_coding	1.09E+08	1.09E+08	ENSMUSG00000033623	MGI:1916837	Pcgf3	polycomb group ring finger 3 [Source:MGI Symbol;Acc:MGI:1916837]
5	protein_coding	1.09E+08	1.09E+08	ENSMUSG00000033615	MGI:104727	Cplx1	complexin 1 [Source:MGI Symbol;Acc:MGI:104727]
5	protein_coding	1.09E+08	1.09E+08	ENSMUSG00000062234	MGI:2442153	Gak	cyclin G associated kinase [Source:MGI Symbol;Acc:MGI:2442153]
5	protein_coding	1.09E+08	1.09E+08	ENSMUSG00000013495	MGI:1919642	Tmem175	transmembrane protein 175 [Source:MGI Symbol;Acc:MGI:1919642]
5	protein_coding	1.09E+08	1.09E+08	ENSMUSG00000004815	MGI:102918	Dgkq	diacylglycerol kinase, theta [Source:MGI Symbol;Acc:MGI:102918]
5	protein_coding	1.09E+08	1.09E+08	ENSMUSG00000033540	MGI:96418	Idua	iduronidase, alpha-L [Source:MGI Symbol;Acc:MGI:96418]
5	protein_coding	1.09E+08	1.09E+08	ENSMUSG00000046959	MGI:2385894	Slc26a1	member 1 [Source:MGI Symbol;Acc:MGI:2385894]
5	protein_coding	1.09E+08	1.09E+08	ENSMUSG00000008090	MGI:2150920	Fgfrl1	fibroblast growth factor receptor-like 1 [Source:MGI Symbol;Acc:MGI:2150920]
5	protein_coding	1.09E+08	1.09E+08	ENSMUSG00000055385	MGI:3645767	Rnf212	ring finger protein 212 [Source:MGI Symbol;Acc:MGI:3645767]
5	protein_coding	1.09E+08	1.09E+08	ENSMUSG00000004821	MGI:1914616	Tmed11	transmembrane p24 trafficking protein 11 [Source:MGI Symbol;Acc:MGI:1914616]
5	protein_coding	1.09E+08	1.09E+08	ENSMUSG00000090961	MGI:3642986	Vmn2r8	vomeronasal 2, receptor 8 [Source:MGI Symbol;Acc:MGI:3642986]
5	protein_coding	1.09E+08	1.09E+08	ENSMUSG00000091624	MGI:3643093	Vmn2r9	vomeronasal 2, receptor 9 [Source:MGI Symbol;Acc:MGI:3643093]
5	protein_coding	1.09E+08	1.09E+08	ENSMUSG00000067010	MGI:1316730	Vmn2r10	vomeronasal 2, receptor 10 [Source:MGI Symbol;Acc:MGI:1316730]
5	protein_coding	1.09E+08	1.09E+08	ENSMUSG00000091450	MGI:3643806	Vmn2r11	vomeronasal 2, receptor 11 [Source:MGI Symbol;Acc:MGI:3643806]
5	protein_coding	1.09E+08	1.09E+08	ENSMUSG00000090688	MGI:3761377	Vmn2r12	vomeronasal 2, receptor 12 [Source:MGI Symbol;Acc:MGI:3761377]

5	protein_coding	1.09E+08	1.09E+08	ENSMUSG00000091635	MGI:3761379	Vmn2r13	vomeronasal 2, receptor 13 [Source:MGI Symbol;Acc:MGI:3761379]
5	protein_coding	1.09E+08	1.09E+08	ENSMUSG00000091059	MGI:3649151	Vmn2r14	vomeronasal 2, receptor 14 [Source:MGI Symbol;Acc:MGI:3649151]
5	protein_coding	1.09E+08	1.09E+08	ENSMUSG00000091375	MGI:3649165	Vmn2r15	vomeronasal 2, receptor 15 [Source:MGI Symbol;Acc:MGI:3649165]
5	protein_coding	1.09E+08	1.10E+08	ENSMUSG00000092080	MGI:3647194	Vmn2r16	vomeronasal 2, receptor 16 [Source:MGI Symbol;Acc:MGI:3647194]
5	protein_coding	1.10E+08	1.10E+08	ENSMUSG00000091879	MGI:3647193	Vmn2r17	vomeronasal 2, receptor 17 [Source:MGI Symbol;Acc:MGI:3647193]
5	protein_coding	1.10E+08	1.10E+08	ENSMUSG00000033467	MGI:1889506	Crlf2	cytokine receptor-like factor 2 [Source:MGI Symbol;Acc:MGI:1889506]
5	protein_coding	1.10E+08	1.10E+08	ENSMUSG00000072763	MGI:1924450	Zfp1007	zinc finger protein 1007 [Source:MGI Symbol;Acc:MGI:1924450]
5	protein_coding	1.10E+08	1.10E+08	ENSMUSG00000072762	MGI:1925270	4930522L14Rik	RIKEN cDNA 4930522L14 gene [Source:MGI Symbol;Acc:MGI:1925270]
5	protein_coding	1.10E+08	1.10E+08	ENSMUSG00000121596	MGI:3642128	AB010352	cDNA sequence AB010352 [Source:MGI Symbol;Acc:MGI:3642128]
5	protein_coding	1.10E+08	1.10E+08	ENSMUSG00000090015	MGI:3709333	Gm15446	predicted gene 15446 [Source:MGI Symbol;Acc:MGI:3709333]
5	protein_coding	1.10E+08	1.10E+08	ENSMUSG00000121597			novel KRAB containing domain zinc finger protein
5	protein_coding	1.10E+08	1.10E+08	ENSMUSG00000066613	MGI:1916754	Zfp932	zinc finger protein 932 [Source:MGI Symbol;Acc:MGI:1916754]
5	protein_coding	1.10E+08	1.10E+08	ENSMUSG00000090963	MGI:4937289	Gm17655	predicted gene, 17655 [Source:MGI Symbol;Acc:MGI:4937289]
5	protein_coding	1.10E+08	1.10E+08	ENSMUSG00000109771	MGI:5594474	Gm35315	predicted gene, 35315 [Source:MGI Symbol;Acc:MGI:5594474]
5	protein_coding	1.10E+08	1.10E+08	ENSMUSG00000064247	MGI:2685422	Plcxd1	phosphatidylinositol-specific phospholipase C, X domain containing 1 [Source:MGI Symbol;Acc:MGI:2685422]
5	protein_coding	1.10E+08	1.10E+08	ENSMUSG00000033434	MGI:1306825	Gtpbp6	GTP binding protein 6 (putative) [Source:MGI Symbol;Acc:MGI:1306825]
5	protein_coding	1.10E+08	1.10E+08	ENSMUSG00000023284	MGI:2444933	Zfp605	zinc finger protein 605 [Source:MGI Symbol;Acc:MGI:2444933]

5	protein_coding	1.10E+08	1.10E+08	ENSMUSG00000014668	MGI:2444898	Chfr	[illegible] domains [Source:MGI Symbol;Acc:MGI:2444898]
5	protein_coding	1.10E+08	1.10E+08	ENSMUSG00000029502	MGI:96958	Golga3	golgin A3 [Source:MGI Symbol;Acc:MGI:96958]
5	protein_coding	1.10E+08	1.10E+08	ENSMUSG00000029501	MGI:1261856	Ankle2	ankyrin repeat and LEM domain containing 2 [Source:MGI Symbol;Acc:MGI:1261856]
5	protein_coding	1.10E+08	1.10E+08	ENSMUSG00000029500	MGI:1919792	Pgam5	phosphoglycerate mutase family member 5 [Source:MGI Symbol;Acc:MGI:1919792]
5	protein_coding	1.10E+08	1.10E+08	ENSMUSG00000029499	MGI:107487	Pxmp2	peroxisomal membrane protein 2 [Source:MGI Symbol;Acc:MGI:107487]
5	protein_coding	1.10E+08	1.10E+08	ENSMUSG00000007080	MGI:1196391	Pole	polymerase (DNA directed), epsilon [Source:MGI Symbol;Acc:MGI:1196391]
5	protein_coding	1.10E+08	1.10E+08	ENSMUSG00000029503	MGI:2665170	P2rx2	[illegible] channel, 2 [Source:MGI Symbol;Acc:MGI:2665170]
5	protein_coding	1.10E+08	1.11E+08	ENSMUSG00000072754	MGI:2686525	Lrcol1	leucine rich colipase-like 1 [Source:MGI Symbol;Acc:MGI:2686525]
5	protein_coding	1.11E+08	1.11E+08	ENSMUSG00000043323	MGI:1920907	Fbrsl1	fibrosin-like 1 [Source:MGI Symbol;Acc:MGI:1920907]
5	protein_coding	1.11E+08	1.11E+08	ENSMUSG00000033316	MGI:2677965	Galnt9	polypeptide N-acetylgalactosaminyltransferase 9 [Source:MGI Symbol;Acc:MGI:2677965]
5	protein_coding	1.11E+08	1.11E+08	ENSMUSG00000033294	MGI:2140843	Noc4l	NOC4 like [Source:MGI Symbol;Acc:MGI:2140843]
5	protein_coding	1.11E+08	1.11E+08	ENSMUSG00000029504	MGI:1916913	Ddx51	DEAD box helicase 51 [Source:MGI Symbol;Acc:MGI:1916913]
5	protein_coding	1.11E+08	1.11E+08	ENSMUSG00000029505	MGI:1276124	Ep400	E1A binding protein p400 [Source:MGI Symbol;Acc:MGI:1276124]
5	protein_coding	1.11E+08	1.11E+08	ENSMUSG00000029507	MGI:1929237	Pus1	pseudouridine synthase 1 [Source:MGI Symbol;Acc:MGI:1929237]
5	protein_coding	1.11E+08	1.11E+08	ENSMUSG00000029512	MGI:1270126	Ulk1	unc-51 like kinase 1 [Source:MGI Symbol;Acc:MGI:1270126]
5	protein_coding	1.11E+08	1.11E+08	ENSMUSG00000043510	MGI:2141135	Hscb	HscB iron-sulfur cluster co-chaperone [Source:MGI Symbol;Acc:MGI:2141135]
5	protein_coding	1.11E+08	1.11E+08	ENSMUSG00000029521	MGI:1355321	Chek2	checkpoint kinase 2 [Source:MGI Symbol;Acc:MGI:1355321]
5	protein_coding	1.11E+08	1.11E+08	ENSMUSG00000033209	MGI:2140873	Ttc28	tetratricopeptide repeat domain 28 [Source:MGI Symbol;Acc:MGI:2140873]

5	protein_coding	1.11E+08	1.12E+08	ENSMUSG00000050017	MGI:1927542	Pitpnb	phosphatidylinositol transfer protein, beta [Source:MGI Symbol;Acc:MGI:1927542]
5	protein_coding	1.12E+08	1.12E+08	ENSMUSG00000070576	MGI:1261813	Mn1	meningioma 1 [Source:MGI Symbol;Acc:MGI:1261813]
5	protein_coding	1.12E+08	1.12E+08	ENSMUSG00000096949	MGI:5477391	Gm26897	predicted gene, 26897 [Source:MGI Symbol;Acc:MGI:5477391]
5	protein_coding	1.12E+08	1.12E+08	ENSMUSG00000066975	MGI:102716	Cryba4	crystallin, beta A4 [Source:MGI Symbol;Acc:MGI:102716]
5	protein_coding	1.12E+08	1.12E+08	ENSMUSG00000029343	MGI:104992	Crybb1	crystallin, beta B1 [Source:MGI Symbol;Acc:MGI:104992]
5	protein_coding	1.12E+08	1.12E+08	ENSMUSG00000029344	MGI:1309516	Tpst2	protein-tyrosine sulfotransferase 2 [Source:MGI Symbol;Acc:MGI:1309516]
5	protein_coding	1.12E+08	1.12E+08	ENSMUSG00000029345	MGI:1930075	Tfip11	tuftelin interacting protein 11 [Source:MGI Symbol;Acc:MGI:1930075]
5	protein_coding	1.12E+08	1.12E+08	ENSMUSG00000029346	MGI:1917368	Srrd	SRR1 domain containing [Source:MGI Symbol;Acc:MGI:1917368]
5	protein_coding	1.12E+08	1.13E+08	ENSMUSG00000042328	MGI:2177742	Hps4	HPS4, biogenesis of lysosomal organelles complex 3 subunit 2 [Source:MGI Symbol;Acc:MGI:2177742]
5	protein_coding	1.13E+08	1.13E+08	ENSMUSG00000051503	MGI:3648511	Ccdc121rt3	coiled-coil domain containing 121, retrogene 3 [Source:MGI Symbol;Acc:MGI:3648511]
5	protein_coding	1.13E+08	1.13E+08	ENSMUSG00000029348	MGI:1920148	Asphd2	aspartate beta-hydroxylase domain containing 2 [Source:MGI Symbol;Acc:MGI:1920148]
5	protein_coding	1.13E+08	1.13E+08	ENSMUSG00000058153	MGI:1935121	Sez6l	seizure related 6 homolog like [Source:MGI Symbol;Acc:MGI:1935121]
5	protein_coding	1.13E+08	1.13E+08	ENSMUSG00000072722	MGI:3648937	Ccdc121rt2	coiled-coil domain containing 121, retrogene 2 [Source:MGI Symbol;Acc:MGI:3648937]
5	protein_coding	1.13E+08	1.13E+08	ENSMUSG00000072720	MGI:1921626	Myo18b	myosin XVIIIb [Source:MGI Symbol;Acc:MGI:1921626]
5	protein_coding	1.13E+08	1.13E+08	ENSMUSG00000042249	MGI:87941	Grk3	G protein-coupled receptor kinase 3 [Source:MGI Symbol;Acc:MGI:87941]
5	protein_coding	1.13E+08	1.13E+08	ENSMUSG00000042240	MGI:88519	Crybb2	crystallin, beta B2 [Source:MGI Symbol;Acc:MGI:88519]
5	protein_coding	1.13E+08	1.13E+08	ENSMUSG00000029352	MGI:102717	Crybb3	crystallin, beta B3 [Source:MGI Symbol;Acc:MGI:102717]

5	protein_coding	1.13E+08	1.13E+08	ENSMUSG00000051339	MGI:1920194	2900026A02Rik	RIKEN cDNA 2900026A02 gene [Source:MGI Symbol;Acc:MGI:1920194]
5	protein_coding	1.13E+08	1.13E+08	ENSMUSG00000066964	MGI:2685700	Lhfpl7	LHFPL tetraspan subfamily member 7 [Source:MGI Symbol;Acc:MGI:2685700]
5	protein_coding	1.13E+08	1.13E+08	ENSMUSG00000042216	MGI:107320	Sgsm1	small G protein signaling modulator 1 [Source:MGI Symbol;Acc:MGI:107320]
5	protein_coding	1.14E+08	1.14E+08	ENSMUSG00000063430	MGI:2445030	Wscd2	WSC domain containing 2 [Source:MGI Symbol;Acc:MGI:2445030]
5	protein_coding	1.14E+08	1.14E+08	ENSMUSG00000042190	MGI:109603	Cmklr1	chemerin chemokine-like receptor 1 [Source:MGI Symbol;Acc:MGI:109603]
5	protein_coding	1.14E+08	1.14E+08	ENSMUSG00000053334	MGI:1098550	Ficd	FIC domain containing [Source:MGI Symbol;Acc:MGI:1098550]
5	protein_coding	1.14E+08	1.14E+08	ENSMUSG00000018974	MGI:1858230	Sart3	[illegible] by T cells 3 [Source:MGI Symbol;Acc:MGI:1858230]
5	protein_coding	1.14E+08	1.14E+08	ENSMUSG00000025825	MGI:1913633	Iscu	iron-sulfur cluster assembly enzyme [Source:MGI Symbol;Acc:MGI:1913633]
5	protein_coding	1.14E+08	1.14E+08	ENSMUSG00000054675	MGI:2385228	Tmem119	transmembrane protein 119 [Source:MGI Symbol;Acc:MGI:2385228]
5	protein_coding	1.14E+08	1.14E+08	ENSMUSG00000048163	MGI:106689	Selplg	selectin, platelet (p-selectin) ligand [Source:MGI Symbol;Acc:MGI:106689]
5	protein_coding	1.14E+08	1.14E+08	ENSMUSG00000004530	MGI:1345964	Coro1c	coronin, actin binding protein 1C [Source:MGI Symbol;Acc:MGI:1345964]
5	protein_coding	1.14E+08	1.14E+08	ENSMUSG00000042121	MGI:2686240	Ssh1	slingshot protein phosphatase 1 [Source:MGI Symbol;Acc:MGI:2686240]
5	protein_coding	1.14E+08	1.14E+08	ENSMUSG00000042096	MGI:94859	Dao	D-amino acid oxidase [Source:MGI Symbol;Acc:MGI:94859]
5	protein_coding	1.14E+08	1.14E+08	ENSMUSG00000042078	MGI:1915916	Svop	SV2 related protein [Source:MGI Symbol;Acc:MGI:1915916]
5	protein_coding	1.14E+08	1.14E+08	ENSMUSG00000029592	MGI:2140991	Usp30	ubiquitin specific peptidase 30 [Source:MGI Symbol;Acc:MGI:2140991]
5	protein_coding	1.14E+08	1.14E+08	ENSMUSG00000044339	MGI:2141032	Alkbh2	alkB homolog 2, alpha-ketoglutarate-dependent dioxygenase [Source:MGI Symbol;Acc:MGI:2141032]
5	protein_coding	1.14E+08	1.14E+08	ENSMUSG00000029591	MGI:109352	Ung	uracil DNA glycosylase [Source:MGI Symbol;Acc:MGI:109352]

5	protein_coding	1.14E+08	1.14E+08	ENSMUSG00000042010	MGI:2140940	Acacb	acetyl-Coenzyme A carboxylase beta [Source:MGI Symbol;Acc:MGI:2140940]
5	protein_coding	1.14E+08	1.14E+08	ENSMUSG00000042002	MGI:2151057	Foxn4	forkhead box N4 [Source:MGI Symbol;Acc:MGI:2151057]
5	protein_coding	1.14E+08	1.15E+08	ENSMUSG00000066952	MGI:1914674	Myo1h	myosin 1H [Source:MGI Symbol;Acc:MGI:1914674]
5	protein_coding	1.15E+08	1.15E+08	ENSMUSG00000001098	MGI:2141207	Kctd10	potassium channel tetramerisation domain containing 10 [Source:MGI Symbol;Acc:MGI:2141207]
5	protein_coding	1.15E+08	1.15E+08	ENSMUSG00000029577	MGI:1891295	Ube3b	ubiquitin protein ligase E3B [Source:MGI Symbol;Acc:MGI:1891295]
5	protein_coding	1.15E+08	1.15E+08	ENSMUSG00000029575	MGI:1924947	Mmab	methylmalonic aciduria (cobalamin deficiency) cblB type homolog (human) [Source:MGI Symbol;Acc:MGI:1924947]
5	protein_coding	1.15E+08	1.15E+08	ENSMUSG00000041939	MGI:107624	Mvk	mevalonate kinase [Source:MGI Symbol;Acc:MGI:107624]
5	protein_coding	1.15E+08	1.15E+08	ENSMUSG00000041930	MGI:3605543	Fam222a	family with sequence similarity 222, member A [Source:MGI Symbol;Acc:MGI:3605543]
5	protein_coding	1.15E+08	1.15E+08	ENSMUSG00000014158	MGI:1926945	Trpv4	transient receptor potential cation channel, subfamily V, member 4 [Source:MGI Symbol;Acc:MGI:1926945]
5	protein_coding	1.15E+08	1.15E+08	ENSMUSG00000011884	MGI:1929253	Gltp	glycolipid transfer protein [Source:MGI Symbol;Acc:MGI:1929253]
5	protein_coding	1.15E+08	1.15E+08	ENSMUSG00000002486	MGI:1925082	Tchp	trichoplein, keratin filament binding [Source:MGI Symbol;Acc:MGI:1925082]
5	protein_coding	1.15E+08	1.15E+08	ENSMUSG00000041890	MGI:1347053	Git2	GIT ArfGAP 2 [Source:MGI Symbol;Acc:MGI:1347053]
5	protein_coding	1.15E+08	1.15E+08	ENSMUSG00000041870	MGI:1915670	Ankrd13a	ankyrin repeat domain 13a [Source:MGI Symbol;Acc:MGI:1915670]
5	protein_coding	1.15E+08	1.15E+08	ENSMUSG00000072694	MGI:7529997	Gm57857	predicted gene, 57857 [Source:MGI Symbol;Acc:MGI:7529997]
5	protein_coding	1.15E+08	1.15E+08	ENSMUSG00000029564	MGI:1914843	4930519G04Rik	RIKEN cDNA 4930519G04 gene [Source:MGI Symbol;Acc:MGI:1914843]
5	protein_coding	1.15E+08	1.15E+08	ENSMUSG00000029561	MGI:1344390	Oasl2	2'-5' oligoadenylate synthetase-like 2 [Source:MGI Symbol;Acc:MGI:1344390]

5	protein_coding	1.15E+08	1.15E+08	ENSMUSG00000041827	MGI:2180849	Oasl1	2'-5' oligoadenylate synthetase-like 1 [Source:MGI Symbol;Acc:MGI:2180849]
5	protein_coding	1.15E+08	1.15E+08	ENSMUSG00000029559	MGI:1919607	2210016L21Rik	RIKEN cDNA 2210016L21 gene [Source:MGI Symbol;Acc:MGI:1919607]
5	protein_coding	1.15E+08	1.15E+08	ENSMUSG00000029556	MGI:98504	Hnf1a	HNF1 homeobox A [Source:MGI Symbol;Acc:MGI:98504]
5	protein_coding	1.15E+08	1.15E+08	ENSMUSG00000029550	MGI:1891433	Sppl3	signal peptide peptidase 3 [Source:MGI Symbol;Acc:MGI:1891433]
5	protein_coding	1.15E+08	1.15E+08	ENSMUSG00000029545	MGI:87868	Acads	acyl-Coenzyme A dehydrogenase, short chain [Source:MGI Symbol;Acc:MGI:87868]
5	protein_coding	1.15E+08	1.15E+08	ENSMUSG00000046562	MGI:2147162	Unc119b	unc-119 lipid binding chaperone B [Source:MGI Symbol;Acc:MGI:2147162]
5	protein_coding	1.15E+08	1.15E+08	ENSMUSG00000048578	MGI:1924015	Mlec	malectin [Source:MGI Symbol;Acc:MGI:1924015]
5	protein_coding	1.15E+08	1.15E+08	ENSMUSG00000029544	MGI:1352750	Cabp1	calcium binding protein 1 [Source:MGI Symbol;Acc:MGI:1352750]
5	protein_coding	1.15E+08	1.15E+08	ENSMUSG00000060152	MGI:2151221	Pop5	processing of precursor 5, ribonuclease P/MRP family (S. cerevisiae) [Source:MGI Symbol;Acc:MGI:2151221]
5	protein_coding	1.15E+08	1.15E+08	ENSMUSG00000041740	MGI:1859162	Rnf10	ring finger protein 10 [Source:MGI Symbol;Acc:MGI:1859162]
5	protein_coding	1.15E+08	1.15E+08	ENSMUSG00000144239			novel protein
5	protein_coding	1.15E+08	1.15E+08	ENSMUSG00000041733	MGI:1098643	Coq5	coenzyme Q5 methyltransferase [Source:MGI Symbol;Acc:MGI:1098643]
5	protein_coding	1.15E+08	1.15E+08	ENSMUSG00000009013	MGI:1861457	Dynll1	dynein light chain LC8-type 1 [Source:MGI Symbol;Acc:MGI:1861457]
5	protein_coding	1.15E+08	1.15E+08	ENSMUSG00000029538	MGI:104896	Srsf9	serine and arginine-rich splicing factor 9 [Source:MGI Symbol;Acc:MGI:104896]
5	protein_coding	1.15E+08	1.15E+08	ENSMUSG00000029536	MGI:1923776	Gatc	glutamyl-tRNA amidotransferase subunit C [Source:MGI Symbol;Acc:MGI:1923776]
5	protein_coding	1.15E+08	1.15E+08	ENSMUSG00000029535	MGI:1916326	Triap1	TP53 regulated inhibitor of apoptosis 1 [Source:MGI Symbol;Acc:MGI:1916326]
5	protein_coding	1.15E+08	1.15E+08	ENSMUSG00000041697	MGI:103099	Cox6a1	cytochrome c oxidase subunit 6A1 [Source:MGI Symbol;Acc:MGI:103099]

5	protein_coding	1.16E+08	1.16E+08	ENSMUSG00000054256	MGI:107376	Msi1	musashi RNA-binding protein 1 [Source:MGI Symbol;Acc:MGI:107376]
5	protein_coding	1.16E+08	1.16E+08	ENSMUSG00000029522	MGI:101842	Pla2g1b	phospholipase A2, group IB, pancreas [Source:MGI Symbol;Acc:MGI:101842]
5	protein_coding	1.16E+08	1.16E+08	ENSMUSG00000029524	MGI:1922637	Sirt4	sirtuin 4 [Source:MGI Symbol;Acc:MGI:1922637]
5	protein_coding	1.16E+08	1.16E+08	ENSMUSG00000029528	MGI:108295	Pxn	paxillin [Source:MGI Symbol;Acc:MGI:108295]
5	protein_coding	1.16E+08	1.16E+08	ENSMUSG00000067274	MGI:1927636	Rplp0	ribosomal protein lateral stalk subunit P0 [Source:MGI Symbol;Acc:MGI:1927636]
5	protein_coding	1.16E+08	1.16E+08	ENSMUSG00000041638	MGI:2444248	Gcn1	GCN1 activator of EIF2AK4 [Source:MGI Symbol;Acc:MGI:2444248]
5	protein_coding	1.16E+08	1.16E+08	ENSMUSG00000029518	MGI:1924657	Rab35	RAB35, member RAS oncogene family [Source:MGI Symbol;Acc:MGI:1924657]
5	protein_coding	1.16E+08	1.16E+08	ENSMUSG00000041609	MGI:1922915	Bicdl1	BICD family like cargo adaptor 1 [Source:MGI Symbol;Acc:MGI:1922915]
5	protein_coding	1.16E+08	1.16E+08	ENSMUSG00000029516	MGI:105313	Cit	citron [Source:MGI Symbol;Acc:MGI:105313]
5	protein_coding	1.16E+08	1.16E+08	ENSMUSG00000029513	MGI:1336167	Prkab1	catalytic subunit [Source:MGI Symbol;Acc:MGI:1336167]
5	protein_coding	1.16E+08	1.16E+08	ENSMUSG00000079278	MGI:3651514	Tmem233	transmembrane protein 233 [Source:MGI Symbol;Acc:MGI:3651514]
5	protein_coding	1.16E+08	1.16E+08	ENSMUSG00000043913	MGI:2141043	Ccdc60	coiled-coil domain containing 60 [Source:MGI Symbol;Acc:MGI:2141043]
5	protein_coding	1.17E+08	1.17E+08	ENSMUSG00000041548	MGI:2135756	Hspb8	heat shock protein 8 [Source:MGI Symbol;Acc:MGI:2135756]
5	protein_coding	1.17E+08	1.17E+08	ENSMUSG00000063919	MGI:1916205	Srrm4	serine/arginine repetitive matrix 4 [Source:MGI Symbol;Acc:MGI:1916205]
5	protein_coding	1.17E+08	1.17E+08	ENSMUSG00000066900	MGI:1919204	Suds3	suppressor of defective silencing 3 homolog (S. cerevisiae) [Source:MGI Symbol;Acc:MGI:1919204]
5	protein_coding	1.17E+08	1.17E+08	ENSMUSG00000061288	MGI:3041177	Taok3	TAO kinase 3 [Source:MGI Symbol;Acc:MGI:3041177]
5	protein_coding	1.17E+08	1.17E+08	ENSMUSG00000032959	MGI:1344408	Pebp1	phosphatidylethanolamine binding protein 1 [Source:MGI Symbol;Acc:MGI:1344408]

5	protein_coding	1.17E+08	1.17E+08	ENSMUSG00000066894	MGI:2448533	Vsig10	V-set and immunoglobulin domain containing 10 [Source:MGI Symbol;Acc:MGI:2448533]
5	protein_coding	1.17E+08	1.18E+08	ENSMUSG00000029364	MGI:2144041	Wsb2	WD repeat and SOCS box-containing 2 [Source:MGI Symbol;Acc:MGI:2144041]
5	protein_coding	1.18E+08	1.18E+08	ENSMUSG00000029363	MGI:1919401	Rfc5	replication factor C (activator 1) 5 [Source:MGI Symbol;Acc:MGI:1919401]
5	protein_coding	1.18E+08	1.18E+08	ENSMUSG00000061578	MGI:3610315	Ksr2	kinase suppressor of ras 2 [Source:MGI Symbol;Acc:MGI:3610315]
5	protein_coding	1.18E+08	1.18E+08	ENSMUSG00000029361	MGI:97360	Nos1	nitric oxide synthase 1, neuronal [Source:MGI Symbol;Acc:MGI:97360]
5	protein_coding	1.18E+08	1.18E+08	ENSMUSG00000032898	MGI:1924223	Fbxo21	F-box protein 21 [Source:MGI Symbol;Acc:MGI:1924223]
5	protein_coding	1.18E+08	1.18E+08	ENSMUSG00000029359	MGI:1930803	Tesc	tescalcin [Source:MGI Symbol;Acc:MGI:1930803]
5	protein_coding	1.18E+08	1.18E+08	ENSMUSG00000032867	MGI:1923041	Fbxw8	F-box and WD-40 domain protein 8 [Source:MGI Symbol;Acc:MGI:1923041]
5	protein_coding	1.18E+08	1.18E+08	ENSMUSG00000046607	MGI:1201608	Hrk	harakiri, BCL2 interacting protein (contains only BH3 domain) [Source:MGI Symbol;Acc:MGI:1201608]
5	protein_coding	1.18E+08	1.18E+08	ENSMUSG00000032850	MGI:2442859	Rnft2	ring finger protein, transmembrane 2 [Source:MGI Symbol;Acc:MGI:2442859]
5	protein_coding	1.18E+08	1.18E+08	ENSMUSG00000032840	MGI:1924042	Spring1	SREBF pathway regulator in golgi 1 [Source:MGI Symbol;Acc:MGI:1924042]
5	protein_coding	1.19E+08	1.19E+08	ENSMUSG00000018076	MGI:2670178	Med13l	mediator complex subunit 13-like [Source:MGI Symbol;Acc:MGI:2670178]
5	protein_coding	1.20E+08	1.20E+08	ENSMUSG00000018604	MGI:98495	Tbx3	T-box 3 [Source:MGI Symbol;Acc:MGI:98495]
5	protein_coding	1.20E+08	1.20E+08	ENSMUSG00000018263	MGI:102541	Tbx5	T-box 5 [Source:MGI Symbol;Acc:MGI:102541]
5	protein_coding	1.20E+08	1.20E+08	ENSMUSG00000029594	MGI:1921361	Rbm19	RNA binding motif protein 19 [Source:MGI Symbol;Acc:MGI:1921361]
5	protein_coding	1.21E+08	1.21E+08	ENSMUSG00000029595	MGI:107792	Lhx5	LIM homeobox protein 5 [Source:MGI Symbol;Acc:MGI:107792]
5	protein_coding	1.21E+08	1.21E+08	ENSMUSG00000029596	MGI:2182607	Sdsl	serine dehydratase-like [Source:MGI Symbol;Acc:MGI:2182607]

5	protein_coding	1.21E+08	1.21E+08	ENSMUSG00000029597	MGI:98270	Sds	serine dehydratase [Source:MGI Symbol;Acc:MGI:98270]
5	protein_coding	1.21E+08	1.21E+08	ENSMUSG00000029598	MGI:1919022	Plbd2	phospholipase B domain containing 2 [Source:MGI Symbol;Acc:MGI:1919022]
5	protein_coding	1.21E+08	1.21E+08	ENSMUSG00000032754	MGI:2180781	Slc8b1	solute carrier family 8 (sodium/lithium/calcium exchanger), member B1 [Source:MGI Symbol;Acc:MGI:2180781]
5	protein_coding	1.21E+08	1.21E+08	ENSMUSG00000032741	MGI:2182472	Tpcn1	two pore channel 1 [Source:MGI Symbol;Acc:MGI:2182472]
5	protein_coding	1.21E+08	1.21E+08	ENSMUSG00000029601	MGI:1922982	Iqcd	IQ motif containing D [Source:MGI Symbol;Acc:MGI:1922982]
5	protein_coding	1.21E+08	1.21E+08	ENSMUSG00000029600	MGI:1922021	Rita1	RBPJ interacting and tubulin associated 1 [Source:MGI Symbol;Acc:MGI:1922021]
5	protein_coding	1.21E+08	1.21E+08	ENSMUSG00000029599	MGI:1919240	Ddx54	DEAD box helicase 54 [Source:MGI Symbol;Acc:MGI:1919240]
5	protein_coding	1.21E+08	1.21E+08	ENSMUSG00000094282	MGI:3779542	Cfap73	cilia and flagella associated protein 73 [Source:MGI Symbol;Acc:MGI:3779542]
5	protein_coding	1.21E+08	1.21E+08	ENSMUSG00000029602	MGI:1330842	Rasal1	RAS protein activator like 1 (GAP1 like) [Source:MGI Symbol;Acc:MGI:1330842]
5	protein_coding	1.21E+08	1.21E+08	ENSMUSG00000029603	MGI:1352744	Dtx1	deltex 1, E3 ubiquitin ligase [Source:MGI Symbol;Acc:MGI:1352744]
5	protein_coding	1.21E+08	1.21E+08	ENSMUSG00000032690	MGI:2180852	Oas2	2'-5' oligoadenylate synthetase 2 [Source:MGI Symbol;Acc:MGI:2180852]
5	protein_coding	1.21E+08	1.21E+08	ENSMUSG00000032661	MGI:2180850	Oas3	2'-5' oligoadenylate synthetase 3 [Source:MGI Symbol;Acc:MGI:2180850]
5	protein_coding	1.21E+08	1.21E+08	ENSMUSG00000066867	MGI:2180856	Oas1e	2'-5' oligoadenylate synthetase 1E [Source:MGI Symbol;Acc:MGI:2180856]
5	protein_coding	1.21E+08	1.21E+08	ENSMUSG00000001166	MGI:2149633	Oas1c	2'-5' oligoadenylate synthetase 1C [Source:MGI Symbol;Acc:MGI:2149633]
5	protein_coding	1.21E+08	1.21E+08	ENSMUSG00000029605	MGI:97430	Oas1b	2'-5' oligoadenylate synthetase 1B [Source:MGI Symbol;Acc:MGI:97430]
5	protein_coding	1.21E+08	1.21E+08	ENSMUSG00000053765	MGI:2180855	Oas1f	2'-5' oligoadenylate synthetase 1F [Source:MGI Symbol;Acc:MGI:2180855]
5	protein_coding	1.21E+08	1.21E+08	ENSMUSG00000001168	MGI:2180853	Oas1h	2'-5' oligoadenylate synthetase 1H [Source:MGI Symbol;Acc:MGI:2180853]

5	protein_coding	1.21E+08	1.21E+08	ENSMUSG00000066861	MGI:97429	Oas1g	2'-5' oligoadenylate synthetase 1G [Source:MGI Symbol;Acc:MGI:97429]
5	protein_coding	1.21E+08	1.21E+08	ENSMUSG00000052776	MGI:2180860	Oas1a	2'-5' oligoadenylate synthetase 1A [Source:MGI Symbol;Acc:MGI:2180860]
5	protein_coding	1.21E+08	1.21E+08	ENSMUSG00000032623	MGI:2140770	Oas1d	2'-5' oligoadenylate synthetase 1D [Source:MGI Symbol;Acc:MGI:2140770]
5	protein_coding	1.21E+08	1.21E+08	ENSMUSG00000029608	MGI:102788	Rph3a	rabphilin 3A [Source:MGI Symbol;Acc:MGI:102788]
5	protein_coding	1.21E+08	1.21E+08	ENSMUSG00000043733	MGI:99511	Ptpn11	protein tyrosine phosphatase, non-receptor type 11 [Source:MGI Symbol;Acc:MGI:99511]
5	protein_coding	1.21E+08	1.21E+08	ENSMUSG00000029614	MGI:108057	Rpl6	ribosomal protein L6 [Source:MGI Symbol;Acc:MGI:108057]
5	protein_coding	1.21E+08	1.22E+08	ENSMUSG00000042744	MGI:3647820	Hectd4	HECT domain E3 ubiquitin protein ligase 4 [Source:MGI Symbol;Acc:MGI:3647820]
5	protein_coding	1.22E+08	1.22E+08	ENSMUSG00000042726	MGI:1923551	Trafd1	TRAF type zinc finger domain containing 1 [Source:MGI Symbol;Acc:MGI:1923551]
5	protein_coding	1.22E+08	1.22E+08	ENSMUSG00000042719	MGI:2442563	Naa25	[illegible] subunit [Source:MGI Symbol;Acc:MGI:2442563]
5	protein_coding	1.22E+08	1.22E+08	ENSMUSG00000029616	MGI:1914647	Erp29	endoplasmic reticulum protein 29 [Source:MGI Symbol;Acc:MGI:1914647]
5	protein_coding	1.22E+08	1.22E+08	ENSMUSG00000029452	MGI:1924712	Tmem116	transmembrane protein 116 [Source:MGI Symbol;Acc:MGI:1924712]
5	protein_coding	1.22E+08	1.22E+08	ENSMUSG00000062438	MGI:2429506	Adam1b	a disintegrin and metallopeptidase domain 1b [Source:MGI Symbol;Acc:MGI:2429506]
5	protein_coding	1.22E+08	1.22E+08	ENSMUSG00000072647	MGI:2429504	Adam1a	a disintegrin and metallopeptidase domain 1a [Source:MGI Symbol;Acc:MGI:2429504]
5	protein_coding	1.22E+08	1.22E+08	ENSMUSG00000105340	MGI:5663015	Gm42878	predicted gene 42878 [Source:MGI Symbol;Acc:MGI:5663015]
5	protein_coding	1.22E+08	1.22E+08	ENSMUSG00000029454	MGI:1333110	Mapkapk5	MAP kinase-activated protein kinase 5 [Source:MGI Symbol;Acc:MGI:1333110]
5	protein_coding	1.22E+08	1.22E+08	ENSMUSG00000121598	MGI:7822511	Gm69851	predicted gene, 69851 [Source:MGI Symbol;Acc:MGI:7822511]
5	protein_coding	1.22E+08	1.22E+08	ENSMUSG00000029455	MGI:99600	Aldh2	aldehyde dehydrogenase 2, mitochondrial [Source:MGI Symbol;Acc:MGI:99600]
5	protein_coding	1.22E+08	1.22E+08	ENSMUSG00000042647	MGI:2443320	Acad12	[illegible] member 12 [Source:MGI Symbol;Acc:MGI:2443320]

5	protein_coding	1.22E+08	1.22E+08	ENSMUSG00000029456	MGI:1919235	Acad10	[illegible] member 10 [Source:MGI Symbol;Acc:MGI:1919235]
5	protein_coding	1.22E+08	1.22E+08	ENSMUSG00000029458	MGI:1919649	Brap	BRCA1 associated protein [Source:MGI Symbol;Acc:MGI:1919649]
5	protein_coding	1.22E+08	1.22E+08	ENSMUSG00000042605	MGI:1277223	Atxn2	ataxin 2 [Source:MGI Symbol;Acc:MGI:1277223]
5	protein_coding	1.22E+08	1.22E+08	ENSMUSG00000042594	MGI:893598	Sh2b3	SH2B adaptor protein 3 [Source:MGI Symbol;Acc:MGI:893598]
5	protein_coding	1.22E+08	1.22E+08	ENSMUSG00000044134	MGI:2442708	Pheta1	[illegible] adaptor 1 [Source:MGI Symbol;Acc:MGI:2442708]
5	protein_coding	1.22E+08	1.22E+08	ENSMUSG00000042589	MGI:107321	Cux2	cut-like homeobox 2 [Source:MGI Symbol;Acc:MGI:107321]
5	protein_coding	1.22E+08	1.22E+08	ENSMUSG00000013936	MGI:97272	Myl2	myosin, light polypeptide 2, regulatory, cardiac, slow [Source:MGI Symbol;Acc:MGI:97272]
5	protein_coding	1.22E+08	1.22E+08	ENSMUSG00000043036	MGI:3607777	Ccdc63	coiled-coil domain containing 63 [Source:MGI Symbol;Acc:MGI:3607777]
5	protein_coding	1.22E+08	1.22E+08	ENSMUSG00000004455	MGI:104872	Ppp1cc	protein phosphatase 1 catalytic subunit gamma [Source:MGI Symbol;Acc:MGI:104872]
5	protein_coding	1.22E+08	1.22E+08	ENSMUSG00000064267	MGI:1921346	Hvcn1	hydrogen voltage-gated channel 1 [Source:MGI Symbol;Acc:MGI:1921346]
5	protein_coding	1.22E+08	1.22E+08	ENSMUSG00000038593	MGI:3603820	Tctn1	tectonic family member 1 [Source:MGI Symbol;Acc:MGI:3603820]
5	protein_coding	1.22E+08	1.22E+08	ENSMUSG00000038582	MGI:2444593	Pptc7	PTC7 protein phosphatase homolog [Source:MGI Symbol;Acc:MGI:2444593]
5	protein_coding	1.22E+08	1.22E+08	ENSMUSG00000038569	MGI:2385231	Rad9b	RAD9 checkpoint clamp component B [Source:MGI Symbol;Acc:MGI:2385231]
5	protein_coding	1.22E+08	1.23E+08	ENSMUSG00000029462	MGI:1928344	Vps29	VPS29 retromer complex component [Source:MGI Symbol;Acc:MGI:1928344]
5	protein_coding	1.23E+08	1.23E+08	ENSMUSG00000029463	MGI:1916198	Fam216a	family with sequence similarity 216, member A [Source:MGI Symbol;Acc:MGI:1916198]
5	protein_coding	1.23E+08	1.23E+08	ENSMUSG00000029464	MGI:1289326	Gpn3	GPN-loop GTPase 3 [Source:MGI Symbol;Acc:MGI:1289326]
5	protein_coding	1.23E+08	1.23E+08	ENSMUSG00000029465	MGI:1928375	Arpc3	actin related protein 2/3 complex, subunit 3 [Source:MGI Symbol;Acc:MGI:1928375]
5	protein_coding	1.23E+08	1.23E+08	ENSMUSG00000029466	MGI:1929711	Anapc7	anaphase promoting complex subunit 7 [Source:MGI Symbol;Acc:MGI:1929711]

5	protein_coding	1.23E+08	1.23E+08	ENSMUSG00000029467	MGI:88110	Atp2a2	slow twitch 2 [Source:MGI Symbol;Acc:MGI:88110]
5	protein_coding	1.23E+08	1.23E+08	ENSMUSG00000029469	MGI:1098597	Ift81	intraflagellar transport 81 [Source:MGI Symbol;Acc:MGI:1098597]
5	protein_coding	1.23E+08	1.23E+08	ENSMUSG00000029468	MGI:1339957	P2rx7	channel, 7 [Source:MGI Symbol;Acc:MGI:1339957]
5	protein_coding	1.23E+08	1.23E+08	ENSMUSG00000029470	MGI:1338859	P2rx4	channel 4 [Source:MGI Symbol;Acc:MGI:1338859]
5	protein_coding	1.23E+08	1.23E+08	ENSMUSG00000029471	MGI:2444812	Camkk2	calcium/calmodulin-dependent protein kinase kinase 2, beta [Source:MGI Symbol;Acc:MGI:2444812]
5	protein_coding	1.23E+08	1.23E+08	ENSMUSG00000029472	MGI:1929722	Anapc5	anaphase-promoting complex subunit 5 [Source:MGI Symbol;Acc:MGI:1929722]
5	protein_coding	1.23E+08	1.23E+08	ENSMUSG00000029474	MGI:2153340	Rnf34	ring finger protein 34 [Source:MGI Symbol;Acc:MGI:2153340]
5	protein_coding	1.23E+08	1.23E+08	ENSMUSG00000029475	MGI:1354737	Kdm2b	lysine (K)-specific demethylase 2B [Source:MGI Symbol;Acc:MGI:1354737]
5	protein_coding	1.23E+08	1.23E+08	ENSMUSG00000049686	MGI:1925542	Orai1	modulator 1 [Source:MGI Symbol;Acc:MGI:1925542]
5	protein_coding	1.23E+08	1.23E+08	ENSMUSG00000029477	MGI:1922140	Morn3	MORN repeat containing 3 [Source:MGI Symbol;Acc:MGI:1922140]
5	protein_coding	1.23E+08	1.23E+08	ENSMUSG00000054434	MGI:3603158	Tmem120b	transmembrane protein 120B [Source:MGI Symbol;Acc:MGI:3603158]
5	protein_coding	1.23E+08	1.23E+08	ENSMUSG00000029449	MGI:1345629	Rhof	ras homolog family member F (in filopodia) [Source:MGI Symbol;Acc:MGI:1345629]
5	protein_coding	1.23E+08	1.23E+08	ENSMUSG00000038384	MGI:2652820	Setd1b	SET domain containing 1B [Source:MGI Symbol;Acc:MGI:2652820]
5	protein_coding	1.23E+08	1.23E+08	ENSMUSG00000029440	MGI:1914401	Psmd9	proteasome (prosome, macropain) 26S subunit, non-ATPase, 9 [Source:MGI Symbol;Acc:MGI:1914401]
5	protein_coding	1.23E+08	1.23E+08	ENSMUSG00000029445	MGI:96213	Hpd	4-hydroxyphenylpyruvic acid dioxygenase [Source:MGI Symbol;Acc:MGI:96213]
5	protein_coding	1.23E+08	1.23E+08	ENSMUSG00000029442	MGI:1918495	Cfap251	cilia and flagella associated protein 251 [Source:MGI Symbol;Acc:MGI:1918495]
5	protein_coding	1.23E+08	1.24E+08	ENSMUSG00000029438	MGI:1924295	Bcl7a	B cell CLL/lymphoma 7A [Source:MGI Symbol;Acc:MGI:1924295]

5	protein_coding	1.24E+08	1.24E+08	ENSMUSG00000038342	MGI:2141183	Mlxip	MLX interacting protein [Source:MGI Symbol;Acc:MGI:2141183]
5	protein_coding	1.24E+08	1.24E+08	ENSMUSG00000029437	MGI:1923649	Il31	interleukin 31 [Source:MGI Symbol;Acc:MGI:1923649]
5	protein_coding	1.24E+08	1.24E+08	ENSMUSG00000063409	MGI:2685907	Lrrc43	leucine rich repeat containing 43 [Source:MGI Symbol;Acc:MGI:2685907]
5	protein_coding	1.24E+08	1.24E+08	ENSMUSG00000029433	MGI:1913843	Diablo	diablo, IAP-binding mitochondrial protein [Source:MGI Symbol;Acc:MGI:1913843]
5	protein_coding	1.24E+08	1.24E+08	ENSMUSG00000029431	MGI:2680208	B3gnt4	UDP-GlcNAc:betaGal beta-1,3-N-acetylglucosaminyltransferase 4 [Source:MGI Symbol;Acc:MGI:2680208]
5	protein_coding	1.24E+08	1.24E+08	ENSMUSG00000114278	MGI:6118396	Gm49027	predicted gene, 49027 [Source:MGI Symbol;Acc:MGI:6118396]
5	protein_coding	1.24E+08	1.24E+08	ENSMUSG00000029434	MGI:1924823	Vps33a	VPS33A CORVET/HOPS core subunit [Source:MGI Symbol;Acc:MGI:1924823]
5	protein_coding	1.24E+08	1.24E+08	ENSMUSG00000049550	MGI:1928401	Clip1	CAP-GLY domain containing linker protein 1 [Source:MGI Symbol;Acc:MGI:1928401]
5	protein_coding	1.24E+08	1.24E+08	ENSMUSG00000029427	MGI:1917900	Zcchc8	zinc finger, CCHC domain containing 8 [Source:MGI Symbol;Acc:MGI:1917900]
5	protein_coding	1.24E+08	1.24E+08	ENSMUSG00000029422	MGI:1913489	Rsrc2	arginine/serine-rich coiled-coil 2 [Source:MGI Symbol;Acc:MGI:1913489]
5	protein_coding	1.24E+08	1.24E+08	ENSMUSG00000029414	MGI:2673709	Kntc1	kinetochore associated 1 [Source:MGI Symbol;Acc:MGI:2673709]
5	protein_coding	1.24E+08	1.24E+08	ENSMUSG00000045502	MGI:1933383	Hcar2	hydroxycarboxylic acid receptor 2 [Source:MGI Symbol;Acc:MGI:1933383]
5	protein_coding	1.24E+08	1.24E+08	ENSMUSG00000049241	MGI:2441671	Hcar1	hydrocarboxylic acid receptor 1 [Source:MGI Symbol;Acc:MGI:2441671]
5	protein_coding	1.24E+08	1.24E+08	ENSMUSG00000023106	MGI:1915434	Denr	density-regulated protein [Source:MGI Symbol;Acc:MGI:1915434]
5	protein_coding	1.24E+08	1.24E+08	ENSMUSG00000061882	MGI:2684996	Ccdc62	coiled-coil domain containing 62 [Source:MGI Symbol;Acc:MGI:2684996]
5	protein_coding	1.24E+08	1.24E+08	ENSMUSG00000000915	MGI:1352504	Hip1r	huntingtin interacting protein 1 related [Source:MGI Symbol;Acc:MGI:1352504]
5	protein_coding	1.24E+08	1.24E+08	ENSMUSG00000066278	MGI:1916724	Vps37b	vacuolar protein sorting 37B [Source:MGI Symbol;Acc:MGI:1916724]

5	protein_coding	1.24E+08	1.24E+08	ENSMUSG00000029408	MGI:1861729	Abcb9	ATP-binding cassette, sub-family B member 9 [Source:MGI Symbol;Acc:MGI:1861729]
5	protein_coding	1.24E+08	1.24E+08	ENSMUSG00000023707	MGI:1913877	Ogfod2	2-oxoglutarate and iron-dependent oxygenase domain containing 2 [Source:MGI Symbol;Acc:MGI:1913877]
5	protein_coding	1.24E+08	1.24E+08	ENSMUSG00000029404	MGI:1929500	Arl6ip4	protein 4 [Source:MGI Symbol;Acc:MGI:1929500]
5	protein_coding	1.24E+08	1.24E+08	ENSMUSG00000029406	MGI:1336192	Pitpnm2	phosphatidylinositol transfer protein, membrane-associated 2 [Source:MGI Symbol;Acc:MGI:1336192]
5	protein_coding	1.24E+08	1.24E+08	ENSMUSG00000038126	MGI:2443138	Mphosph9	M-phase phosphoprotein 9 [Source:MGI Symbol;Acc:MGI:2443138]
5	protein_coding	1.24E+08	1.24E+08	ENSMUSG00000047635	MGI:1919900	Mtrfr	rescue [Source:MGI Symbol;Acc:MGI:1919900]
5	protein_coding	1.24E+08	1.25E+08	ENSMUSG00000029394	MGI:1202069	Cdk2ap1	cyclin dependent kinase 2 associated protein 1 [Source:MGI Symbol;Acc:MGI:1202069]
5	protein_coding	1.25E+08	1.25E+08	ENSMUSG00000038095	MGI:2384298	Sbno1	strawberry notch 1 [Source:MGI Symbol;Acc:MGI:2384298]
5	protein_coding	1.25E+08	1.25E+08	ENSMUSG00000049327	MGI:1915206	Kmt5a	lysine methyltransferase 5A [Source:MGI Symbol;Acc:MGI:1915206]
5	protein_coding	1.25E+08	1.25E+08	ENSMUSG00000029401	MGI:1933112	Rilpl2	Rab interacting lysosomal protein-like 2 [Source:MGI Symbol;Acc:MGI:1933112]
5	protein_coding	1.25E+08	1.25E+08	ENSMUSG00000029402	MGI:1923417	Snrnp35	small nuclear ribonucleoprotein 35 (U11/U12) [Source:MGI Symbol;Acc:MGI:1923417]
5	protein_coding	1.25E+08	1.25E+08	ENSMUSG00000029392	MGI:1922945	Rilpl1	Rab interacting lysosomal protein-like 1 [Source:MGI Symbol;Acc:MGI:1922945]
5	protein_coding	1.25E+08	1.25E+08	ENSMUSG00000029390	MGI:1929269	Tmed2	transmembrane p24 trafficking protein 2 [Source:MGI Symbol;Acc:MGI:1929269]
5	protein_coding	1.25E+08	1.25E+08	ENSMUSG00000029389	MGI:1915098	Ddx55	DEAD box helicase 55 [Source:MGI Symbol;Acc:MGI:1915098]
5	protein_coding	1.25E+08	1.25E+08	ENSMUSG00000029388	MGI:2384802	Eif2b1	subunit alpha [Source:MGI Symbol;Acc:MGI:2384802]
5	protein_coding	1.25E+08	1.25E+08	ENSMUSG00000029387	MGI:1277143	Gtf2h3	general transcription factor IIH, polypeptide 3 [Source:MGI Symbol;Acc:MGI:1277143]
5	protein_coding	1.25E+08	1.25E+08	ENSMUSG00000118662	MGI:1915228	Tctn2	tectonic family member 2 [Source:MGI Symbol;Acc:MGI:1915228]

5	protein_coding	1.25E+08	1.25E+08	ENSMUSG00000038023	MGI:104855	Atp6v0a2	subunit A2 [Source:MGI Symbol;Acc:MGI:104855]
5	protein_coding	1.25E+08	1.25E+08	ENSMUSG00000038011	MGI:1860299	Dnah10	dynein, axonemal, heavy chain 10 [Source:MGI Symbol;Acc:MGI:1860299]
5	protein_coding	1.25E+08	1.25E+08	ENSMUSG00000037979	MGI:106485	Ccdc92	coiled-coil domain containing 92 [Source:MGI Symbol;Acc:MGI:106485]
5	protein_coding	1.25E+08	1.25E+08	ENSMUSG00000079215	MGI:2442505	Zfp664	zinc finger protein 664 [Source:MGI Symbol;Acc:MGI:2442505]
5	protein_coding	1.25E+08	1.25E+08	ENSMUSG00000037962	MGI:1920371	Rflna	refilin A [Source:MGI Symbol;Acc:MGI:1920371]
5	protein_coding	1.25E+08	1.25E+08	ENSMUSG00000029478	MGI:1337080	Ncor2	nuclear receptor co-repressor 2 [Source:MGI Symbol;Acc:MGI:1337080]
5	protein_coding	1.25E+08	1.25E+08	ENSMUSG00000037936	MGI:893578	Scarb1	scavenger receptor class B, member 1 [Source:MGI Symbol;Acc:MGI:893578]
5	protein_coding	1.25E+08	1.25E+08	ENSMUSG00000008348	MGI:98889	Ubc	ubiquitin C [Source:MGI Symbol;Acc:MGI:98889]
5	protein_coding	1.25E+08	1.26E+08	ENSMUSG00000029480	MGI:3028576	Dhx37	DEAH-box helicase 37 [Source:MGI Symbol;Acc:MGI:3028576]
5	protein_coding	1.26E+08	1.26E+08	ENSMUSG00000037905	MGI:1924059	Bri3bp	Bri3 binding protein [Source:MGI Symbol;Acc:MGI:1924059]
5	protein_coding	1.26E+08	1.26E+08	ENSMUSG00000029482	MGI:1926144	Aacs	acetoacetyl-CoA synthetase [Source:MGI Symbol;Acc:MGI:1926144]
5	protein_coding	1.26E+08	1.26E+08	ENSMUSG00000070498	MGI:3609245	Tmem132b	transmembrane protein 132B [Source:MGI Symbol;Acc:MGI:3609245]
5	rRNA	53300502	53300580	ENSMUSG00002076332	MGI:6848481	Gm56011	predicted gene, 56011 [Source:MGI Symbol;Acc:MGI:6848481]
5	rRNA	53503601	53503714	ENSMUSG00002076314	MGI:6848483	Gm56012	predicted gene, 56012 [Source:MGI Symbol;Acc:MGI:6848483]
5	rRNA	53575915	53576024	ENSMUSG00002075450	MGI:6848485	Gm56013	predicted gene, 56013 [Source:MGI Symbol;Acc:MGI:6848485]
5	rRNA	65293645	65293763	ENSMUSG00002076210	MGI:6848657	Gm56099	predicted gene, 56099 [Source:MGI Symbol;Acc:MGI:6848657]
5	rRNA	76166979	76167101	ENSMUSG00002075417	MGI:6847279	Gm55404	predicted gene, 55404 [Source:MGI Symbol;Acc:MGI:6847279]
5	rRNA	91041239	91041355	ENSMUSG00002075252	MGI:6848414	Gm55977	predicted gene, 55977 [Source:MGI Symbol;Acc:MGI:6848414]

5	rRNA	92266036	92266142	ENSMUSG00002075648	MGI:6848453	Gm55997	predicted gene, 55997 [Source:MGI Symbol;Acc:MGI:6848453]
5	rRNA	1.08E+08	1.08E+08	ENSMUSG00000064554	MGI:4422038	n-R5s173	nuclear encoded rRNA 5S 173 [Source:MGI Symbol;Acc:MGI:4422038]
5	rRNA	1.17E+08	1.17E+08	ENSMUSG00002075044	MGI:6848505	Gm56023	predicted gene, 56023 [Source:MGI Symbol;Acc:MGI:6848505]
5	rRNA	1.19E+08	1.19E+08	ENSMUSG00002076829	MGI:6848615	Gm56078	predicted gene, 56078 [Source:MGI Symbol;Acc:MGI:6848615]
5	rRNA	1.20E+08	1.20E+08	ENSMUSG00002075687	MGI:6848651	Gm56096	predicted gene, 56096 [Source:MGI Symbol;Acc:MGI:6848651]
5	scaRNA	92203923	92204051	ENSMUSG00000088712	MGI:5452505	Gm22728	predicted gene, 22728 [Source:MGI Symbol;Acc:MGI:5452505]
5	scaRNA	92577644	92577787	ENSMUSG00002076272	MGI:6848523	Gm56032	predicted gene, 56032 [Source:MGI Symbol;Acc:MGI:6848523]
5	scaRNA	1.24E+08	1.24E+08	ENSMUSG00002076894	MGI:6847105	Gm55317	predicted gene, 55317 [Source:MGI Symbol;Acc:MGI:6847105]
5	snoRNA	53116693	53116804	ENSMUSG00000077757	MGI:5453309	Gm23532	predicted gene, 23532 [Source:MGI Symbol;Acc:MGI:5453309]
5	snoRNA	54984557	54984685	ENSMUSG00000077507	MGI:5454012	Gm24235	predicted gene, 24235 [Source:MGI Symbol;Acc:MGI:5454012]
5	snoRNA	56893432	56893561	ENSMUSG00000088734	MGI:5453673	Gm23896	predicted gene, 23896 [Source:MGI Symbol;Acc:MGI:5453673]
5	snoRNA	57268166	57268288	ENSMUSG00000088751	MGI:5454679	Gm24902	predicted gene, 24902 [Source:MGI Symbol;Acc:MGI:5454679]
5	snoRNA	57285987	57286114	ENSMUSG00000077333	MGI:5452792	Gm23015	predicted gene, 23015 [Source:MGI Symbol;Acc:MGI:5452792]
5	snoRNA	58571571	58571704	ENSMUSG00000088796	MGI:5452313	Gm22536	predicted gene, 22536 [Source:MGI Symbol;Acc:MGI:5452313]
5	snoRNA	60874660	60874782	ENSMUSG00000077555	MGI:5453485	Gm23708	predicted gene, 23708 [Source:MGI Symbol;Acc:MGI:5453485]
5	snoRNA	62289696	62289827	ENSMUSG00000077228	MGI:5452050	Gm22273	predicted gene, 22273 [Source:MGI Symbol;Acc:MGI:5452050]
5	snoRNA	62680751	62680882	ENSMUSG00000119858	MGI:5453856	Gm24079	predicted gene, 24079 [Source:MGI Symbol;Acc:MGI:5453856]
5	snoRNA	64159696	64159827	ENSMUSG00000077209	MGI:5455385	Gm25608	predicted gene, 25608 [Source:MGI Symbol;Acc:MGI:5455385]

5	snoRNA	64301591	64301724	ENSMUSG00000087954	MGI:5452336	Gm22559	predicted gene, 22559 [Source:MGI Symbol;Acc:MGI:5452336]
5	snoRNA	65934357	65934550	ENSMUSG00002076604	MGI:6848714	Gm56128	predicted gene, 56128 [Source:MGI Symbol;Acc:MGI:6848714]
5	snoRNA	68723513	68723639	ENSMUSG00000087880	MGI:5453382	Gm23605	predicted gene, 23605 [Source:MGI Symbol;Acc:MGI:5453382]
5	snoRNA	74254191	74254311	ENSMUSG00000093355	MGI:3819495	Snora26	small nucleolar RNA, H/ACA box 26 [Source:MGI Symbol;Acc:MGI:3819495]
5	snoRNA	77817571	77817693	ENSMUSG00000089415	MGI:5451788	Gm22011	predicted gene, 22011 [Source:MGI Symbol;Acc:MGI:5451788]
5	snoRNA	78927706	78927841	ENSMUSG00000077178	MGI:5455533	Gm25756	predicted gene, 25756 [Source:MGI Symbol;Acc:MGI:5455533]
5	snoRNA	83796670	83796801	ENSMUSG00000077288	MGI:5455542	Gm25765	predicted gene, 25765 [Source:MGI Symbol;Acc:MGI:5455542]
5	snoRNA	83871309	83871436	ENSMUSG00000077474	MGI:5454569	Gm24792	predicted gene, 24792 [Source:MGI Symbol;Acc:MGI:5454569]
5	snoRNA	87881387	87881509	ENSMUSG00000119930	MGI:5454843	Gm25066	predicted gene, 25066 [Source:MGI Symbol;Acc:MGI:5454843]
5	snoRNA	87912538	87912660	ENSMUSG00000119270	MGI:5454091	Gm24314	predicted gene, 24314 [Source:MGI Symbol;Acc:MGI:5454091]
5	snoRNA	89976730	89976858	ENSMUSG00000077175	MGI:5455535	Gm25758	predicted gene, 25758 [Source:MGI Symbol;Acc:MGI:5455535]
5	snoRNA	92341181	92341324	ENSMUSG00000084745	MGI:5455067	Gm25290	predicted gene, 25290 [Source:MGI Symbol;Acc:MGI:5455067]
5	snoRNA	93221720	93221855	ENSMUSG00000088240	MGI:5452692	Gm22915	predicted gene, 22915 [Source:MGI Symbol;Acc:MGI:5452692]
5	snoRNA	98689343	98689473	ENSMUSG00000077424	MGI:5454056	Gm24279	predicted gene, 24279 [Source:MGI Symbol;Acc:MGI:5454056]
5	snoRNA	99803560	99803691	ENSMUSG00002076040	MGI:6848697	Gm56119	predicted gene, 56119 [Source:MGI Symbol;Acc:MGI:6848697]
5	snoRNA	1.00E+08	1.00E+08	ENSMUSG00002076587	MGI:6848701	Gm56121	predicted gene, 56121 [Source:MGI Symbol;Acc:MGI:6848701]
5	snoRNA	1.00E+08	1.00E+08	ENSMUSG00002076905	MGI:6848730	Gm56136	predicted gene, 56136 [Source:MGI Symbol;Acc:MGI:6848730]
5	snoRNA	1.01E+08	1.01E+08	ENSMUSG00000077354	MGI:5456063	Gm26286	predicted gene, 26286 [Source:MGI Symbol;Acc:MGI:5456063]

5	snoRNA	1.08E+08	1.08E+08	ENSMUSG00000064453	MGI:102855	Snord21	small nucleolar RNA, C/D box 21 [Source:MGI Symbol;Acc:MGI:102855]
5	snoRNA	1.08E+08	1.08E+08	ENSMUSG00000077222	MGI:5452047	Gm22270	predicted gene, 22270 [Source:MGI Symbol;Acc:MGI:5452047]
5	snoRNA	1.08E+08	1.08E+08	ENSMUSG00000077426	MGI:5456164	Gm26387	predicted gene, 26387 [Source:MGI Symbol;Acc:MGI:5456164]
5	snoRNA	1.10E+08	1.10E+08	ENSMUSG00000087722	MGI:5456054	Gm26277	predicted gene, 26277 [Source:MGI Symbol;Acc:MGI:5456054]
5	snoRNA	1.11E+08	1.11E+08	ENSMUSG00000077254	MGI:5455856	Gm26079	predicted gene, 26079 [Source:MGI Symbol;Acc:MGI:5455856]
5	snoRNA	1.18E+08	1.18E+08	ENSMUSG00000119733	MGI:5454853	Gm25076	predicted gene, 25076 [Source:MGI Symbol;Acc:MGI:5454853]
5	snoRNA	1.21E+08	1.21E+08	ENSMUSG00000064841	MGI:5455982	Gm26205	predicted gene, 26205 [Source:MGI Symbol;Acc:MGI:5455982]
5	snRNA	40563093	40563272	ENSMUSG00000089393	MGI:5452799	Gm23022	predicted gene, 23022 [Source:MGI Symbol;Acc:MGI:5452799]
5	snRNA	50447722	50447828	ENSMUSG00000119176	MGI:5452395	Gm22618	predicted gene, 22618 [Source:MGI Symbol;Acc:MGI:5452395]
5	snRNA	52500460	52500564	ENSMUSG00002075892	MGI:6848430	Gm55985	predicted gene, 55985 [Source:MGI Symbol;Acc:MGI:6848430]
5	snRNA	55460762	55460923	ENSMUSG00000065795	MGI:5455290	Gm25513	predicted gene, 25513 [Source:MGI Symbol;Acc:MGI:5455290]
5	snRNA	59372289	59372395	ENSMUSG00000119859	MGI:5452085	Gm22308	predicted gene, 22308 [Source:MGI Symbol;Acc:MGI:5452085]
5	snRNA	60658176	60658283	ENSMUSG00000088048	MGI:5453108	Gm23331	predicted gene, 23331 [Source:MGI Symbol;Acc:MGI:5453108]
5	snRNA	65818149	65818222	ENSMUSG00000088494	MGI:5454176	Gm24399	predicted gene, 24399 [Source:MGI Symbol;Acc:MGI:5454176]
5	snRNA	66321899	66322005	ENSMUSG00000093258	MGI:5452706	Gm22929	predicted gene, 22929 [Source:MGI Symbol;Acc:MGI:5452706]
5	snRNA	69543409	69543515	ENSMUSG00000119086	MGI:5453709	Gm23932	predicted gene, 23932 [Source:MGI Symbol;Acc:MGI:5453709]
5	snRNA	69909746	69909871	ENSMUSG00000080566	MGI:5455821	Gm26044	predicted gene, 26044 [Source:MGI Symbol;Acc:MGI:5455821]
5	snRNA	70076438	70076524	ENSMUSG00000084579	MGI:5453438	Gm23661	predicted gene, 23661 [Source:MGI Symbol;Acc:MGI:5453438]

5	snRNA	70344128	70344189	ENSMUSG00000088863	MGI:5452844	Gm23067	predicted gene, 23067 [Source:MGI Symbol;Acc:MGI:5452844]
5	snRNA	70708184	70708290	ENSMUSG00000118789	MGI:5455849	Gm26072	predicted gene, 26072 [Source:MGI Symbol;Acc:MGI:5455849]
5	snRNA	72462708	72462805	ENSMUSG00000089195	MGI:5455545	Gm25768	predicted gene, 25768 [Source:MGI Symbol;Acc:MGI:5455545]
5	snRNA	72576056	72576162	ENSMUSG00000119356	MGI:5453489	Gm23712	predicted gene, 23712 [Source:MGI Symbol;Acc:MGI:5453489]
5	snRNA	73128522	73128607	ENSMUSG00002076468	MGI:6846538	Gm55032	predicted gene, 55032 [Source:MGI Symbol;Acc:MGI:6846538]
5	snRNA	75532702	75532806	ENSMUSG00002076047	MGI:6847201	Gm55365	predicted gene, 55365 [Source:MGI Symbol;Acc:MGI:6847201]
5	snRNA	75721706	75721810	ENSMUSG00000089078	MGI:5454279	Gm24502	predicted gene, 24502 [Source:MGI Symbol;Acc:MGI:5454279]
5	snRNA	79898228	79898376	ENSMUSG00000084435	MGI:5453206	Gm23429	predicted gene, 23429 [Source:MGI Symbol;Acc:MGI:5453206]
5	snRNA	80246755	80246861	ENSMUSG00000119601	MGI:5454081	Gm24304	predicted gene, 24304 [Source:MGI Symbol;Acc:MGI:5454081]
5	snRNA	81041501	81041603	ENSMUSG00000065235	MGI:5452751	Gm22974	predicted gene, 22974 [Source:MGI Symbol;Acc:MGI:5452751]
5	snRNA	82951612	82951708	ENSMUSG00000089162	MGI:5453906	Gm24129	predicted gene, 24129 [Source:MGI Symbol;Acc:MGI:5453906]
5	snRNA	83837851	83837972	ENSMUSG00000084731	MGI:5455583	Gm25806	predicted gene, 25806 [Source:MGI Symbol;Acc:MGI:5455583]
5	snRNA	85977486	85977646	ENSMUSG00000064555	MGI:5454403	Gm24626	predicted gene, 24626 [Source:MGI Symbol;Acc:MGI:5454403]
5	snRNA	86227625	86227794	ENSMUSG00000064859	MGI:5454301	Gm24524	predicted gene, 24524 [Source:MGI Symbol;Acc:MGI:5454301]
5	snRNA	86384714	86384820	ENSMUSG00000119790	MGI:5455250	Gm25473	predicted gene, 25473 [Source:MGI Symbol;Acc:MGI:5455250]
5	snRNA	87064075	87064181	ENSMUSG00000119825	MGI:5454988	Gm25211	predicted gene, 25211 [Source:MGI Symbol;Acc:MGI:5454988]
5	snRNA	87076762	87076867	ENSMUSG00000075983	MGI:5453898	Gm24121	predicted gene, 24121 [Source:MGI Symbol;Acc:MGI:5453898]
5	snRNA	91365757	91365863	ENSMUSG00000119278	MGI:5452869	Gm23092	predicted gene, 23092 [Source:MGI Symbol;Acc:MGI:5452869]

5	snRNA	92450033	92450151	ENSMUSG00000088623	MGI:5452808	Gm23031	predicted gene, 23031 [Source:MGI Symbol;Acc:MGI:5452808]
5	snRNA	93160891	93160998	ENSMUSG00000089018	MGI:5455298	Gm25521	predicted gene, 25521 [Source:MGI Symbol;Acc:MGI:5455298]
5	snRNA	96015193	96015299	ENSMUSG00000119041	MGI:5455714	Gm25937	predicted gene, 25937 [Source:MGI Symbol;Acc:MGI:5455714]
5	snRNA	97055491	97055684	ENSMUSG00000064550	MGI:5454400	Gm24623	predicted gene, 24623 [Source:MGI Symbol;Acc:MGI:5454400]
5	snRNA	1.00E+08	1.00E+08	ENSMUSG00002076497	MGI:6848703	Gm56122	predicted gene, 56122 [Source:MGI Symbol;Acc:MGI:6848703]
5	snRNA	1.01E+08	1.01E+08	ENSMUSG00002076815	MGI:6849022	Gm56282	predicted gene, 56282 [Source:MGI Symbol;Acc:MGI:6849022]
5	snRNA	1.04E+08	1.04E+08	ENSMUSG00000088121	MGI:5455498	Gm25721	predicted gene, 25721 [Source:MGI Symbol;Acc:MGI:5455498]
5	snRNA	1.04E+08	1.04E+08	ENSMUSG00000119515	MGI:5454674	Gm24897	predicted gene, 24897 [Source:MGI Symbol;Acc:MGI:5454674]
5	snRNA	1.05E+08	1.05E+08	ENSMUSG00000119653	MGI:5453874	Gm24097	predicted gene, 24097 [Source:MGI Symbol;Acc:MGI:5453874]
5	snRNA	1.06E+08	1.06E+08	ENSMUSG00000119467	MGI:5455449	Gm25672	predicted gene, 25672 [Source:MGI Symbol;Acc:MGI:5455449]
5	snRNA	1.07E+08	1.07E+08	ENSMUSG00002076219	MGI:6846582	Gm55054	predicted gene, 55054 [Source:MGI Symbol;Acc:MGI:6846582]
5	snRNA	1.07E+08	1.07E+08	ENSMUSG00000088429	MGI:5452334	Gm22557	predicted gene, 22557 [Source:MGI Symbol;Acc:MGI:5452334]
5	snRNA	1.11E+08	1.11E+08	ENSMUSG00000119880	MGI:5452493	Gm22716	predicted gene, 22716 [Source:MGI Symbol;Acc:MGI:5452493]
5	snRNA	1.11E+08	1.11E+08	ENSMUSG00000118827	MGI:5452360	Gm22583	predicted gene, 22583 [Source:MGI Symbol;Acc:MGI:5452360]
5	snRNA	1.13E+08	1.13E+08	ENSMUSG00000065373	MGI:5452517	Gm22740	predicted gene, 22740 [Source:MGI Symbol;Acc:MGI:5452517]
5	snRNA	1.14E+08	1.14E+08	ENSMUSG00000065320	MGI:5451833	Gm22056	predicted gene, 22056 [Source:MGI Symbol;Acc:MGI:5451833]
5	snRNA	1.16E+08	1.16E+08	ENSMUSG00000119520	MGI:5454042	Gm24265	predicted gene, 24265 [Source:MGI Symbol;Acc:MGI:5454042]
5	snRNA	1.16E+08	1.16E+08	ENSMUSG00000119132	MGI:5454184	Gm24407	predicted gene, 24407 [Source:MGI Symbol;Acc:MGI:5454184]

5	snRNA	1.18E+08	1.18E+08	ENSMUSG00002075698	MGI:6848573	Gm56057	predicted gene, 56057 [Source:MGI Symbol;Acc:MGI:6848573]
5	snRNA	1.18E+08	1.18E+08	ENSMUSG00000088293	MGI:5456188	Gm26411	predicted gene, 26411 [Source:MGI Symbol;Acc:MGI:5456188]
5	snRNA	1.21E+08	1.21E+08	ENSMUSG00000077347	MGI:5454448	Gm24671	predicted gene, 24671 [Source:MGI Symbol;Acc:MGI:5454448]
5	snRNA	1.23E+08	1.23E+08	ENSMUSG00000088583	MGI:5452742	Gm22965	predicted gene, 22965 [Source:MGI Symbol;Acc:MGI:5452742]
5	snRNA	1.23E+08	1.23E+08	ENSMUSG00000065191	MGI:5453235	Gm23458	predicted gene, 23458 [Source:MGI Symbol;Acc:MGI:5453235]
5	snRNA	1.24E+08	1.24E+08	ENSMUSG00000084552	MGI:5454617	Gm24840	predicted gene, 24840 [Source:MGI Symbol;Acc:MGI:5454617]
5	snRNA	1.25E+08	1.25E+08	ENSMUSG00002074914	MGI:6847769	Gm55651	predicted gene, 55651 [Source:MGI Symbol;Acc:MGI:6847769]
5	TEC	41438579	41440243	ENSMUSG00000106865	MGI:1921963	4930519E07Rik	RIKEN cDNA 4930519E07 gene [Source:MGI Symbol;Acc:MGI:1921963]
5	TEC	43216598	43218226	ENSMUSG00000105416	MGI:5662690	Gm42553	predicted gene 42553 [Source:MGI Symbol;Acc:MGI:5662690]
5	TEC	43301174	43305670	ENSMUSG00000105087	MGI:5662689	Gm42552	predicted gene 42552 [Source:MGI Symbol;Acc:MGI:5662689]
5	TEC	43405528	43407100	ENSMUSG00000105374	MGI:5662688	Gm42551	predicted gene 42551 [Source:MGI Symbol;Acc:MGI:5662688]
5	TEC	43422500	43423711	ENSMUSG00000105326	MGI:1924319	6030400A10Rik	RIKEN cDNA 6030400A10 gene [Source:MGI Symbol;Acc:MGI:1924319]
5	TEC	43685969	43690236	ENSMUSG00000104547	MGI:5663159	Gm43022	predicted gene 43022 [Source:MGI Symbol;Acc:MGI:5663159]
5	TEC	43733163	43735268	ENSMUSG00000104912	MGI:5663160	Gm43023	predicted gene 43023 [Source:MGI Symbol;Acc:MGI:5663160]
5	TEC	43910251	43911613	ENSMUSG00000104728	MGI:5662599	Gm42462	predicted gene 42462 [Source:MGI Symbol;Acc:MGI:5662599]
5	TEC	43939585	43942112	ENSMUSG00000104823	MGI:5663319	Gm43182	predicted gene 43182 [Source:MGI Symbol;Acc:MGI:5663319]
5	TEC	43941388	43943459	ENSMUSG00000106609	MGI:5663318	Gm43181	predicted gene 43181 [Source:MGI Symbol;Acc:MGI:5663318]
5	TEC	44105458	44106004	ENSMUSG00000106573	MGI:5663320	Gm43183	predicted gene 43183 [Source:MGI Symbol;Acc:MGI:5663320]

5	TEC	44105462	44106003	ENSMUSG00000105831	MGI:5663321	Gm43184	predicted gene 43184 [Source:MGI Symbol;Acc:MGI:5663321]
5	TEC	44263191	44264881	ENSMUSG00000105048	MGI:5663103	Gm42966	predicted gene 42966 [Source:MGI Symbol;Acc:MGI:5663103]
5	TEC	44291735	44292292	ENSMUSG00000105373	MGI:5662566	Gm42429	predicted gene 42429 [Source:MGI Symbol;Acc:MGI:5662566]
5	TEC	44295322	44300613	ENSMUSG00000105263	MGI:5662564	Gm42427	predicted gene 42427 [Source:MGI Symbol;Acc:MGI:5662564]
5	TEC	44371143	44374601	ENSMUSG00000105519	MGI:2444377	E430021H15Rik	RIKEN cDNA E430021H15 gene [Source:MGI Symbol;Acc:MGI:2444377]
5	TEC	44381852	44382666	ENSMUSG00000105149	MGI:5663120	Gm42983	predicted gene 42983 [Source:MGI Symbol;Acc:MGI:5663120]
5	TEC	44513047	44513389	ENSMUSG00000104541	MGI:5663251	Gm43114	predicted gene 43114 [Source:MGI Symbol;Acc:MGI:5663251]
5	TEC	44649512	44652763	ENSMUSG00000105282	MGI:5663118	Gm42981	predicted gene 42981 [Source:MGI Symbol;Acc:MGI:5663118]
5	TEC	44671000	44671902	ENSMUSG00000105136	MGI:1924798	8030487O14Rik	RIKEN cDNA 8030487O14 gene [Source:MGI Symbol;Acc:MGI:1924798]
5	TEC	44674303	44675055	ENSMUSG00000106332	MGI:5663643	Gm43506	predicted gene 43506 [Source:MGI Symbol;Acc:MGI:5663643]
5	TEC	44742405	44742543	ENSMUSG00000105648	MGI:5663642	Gm43505	predicted gene 43505 [Source:MGI Symbol;Acc:MGI:5663642]
5	TEC	44781261	44785043	ENSMUSG00000105861	MGI:5663645	Gm43508	predicted gene 43508 [Source:MGI Symbol;Acc:MGI:5663645]
5	TEC	44822036	44824073	ENSMUSG00000104990	MGI:5663641	Gm43504	predicted gene 43504 [Source:MGI Symbol;Acc:MGI:5663641]
5	TEC	44837536	44840943	ENSMUSG00000105347	MGI:5663640	Gm43503	predicted gene 43503 [Source:MGI Symbol;Acc:MGI:5663640]
5	TEC	44893679	44895895	ENSMUSG00000104851	MGI:2444788	E030026E10Rik	RIKEN cDNA E030026E10 gene [Source:MGI Symbol;Acc:MGI:2444788]
5	TEC	44908506	44911099	ENSMUSG00000106603	MGI:5663646	Gm43509	predicted gene 43509 [Source:MGI Symbol;Acc:MGI:5663646]
5	TEC	45400994	45404649	ENSMUSG00000105247	MGI:5662656	Gm42519	predicted gene 42519 [Source:MGI Symbol;Acc:MGI:5662656]
5	TEC	45688414	45690453	ENSMUSG00000105745	MGI:5662658	Gm42521	predicted gene 42521 [Source:MGI Symbol;Acc:MGI:5662658]

5	TEC	45714022	45715523	ENSMUSG00000104837	MGI:5662657	Gm42520	predicted gene 42520 [Source:MGI Symbol;Acc:MGI:5662657]
5	TEC	45817409	45820063	ENSMUSG00000104665	MGI:5663503	Gm43366	predicted gene 43366 [Source:MGI Symbol;Acc:MGI:5663503]
5	TEC	45826044	45827050	ENSMUSG00000104986	MGI:1917038	1600023N17Rik	RIKEN cDNA 1600023N17 gene [Source:MGI Symbol;Acc:MGI:1917038]
5	TEC	45860075	45862102	ENSMUSG00000104342	MGI:5595560	Gm36401	predicted gene, 36401 [Source:MGI Symbol;Acc:MGI:5595560]
5	TEC	45915617	45919032	ENSMUSG00000104852	MGI:5663338	Gm43201	predicted gene 43201 [Source:MGI Symbol;Acc:MGI:5663338]
5	TEC	45927362	45929522	ENSMUSG00000105457	MGI:5663337	Gm43200	predicted gene 43200 [Source:MGI Symbol;Acc:MGI:5663337]
5	TEC	46014984	46016106	ENSMUSG00000105459	MGI:1921205	4930449I04Rik	RIKEN cDNA 4930449I04 gene [Source:MGI Symbol;Acc:MGI:1921205]
5	TEC	46659471	46660683	ENSMUSG00000106875	MGI:5663230	Gm43093	predicted gene 43093 [Source:MGI Symbol;Acc:MGI:5663230]
5	TEC	46952022	46953875	ENSMUSG00000107113	MGI:5663229	Gm43092	predicted gene 43092 [Source:MGI Symbol;Acc:MGI:5663229]
5	TEC	47310993	47311937	ENSMUSG00000104660	MGI:5663738	Gm43601	predicted gene 43601 [Source:MGI Symbol;Acc:MGI:5663738]
5	TEC	48215393	48217989	ENSMUSG00000106214	MGI:5663563	Gm43426	predicted gene 43426 [Source:MGI Symbol;Acc:MGI:5663563]
5	TEC	48284426	48286038	ENSMUSG00000105502	MGI:5662673	Gm42536	predicted gene 42536 [Source:MGI Symbol;Acc:MGI:5662673]
5	TEC	48369014	48371587	ENSMUSG00000105184	MGI:5662674	Gm42537	predicted gene 42537 [Source:MGI Symbol;Acc:MGI:5662674]
5	TEC	48437009	48438762	ENSMUSG00000105086	MGI:5662671	Gm42534	predicted gene 42534 [Source:MGI Symbol;Acc:MGI:5662671]
5	TEC	48481954	48483352	ENSMUSG00000106604	MGI:5662672	Gm42535	predicted gene 42535 [Source:MGI Symbol;Acc:MGI:5662672]
5	TEC	48692686	48695283	ENSMUSG00000105653	MGI:5663968	Gm43831	predicted gene 43831 [Source:MGI Symbol;Acc:MGI:5663968]
5	TEC	48782011	48784495	ENSMUSG00000105574	MGI:5663785	Gm43648	predicted gene 43648 [Source:MGI Symbol;Acc:MGI:5663785]
5	TEC	48869432	48873233	ENSMUSG00000105530	MGI:5663966	Gm43829	predicted gene 43829 [Source:MGI Symbol;Acc:MGI:5663966]

5	TEC	48948242	48949308	ENSMUSG00000105454	MGI:5663967	Gm43830	predicted gene 43830 [Source:MGI Symbol;Acc:MGI:5663967]
5	TEC	48956352	48958621	ENSMUSG00000105958	MGI:5663456	Gm43319	predicted gene 43319 [Source:MGI Symbol;Acc:MGI:5663456]
5	TEC	48960070	48961353	ENSMUSG00000106404	MGI:5663056	Gm42919	predicted gene 42919 [Source:MGI Symbol;Acc:MGI:5663056]
5	TEC	48976818	48979226	ENSMUSG00000106408	MGI:5663458	Gm43321	predicted gene 43321 [Source:MGI Symbol;Acc:MGI:5663458]
5	TEC	49000762	49003677	ENSMUSG00000106414	MGI:5663457	Gm43320	predicted gene 43320 [Source:MGI Symbol;Acc:MGI:5663457]
5	TEC	49082296	49086082	ENSMUSG00000103544	MGI:3644560	Gm10048	predicted gene 10048 [Source:MGI Symbol;Acc:MGI:3644560]
5	TEC	49105363	49107311	ENSMUSG00000105974	MGI:5663455	Gm43318	predicted gene 43318 [Source:MGI Symbol;Acc:MGI:5663455]
5	TEC	49135620	49136927	ENSMUSG00000105791	MGI:5663478	Gm43341	predicted gene 43341 [Source:MGI Symbol;Acc:MGI:5663478]
5	TEC	49155054	49155881	ENSMUSG00000106469	MGI:1924665	C030015E24Rik	RIKEN cDNA C030015E24 gene [Source:MGI Symbol;Acc:MGI:1924665]
5	TEC	49162754	49165297	ENSMUSG00000104655	MGI:5663454	Gm43317	predicted gene 43317 [Source:MGI Symbol;Acc:MGI:5663454]
5	TEC	49174855	49176149	ENSMUSG00000105332	MGI:5663453	Gm43316	predicted gene 43316 [Source:MGI Symbol;Acc:MGI:5663453]
5	TEC	49178349	49179680	ENSMUSG00000104929	MGI:5662903	Gm42766	predicted gene 42766 [Source:MGI Symbol;Acc:MGI:5662903]
5	TEC	49226352	49228802	ENSMUSG00000106244	MGI:5663183	Gm43046	predicted gene 43046 [Source:MGI Symbol;Acc:MGI:5663183]
5	TEC	49227345	49230804	ENSMUSG00000105620	MGI:5663184	Gm43047	predicted gene 43047 [Source:MGI Symbol;Acc:MGI:5663184]
5	TEC	49231618	49232249	ENSMUSG00000104562	MGI:5662904	Gm42767	predicted gene 42767 [Source:MGI Symbol;Acc:MGI:5662904]
5	TEC	49241216	49244112	ENSMUSG00000104661	MGI:5662905	Gm42768	predicted gene 42768 [Source:MGI Symbol;Acc:MGI:5662905]
5	TEC	49273101	49274759	ENSMUSG00000105922	MGI:5662906	Gm42769	predicted gene 42769 [Source:MGI Symbol;Acc:MGI:5662906]
5	TEC	49281929	49286083	ENSMUSG00000105983	MGI:5662907	Gm42770	predicted gene 42770 [Source:MGI Symbol;Acc:MGI:5662907]

5	TEC	49374643	49375030	ENSMUSG00000105828	MGI:5663185	Gm43048	predicted gene 43048 [Source:MGI Symbol;Acc:MGI:5663185]
5	TEC	49377018	49379503	ENSMUSG00000106579	MGI:5662908	Gm42771	predicted gene 42771 [Source:MGI Symbol;Acc:MGI:5662908]
5	TEC	49566134	49569174	ENSMUSG00000105700	MGI:5662909	Gm42772	predicted gene 42772 [Source:MGI Symbol;Acc:MGI:5662909]
5	TEC	49582359	49584125	ENSMUSG00000106539	MGI:5663807	Gm43670	predicted gene 43670 [Source:MGI Symbol;Acc:MGI:5663807]
5	TEC	49673167	49676279	ENSMUSG00000105771	MGI:1920226	2900064K03Rik	RIKEN cDNA 2900064K03 gene [Source:MGI Symbol;Acc:MGI:1920226]
5	TEC	50100751	50101900	ENSMUSG00000104634	MGI:5662598	Gm42461	predicted gene 42461 [Source:MGI Symbol;Acc:MGI:5662598]
5	TEC	50216438	50219004	ENSMUSG00000106430	MGI:5662597	Gm42460	predicted gene 42460 [Source:MGI Symbol;Acc:MGI:5662597]
5	TEC	50995659	50998101	ENSMUSG00000105931	MGI:5663151	Gm43014	predicted gene 43014 [Source:MGI Symbol;Acc:MGI:5663151]
5	TEC	51640094	51641647	ENSMUSG00000105442	MGI:5662751	Gm42614	predicted gene 42614 [Source:MGI Symbol;Acc:MGI:5662751]
5	TEC	51648439	51650258	ENSMUSG00000105622	MGI:5662752	Gm42615	predicted gene 42615 [Source:MGI Symbol;Acc:MGI:5662752]
5	TEC	51656870	51658800	ENSMUSG00000106575	MGI:5662750	Gm42613	predicted gene 42613 [Source:MGI Symbol;Acc:MGI:5662750]
5	TEC	51668210	51671635	ENSMUSG00000105762	MGI:5663742	Gm43605	predicted gene 43605 [Source:MGI Symbol;Acc:MGI:5663742]
5	TEC	52012562	52016117	ENSMUSG00000105663	MGI:5663743	Gm43606	predicted gene 43606 [Source:MGI Symbol;Acc:MGI:5663743]
5	TEC	52032761	52036944	ENSMUSG00000106010	MGI:5662753	Gm42616	predicted gene 42616 [Source:MGI Symbol;Acc:MGI:5662753]
5	TEC	52049328	52050556	ENSMUSG00000104919	MGI:5662754	Gm42617	predicted gene 42617 [Source:MGI Symbol;Acc:MGI:5662754]
5	TEC	52064464	52068273	ENSMUSG00000105942	MGI:5663312	Gm43175	predicted gene 43175 [Source:MGI Symbol;Acc:MGI:5663312]
5	TEC	52237230	52239674	ENSMUSG00000106292	MGI:5663311	Gm43174	predicted gene 43174 [Source:MGI Symbol;Acc:MGI:5663311]
5	TEC	52296927	52299915	ENSMUSG00000106648	MGI:5663313	Gm43176	predicted gene 43176 [Source:MGI Symbol;Acc:MGI:5663313]

5	TEC	52331712	52333097	ENSMUSG00000106607	MGI:5663315	Gm43178	predicted gene 43178 [Source:MGI Symbol;Acc:MGI:5663315]
5	TEC	52396678	52399563	ENSMUSG00000105328	MGI:5663317	Gm43180	predicted gene 43180 [Source:MGI Symbol;Acc:MGI:5663317]
5	TEC	52700248	52702686	ENSMUSG00000104974	MGI:5663823	Gm43686	predicted gene 43686 [Source:MGI Symbol;Acc:MGI:5663823]
5	TEC	54062506	54066560	ENSMUSG00000107182	MGI:5663405	Gm43268	predicted gene 43268 [Source:MGI Symbol;Acc:MGI:5663405]
5	TEC	54075786	54077556	ENSMUSG00000107173	MGI:5663403	Gm43266	predicted gene 43266 [Source:MGI Symbol;Acc:MGI:5663403]
5	TEC	54964676	54967356	ENSMUSG00000106126	MGI:5663918	Gm43781	predicted gene 43781 [Source:MGI Symbol;Acc:MGI:5663918]
5	TEC	55660871	55664470	ENSMUSG00000105702	MGI:5663917	Gm43780	predicted gene 43780 [Source:MGI Symbol;Acc:MGI:5663917]
5	TEC	55810291	55810489	ENSMUSG00000105186	MGI:5663915	Gm43778	predicted gene 43778 [Source:MGI Symbol;Acc:MGI:5663915]
5	TEC	55946513	55950229	ENSMUSG00000105678	MGI:5663842	Gm43705	predicted gene 43705 [Source:MGI Symbol;Acc:MGI:5663842]
5	TEC	57608968	57611619	ENSMUSG00000105693	MGI:5663843	Gm43706	predicted gene 43706 [Source:MGI Symbol;Acc:MGI:5663843]
5	TEC	57775186	57775340	ENSMUSG00000104924	MGI:5663139	Gm43002	predicted gene 43002 [Source:MGI Symbol;Acc:MGI:5663139]
5	TEC	57853339	57853719	ENSMUSG00000106517	MGI:5662771	Gm42634	predicted gene 42634 [Source:MGI Symbol;Acc:MGI:5662771]
5	TEC	57881735	57884305	ENSMUSG00000105940	MGI:5662772	Gm42635	predicted gene 42635 [Source:MGI Symbol;Acc:MGI:5662772]
5	TEC	57936215	57938392	ENSMUSG00000104821	MGI:5662618	Gm42481	predicted gene 42481 [Source:MGI Symbol;Acc:MGI:5662618]
5	TEC	57950501	57953095	ENSMUSG00000105637	MGI:5662617	Gm42480	predicted gene 42480 [Source:MGI Symbol;Acc:MGI:5662617]
5	TEC	57955845	57957791	ENSMUSG00000105050	MGI:5662619	Gm42482	predicted gene 42482 [Source:MGI Symbol;Acc:MGI:5662619]
5	TEC	58061981	58064695	ENSMUSG00000105079	MGI:5663852	Gm43715	predicted gene 43715 [Source:MGI Symbol;Acc:MGI:5663852]
5	TEC	58082962	58084317	ENSMUSG00000105877	MGI:5662616	Gm42479	predicted gene 42479 [Source:MGI Symbol;Acc:MGI:5662616]

5	TEC	58087092	58088558	ENSMUSG00000106337	MGI:5662615	Gm42478	predicted gene 42478 [Source:MGI Symbol;Acc:MGI:5662615]
5	TEC	58102384	58104254	ENSMUSG00000104871	MGI:5662776	Gm42639	predicted gene 42639 [Source:MGI Symbol;Acc:MGI:5662776]
5	TEC	58118936	58120130	ENSMUSG00000105431	MGI:5662777	Gm42640	predicted gene 42640 [Source:MGI Symbol;Acc:MGI:5662777]
5	TEC	58122756	58124859	ENSMUSG00000104709	MGI:5662621	Gm42484	predicted gene 42484 [Source:MGI Symbol;Acc:MGI:5662621]
5	TEC	58141554	58142794	ENSMUSG00000103959	MGI:5610948	Gm37720	predicted gene, 37720 [Source:MGI Symbol;Acc:MGI:5610948]
5	TEC	58204673	58206414	ENSMUSG00000106646	MGI:5662620	Gm42483	predicted gene 42483 [Source:MGI Symbol;Acc:MGI:5662620]
5	TEC	58278781	58282266	ENSMUSG00000105049	MGI:5662623	Gm42486	predicted gene 42486 [Source:MGI Symbol;Acc:MGI:5662623]
5	TEC	58330777	58332871	ENSMUSG00000106146	MGI:5663182	Gm43045	predicted gene 43045 [Source:MGI Symbol;Acc:MGI:5663182]
5	TEC	59236781	59239020	ENSMUSG00000106467	MGI:5663532	Gm43395	predicted gene 43395 [Source:MGI Symbol;Acc:MGI:5663532]
5	TEC	60110854	60113483	ENSMUSG00000104563	MGI:5663178	Gm43041	predicted gene 43041 [Source:MGI Symbol;Acc:MGI:5663178]
5	TEC	60154284	60156904	ENSMUSG00000105158	MGI:5662999	Gm42862	predicted gene 42862 [Source:MGI Symbol;Acc:MGI:5662999]
5	TEC	60961059	60965049	ENSMUSG00000106044	MGI:5662997	Gm42860	predicted gene 42860 [Source:MGI Symbol;Acc:MGI:5662997]
5	TEC	62586062	62586420	ENSMUSG00000105285	MGI:5663375	Gm43238	predicted gene 43238 [Source:MGI Symbol;Acc:MGI:5663375]
5	TEC	62769758	62770801	ENSMUSG00000105694	MGI:5663374	Gm43237	predicted gene 43237 [Source:MGI Symbol;Acc:MGI:5663374]
5	TEC	62814142	62817450	ENSMUSG00000105707	MGI:5663890	Gm43753	predicted gene 43753 [Source:MGI Symbol;Acc:MGI:5663890]
5	TEC	63803513	63808237	ENSMUSG00000104868	MGI:3642120	Nwd2os	NACHT and WD repeat domain containing 2, opposite strand [Source:MGI Symbol;Acc:MGI:3642120]
5	TEC	64391273	64393585	ENSMUSG00000104947	MGI:5663858	Gm43721	predicted gene 43721 [Source:MGI Symbol;Acc:MGI:5663858]

5	TEC	64625400	64626353	ENSMUSG00000105251	MGI:1922486	4930526M16Rik	RIKEN cDNA 4930526M16 gene [Source:MGI Symbol;Acc:MGI:1922486]
5	TEC	64715526	64715906	ENSMUSG00000106566	MGI:5663973	Gm43836	predicted gene 43836 [Source:MGI Symbol;Acc:MGI:5663973]
5	TEC	64721114	64721262	ENSMUSG00000104961	MGI:5663972	Gm43835	predicted gene 43835 [Source:MGI Symbol;Acc:MGI:5663972]
5	TEC	64721520	64722162	ENSMUSG00000104806	MGI:5662703	Gm42566	predicted gene 42566 [Source:MGI Symbol;Acc:MGI:5662703]
5	TEC	64961357	64963078	ENSMUSG00000106205	MGI:3041194	C230096K16Rik	RIKEN cDNA C230096K16 gene [Source:MGI Symbol;Acc:MGI:3041194]
5	TEC	65129993	65132291	ENSMUSG00000106186	MGI:5663764	Gm43627	predicted gene 43627 [Source:MGI Symbol;Acc:MGI:5663764]
5	TEC	65465151	65466337	ENSMUSG00000105935	MGI:5663765	Gm43628	predicted gene 43628 [Source:MGI Symbol;Acc:MGI:5663765]
5	TEC	65563094	65563860	ENSMUSG00000106291	MGI:1915726	1110003F10Rik	RIKEN cDNA 1110003F10 gene [Source:MGI Symbol;Acc:MGI:1915726]
5	TEC	65567994	65570370	ENSMUSG00000105896	MGI:5663448	Gm43311	predicted gene 43311 [Source:MGI Symbol;Acc:MGI:5663448]
5	TEC	65587449	65588923	ENSMUSG00000104917	MGI:5663426	Gm43289	predicted gene 43289 [Source:MGI Symbol;Acc:MGI:5663426]
5	TEC	65633848	65635404	ENSMUSG00000104641	MGI:5663427	Gm43290	predicted gene 43290 [Source:MGI Symbol;Acc:MGI:5663427]
5	TEC	65650439	65651358	ENSMUSG00000107195	MGI:1925464	4930589O11Rik	RIKEN cDNA 4930589O11 gene [Source:MGI Symbol;Acc:MGI:1925464]
5	TEC	65688911	65693103	ENSMUSG00000105601	MGI:5662862	Gm42725	predicted gene 42725 [Source:MGI Symbol;Acc:MGI:5662862]
5	TEC	65699272	65701023	ENSMUSG00000107362	MGI:5623194	Gm40309	predicted gene, 40309 [Source:MGI Symbol;Acc:MGI:5623194]
5	TEC	65774292	65777114	ENSMUSG00000106767	MGI:5662864	Gm42727	predicted gene 42727 [Source:MGI Symbol;Acc:MGI:5662864]
5	TEC	65781277	65784206	ENSMUSG00000106696	MGI:5662866	Gm42729	predicted gene 42729 [Source:MGI Symbol;Acc:MGI:5662866]
5	TEC	65829952	65831503	ENSMUSG00000107158	MGI:5662865	Gm42728	predicted gene 42728 [Source:MGI Symbol;Acc:MGI:5662865]
5	TEC	65837357	65839687	ENSMUSG00000107050	MGI:2441875	C030017G13Rik	RIKEN cDNA C030017G13 gene [Source:MGI Symbol;Acc:MGI:2441875]

5	TEC	65935357	65937861	ENSMUSG00000106682	MGI:5662785	Gm42648	predicted gene 42648 [Source:MGI Symbol;Acc:MGI:5662785]
5	TEC	66010675	66012301	ENSMUSG00000106713	MGI:5662782	Gm42645	predicted gene 42645 [Source:MGI Symbol;Acc:MGI:5662782]
5	TEC	66014661	66015574	ENSMUSG00000106776	MGI:5662783	Gm42646	predicted gene 42646 [Source:MGI Symbol;Acc:MGI:5662783]
5	TEC	66016660	66017362	ENSMUSG00000106681	MGI:5663141	Gm43004	predicted gene 43004 [Source:MGI Symbol;Acc:MGI:5663141]
5	TEC	66068967	66073289	ENSMUSG00000107254	MGI:5663906	Gm43769	predicted gene 43769 [Source:MGI Symbol;Acc:MGI:5663906]
5	TEC	66108648	66109548	ENSMUSG00000106768	MGI:1922236	4930480C01Rik	RIKEN cDNA 4930480C01 gene [Source:MGI Symbol;Acc:MGI:1922236]
5	TEC	66121194	66122771	ENSMUSG00000107049	MGI:5663908	Gm43771	predicted gene 43771 [Source:MGI Symbol;Acc:MGI:5663908]
5	TEC	66226398	66230238	ENSMUSG00000107304	MGI:5663912	Gm43775	predicted gene 43775 [Source:MGI Symbol;Acc:MGI:5663912]
5	TEC	66237552	66241809	ENSMUSG00000107390	MGI:5663460	Gm43323	predicted gene 43323 [Source:MGI Symbol;Acc:MGI:5663460]
5	TEC	66372090	66375024	ENSMUSG00000107125	MGI:3781994	Gm3822	predicted gene 3822 [Source:MGI Symbol;Acc:MGI:3781994]
5	TEC	66478348	66480360	ENSMUSG00000106818	MGI:5663927	Gm43790	predicted gene 43790 [Source:MGI Symbol;Acc:MGI:5663927]
5	TEC	66503236	66504568	ENSMUSG00000107155	MGI:5663930	Gm43793	predicted gene 43793 [Source:MGI Symbol;Acc:MGI:5663930]
5	TEC	66587260	66589469	ENSMUSG00000106732	MGI:5663929	Gm43792	predicted gene 43792 [Source:MGI Symbol;Acc:MGI:5663929]
5	TEC	66601075	66601766	ENSMUSG00000106772	MGI:5663931	Gm43794	predicted gene 43794 [Source:MGI Symbol;Acc:MGI:5663931]
5	TEC	66681530	66683455	ENSMUSG00000107091	MGI:5663480	Gm43343	predicted gene 43343 [Source:MGI Symbol;Acc:MGI:5663480]
5	TEC	66689139	66691504	ENSMUSG00000106720	MGI:5663932	Gm43795	predicted gene 43795 [Source:MGI Symbol;Acc:MGI:5663932]
5	TEC	66749805	66752913	ENSMUSG00000106706	MGI:2444587	C530043K16Rik	RIKEN cDNA C530043K16 gene [Source:MGI Symbol;Acc:MGI:2444587]
5	TEC	66846225	66848736	ENSMUSG00000107300	MGI:5663416	Gm43279	predicted gene 43279 [Source:MGI Symbol;Acc:MGI:5663416]

5	TEC	66875713	66877262	ENSMUSG00000107284	MGI:5663417	Gm43280	predicted gene 43280 [Source:MGI Symbol;Acc:MGI:5663417]
5	TEC	67073351	67076054	ENSMUSG00000106749	MGI:5663418	Gm43281	predicted gene 43281 [Source:MGI Symbol;Acc:MGI:5663418]
5	TEC	67126772	67128928	ENSMUSG00000107290	MGI:5663419	Gm43282	predicted gene 43282 [Source:MGI Symbol;Acc:MGI:5663419]
5	TEC	67195775	67196719	ENSMUSG00000106785	MGI:5662850	Gm42713	predicted gene 42713 [Source:MGI Symbol;Acc:MGI:5662850]
5	TEC	67284846	67287488	ENSMUSG00000106995	MGI:5592326	Gm33167	predicted gene, 33167 [Source:MGI Symbol;Acc:MGI:5592326]
5	TEC	67585336	67587746	ENSMUSG00000106832	MGI:5662769	Gm42632	predicted gene 42632 [Source:MGI Symbol;Acc:MGI:5662769]
5	TEC	67764561	67765169	ENSMUSG00000106947	MGI:1916650	1700025A08Rik	RIKEN cDNA 1700025A08 gene [Source:MGI Symbol;Acc:MGI:1916650]
5	TEC	67791006	67792651	ENSMUSG00000107041	MGI:5662872	Gm42735	predicted gene 42735 [Source:MGI Symbol;Acc:MGI:5662872]
5	TEC	67914919	67916673	ENSMUSG00000106859	MGI:5662874	Gm42737	predicted gene 42737 [Source:MGI Symbol;Acc:MGI:5662874]
5	TEC	67990693	67994171	ENSMUSG00000106837	MGI:2443017	D630030B08Rik	RIKEN cDNA D630030B08 gene [Source:MGI Symbol;Acc:MGI:2443017]
5	TEC	68000463	68001763	ENSMUSG00000107136	MGI:5662603	Gm42466	predicted gene 42466 [Source:MGI Symbol;Acc:MGI:5662603]
5	TEC	68030225	68031577	ENSMUSG00000107206	MGI:5662873	Gm42736	predicted gene 42736 [Source:MGI Symbol;Acc:MGI:5662873]
5	TEC	68138014	68141704	ENSMUSG00000106897	MGI:5663164	Gm43027	predicted gene 43027 [Source:MGI Symbol;Acc:MGI:5663164]
5	TEC	68239832	68241717	ENSMUSG00000106747	MGI:5663162	Gm43025	predicted gene 43025 [Source:MGI Symbol;Acc:MGI:5663162]
5	TEC	69114751	69117732	ENSMUSG00000107117	MGI:5663979	Gm43842	predicted gene 43842 [Source:MGI Symbol;Acc:MGI:5663979]
5	TEC	69326015	69327591	ENSMUSG00000107208	MGI:2140783	D130004A15Rik	RIKEN cDNA D130004A15 gene [Source:MGI Symbol;Acc:MGI:2140783]
5	TEC	69338426	69339456	ENSMUSG00000107234	MGI:5663508	Gm43371	predicted gene 43371 [Source:MGI Symbol;Acc:MGI:5663508]
5	TEC	69374764	69377633	ENSMUSG00000107363	MGI:5663510	Gm43373	predicted gene 43373 [Source:MGI Symbol;Acc:MGI:5663510]

5	TEC	69379188	69381578	ENSMUSG00000106673	MGI:5663511	Gm43374	predicted gene 43374 [Source:MGI Symbol;Acc:MGI:5663511]
5	TEC	69420928	69423056	ENSMUSG00000106753	MGI:5663367	Gm43230	predicted gene 43230 [Source:MGI Symbol;Acc:MGI:5663367]
5	TEC	69433064	69434913	ENSMUSG00000107312	MGI:5663366	Gm43229	predicted gene 43229 [Source:MGI Symbol;Acc:MGI:5663366]
5	TEC	69469050	69473410	ENSMUSG00000107148	MGI:5663025	Gm42888	predicted gene 42888 [Source:MGI Symbol;Acc:MGI:5663025]
5	TEC	69661185	69662303	ENSMUSG00000106986	MGI:5663024	Gm42887	predicted gene 42887 [Source:MGI Symbol;Acc:MGI:5663024]
5	TEC	69713900	69714225	ENSMUSG00000107291	MGI:1920391	3110031N09Rik	RIKEN cDNA 3110031N09 gene [Source:MGI Symbol;Acc:MGI:1920391]
5	TEC	70718608	70721446	ENSMUSG00000106549	MGI:5662790	Gm42653	predicted gene 42653 [Source:MGI Symbol;Acc:MGI:5662790]
5	TEC	72711583	72712572	ENSMUSG00000105510	MGI:5663952	Gm43815	predicted gene 43815 [Source:MGI Symbol;Acc:MGI:5663952]
5	TEC	72967311	72969417	ENSMUSG00000106739	MGI:5663636	Gm43499	predicted gene 43499 [Source:MGI Symbol;Acc:MGI:5663636]
5	TEC	73069930	73072285	ENSMUSG00000052154	MGI:3708720	Gm9870	predicted gene 9870 [Source:MGI Symbol;Acc:MGI:3708720]
5	TEC	73172542	73175028	ENSMUSG00000107373	MGI:5662805	Gm42668	predicted gene 42668 [Source:MGI Symbol;Acc:MGI:5662805]
5	TEC	73199120	73202497	ENSMUSG00000107171	MGI:5662709	Gm42572	predicted gene 42572 [Source:MGI Symbol;Acc:MGI:5662709]
5	TEC	73345297	73347760	ENSMUSG00000107109	MGI:5662708	Gm42571	predicted gene 42571 [Source:MGI Symbol;Acc:MGI:5662708]
5	TEC	73430215	73431092	ENSMUSG00000106999	MGI:5662871	Gm42734	predicted gene 42734 [Source:MGI Symbol;Acc:MGI:5662871]
5	TEC	73465402	73465920	ENSMUSG00000107292	MGI:5662870	Gm42733	predicted gene 42733 [Source:MGI Symbol;Acc:MGI:5662870]
5	TEC	73468707	73469903	ENSMUSG00000107331	MGI:5662869	Gm42732	predicted gene 42732 [Source:MGI Symbol;Acc:MGI:5662869]
5	TEC	73616672	73617065	ENSMUSG00000106675	MGI:5663935	Gm43798	predicted gene 43798 [Source:MGI Symbol;Acc:MGI:5663935]
5	TEC	73804356	73804934	ENSMUSG00000106773	MGI:5663936	Gm43799	predicted gene 43799 [Source:MGI Symbol;Acc:MGI:5663936]

5	TEC	74208960	74214166	ENSMUSG00000106993	MGI:5663554	Gm43417	predicted gene 43417 [Source:MGI Symbol;Acc:MGI:5663554]
5	TEC	74220699	74221448	ENSMUSG00000106973	MGI:5663552	Gm43415	predicted gene 43415 [Source:MGI Symbol;Acc:MGI:5663552]
5	TEC	74252798	74253812	ENSMUSG00000107255	MGI:5663553	Gm43416	predicted gene 43416 [Source:MGI Symbol;Acc:MGI:5663553]
5	TEC	74332822	74336711	ENSMUSG00000106735	MGI:2442263	A330058E17Rik	RIKEN cDNA A330058E17 gene [Source:MGI Symbol;Acc:MGI:2442263]
5	TEC	74409809	74410277	ENSMUSG00000107209	MGI:5662736	Gm42599	predicted gene 42599 [Source:MGI Symbol;Acc:MGI:5662736]
5	TEC	74527824	74530576	ENSMUSG00000107009	MGI:1925146	6720475M21Rik	RIKEN cDNA 6720475M21 gene [Source:MGI Symbol;Acc:MGI:1925146]
5	TEC	74590161	74591766	ENSMUSG00000106992	MGI:5663304	Gm43167	predicted gene 43167 [Source:MGI Symbol;Acc:MGI:5663304]
5	TEC	74736608	74737937	ENSMUSG00000107165	MGI:5663884	Gm43747	predicted gene 43747 [Source:MGI Symbol;Acc:MGI:5663884]
5	TEC	75146517	75149727	ENSMUSG00000107306	MGI:5662714	Gm42577	predicted gene 42577 [Source:MGI Symbol;Acc:MGI:5662714]
5	TEC	75180897	75183742	ENSMUSG00000107205	MGI:5662713	Gm42576	predicted gene 42576 [Source:MGI Symbol;Acc:MGI:5662713]
5	TEC	75315546	75315996	ENSMUSG00000107245	MGI:5662940	Gm42803	predicted gene 42803 [Source:MGI Symbol;Acc:MGI:5662940]
5	TEC	75898542	75900865	ENSMUSG00000107251	MGI:5663239	Gm43102	predicted gene 43102 [Source:MGI Symbol;Acc:MGI:5663239]
5	TEC	76006749	76008870	ENSMUSG00000103684	MGI:5611408	Gm38180	predicted gene, 38180 [Source:MGI Symbol;Acc:MGI:5611408]
5	TEC	76445447	76449622	ENSMUSG00000106815	MGI:5663795	Gm43658	predicted gene 43658 [Source:MGI Symbol;Acc:MGI:5663795]
5	TEC	76803776	76806202	ENSMUSG00000103529	MGI:2443970	A730089K16Rik	RIKEN cDNA A730089K16 gene [Source:MGI Symbol;Acc:MGI:2443970]
5	TEC	81397096	81400537	ENSMUSG00000107226	MGI:5663731	Gm43594	predicted gene 43594 [Source:MGI Symbol;Acc:MGI:5663731]
5	TEC	81683573	81684519	ENSMUSG00000107375	MGI:5663221	Gm43084	predicted gene 43084 [Source:MGI Symbol;Acc:MGI:5663221]
5	TEC	81974663	81975670	ENSMUSG00000106746	MGI:1920293	2900064F13Rik	RIKEN cDNA 2900064F13 gene [Source:MGI Symbol;Acc:MGI:1920293]

5	TEC	82267526	82269209	ENSMUSG00000106860	MGI:1913575	1700008H02Rik	RIKEN cDNA 1700008H02 gene [Source:MGI Symbol;Acc:MGI:1913575]
5	TEC	87503694	87504780	ENSMUSG00000105192	MGI:5662933	Gm42796	predicted gene 42796 [Source:MGI Symbol;Acc:MGI:5662933]
5	TEC	89670719	89672859	ENSMUSG00000104715	MGI:5663652	Gm43515	predicted gene 43515 [Source:MGI Symbol;Acc:MGI:5663652]
5	TEC	90545132	90547778	ENSMUSG00000106725	MGI:5663500	Gm43363	predicted gene 43363 [Source:MGI Symbol;Acc:MGI:5663500]
5	TEC	90951120	90954251	ENSMUSG00000106957	MGI:5663222	Gm43085	predicted gene 43085 [Source:MGI Symbol;Acc:MGI:5663222]
5	TEC	91933920	91934741	ENSMUSG00000106712	MGI:5011834	Gm19649	predicted gene, 19649 [Source:MGI Symbol;Acc:MGI:5011834]
5	TEC	92105859	92106821	ENSMUSG00000107200	MGI:5663176	Gm43039	predicted gene 43039 [Source:MGI Symbol;Acc:MGI:5663176]
5	TEC	92339219	92339776	ENSMUSG00000107354	MGI:5663177	Gm43040	predicted gene 43040 [Source:MGI Symbol;Acc:MGI:5663177]
5	TEC	93238844	93241210	ENSMUSG00000107194	MGI:5663310	Gm43173	predicted gene 43173 [Source:MGI Symbol;Acc:MGI:5663310]
5	TEC	93238853	93240989	ENSMUSG00000107374	MGI:5663309	Gm43172	predicted gene 43172 [Source:MGI Symbol;Acc:MGI:5663309]
5	TEC	93324829	93326918	ENSMUSG00000107019	MGI:5663819	Gm43682	predicted gene 43682 [Source:MGI Symbol;Acc:MGI:5663819]
5	TEC	93331715	93333617	ENSMUSG00000106870	MGI:5663818	Gm43681	predicted gene 43681 [Source:MGI Symbol;Acc:MGI:5663818]
5	TEC	96633625	96634009	ENSMUSG00000105446	MGI:5662742	Gm42605	predicted gene 42605 [Source:MGI Symbol;Acc:MGI:5662742]
5	TEC	96946066	96948211	ENSMUSG00000105356	MGI:5662740	Gm42603	predicted gene 42603 [Source:MGI Symbol;Acc:MGI:5662740]
5	TEC	97027377	97030600	ENSMUSG00000104698	MGI:5662739	Gm42602	predicted gene 42602 [Source:MGI Symbol;Acc:MGI:5662739]
5	TEC	97060559	97061100	ENSMUSG00000105871	MGI:5663284	Gm43147	predicted gene 43147 [Source:MGI Symbol;Acc:MGI:5663284]
5	TEC	97084672	97085191	ENSMUSG00000105377	MGI:5663285	Gm43148	predicted gene 43148 [Source:MGI Symbol;Acc:MGI:5663285]
5	TEC	97088156	97088640	ENSMUSG00000105257	MGI:5663281	Gm43144	predicted gene 43144 [Source:MGI Symbol;Acc:MGI:5663281]

5	TEC	97194040	97194667	ENSMUSG00000104591	MGI:5663282	Gm43145	predicted gene 43145 [Source:MGI Symbol;Acc:MGI:5663282]
5	TEC	98147243	98148809	ENSMUSG00000105801	MGI:3028058	C430019N01Rik	RIKEN cDNA C430019N01 gene [Source:MGI Symbol;Acc:MGI:3028058]
5	TEC	98179426	98181864	ENSMUSG00000106518	MGI:5662896	Gm42759	predicted gene 42759 [Source:MGI Symbol;Acc:MGI:5662896]
5	TEC	98494253	98497183	ENSMUSG00000104545	MGI:2442014	E030032P16Rik	RIKEN cDNA E030032P16 gene [Source:MGI Symbol;Acc:MGI:2442014]
5	TEC	1.01E+08	1.01E+08	ENSMUSG00000106391	MGI:5662827	Gm42690	predicted gene 42690 [Source:MGI Symbol;Acc:MGI:5662827]
5	TEC	1.01E+08	1.01E+08	ENSMUSG00000104761	MGI:5663648	Gm43511	predicted gene 43511 [Source:MGI Symbol;Acc:MGI:5663648]
5	TEC	1.01E+08	1.01E+08	ENSMUSG00000105607	MGI:5663650	Gm43513	predicted gene 43513 [Source:MGI Symbol;Acc:MGI:5663650]
5	TEC	1.01E+08	1.01E+08	ENSMUSG00000106178	MGI:5663124	Gm42987	predicted gene 42987 [Source:MGI Symbol;Acc:MGI:5663124]
5	TEC	1.02E+08	1.02E+08	ENSMUSG00000104966	MGI:5663410	Gm43273	predicted gene 43273 [Source:MGI Symbol;Acc:MGI:5663410]
5	TEC	1.02E+08	1.02E+08	ENSMUSG00000105632	MGI:5663409	Gm43272	predicted gene 43272 [Source:MGI Symbol;Acc:MGI:5663409]
5	TEC	1.02E+08	1.02E+08	ENSMUSG00000106451	MGI:5663071	Gm42934	predicted gene 42934 [Source:MGI Symbol;Acc:MGI:5663071]
5	TEC	1.02E+08	1.02E+08	ENSMUSG00000105761	MGI:5663924	Gm43787	predicted gene 43787 [Source:MGI Symbol;Acc:MGI:5663924]
5	TEC	1.03E+08	1.03E+08	ENSMUSG00000105947	MGI:5663926	Gm43789	predicted gene 43789 [Source:MGI Symbol;Acc:MGI:5663926]
5	TEC	1.03E+08	1.03E+08	ENSMUSG00000104808	MGI:5662756	Gm42619	predicted gene 42619 [Source:MGI Symbol;Acc:MGI:5662756]
5	TEC	1.04E+08	1.04E+08	ENSMUSG00000105018	MGI:5663683	Gm43546	predicted gene 43546 [Source:MGI Symbol;Acc:MGI:5663683]
5	TEC	1.04E+08	1.04E+08	ENSMUSG00000105019	MGI:5663684	Gm43547	predicted gene 43547 [Source:MGI Symbol;Acc:MGI:5663684]
5	TEC	1.04E+08	1.04E+08	ENSMUSG00000106035	MGI:5663682	Gm43545	predicted gene 43545 [Source:MGI Symbol;Acc:MGI:5663682]
5	TEC	1.04E+08	1.04E+08	ENSMUSG00000105261	MGI:5663470	Gm43333	predicted gene 43333 [Source:MGI Symbol;Acc:MGI:5663470]

5	TEC	1.05E+08	1.05E+08	ENSMUSG00000105954	MGI:5662930	Gm42793	predicted gene 42793 [Source:MGI Symbol;Acc:MGI:5662930]
5	TEC	1.05E+08	1.05E+08	ENSMUSG00000105555	MGI:5663993	Gm43856	predicted gene 43856 [Source:MGI Symbol;Acc:MGI:5663993]
5	TEC	1.06E+08	1.06E+08	ENSMUSG00000105826	MGI:5625026	Gm42141	predicted gene, 42141 [Source:MGI Symbol;Acc:MGI:5625026]
5	TEC	1.06E+08	1.06E+08	ENSMUSG00000105341	MGI:5663954	Gm43817	predicted gene 43817 [Source:MGI Symbol;Acc:MGI:5663954]
5	TEC	1.07E+08	1.07E+08	ENSMUSG00000104706	MGI:5663558	Gm43421	predicted gene 43421 [Source:MGI Symbol;Acc:MGI:5663558]
5	TEC	1.07E+08	1.07E+08	ENSMUSG00000106643	MGI:5663559	Gm43422	predicted gene 43422 [Source:MGI Symbol;Acc:MGI:5663559]
5	TEC	1.07E+08	1.07E+08	ENSMUSG00000105033	MGI:5663560	Gm43423	predicted gene 43423 [Source:MGI Symbol;Acc:MGI:5663560]
5	TEC	1.07E+08	1.07E+08	ENSMUSG00000106671	MGI:5663037	Gm42900	predicted gene 42900 [Source:MGI Symbol;Acc:MGI:5663037]
5	TEC	1.08E+08	1.08E+08	ENSMUSG00000105421	MGI:1923267	5830411K02Rik	RIKEN cDNA 5830411K02 gene [Source:MGI Symbol;Acc:MGI:1923267]
5	TEC	1.08E+08	1.08E+08	ENSMUSG00000106288	MGI:5663730	Gm43593	predicted gene 43593 [Source:MGI Symbol;Acc:MGI:5663730]
5	TEC	1.08E+08	1.08E+08	ENSMUSG00000104748	MGI:5663729	Gm43592	predicted gene 43592 [Source:MGI Symbol;Acc:MGI:5663729]
5	TEC	1.08E+08	1.08E+08	ENSMUSG00000106103	MGI:5663218	Gm43081	predicted gene 43081 [Source:MGI Symbol;Acc:MGI:5663218]
5	TEC	1.09E+08	1.09E+08	ENSMUSG00000105590	MGI:5663501	Gm43364	predicted gene 43364 [Source:MGI Symbol;Acc:MGI:5663501]
5	TEC	1.09E+08	1.09E+08	ENSMUSG00000105084	MGI:5663502	Gm43365	predicted gene 43365 [Source:MGI Symbol;Acc:MGI:5663502]
5	TEC	1.09E+08	1.09E+08	ENSMUSG00000103580	MGI:3642677	Gm10417	predicted gene 10417 [Source:MGI Symbol;Acc:MGI:3642677]
5	TEC	1.10E+08	1.10E+08	ENSMUSG00000105044	MGI:3645037	Gm10416	predicted pseudogene 10416 [Source:MGI Symbol;Acc:MGI:3645037]
5	TEC	1.11E+08	1.11E+08	ENSMUSG00000105112	MGI:5662915	Gm42778	predicted gene 42778 [Source:MGI Symbol;Acc:MGI:5662915]
5	TEC	1.11E+08	1.11E+08	ENSMUSG00000105890	MGI:5662917	Gm42780	predicted gene 42780 [Source:MGI Symbol;Acc:MGI:5662917]

5	TEC	1.11E+08	1.11E+08	ENSMUSG00000106153	MGI:5663814	Gm43677	predicted gene 43677 [Source:MGI Symbol;Acc:MGI:5663814]
5	TEC	1.11E+08	1.11E+08	ENSMUSG00000105027	MGI:5663813	Gm43676	predicted gene 43676 [Source:MGI Symbol;Acc:MGI:5663813]
5	TEC	1.11E+08	1.11E+08	ENSMUSG00000106374	MGI:5531062	Gm27680	predicted gene, 27680 [Source:MGI Symbol;Acc:MGI:5531062]
5	TEC	1.12E+08	1.12E+08	ENSMUSG00000106560	MGI:5663256	Gm43119	predicted gene 43119 [Source:MGI Symbol;Acc:MGI:5663256]
5	TEC	1.13E+08	1.13E+08	ENSMUSG00000105368	MGI:5663896	Gm43759	predicted gene 43759 [Source:MGI Symbol;Acc:MGI:5663896]
5	TEC	1.13E+08	1.13E+08	ENSMUSG00000104885	MGI:5663895	Gm43758	predicted gene 43758 [Source:MGI Symbol;Acc:MGI:5663895]
5	TEC	1.13E+08	1.13E+08	ENSMUSG00000105735	MGI:1918422	4933415J04Rik	RIKEN cDNA 4933415J04 gene [Source:MGI Symbol;Acc:MGI:1918422]
5	TEC	1.13E+08	1.13E+08	ENSMUSG00000106541	MGI:5663383	Gm43246	predicted gene 43246 [Source:MGI Symbol;Acc:MGI:5663383]
5	TEC	1.13E+08	1.13E+08	ENSMUSG00000104576	MGI:3642056	F830115B05Rik	RIKEN cDNA F830115B05 gene [Source:MGI Symbol;Acc:MGI:3642056]
5	TEC	1.13E+08	1.13E+08	ENSMUSG00000106191	MGI:1922593	4930557B06Rik	RIKEN cDNA 4930557B06 gene [Source:MGI Symbol;Acc:MGI:1922593]
5	TEC	1.13E+08	1.13E+08	ENSMUSG00000106206	MGI:5663231	Gm43094	predicted gene 43094 [Source:MGI Symbol;Acc:MGI:5663231]
5	TEC	1.14E+08	1.14E+08	ENSMUSG00000106623	MGI:5663472	Gm43335	predicted gene 43335 [Source:MGI Symbol;Acc:MGI:5663472]
5	TEC	1.14E+08	1.14E+08	ENSMUSG00000105071	MGI:5663473	Gm43336	predicted gene 43336 [Source:MGI Symbol;Acc:MGI:5663473]
5	TEC	1.14E+08	1.14E+08	ENSMUSG00000105514	MGI:5663050	Gm42913	predicted gene 42913 [Source:MGI Symbol;Acc:MGI:5663050]
5	TEC	1.15E+08	1.15E+08	ENSMUSG00000107256	MGI:3652003	Gm13821	predicted gene 13821 [Source:MGI Symbol;Acc:MGI:3652003]
5	TEC	1.15E+08	1.15E+08	ENSMUSG00000106838	MGI:1916294	1810017P11Rik	RIKEN cDNA 1810017P11 gene [Source:MGI Symbol;Acc:MGI:1916294]
5	TEC	1.15E+08	1.15E+08	ENSMUSG00000107054	MGI:3026961	C730045M19Rik	RIKEN cDNA C730045M19 gene [Source:MGI Symbol;Acc:MGI:3026961]
5	TEC	1.16E+08	1.16E+08	ENSMUSG00000106887	MGI:3528178	A930005G22Rik	RIKEN cDNA A930005G22 gene [Source:MGI Symbol;Acc:MGI:3528178]

5	TEC	1.16E+08	1.16E+08	ENSMUSG00000107240	MGI:5663368	Gm43231	predicted gene 43231 [Source:MGI Symbol;Acc:MGI:5663368]
5	TEC	1.16E+08	1.16E+08	ENSMUSG00000106869	MGI:5662610	Gm42473	predicted gene 42473 [Source:MGI Symbol;Acc:MGI:5662610]
5	TEC	1.16E+08	1.16E+08	ENSMUSG00000107263	MGI:5662608	Gm42471	predicted gene 42471 [Source:MGI Symbol;Acc:MGI:5662608]
5	TEC	1.17E+08	1.17E+08	ENSMUSG00000106766	MGI:1914014	4933424N20Rik	RIKEN cDNA 4933424N20 gene [Source:MGI Symbol;Acc:MGI:1914014]
5	TEC	1.17E+08	1.17E+08	ENSMUSG00000107021	MGI:5662990	Gm42853	predicted gene 42853 [Source:MGI Symbol;Acc:MGI:5662990]
5	TEC	1.17E+08	1.17E+08	ENSMUSG00000107172	MGI:1918558	4933430H06Rik	RIKEN cDNA 4933430H06 gene [Source:MGI Symbol;Acc:MGI:1918558]
5	TEC	1.17E+08	1.17E+08	ENSMUSG00000041512	MGI:1925305	4930569F06Rik	RIKEN cDNA 4930569F06 gene [Source:MGI Symbol;Acc:MGI:1925305]
5	TEC	1.17E+08	1.17E+08	ENSMUSG00000107225	MGI:5663774	Gm43637	predicted gene 43637 [Source:MGI Symbol;Acc:MGI:5663774]
5	TEC	1.17E+08	1.17E+08	ENSMUSG00000106714	MGI:5662683	Gm42546	predicted gene 42546 [Source:MGI Symbol;Acc:MGI:5662683]
5	TEC	1.18E+08	1.18E+08	ENSMUSG00000107199	MGI:5662687	Gm42550	predicted gene 42550 [Source:MGI Symbol;Acc:MGI:5662687]
5	TEC	1.18E+08	1.18E+08	ENSMUSG00000104071	MGI:5610256	Gm37028	predicted gene, 37028 [Source:MGI Symbol;Acc:MGI:5610256]
5	TEC	1.18E+08	1.18E+08	ENSMUSG00000107058	MGI:5662682	Gm42545	predicted gene 42545 [Source:MGI Symbol;Acc:MGI:5662682]
5	TEC	1.19E+08	1.19E+08	ENSMUSG00000106912	MGI:5663922	Gm43785	predicted gene 43785 [Source:MGI Symbol;Acc:MGI:5663922]
5	TEC	1.19E+08	1.19E+08	ENSMUSG00000107286	MGI:5663925	Gm43788	predicted gene 43788 [Source:MGI Symbol;Acc:MGI:5663925]
5	TEC	1.19E+08	1.19E+08	ENSMUSG00000106798	MGI:5663412	Gm43275	predicted gene 43275 [Source:MGI Symbol;Acc:MGI:5663412]
5	TEC	1.19E+08	1.19E+08	ENSMUSG00000107116	MGI:5663411	Gm43274	predicted gene 43274 [Source:MGI Symbol;Acc:MGI:5663411]
5	TEC	1.19E+08	1.19E+08	ENSMUSG00000107115	MGI:5663189	Gm43052	predicted gene 43052 [Source:MGI Symbol;Acc:MGI:5663189]
5	TEC	1.19E+08	1.19E+08	ENSMUSG00000107364	MGI:5663188	Gm43051	predicted gene 43051 [Source:MGI Symbol;Acc:MGI:5663188]

5	TEC	1.19E+08	1.19E+08	ENSMUSG00000107371	MGI:5663921	Gm43784	predicted gene 43784 [Source:MGI Symbol;Acc:MGI:5663921]
5	TEC	1.20E+08	1.20E+08	ENSMUSG00000106915	MGI:5662792	Gm42655	predicted gene 42655 [Source:MGI Symbol;Acc:MGI:5662792]
5	TEC	1.21E+08	1.21E+08	ENSMUSG00000106825	MGI:1917539	2510016D11Rik	RIKEN cDNA 2510016D11 gene [Source:MGI Symbol;Acc:MGI:1917539]
5	TEC	1.21E+08	1.21E+08	ENSMUSG00000107075	MGI:5663205	Gm43068	predicted gene 43068 [Source:MGI Symbol;Acc:MGI:5663205]
5	TEC	1.21E+08	1.21E+08	ENSMUSG00000106841	MGI:5662793	Gm42656	predicted gene 42656 [Source:MGI Symbol;Acc:MGI:5662793]
5	TEC	1.21E+08	1.21E+08	ENSMUSG00000106940	MGI:5663067	Gm42930	predicted gene 42930 [Source:MGI Symbol;Acc:MGI:5663067]
5	TEC	1.22E+08	1.22E+08	ENSMUSG00000106115	MGI:5663557	Gm43420	predicted gene 43420 [Source:MGI Symbol;Acc:MGI:5663557]
5	TEC	1.22E+08	1.22E+08	ENSMUSG00000104999	MGI:1922233	4930477O15Rik	RIKEN cDNA 4930477O15 gene [Source:MGI Symbol;Acc:MGI:1922233]
5	TEC	1.22E+08	1.22E+08	ENSMUSG00000106590	MGI:5663016	Gm42879	predicted gene 42879 [Source:MGI Symbol;Acc:MGI:5663016]
5	TEC	1.22E+08	1.22E+08	ENSMUSG00000106110	MGI:5663708	Gm43571	predicted gene 43571 [Source:MGI Symbol;Acc:MGI:5663708]
5	TEC	1.22E+08	1.22E+08	ENSMUSG00000105179	MGI:5663003	Gm42866	predicted gene 42866 [Source:MGI Symbol;Acc:MGI:5663003]
5	TEC	1.22E+08	1.22E+08	ENSMUSG00000104719	MGI:1918553	4933437G19Rik	RIKEN cDNA 4933437G19 gene [Source:MGI Symbol;Acc:MGI:1918553]
5	TEC	1.22E+08	1.22E+08	ENSMUSG00000105742	MGI:5662885	Gm42748	predicted gene 42748 [Source:MGI Symbol;Acc:MGI:5662885]
5	TEC	1.23E+08	1.23E+08	ENSMUSG00000104916	MGI:5662967	Gm42830	predicted gene 42830 [Source:MGI Symbol;Acc:MGI:5662967]
5	TEC	1.23E+08	1.23E+08	ENSMUSG00000105434	MGI:5663496	Gm43359	predicted gene 43359 [Source:MGI Symbol;Acc:MGI:5663496]
5	TEC	1.23E+08	1.23E+08	ENSMUSG00000105970	MGI:5663497	Gm43360	predicted gene 43360 [Source:MGI Symbol;Acc:MGI:5663497]
5	TEC	1.23E+08	1.23E+08	ENSMUSG00000106375	MGI:5663498	Gm43361	predicted gene 43361 [Source:MGI Symbol;Acc:MGI:5663498]
5	TEC	1.23E+08	1.23E+08	ENSMUSG00000105815	MGI:5663678	Gm43541	predicted gene 43541 [Source:MGI Symbol;Acc:MGI:5663678]

5	TEC	1.23E+08	1.23E+08	ENSMUSG00000105613	MGI:5663821	Gm43684	predicted gene 43684 [Source:MGI Symbol;Acc:MGI:5663821]
5	TEC	1.23E+08	1.23E+08	ENSMUSG00000105979	MGI:5663548	Gm43411	predicted gene 43411 [Source:MGI Symbol;Acc:MGI:5663548]
5	TEC	1.23E+08	1.23E+08	ENSMUSG00000105107	MGI:5663549	Gm43412	predicted gene 43412 [Source:MGI Symbol;Acc:MGI:5663549]
5	TEC	1.23E+08	1.23E+08	ENSMUSG00000104951	MGI:5663550	Gm43413	predicted gene 43413 [Source:MGI Symbol;Acc:MGI:5663550]
5	TEC	1.24E+08	1.24E+08	ENSMUSG00000106254	MGI:5663044	Gm42907	predicted gene 42907 [Source:MGI Symbol;Acc:MGI:5663044]
5	TEC	1.24E+08	1.24E+08	ENSMUSG00000104940	MGI:5663045	Gm42908	predicted gene 42908 [Source:MGI Symbol;Acc:MGI:5663045]
5	TEC	1.24E+08	1.24E+08	ENSMUSG00000104704	MGI:5663270	Gm43133	predicted gene 43133 [Source:MGI Symbol;Acc:MGI:5663270]
5	TEC	1.24E+08	1.24E+08	ENSMUSG00000104877	MGI:5662788	Gm42651	predicted gene 42651 [Source:MGI Symbol;Acc:MGI:5662788]
5	TEC	1.24E+08	1.24E+08	ENSMUSG00000104694	MGI:5663934	Gm43797	predicted gene 43797 [Source:MGI Symbol;Acc:MGI:5663934]
5	TEC	1.24E+08	1.24E+08	ENSMUSG00000102725	MGI:5611167	Gm37939	predicted gene, 37939 [Source:MGI Symbol;Acc:MGI:5611167]
5	TEC	1.24E+08	1.24E+08	ENSMUSG00000105382	MGI:5663476	Gm43339	predicted gene 43339 [Source:MGI Symbol;Acc:MGI:5663476]
5	TEC	1.24E+08	1.24E+08	ENSMUSG00000105228	MGI:5663477	Gm43340	predicted gene 43340 [Source:MGI Symbol;Acc:MGI:5663477]
5	TEC	1.25E+08	1.25E+08	ENSMUSG00000103864	MGI:5610643	Gm37415	predicted gene, 37415 [Source:MGI Symbol;Acc:MGI:5610643]
5	TEC	1.25E+08	1.25E+08	ENSMUSG00000106662	MGI:5663171	Gm43034	predicted gene 43034 [Source:MGI Symbol;Acc:MGI:5663171]
5	TEC	1.25E+08	1.25E+08	ENSMUSG00000106458	MGI:5662770	Gm42633	predicted gene 42633 [Source:MGI Symbol;Acc:MGI:5662770]
5	TEC	1.26E+08	1.26E+08	ENSMUSG00000104793	MGI:5663893	Gm43756	predicted gene 43756 [Source:MGI Symbol;Acc:MGI:5663893]
5	transcribed_processed_pseudogene	44101245	44101824	ENSMUSG00000059565	MGI:3645663	Gm5292	predicted gene 5292 [Source:MGI Symbol;Acc:MGI:3645663]

5	transcribed_processed_pseudogene	45926209	45927085	ENSMUSG00000101587	MGI:5579742	Gm29036	predicted gene 29036 [Source:MGI Symbol;Acc:MGI:5579742]
5	transcribed_processed_pseudogene	46571686	46571956	ENSMUSG00000067321	MGI:3643448	Gm7931	predicted pseudogene 7931 [Source:MGI Symbol;Acc:MGI:3643448]
5	transcribed_processed_pseudogene	50674815	50675556	ENSMUSG00000106345	MGI:5662596	Gm42459	predicted gene 42459 [Source:MGI Symbol;Acc:MGI:5662596]
5	transcribed_processed_pseudogene	50837058	50840034	ENSMUSG00000106038	MGI:3643111	Gm4962	predicted gene 4962 [Source:MGI Symbol;Acc:MGI:3643111]
5	transcribed_processed_pseudogene	56571552	56647574	ENSMUSG00000106326	MGI:5663114	Gm42977	predicted gene 42977 [Source:MGI Symbol;Acc:MGI:5663114]
5	transcribed_processed_pseudogene	58057076	58057649	ENSMUSG00000104564	MGI:5010162	Gm17977	predicted gene, 17977 [Source:MGI Symbol;Acc:MGI:5010162]
5	transcribed_processed_pseudogene	66332308	66332686	ENSMUSG00000107069	MGI:5663909	Gm43772	predicted gene 43772 [Source:MGI Symbol;Acc:MGI:5663909]
5	transcribed_processed_pseudogene	67610822	67611113	ENSMUSG00000106925	MGI:5012257	Gm20072	predicted gene, 20072 [Source:MGI Symbol;Acc:MGI:5012257]
5	transcribed_processed_pseudogene	72784201	72785371	ENSMUSG00000105124	MGI:3646414	Gm5297	predicted gene 5297 [Source:MGI Symbol;Acc:MGI:3646414]

5	transcribed_processed_pseudogene	73547992	73548445	ENSMUSG00000107272	MGI:5593742	Gm34583	predicted gene, 34583 [Source:MGI Symbol;Acc:MGI:5593742]
5	transcribed_processed_pseudogene	73633790	73634696	ENSMUSG00000082039	MGI:3783097	Gm15653	predicted gene 15653 [Source:MGI Symbol;Acc:MGI:3783097]
5	transcribed_processed_pseudogene	74477239	74477695	ENSMUSG00000080700	MGI:3801723	Gm15981	predicted gene 15981 [Source:MGI Symbol;Acc:MGI:3801723]
5	transcribed_processed_pseudogene	75503104	75504493	ENSMUSG00000106689	MGI:5010530	Gm18345	predicted gene, 18345 [Source:MGI Symbol;Acc:MGI:5010530]
5	transcribed_processed_pseudogene	76344472	76344941	ENSMUSG00000083152	MGI:1930261	Apc-ps1	adenomatosis polyposis coli, pseudogene 1 [Source:MGI Symbol;Acc:MGI:1930261]
5	transcribed_processed_pseudogene	77659025	77659411	ENSMUSG00000047652	MGI:3525016	Pea15b-ps	proliferation and apoptosis adaptor protein 15B, pseudogene [Source:MGI Symbol;Acc:MGI:3525016]
5	transcribed_processed_pseudogene	84271172	84271450	ENSMUSG00000079067	MGI:3783162	Hmgn2-ps1	high mobility group nucleosomal binding domain 2, pseudogene 1 [Source:MGI Symbol;Acc:MGI:3783162]
5	transcribed_processed_pseudogene	86214616	86215615	ENSMUSG00000106282	MGI:3780458	Gm2287	predicted gene 2287 [Source:MGI Symbol;Acc:MGI:3780458]
5	transcribed_processed_pseudogene	87107980	87109573	ENSMUSG00000104798	MGI:5012306	Gm20121	predicted gene, 20121 [Source:MGI Symbol;Acc:MGI:5012306]

5	transcribed_processed_pseudogene	87998377	87999501	ENSMUSG00000067156	MGI:3646689	Gm7337	predicted gene 7337 [Source:MGI Symbol;Acc:MGI:3646689]
5	transcribed_processed_pseudogene	88358518	88358896	ENSMUSG00000105576	MGI:5625049	Gm42164	predicted gene, 42164 [Source:MGI Symbol;Acc:MGI:5625049]
5	transcribed_processed_pseudogene	91908555	91909434	ENSMUSG00000106917	MGI:3779767	Gm7832	predicted gene 7832 [Source:MGI Symbol;Acc:MGI:3779767]
5	transcribed_processed_pseudogene	1.01E+08	1.01E+08	ENSMUSG00000104993	MGI:5663647	Gm43510	predicted gene 43510 [Source:MGI Symbol;Acc:MGI:5663647]
5	transcribed_processed_pseudogene	1.08E+08	1.08E+08	ENSMUSG00000084855	MGI:3642875	Gm9850	predicted gene 9850 [Source:MGI Symbol;Acc:MGI:3642875]
5	transcribed_processed_pseudogene	1.15E+08	1.15E+08	ENSMUSG00000072692	MGI:3651519	Rpl37rt	ribosomal protein L37, retrotransposed [Source:MGI Symbol;Acc:MGI:3651519]
5	transcribed_processed_pseudogene	1.16E+08	1.16E+08	ENSMUSG00000081282	MGI:3650922	Gm13836	predicted gene 13836 [Source:MGI Symbol;Acc:MGI:3650922]
5	transcribed_processed_pseudogene	1.19E+08	1.19E+08	ENSMUSG00000082365	MGI:3783197	Btf3-ps11	basic transcription factor 3, pseudogene 11 [Source:MGI Symbol;Acc:MGI:3783197]
5	transcribed_processed_pseudogene	1.26E+08	1.26E+08	ENSMUSG00000106271	MGI:5663889	Gm43752	predicted gene 43752 [Source:MGI Symbol;Acc:MGI:5663889]

5	transcribed_processed_pseudogene	1.26E+08	1.26E+08	ENSMUSG00000091970	MGI:3648712	Gm4868	predicted gene 4868 [Source:MGI Symbol;Acc:MGI:3648712]
5	transcribed_unitary_pseudogene	73170871	73174579	ENSMUSG00000081849	MGI:3783237	Zar1-ps	zygote arrest 1, pseudogene [Source:MGI Symbol;Acc:MGI:3783237]
5	transcribed_unitary_pseudogene	92111565	92117654	ENSMUSG00000102644	MGI:1922436	Thap6	THAP domain containing 6 [Source:MGI Symbol;Acc:MGI:1922436]
5	transcribed_unitary_pseudogene	1.00E+08	1.01E+08	ENSMUSG00000085915	MGI:3643169	Gm8091	predicted gene 8091 [Source:MGI Symbol;Acc:MGI:3643169]
5	transcribed_unitary_pseudogene	1.09E+08	1.09E+08	ENSMUSG00000118523	MGI:5593478	Gm34319	predicted gene, 34319 [Source:MGI Symbol;Acc:MGI:5593478]
5	transcribed_unprocessed_pseudogene	50374589	50548703	ENSMUSG00000105020	MGI:3643966	Gm7205	predicted gene 7205 [Source:MGI Symbol;Acc:MGI:3643966]
5	transcribed_unprocessed_pseudogene	74517244	74519080	ENSMUSG00000084132	MGI:3801722	Gm15982	predicted gene 15982 [Source:MGI Symbol;Acc:MGI:3801722]
5	transcribed_unprocessed_pseudogene	77076219	77095456	ENSMUSG00000072852	MGI:1917534	2310040G07Rik	RIKEN cDNA 2310040G07 gene [Source:MGI Symbol;Acc:MGI:1917534]

5	transcribed_unprocessed_pseudogene	87524017	87527779	ENSMUSG00000106397	MGI:5434404	Gm21049	predicted gene, 21049 [Source:MGI Symbol;Acc:MGI:5434404]
5	transcribed_unprocessed_pseudogene	88344847	88349266	ENSMUSG00000106065	MGI:5662758	Gm42621	predicted gene 42621 [Source:MGI Symbol;Acc:MGI:5662758]
5	transcribed_unprocessed_pseudogene	94450679	94453474	ENSMUSG00000094560	MGI:2683305	Pramel39-ps	PRAME like 39, pseudogene [Source:MGI Symbol;Acc:MGI:2683305]
5	transcribed_unprocessed_pseudogene	94529232	94531954	ENSMUSG00000073497	MGI:2140789	Pramel52-ps	PRAME like 52, pseudogene [Source:MGI Symbol;Acc:MGI:2140789]
5	transcribed_unprocessed_pseudogene	95170764	95173493	ENSMUSG00000096742	MGI:3704106	Pramel59	PRAME like 59 [Source:MGI Symbol;Acc:MGI:3704106]
5	transcribed_unprocessed_pseudogene	95428321	95431051	ENSMUSG00000107348	MGI:3646666	Gm6468	predicted gene 6468 [Source:MGI Symbol;Acc:MGI:3646666]
5	transcribed_unprocessed_pseudogene	95750437	95753167	ENSMUSG00000106690	MGI:3643967	Gm6348	predicted gene 6348 [Source:MGI Symbol;Acc:MGI:3643967]

5	transcribed_unprocessed_pseudogene	1.05E+08	1.05E+08	ENSMUSG00000118625	MGI:6846487	Gm55006	predicted gene, 55006 [Source:MGI Symbol;Acc:MGI:6846487]
5	transcribed_unprocessed_pseudogene	1.10E+08	1.10E+08	ENSMUSG00000106028	MGI:3779800	Gm8493	predicted gene 8493 [Source:MGI Symbol;Acc:MGI:3779800]
5	translated_unprocessed_pseudogene	1.10E+08	1.10E+08	ENSMUSG00000093663	MGI:3649028	Gm7171	predicted gene 7171 [Source:MGI Symbol;Acc:MGI:3649028]
5	unitary_pseudogene	43974000	43975191	ENSMUSG00000144294			family with sequence similarity 200, member B (Fam200B) pseudogene
5	unitary_pseudogene	45814290	45814914	ENSMUSG00000105634	MGI:5663504	Gm43367	predicted gene 43367 [Source:MGI Symbol;Acc:MGI:5663504]
5	unitary_pseudogene	65054266	65056312	ENSMUSG00000118495	MGI:5434366	Gm21011	predicted gene, 21011 [Source:MGI Symbol;Acc:MGI:5434366]
5	unitary_pseudogene	87860712	87860762	ENSMUSG00000144277			Statherin/Histatin pseudogene
5	unitary_pseudogene	87901075	87901125	ENSMUSG00000144278			Statherin/Histatin pseudogene
5	unitary_pseudogene	1.01E+08	1.01E+08	ENSMUSG00000144266			THAP domain containing 9 (Thap9) pseudogene
5	unprocessed_pseudogene	58518976	58520854	ENSMUSG00000105269	MGI:5663136	Gm42999	predicted gene 42999 [Source:MGI Symbol;Acc:MGI:5663136]

5	unprocessed_pseudogene	68726847	68752089	ENSMUSG00000106854	MGI:5662987	Gm42850	predicted gene 42850 [Source:MGI Symbol;Acc:MGI:5662987]
5	unprocessed_pseudogene	75302009	75302219	ENSMUSG00000107258	MGI:3031235	Olfr1401-ps1	olfactory receptor 1401, pseudogene 1 [Source:MGI Symbol;Acc:MGI:3031235]
5	unprocessed_pseudogene	77835223	77837059	ENSMUSG00000106996	MGI:5590208	Gm31049	predicted gene, 31049 [Source:MGI Symbol;Acc:MGI:5590208]
5	unprocessed_pseudogene	84011021	84011609	ENSMUSG00000106576	MGI:3646530	Gm4866	predicted gene 4866 [Source:MGI Symbol;Acc:MGI:3646530]
5	unprocessed_pseudogene	87008516	87011365	ENSMUSG00000105309	MGI:5434816	Gm21461	predicted gene, 21461 [Source:MGI Symbol;Acc:MGI:5434816]
5	unprocessed_pseudogene	87124679	87139636	ENSMUSG00000106185	MGI:3644524	Gm7631	predicted gene 7631 [Source:MGI Symbol;Acc:MGI:3644524]
5	unprocessed_pseudogene	87202462	87203524	ENSMUSG00000106003	MGI:5662602	Gm42465	predicted gene 42465 [Source:MGI Symbol;Acc:MGI:5662602]
5	unprocessed_pseudogene	87245041	87248254	ENSMUSG00000105625	MGI:5590389	Gm31230	predicted gene, 31230 [Source:MGI Symbol;Acc:MGI:5590389]
5	unprocessed_pseudogene	87310138	87323149	ENSMUSG00000106111	MGI:3649128	Gm5717	predicted gene 5717 [Source:MGI Symbol;Acc:MGI:3649128]
5	unprocessed_pseudogene	87354976	87365970	ENSMUSG00000105240	MGI:5011603	Gm19418	predicted gene, 19418 [Source:MGI Symbol;Acc:MGI:5011603]
5	unprocessed_pseudogene	87438038	87459676	ENSMUSG00000105897	MGI:3646880	Gm7646	predicted gene 7646 [Source:MGI Symbol;Acc:MGI:3646880]
5	unprocessed_pseudogene	87499339	87512677	ENSMUSG00000106303	MGI:3643731	Gm7652	predicted gene 7652 [Source:MGI Symbol;Acc:MGI:3643731]

5	unprocessed_pseudogene	87549178	87550837	ENSMUSG00000106132	MGI:5663507	Gm43370	predicted gene 43370 [Source:MGI Symbol;Acc:MGI:5663507]
5	unprocessed_pseudogene	93558621	93559719	ENSMUSG00000107356	MGI:5010303	Gm18118	predicted gene, 18118 [Source:MGI Symbol;Acc:MGI:5010303]
5	unprocessed_pseudogene	93569531	93571255	ENSMUSG00000106699	MGI:5663361	Gm43224	predicted gene 43224 [Source:MGI Symbol;Acc:MGI:5663361]
5	unprocessed_pseudogene	93842853	93845580	ENSMUSG00000107346	MGI:5663362	Gm43225	predicted gene 43225 [Source:MGI Symbol;Acc:MGI:5663362]
5	unprocessed_pseudogene	93885955	93888642	ENSMUSG00000107159	MGI:3645426	Gm7919	predicted gene 7919 [Source:MGI Symbol;Acc:MGI:3645426]
5	unprocessed_pseudogene	93918159	93919659	ENSMUSG00000107247	MGI:5663364	Gm43227	predicted gene 43227 [Source:MGI Symbol;Acc:MGI:5663364]
5	unprocessed_pseudogene	94084614	94087344	ENSMUSG00000106701	MGI:3644199	Gm6346	predicted gene 6346 [Source:MGI Symbol;Acc:MGI:3644199]
5	unprocessed_pseudogene	94144340	94147033	ENSMUSG00000106916	MGI:3781265	Gm3089	predicted gene 3089 [Source:MGI Symbol;Acc:MGI:3781265]
5	unprocessed_pseudogene	94582216	94584784	ENSMUSG00000107315	MGI:3770624	BC080696	cDNA sequence BC080696 [Source:MGI Symbol;Acc:MGI:3770624]
5	unprocessed_pseudogene	94830504	94833233	ENSMUSG00000092073	MGI:3779572	Pramel58	PRAME like 58 [Source:MGI Symbol;Acc:MGI:3779572]
5	unprocessed_pseudogene	94881215	94883946	ENSMUSG00000107064	MGI:5662698	Gm42561	predicted gene 42561 [Source:MGI Symbol;Acc:MGI:5662698]
5	unprocessed_pseudogene	94917360	94919898	ENSMUSG00000107187	MGI:3779755	Gm7663	predicted gene 7663 [Source:MGI Symbol;Acc:MGI:3779755]

5	unprocessed_pseudogene	95099396	95101993	ENSMUSG00000107285	MGI:3781355	Gm3176	predicted gene 3176 [Source:MGI Symbol;Acc:MGI:3781355]
5	unprocessed_pseudogene	95110877	95113553	ENSMUSG00000073510	MGI:3779753	Gm7647	predicted gene 7647 [Source:MGI Symbol;Acc:MGI:3779753]
5	unprocessed_pseudogene	95211435	95214166	ENSMUSG00000107376	MGI:5662798	Gm42661	predicted gene 42661 [Source:MGI Symbol;Acc:MGI:5662798]
5	unprocessed_pseudogene	95247883	95250141	ENSMUSG00000106780	MGI:3647260	Gm7939	predicted gene 7939 [Source:MGI Symbol;Acc:MGI:3647260]
5	unprocessed_pseudogene	95528913	95531643	ENSMUSG00000107122	MGI:5591231	Gm32072	predicted gene, 32072 [Source:MGI Symbol;Acc:MGI:5591231]
5	unprocessed_pseudogene	95564969	95567610	ENSMUSG00000106821	MGI:3643212	Gm7961	predicted gene 7961 [Source:MGI Symbol;Acc:MGI:3643212]
5	unprocessed_pseudogene	95573794	95576487	ENSMUSG00000107357	MGI:5662799	Gm42662	predicted gene 42662 [Source:MGI Symbol;Acc:MGI:5662799]
5	unprocessed_pseudogene	95810164	95812857	ENSMUSG00000107213	MGI:3781480	Gm3302	predicted gene 3302 [Source:MGI Symbol;Acc:MGI:3781480]
5	unprocessed_pseudogene	95982820	95985309	ENSMUSG00000106805	MGI:5662851	Gm42714	predicted gene 42714 [Source:MGI Symbol;Acc:MGI:5662851]
5	unprocessed_pseudogene	1.07E+08	1.07E+08	ENSMUSG00000067047	MGI:3030553	Olfr719-ps	olfactory receptor 719, pseudogene [Source:MGI Symbol;Acc:MGI:3030553]
5	unprocessed_pseudogene	1.09E+08	1.09E+08	ENSMUSG00000093510	MGI:3761376	Vmn2r-ps21	vomeronasal 2, receptor, pseudogene 21 [Source:MGI Symbol;Acc:MGI:3761376]
5	unprocessed_pseudogene	1.21E+08	1.21E+08	ENSMUSG00000118628	MGI:3645862	Gm7573	predicted gene 7573 [Source:MGI Symbol;Acc:MGI:3645862]
