# Supplementary material for: Genetic diversity of collaborative cross mice enables the establishment of a novel Chlamydia muridarum female genital tract infection model
Source: Infect Immun. 2026 May 5;94(6):e00746-25. doi: 10.1128/iai.00746-25 (PMC13248643; doi:10.1128/iai.00746-25)
Supplement: Supplemental tables — Tables S6 to S8. [file iai.00746-25-s0003.pdf]

Supplementary Table 6. Cervical mRNAs significantly associated with *C. muridarum* burden at day 3 with Bonferroni corrected P<0.05

Host Target	Estimate	Day3_Pval
Il23a	4.55E-01	4.43E-08
Il1b	3.64E-01	5.31E-08
Il22	3.42E-01	1.92E-07
Cxcl1	4.28E-01	1.99E-07
Ifnb1	4.88E-01	4.30E-07
Il10	3.49E-01	8.14E-07
Il1a	2.98E-01	1.02E-06
Cd40lg	4.45E-01	1.88E-06
Il12a	3.85E-01	6.94E-06
Il17a	4.25E-01	8.24E-06
Stat3	6.07E-01	9.16E-06
Il27	2.95E-01	9.43E-06
Il16	6.03E-01	1.41E-05
Cxcl10	2.85E-01	2.23E-05
Ccl4	3.54E-01	2.41E-05
Eomes	5.12E-01	2.56E-05
Ifng	3.76E-01	3.15E-05
Ccl3	3.36E-01	5.72E-05
Tlr2	4.80E-01	6.33E-05
Il6	2.67E-01	7.00E-05
Il1rn	4.43E-01	2.01E-04
Tnf	4.45E-01	2.11E-04
Il15	3.42E-01	4.79E-04
Il18	4.49E-01	8.69E-04
Gata3	6.74E-01	8.81E-04

Supplementary Table 7. Cervical mRNAs significantly associated with *C. muridarum* burden at day 5 with Bonferroni corrected P<0.05

Host Target	Estimate	Day5_Pval
Il17a	5.11E-01	1.33E-06
Il12a	6.12E-01	1.75E-06
Ifng	5.12E-01	4.24E-06
Il22	5.91E-01	4.96E-05
Il10	5.83E-01	6.05E-05
Ifnb1	5.40E-01	2.11E-04
Cxcl10	3.91E-01	2.67E-04
Eomes	6.33E-01	5.80E-04

Supplementary Table 8. Cervical mRNAs significantly associated with *C. muridarum* burden at day 7 with Bonferroni corrected P<0.05

Host Target	Estimate	Day7_Pval
Cd40lg	1.43E+00	1.41E-07
Ifng	8.63E-01	1.47E-05
Il17a	9.47E-01	1.56E-05
Il22	9.76E-01	3.52E-05
Il10	8.35E-01	5.15E-05
Ltb	1.82E+00	5.64E-05
Il1b	1.07E+00	8.10E-05
Il12a	1.35E+00	1.39E-04
Il27	1.13E+00	3.31E-04
Ifnb1	1.11E+00	5.74E-04
Il16	1.21E+00	8.75E-04
